# Information sharing between intensive care and primary care after an episode of critical illness; A mixed methods analysis

**DOI:** 10.1371/journal.pone.0212438

**Published:** 2019-02-28

**Authors:** Gabor Zilahi, Enda O’Connor

**Affiliations:** 1 Department of Intensive Care and Anaesthesia, Saint James’s Hospital, Dublin, Ireland; 2 School of Medicine, Trinity College Dublin, College Green, Dublin, Ireland; University of Plymouth, UNITED KINGDOM

## Abstract

**Introduction:**

Poor quality communication between hospital doctors and GPs at the time of hospital discharge is associated with adverse patient outcomes. This may be more marked after an episode of critical illness, the complications of which can persist long after hospital discharge.

**Aims:**

1. to evaluate information sharing between ICU staff and GPs after a critical illness

2. to identify factors influencing the flow and utilisation of this information.

**Methods:**

Parallel mixed methods observational study in an Irish setting, with equal emphasis on quantitative and qualitative data. Descriptive analysis was performed on quantitative data derived from GP and ICU consultant questionnaires. Qualitative data came from semi-structured interviews with GPs and consultants, and were analysed using directed content analysis. Mixing of data occurred at the stage of interpretation.

**Results:**

GPs rarely received information about an episode of critical illness directly from ICU staff, with most coming from patients and relatives. Information received from hospital sources was frequently brief and incomplete. Common communication barriers reported by consultants were insufficient time, low perceived importance and difficulty establishing GP contact. When provided information, GPs seldom actioned specific interventions, citing insufficient guidance in hospital correspondence and poor knowledge about critical illness complications and their management. A majority of all respondents thought that improved information sharing would benefit patients. Cultural influences on practice were identified in qualitative data. *A priori* qualitative themes were: (1) perceived benefits of information sharing, (2) factors influencing current practice and (3) strategies for optimal information sharing. Emergent themes were: (4) the central role of the GP in patient care, (5) the concept of the “whole patient journey” and (6) a culture of expectation around a GP’s knowledge of hospital care.

**Conclusions:**

Practical and cultural factors contribute to suboptimal information sharing between ICU and primary care doctors around an episode of critical illness in ICU. We propose a three-milestone strategy to improve the flow and utilisation of information when patients are admitted, discharged or die within the ICU.

## Introduction

The adverse consequences of an episode of critical illness are well recognised. An unplanned admission to an intensive care unit (ICU) is an independent risk factor for mortality and early readmission after hospital discharge [1]. The physical, cognitive and psychological effects of critical illness commonly hinder the resumption of patients’ social and professional activities, creating functional and financial dependence on others [2–6].

Knowledge about the nature of a patient’s critical illness may help stratify their risk of adverse outcomes after hospital discharge [7,8]. For example, in a recent study of over 14 million patients, a diagnosis of sepsis was the strongest predictor of hospital readmission within 30 days [8]. In an analysis of nearly 20,000 patients, severity of illness and the number of organ failures were independently associated with survival after hospital discharge [9].

Hospital medical summaries to general practitioners (GPs) frequently omit key relevant information about a patient’s hospitalisation and their expected clinical trajectory [10,11]. There is evidence that these omissions contribute to adverse outcomes after hospital discharge [12,13]. Philbert, in an overview of the HANDOVER project showed that direct communication between hospital physicians and GPs was rare and that, in 25% of patients, delayed or missing inpatient information contributed to poor quality of care after hospital discharge [14]. Efforts to improve information transfer at the hospital-to-community interface have led to reductions in post-discharge adverse events [15,16], though not in patients recovering from critical illness.

Several small studies evaluate the transfer of patient information to GPs following an unplanned admission to an ICU. They demonstrate a paucity of clinical information, either directly from ICU staff or indirectly from other hospital medical teams [17–19]. Consequently, there is a discrepancy between the clinical information GPs expect and the information they receive about an ICU stay [20]. Patients are seldom discharged directly from an ICU to the community, therefore the importance of sharing information between ICU staff and GPs is unclear.

In Ireland, several factors may hinder the standardisation of effective communication practices about critical illness in the healthcare setting. Adult critical care services in Ireland are spread over a wide geographical area; there are almost 300 adult critical care beds in 37 hospitals serving a population of 4.76 million people. This is further compounded by a low population density, a high proportion of remote rural GPs, and an incomplete national broadband coverage. In an attempt to further understand the sharing of critical illness information in this context, our study had two overarching objectives. First, we sought to evaluate information sharing in Ireland between ICU medical staff and GPs around an episode of critical illness. Second, we sought to explore the factors affecting both the quality of information sharing as well as how the information is utilised by GPs when received.

## Methods

### Setting

The study was conducted in the ICU of St James’s Hospital and Trinity College, Dublin, Ireland from May 2016 to July 2016. Ethics approval was obtained from the School of Medicine Research Ethics Committee.

### Design

We used a parallel mixed methods design with individual collection and analysis of qualitative and quantitative data [21]. We placed equal weight on each type of data in our overall interpretation [22]. Though parallel mixed methods studies usually have simultaneous phases, our qualitative phase was delayed allowing for participant recruitment.

### Participants

All intensive care medicine consultants (ICMCs) in the Intensive Care Society of Ireland database, comprising >90% of ICMCs in Ireland, were invited to participate. Those who had retired, had no current clinical duties in ICM or were working overseas were excluded. Interview participation was by self-selection; consultants who completed the survey were invited to participate by contacting a third party through an email address listed at the end of the survey. A group of 200 GPs was selected by proportionate stratified random sampling from the Irish College of General Practitioners (ICGP) database [23] which includes 85% of GPs in Ireland. Each of the 26 Irish counties was weighted according to its population distribution [24] and a corresponding proportion of the 200 surveys was distributed to a random sample of GPs in each county. For example, a county containing 11.3% of the total population had 23 of the total 200 GPs. An online random number generator was used to select participants from GPs listed numerically on the ICGP website for each county. GPs also volunteered for an interview by emailing a third party. Written informed consent was obtained from all interview participants.

### Data collection

Separate questionnaires were distributed to consultants and GPs, each containing 11 questions (S1 Appendix). To enhance construct validity, the ICMC and GP surveys were pretested by 2 ICU doctors and 2 GPs who were subsequently not involved in the study. During this process, each question was reviewed for clarity, and for the information it was seeking and how this information aligned with the overall research objectives. Consultants received an online questionnaire using Survey Monkey and two subsequent email reminders. Due to an incomplete email database, all GPs were first sent a paper questionnaire. If a GP’s email address was available, an identical online reminder questionnaire was sent. Otherwise, a phone call was placed to the GP practice by a third party enquiring about participation.

Qualitative data derived from 2 sources; from free-text answers in the questionnaires, and from semi-structured interviews with GPs and ICMCs (S2 Appendix). All interviews were moderated by both researchers. Interview questions differed between the 2 participant groups and were piloted prior to use in a similar manner to the survey questions. Each interview contained one open question to allow for the discovery of unanticipated data during the discussions. Quantitative data can be viewed at the following links (S1 Dataset: https://www.surveymonkey.com/results/SM-HC9MLCVTL/ and S2 Dataset https://www.surveymonkey.com/results/SM-L7VZ9CVTL/). Anonymised qualitative data after the first round of coding can be viewed in the S1 File.

### Data analysis and integration

Quantitative data were stored and descriptive analysis performed using Microsoft Excel. Interviews were recorded and transcribed verbatim by an independent third party. Interviewees were invited to review the transcripts for errors and, if appropriate, to recommend changes. Manual coding of the transcripts was independently carried out by each researcher, both trained in qualitative data analysis. A deductive coding strategy (directed content analysis) was used as described by Hsieh whereby the decision about appropriate coding themes preceded the process of data analysis [25]. Selection of these themes was based on previous related literature and on theories underpinning the research construct. Additional open questions in the interviews allowed for some inductive enquiry. We commenced with three *a priori* coding themes derived from published literature (benefits of information sharing; factors influencing current practice; strategies for optimal information sharing) [19,20,26]. Coding began by allocating data (phrases and sentences in the discussions) to each of these three themes. Any data falling outside these were allocated to a miscellaneous category, and subsequently evaluated for any emergent themes. Any divergence or disagreements in coding were resolved by discussion. [25]

In keeping with our convergent study design, mixing of qualitative and quantitative data was done during the process of interpretation [27]. Data were presented using a strategy called “parallel database variant” of convergent study design [28]; individual description of quantitative and qualitative data was followed by comments about the relationships between the two datasets. This enabled the evaluation of convergence and divergence between datasets and revealed insights which would have been less apparent in either dataset alone. For the purposes of the study, information sharing was defined as any contact with a patient’s GP initiated by intensive care staff for the purposes of delivering or requesting patient information.

## Results

Sixty-five of 97 ICMCs, and 97 of 200 GPs answered the survey. The response rates were 67% and 48.5% respectively. Almost all consultants (63/65; 96.9%) worked in a university-affiliated hospital, and 29(44.6%) spent ≧50% of their clinical time working in the ICU. A majority (59/97; 60.1%) of GPs worked in a rural setting, and 21 of the 26 national county regions were represented in the survey responses. 78(80.4%) of GP respondents had worked in primary care for ≧10years. Thirteen volunteers participated in the interviews, 5 ICMCs (working in 5 different ICUs in 4 different cities) and 8 GPs (6 urban and 2 rural practice). For logistical reasons, 4 interviews were conducted by telephone (2 ICMCs; 2 GPs). Interviews were between 17 and 32 minutes duration.

### Quantitative data

#### Methods and content of sharing ICU information

**Fig 1:**
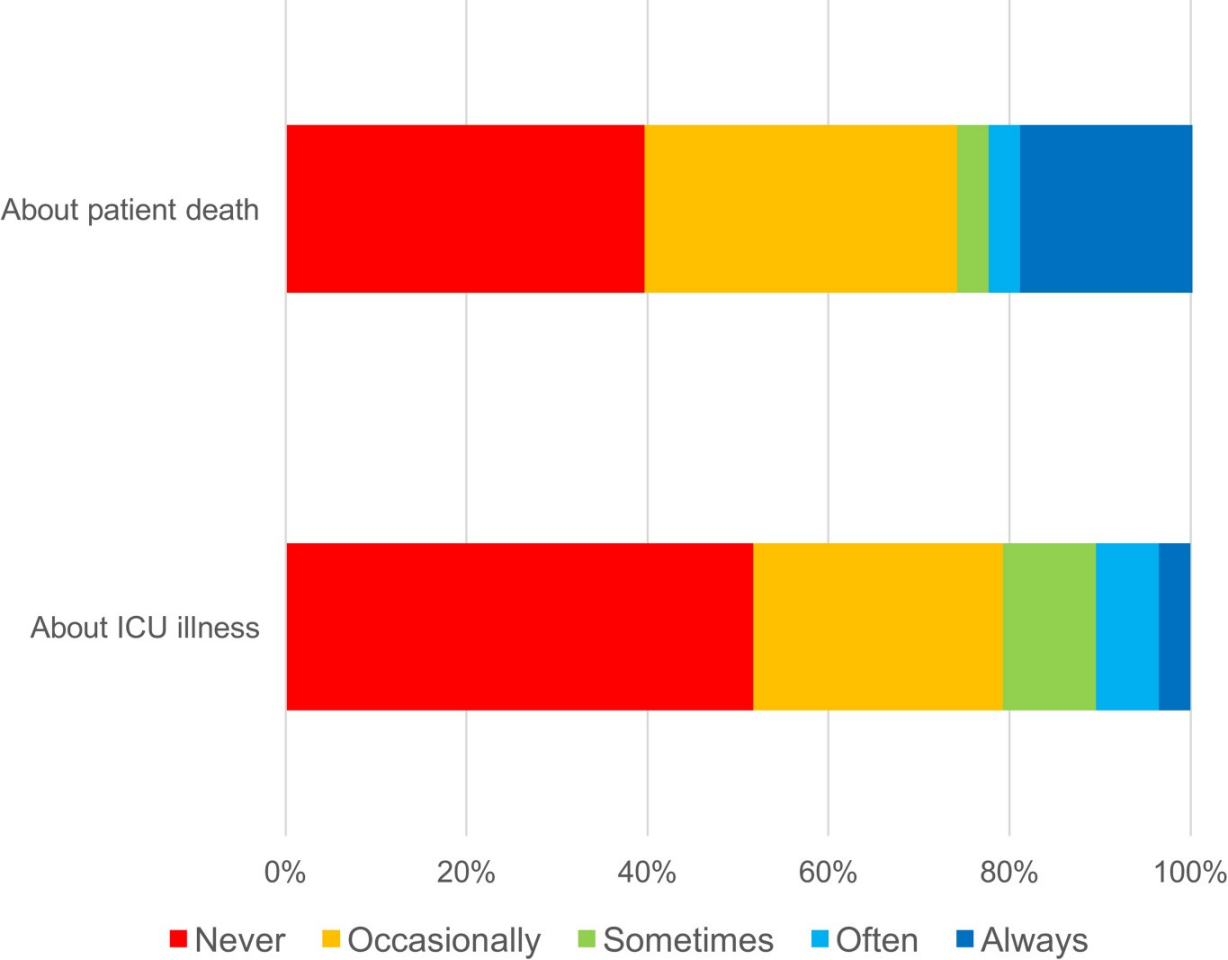
ICM consultant responses about information sharing 1. ICMC responses to question: How often would you (or a member of your ICU team) contact the GP to tell them (a) about patient death in ICU or (b) about details of patient’s ICU illness [%].

**Fig 2:**
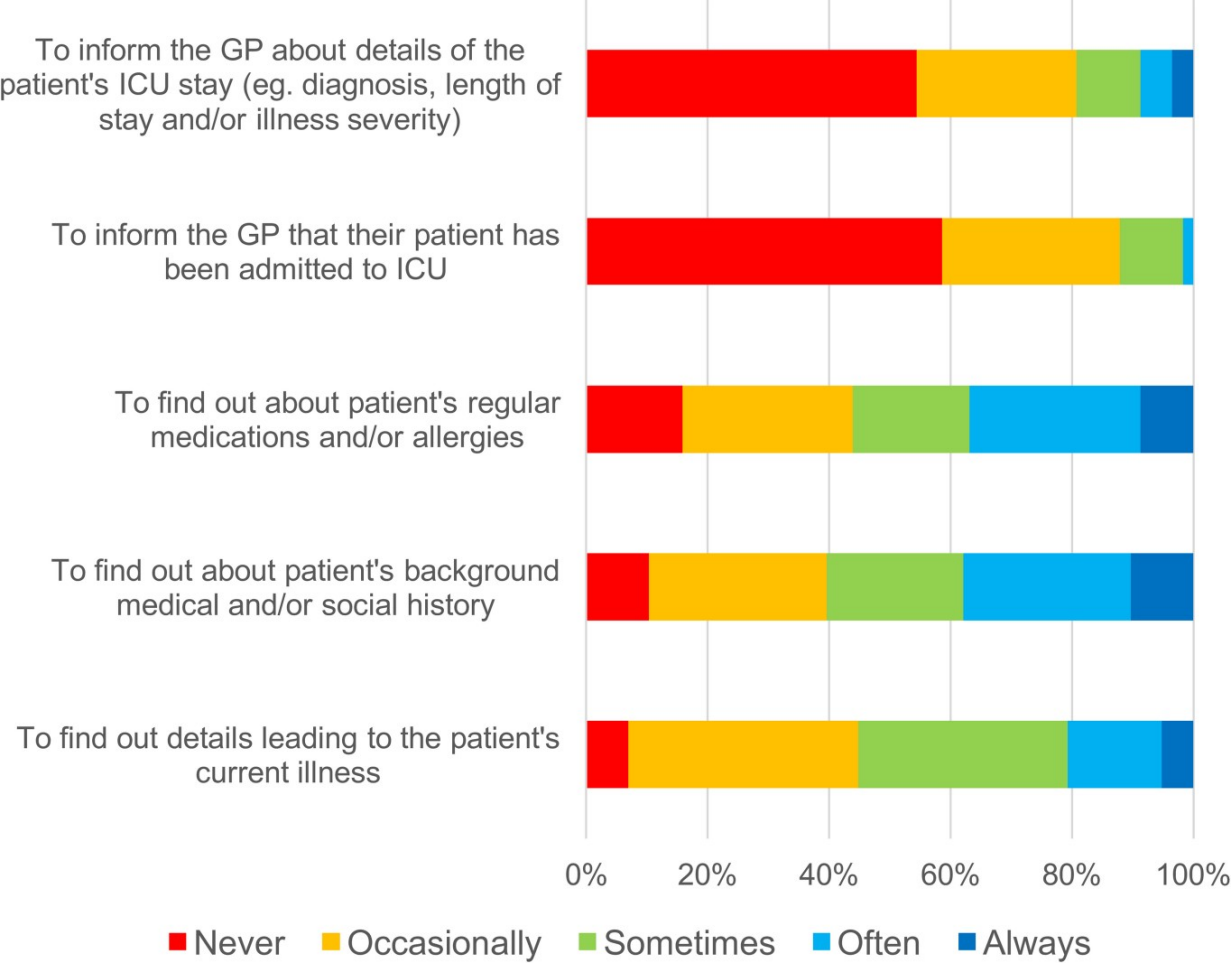
ICM consultant responses about information sharing 2. ICMC responses to question: If you (or a member of your ICU team) make contact with a patient's GP, what is the purpose of this communication? [%].

**Fig 3:**
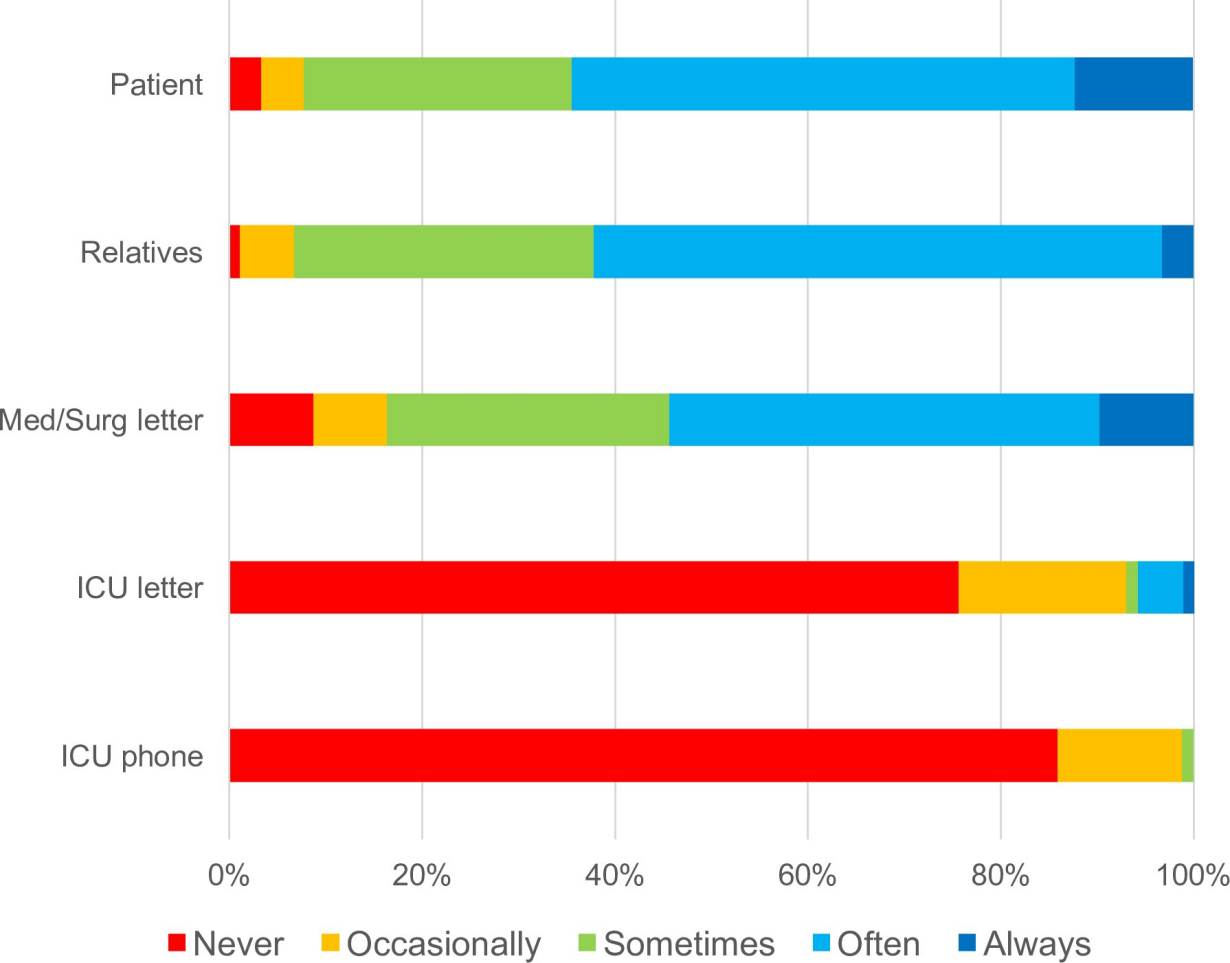
GP responses about receiving information flow. GP responses to question: If you receive information about your patient’s ICU stay, by which method(s) would you receive this information? [%].

ICMCs reported infrequent contact made with GPs (Fig 1). When contact did occur, it was usually by telephone (54/58; 93.1%), never by email, and usually for the purposes of seeking rather than sharing clinical information (Fig 2). For example, 37.9% of ICMCs often or always called a GP seeking information about a patient’s background medical history. In contrast, 8.8% of ICMCs often or always called the GP to share information about the patient’s recent ICU admission. GPs most commonly learned about an ICU admission from a patient (64.4% always or often) or a patient’s relative (62.2% always or often) after hospital discharge, and infrequently received information directly from ICU medical staff, either by phone or in writing (2.9% always or often) (Fig 3).

GPs commonly received information about an ICU admission within the general medical/surgical discharge summary (54.4% always or often). The information GPs received however frequently lacked details about the medical nature of the critical illness. Accordingly, technical information about organ support therapies (i.e. the need for dialysis or positive pressure ventilation) predominated over clinical information. A majority of GPs either never or occasionally received details about common critical illness complications such as acute respiratory distress syndrome (ARDS), shock, neuromuscular weakness and delirium.

#### Factors influencing current practices

A majority of ICMCs (47/62; 75.8%) and GPs (92/96; 95.8%) thought that information sharing about an ICU admission would benefit patients after hospital discharge. The most common barriers to communication reported by ICMCs were time constraints (21/63; 33.3%), its perception as a low-priority intervention (15/63; 23.8%) and a difficulty contacting the relevant GP (11/63; 17.5%). A minority (8/63; 12.7%) thought the ICU stay should be summarised in the hospital discharge summary.

A minority of GPs instigated a change in patient management when they learned about a recent ICU admission [25/95(26.3%) made direct contact with the patient; 28/94(29.8%) scheduled an early consultation].

### Qualitative data

All GP interviewees reported infrequent direct contact from the ICU; *“generally no”(GP5); “almost never”(GP4); “No*, *never”(GP3); “never that I can think of”(GP6); “…generally not*. *Once I think*.*”(GP2)*.

Qualitative findings were divided into *a priori* and emergent themes (Table 1).

**Table 1:** Themes and subthemes in qualitative data. Subthemes were either related to the three *a priori* themes (perceived benefits of information sharing; factors influencing current practice; strategies for optimal information sharing) or were discovered in the data (emergent themes).

Perceived benefits of information sharing	Factors influencing current practice	Strategies for optimal information sharing	Emergent themes
Identify high risk patients	Poor ICU information in ward team discharge summaries	Three critical illness milestones for information sharing	Centrality of GP in patient care
Guide provision of counselling and support	Laypersons are poor information couriers	Brief contact after unplanned ICU admission	The “whole patient journey”
Pre-empt patient or relative’s consultation	Lack of guidance about patient care in discharge summaries	Detailed summary after ICU discharge	Culture of expectation around GP’s knowledge of hospital care
		Notification after death in ICU	

#### Perceived benefits of information sharing

GPs and ICMCs recognised the value of ICU information to identify high-risk patients.

“*…puts us on alert to say right*, *this was a very ill person*.*”(GP3)*

“*I think it’s a flag that someone is in a very high risk group*.*”(ICMC4)*

Timely ICU information was also viewed as a tool to guide the provision of counselling or psychological support to patients after an acute illness or to enable future discussions about expectations of care in the event of illness recurrence.

*“*…*we are in a better position maybe to help them on the road…what needs they have and set realistic goals for them”(GP3)*

*“*…*generate that conversation about how the patient wants to proceed when they get unwell again”(ICMC3)*

GPs interviewees also valued early notification about ICU admission because it enabled them to be *“forewarned and forearmed”(GP4)*, to be *“already flagged”(GP3)*, thereby preparing them for consultations with relatives or patients seeking information about the ICU stay.

#### Factors influencing current practice

Three factors predominated in the interviews. First, the hospital ward team discharge summary was viewed as an unreliable source of ICU information. A non-ICU doctor would write “…*that the patient was admitted into ICU without writing about the course in the ICU”(ICMC3)*. These concerns were shared by GPs;

*“*…*probably the intern or the SHO does that brief discharge and may not have been involved in the care at all…in the ICU.”(GP4)*

Second, ICU patients or their relatives were viewed as unreliable information couriers;

*(The ICU)“…kind of fazes relatives*. *They’re very high tech.”(GP1)*

“Intensive care…they wouldn’t often remember a lot about it.”(GP3)

Third, when in receipt of ICU information, GP interviewees described non-specific patient interventions such as being “*a bit more proactive”(GP2)* or trying to “*tread a bit more carefully”(GP5)*. A common reason cited for not actioning more specific interventions was a lack of guidance in discharge summaries about what *“to anticipate in the future”(GP3)* or *“to look out for”(GP1)*.

#### Strategies for optimal information sharing

Both interviewee groups highlighted three milestones–unplanned ICU admission, ICU discharge and death in the ICU–which should trigger information sharing between the ICU and the patient’s GP. They also suggested ways of doing this effectively. Communication at the time of ICU admission should be brief, preferably by phone or email and could come from a non-medical ICU staff member. Early information sharing would also help ICU staff get useful clinical data to guide early patient management;

*“one of the first places where we can find reliable information is the GP*. *(ICMC5)*

Information provided at ICU discharge should be more detailed, as described by one GP, *“why they were in there[ICU]*, *what their consequences were*, *what we are to look out for”(GP1)*, ideally using a written or electronic form of communication. A common opinion from ICMC interviewees was that this should be the responsibility of the ICU team.

*“I think it is probably best that ICU relays that information*.*”(ICMC1)*

All interviewees agreed that GPs should be notified in person if their patient dies in the ICU, which one GP called *“the dying notice”(GP4)*.

#### Emergent themes

Three topics emerged during the interviews which define the context in which information sharing occurs. First, both groups recognised the central role of the GP in patient care;

*“…we’re there at the start and we’re there at the end*.*”(GP4)*

*“GPs are at the heart of the community*.*”(ICMC5)*

*“…your patients are our patients*.*”(GP5)*

Second, a consultant described the concept of the *“whole patient journey”(ICMC4)*, whereby the ICU admission was a short period in that journey but with potentially long term adverse effects.

Third, GPs reported that patients and relatives expected their family doctor to know about recent hospital events. Uninformed GPs felt ill-prepared if they were *“doorstepped”(ICMC1)* or *“caught in the headlights”(GP1)* by a patient or relative seeking hospital information. This can engender negative responses, a *“perception that you don’t care”(GP4)* or a situation where *“I mightn’t hear for weeks that somebody died and that’s disastrous”(GP3)*.

### Integrated data

Our mixed-methods approach revealed an apparent contradiction between the perceived patient benefits of information sharing between GPs and ICM consultants around an episode of critical illness and the suboptimal practice currently in place. The most common sources of ICU information for GPs (laypersons and hospital discharge summaries) were perceived to be the least reliable. It was clear from the interviews that, notwithstanding the barriers of limited time, resources and difficulty contacting a GP, the most reliable sources of information about intensive care developments were thought to be the healthcare staff working in the ICU.

Furthermore, while survey results suggested that GPs seldom actioned specific interventions for patients following a recent critical illness, two insights emerged from qualitative data to clarify this position. First, GPs did report a higher degree of vigilance and a readiness to refer for supportive care for these patients as required, but had concerns about poor availability of these services, especially physiotherapy and psychology support. Second, GPs felt poorly equipped to deal with post-ICU issues, citing a lack of experience and knowledge in this area. Of note, few GP survey respondents were familiar with the NICE guidelines on rehabilitation after critical illness [29].

## Discussion

Our study demonstrated infrequent clinical communication from ICU medical staff to GPs and, when present, was usually for the purpose of seeking rather than sharing clinical information. Accordingly, clinical details which would help GPs to evaluate a patient’s risk of critical illness complications, to guide patient management or to provide information to patients or relatives were seldom made available to them. In addition, concerns about a lack of community health resources and inexperience about critical illness complications were barriers to primary care management of post-ICU patients after hospital discharge.

Our results suggest a culture within the intensive care community of not sharing information with GPs about either an unplanned admission or, to a lesser extent, the death of their patient. While time constraints were a common mitigating factor, there was scant evidence that ICU departments explored other options for information sharing such as delegating communication with GPs to non-medical staff or exploring the use of electronic methods of communication. Of note, ICMCs were unaware of the presence of a national web-based clinical messaging system for healthcare professionals [30] which was highlighted by GP interviewees. A shared understanding of the nature of a patient’s medical illness as well as healthcare structure and process is important during patient transitions within the care continuum [31] and our data suggest that there are deficits in this so-called “common ground” [32] between intensive care and primary care practitioners.

Notwithstanding this, our study proposes methods for improving the quality of information sharing and promoting cultural change, in particular by protocolising the process of direct communication between ICUs and GPs at three critical illness milestones (unplanned ICU admission, ICU discharge and ICU death), by utilising the extant web-based messaging system, and by improving GPs’ knowledge about managing post-ICU problems. Moreover, strong sentiments about the patient benefits of information sharing suggest a culture amenable to change.

Our results raise the issue about which medical team should be responsible for sharing ICU information with GPs. Some consultants thought that, as critically ill patients are seldom discharged home, communication with GPs was a low priority for the ICU and was the responsibility of hospital ward doctors. Most interviewees however thought that hospital ward summaries were unreliable sources of ICU information. Whether this reflects poor information flow from the ICU to the ward or a poor understanding of critical illness by the ward doctors writing the discharges was not explored in our study. A subsequent audit in our ICU however has identified suboptimal sharing of key clinical information in ICU discharge summaries when patients were transferred to the hospital ward [O’Connor 2018, unpublished data]. In summary, a strong sentiment in our study was that sharing ICU information should be the responsibility of the medical staff directly involved in that care, and should either be by direct communication with the GP or indirectly by including an accurate ICU summary with the hospital ward discharge documents. [31,33]

**Fig 4:**
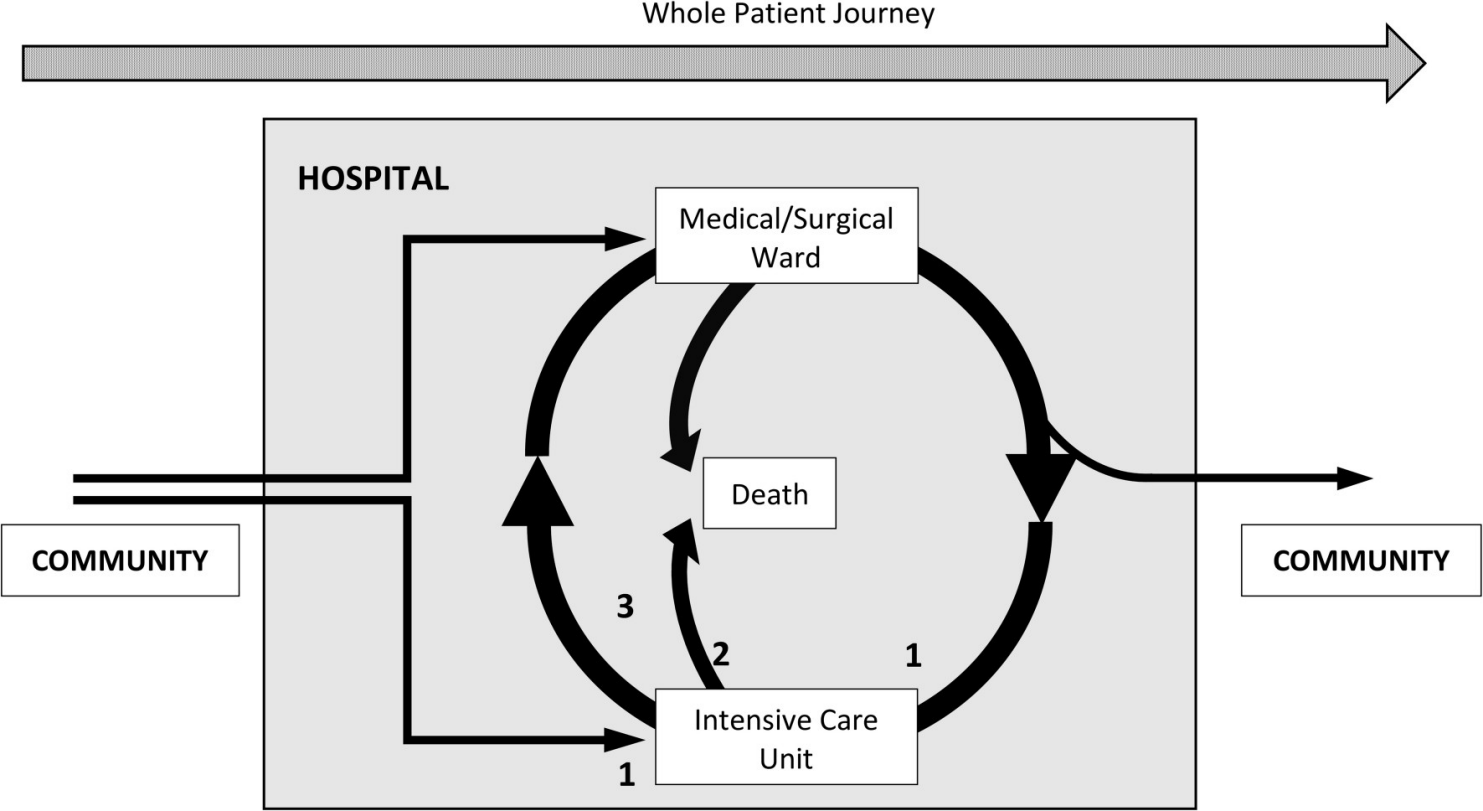
Critical illness milestones in the “whole patient journey”. After initial hospital assessment, patients may require admission to a hospital ward or directly to an ICU. In addition, hospital ward inpatients may subsequently have an unplanned ICU admission if their clinical condition deteriorates. The three critical illness milestones when information sharing between the ICU and the patient’s GP should occur are 1 (unplanned ICU admission), 2 (patient death in ICU) and 3 (ICU discharge to hospital ward).

Two overlapping constructs that emerged from the qualitative data were the healthcare “journey” that each patient takes, occurring mainly in the community, and the role of the GP as the central caregiver, who shares that journey with the patient and their relatives. The ICU stay is a transition within this journey, after which most patients ultimately return to the community. Our data suggest that this transition might be better navigated with the help of shared critical illness information around the key critical illness milestones (Fig 4).

For example, at the time of an unplanned ICU admission, patients may be too unwell to provide relevant clinical information. Seeking this information from the GP may enhance early critical illness management while also alerting the GP to a dramatic change in their patient’s condition. Similarly, as a consequence of a life-threatening illness, the patient’s journey may end in the ICU. Direct communication would enable the timely delivery of this important information to the GP. Indeed, depending on the model of ICU care in the hospital–closed versus open model [34]–a non-ICU medical team may have minimal input in the patient’s early critical illness management and may therefore be poorly placed to be the point of contact with the patient’s GP.

The third key critical illness milestone is when the patient is discharged from the ICU to the hospital ward. Though the total journey time for the patient in the ICU may be short, the physical and psychological effects thereof can be long lasting [5,25]. In addition, the medical and technical complexities of ICU care may be poorly understood by the patient, their relatives and their GP. Information sharing at this time may therefore improve the reliability of delivered information, facilitating a smooth reintegration of the patient into the community. Transitions of care and clinical handovers are commonly associated with loss of clinical information which may adversely affect patient outcomes [10,12,35]. Accordingly, reducing the communication steps by having direct contact between the sending unit (ICU) and the ultimate receiving unit (the GP) might optimise the fidelity and timeliness of information transfer.

In addition to facilitating patient-centred care, information sharing is a courtesy to and supports the professional role of medical colleagues in the community. Related to the GP’s role as key caregiver was our finding of a culture of expectation whereby relatives and patients assumed a level of GP awareness about in-hospital developments. This was viewed as fundamental to effective patient care after hospital discharge as well as being central to patients’ perceptions of GPs’ trustworthiness and professional competence.

Some of our findings overlap with existing literature. A recent multisource qualitative study identified infrequent information flow from ICUs to GPs around an ICU inpatient stay and a reliance on laypersons and junior non-ICU doctors for information transfer [18]. In a French survey study, GPs seldom received notification about an ICU admission, notwithstanding the large majority (93%) of patients whose relatives attended the GP seeking information during the admission [19]. The strongest predictors of GP dissatisfaction were the lack of either an ICU discharge letter or a notification about an unplanned ICU admission. In a further study, notifying GPs within 24hours of their patient’s admission to ICU helped GPs prepare for the discharge process back to the community [17]. Two recent qualitative studies explored patients’ and relatives’ perspectives about hospital discharge after an ICU admission. Both emphasised the benefits of tailored patient-centred information flow to help navigate this process [6,36].

Our study builds on this existing literature. The consultant perspective in our results yielded new insights about cultural influences on practice, previously explored only in the setting of ICU discharge to the ward [31]. Importantly, we also explored the impact of critical illness information on the quality of patient care delivered by GPs after hospital discharge, and how this process could be improved. Finally, our findings provide a blueprint, based on the three critical illness milestones, to operationally improve current practice and enhance the quality of information sharing around an episode of critical illness in an ICU.

Widening our sampling frames to include non-medical healthcare staff may have provided a more complete picture of the discharge process from ICU to hospital ward to the community. In this study however, we did not intend focusing primarily on discharge care. Multidisciplinary discharge planning, involving nursing and allied health interventions, has already been well described [37]. So also has a recent phenomenon of discharging selected ICU patients directly to the community, albeit those with a short hospital and ICU stay and a lesser need for community supports after discharge [38]. Instead, our research explored the flow and use of information between ICU doctors and GPs around the time of an unplanned ICU admission. Consequently, though our findings may help inform effective communication practices at the time of hospital discharge, they contain equally important messages about effective information sharing at other stages of the care continuum, namely at ICU admission and after a patient death in ICU.

Our study has several limitations. A small percentage of the total GPs in Ireland was included. Nevertheless, GPs from over three quarters of national county regions, with a broad urban and rural representation, participated. Furthermore, a high ICM consultant response rate supported the GP findings. Data consistency was seen across different sources and collection methods, reflecting the benefits of data triangulation [39]. That our survey results reflected findings in existing international literature supports the internal validity, credibility and generalisability of our data. Voluntary participation generated a small qualitative sample size which was not determined by data saturation, therefore there is a possibility that data relevant to the research area remained undiscovered. Nevertheless, a lack of new findings in the final interviews suggests that data saturation may have been achieved. We view our samples as having sufficient “information power” to add “new insights that contribute substantially to …current understandings” [40].

The use of predetermined codes for analysing qualitative data increased the risk of researcher bias. This was countered by a transparent research design, participant member-checking and independent researcher coding [39,41]. Finally, the use of mixed methods design inherently adds credibility to our findings [21], enabling “a better understanding of research problems than either approach [qualitative or quantitative] alone” [42].

## Conclusion

In summary, our study identified practical and cultural barriers to effective information sharing between ICU medical staff and GPs when a patient has an episode of critical illness. We also noted an underutilisation of critical illness information when received by GPs. Three critical illness milestones form the basis of a framework to improve information sharing during this important transition in a patient’s healthcare journey. Further research will focus on implementing and evaluating this framework.

## Supporting information

S1 Appendix Survey questionnaires to GPs and ICM consultants. Question structure for surveys distributed to GPs and ICM consultants.
(DOCX)

S2 Appendix Interview questions for GPs and ICM consultants. Question structure for interviews of GPs and consultants.
(DOCX)

S1 Dataset Survey results for ICM consultants. (PDF)

S2 Dataset Survey results for GPs. (PDF)

S1 File Anonymised qualitative data after first round of coding. (DOCX)
