# Supplementary material for: Information sharing between intensive care and primary care after an episode of critical illness; A mixed methods analysis
Source: PLoS One. 2019 Feb 28;14(2):e0212438. doi: 10.1371/journal.pone.0212438 (PMC6394993; doi:10.1371/journal.pone.0212438)
Supplement: S1 Appendix — Question structure for surveys distributed to GPs and ICM consultants. (DOCX) [file pone.0212438.s001.docx]

**ICU Consultant Questionnaire**

**1) Which of the following best describes your current professional position?**

Consultant with >50% clinical duties in the ICU []

Consultant with <50% clinical duties in the ICU []

Other (please explain below) []

|  |
| --- |

**2) In which type of hospital do you conduct most of your intensive care work?**

University-affiliated hospital []

Non-university hospital []

**3) When managing patients in your ICU, how often would you (or a member of your medical team) make direct contact, *for any purpose*, with the patient’s GP? (Circle answer)**

Never Occasionally Sometimes Often Always

**4) If you (or a member of your medical team) make contact with a patient’s GP, what is the purpose of this communication?**

|  | Never | Occasionally | Sometimes | Often | Always |
| --- | --- | --- | --- | --- | --- |
| To find out details leading up to patient’s current illness |  |  |  |  |  |
| To find out about the patient’s background medical or social history |  |  |  |  |  |
| To find out about patient’s regular medications and/or allergies |  |  |  |  |  |
| To inform the GP that their patient has been admitted to the ICU |  |  |  |  |  |
| To inform the GP about details of the patient’s ICU stay (eg. diagnosis, length of stay, illness severity) |  |  |  |  |  |
| Other purpose (please explain) | | | | | |

**5) When managing patients in your ICU, how often would you (or a member of your team) contact the GP *either during or after ICU admission* to give them details about their patient’s ICU stay (eg. diagnosis, length of stay, illness severity)? (Circle answer)**

Never Occasionally Sometimes Often Always

**6) When managing end-of-life care in your ICU, how often would you (or a member of your team) contact the GP to tell them their patient had died? (Circle answer)**

Never Occasionally Sometimes Often Always

**7) When/if you make contact with a patient’s GP, which method(s) of communication do you use (more than one if applicable)?**

Phone call []

Email []

Letter (either proforma or free-text) []

I do not make contact with GP []

**8) What do you think are the factors discouraging you from contacting a GP when their patient is in your ICU?**

|  |
| --- |

**9) Please comment on the following statement: “Effective communication between ICU doctors and GPs is beneficial for ICU patients after their hospital discharge?”**

Strongly agree Agree Neutral Disagree Strongly disagree

| (Please explain your answer) |
| --- |

**10) Please comment on the following statement: “Effective communication between ICU doctors and GPs is beneficial for *the relatives* of ICU inpatients?”**

Strongly agree Agree Neutral Disagree Strongly disagree

| (Please explain your answer) |
| --- |

**11) Do you participate in any outpatient clinic following up patients who have had a previous ICU stay with critical illness?**

Yes No

**12) A last request:** the second phase of this study involves focus group discussions with ICU consultants and GPs, exploring, in more detail, the themes within this questionnaire. Each participant would be asked to attend 1 group discussion, at a convenient time and location, lasting approximately 60minutes, chaired by a study researcher. Transport costs will be reimbursed. Snacks will be provided. It will be an accredited CPD activity.

If you are willing to take part (or want more information before deciding), please send a brief email to our departmental secretary at [XXXXXXXXXXXXXXX](mailto:mroberts@stjames.ie) and your details will be forwarded to the researchers. By responding to this request, you are in no way obligated to take part in the study. THANK YOU

**General Practitioner Questionnaire**

**1) Which of the following best describes your GP practice?**

Urban practice (Dublin, Cork, Galway or Limerick city) [] Rural practice (outside city) []

**2) Please indicate how long you have been working as a general practitioner**

<10years [] 10 years to 20 years [] >20years []

**3) In which county do you conduct most of your GP work?** ________________________

**4) Please comment on the following statements regarding communication you receive after your patients are discharged from hospital.**

|  | Never | Rarely | Occasionally | Often | Always |
| --- | --- | --- | --- | --- | --- |
| I receive details of the patient’s admission |  |  |  |  |  |
| I receive details of their admission within 30days of patient’s discharge |  |  |  |  |  |
| The details I receive include whether the patient was admitted to the ICU or not |  |  |  |  |  |

**5) If you received information *about your patient’s ICU stay* during their recent admission to hospital, by which method(s) would you receive this information?**

(If you never receive information about admission to ICU, please tick this box [])

|  | Never | Rarely | Occasionally | Often | Always |
| --- | --- | --- | --- | --- | --- |
| The ICU/anaesthetic staff phone me directly |  |  |  |  |  |
| In a summary or letter from *ICU medical team* |  |  |  |  |  |
| In a discharge summary or letter from other *non-ICU medical/surgical team* |  |  |  |  |  |
| I contact the hospital myself to find out |  |  |  |  |  |
| The patient’s relatives tell me |  |  |  |  |  |
| The patient tells me after hospital discharge |  |  |  |  |  |

**6) If you received information *about your patient’s stay in ICU* during their recent admission to hospital, how often would it include details about the following aspects of their critical illness?**

(If you never receive information about admission to ICU, please tick this box [])

|  | Never | Rarely | Occasionally | Often | Always |
| --- | --- | --- | --- | --- | --- |
| Shock |  |  |  |  |  |
| Respiratory failure and mechanical ventilation |  |  |  |  |  |
| Acute kidney injury requiring acute dialysis |  |  |  |  |  |
| Acute encephalopathy / Delirium |  |  |  |  |  |
| ARDS (acute respiratory distress syndrome) |  |  |  |  |  |
| Neuromuscular weakness |  |  |  |  |  |
| Tracheostomy insertion |  |  |  |  |  |
| The duration of patient’s stay in ICU |  |  |  |  |  |

**7) If, during a recent hospital stay, your patient was in ICU, would you record this ICU admission in the medical/surgical history section of their notes?** **(Circle yes or no)**

Yes No

**8) You receive a discharge summary in the post about your patient who was recently discharged from hospital after a severe illness. The summary confirms that they were in ICU during the hospital stay.**

**Please comment on the following statements about the patient’s follow-up care:**

|  | Strongly agree | Agree | Neutral | Disagree | Strongly disagree |
| --- | --- | --- | --- | --- | --- |
| The fact that the patient has had an ICU admission *would not change* how I manage their follow up care |  |  |  |  |  |
| If the patient did not self-present to my surgery for follow-up, *the recent ICU admission* would prompt me to schedule a consultation with them |  |  |  |  |  |
| If the patient did not self-present to my surgery for follow-up, *the recent ICU admission* would prompt me to schedule a consultation with the patient *and a close relative* |  |  |  |  |  |

**9) Have you ever attended an educational meeting at which you learned about the long term complications of critical illness? (Circle yes or no)**

YES NO

| (If Yes, what was the event? If No, would you attend one if it was available?) |
| --- |

**10) Are you aware of any published Guidelines about the rehabilitation of patients following hospital discharge after ICU admission with critical illness? (Circle yes or no)**

YES NO

| (If Yes, what is the Guideline?) |
| --- |

**11) Do you think it would benefit your patient *and/or their family members* if you received details about their ICU admission? (Circle yes or no)**

YES NO

| (If Yes, what the possible benefits?) |
| --- |

**12) A last request:** the second phase of this study involves focus group discussions with ICU consultants and GPs, exploring, in more detail, the themes within this questionnaire. Each participant would be asked to attend 1 group discussion, at a convenient time and location, lasting approximately 60minutes, chaired by a study researcher. Transport costs will be reimbursed. Snacks will be provided. It will be an accredited CPD activity.

If you are willing to take part (or want more information before deciding), please send a brief email to our departmental secretary at [XXXXXXXXXXXXX](mailto:mroberts@stjames.ie) and your details will be forwarded to the researchers. By responding to this request, you are in no way obligated to take part in the study.

THANK YOU
