# Supplementary material for: Information sharing between intensive care and primary care after an episode of critical illness; A mixed methods analysis
Source: PLoS One. 2019 Feb 28;14(2):e0212438. doi: 10.1371/journal.pone.0212438 (PMC6394993; doi:10.1371/journal.pone.0212438)
Supplement: S2 Appendix — Question structure for interviews of GPs and consultants. (DOCX) [file pone.0212438.s002.docx]

**Interview questions for general practitioners**

Regarding patients of yours who have been admitted to the ICU during a recent hospitalisation, would you receive much information about the ICU aspect of their care in hospital?

If you did receive information about the ICU stay, how would you receive this information?

Do you think your management of these patients after hospital discharge would change if you did receive information about their ICU admission?

Do you think that if you had more detailed information about an ICU admission, it would be likely to benefit patients in any way?

Have you any suggestions about how information sharing between the ICU and GPs could be improved?

Do you think there is a role of educating GPs about the complications of critical illness and unplanned ICU admission?

Have you any other comments or thoughts that you think are important about receiving information from the ICU when your patients are admitted there?

**Interview questions for ICU consultants**

Can you tell me some of your overall thoughts about the value of communication and information sharing between the ICU and a GP when their patient is in the ICU?

There is an opinion that the responsibility for the final discharge, containing all aspects of the hospitalisation including the ICU stay, should fall to the hospital ward team. What are your thoughts on that?

Do you think that it would benefit patients if their GP had a better insight into what happened to them while in the ICU?

Can you suggest ways that the sharing of ICU information with GPs could be improved while their patient is in the ICU?

Do you have any other thoughts about anything related or relevant to this topic? Is there anything else you would like to add?
