# Supplementary material for: Information sharing between intensive care and primary care after an episode of critical illness; A mixed methods analysis
Source: PLoS One. 2019 Feb 28;14(2):e0212438. doi: 10.1371/journal.pone.0212438 (PMC6394993; doi:10.1371/journal.pone.0212438)
Supplement: S1 Dataset — (PDF) [file pone.0212438.s003.pdf]

#1

COMPLETE

**Collector:**	Web Link 2 (Web Link)
**Started:**	Tuesday, June 07, 2016 9:14:48 AM
**Last Modified:**	Tuesday, June 07, 2016 9:17:37 AM
**Time Spent:**	00:02:49
**IP Address:**	213.233.132.148

Page 1

Q1 Which of the following best describes your current professional position?

Consultant with <50% clinical duties in ICU

Page 2

Q2 In which type of hospital do you conduct most of your intensive care work?

University-affiliated hospital

Page 3

Q3 When managing patients in your ICU, how often would you (or a member of your ICU team) make direct contact, for any purpose, with the patient's GP?

Often

Page 4

Q4 If you (or a member of your ICU team) make contact with a patient's GP, what is the purpose of this communication?

To find out details leading to the patient's current illness	**Often**
To find out about patient's background medical and/or social history	**Often**
To find out about patient's regular medications and/or allergies	**Sometimes**
To inform the GP that their patient has been admitted to ICU	**Occasionally**
To inform the GP about details of the patient's ICU stay (eg. diagnosis, length of stay and/or illness severity)	**Never**

Page 5

Q5 When managing patients in your ICU, how often would you (or a member of your ICU team) contact the GP either during or after ICU admission to give them details about their patient's ICU stay (eg. diagnosis, length of stay, illness severity)?

Sometimes

Page 6

Q6 When managing end-of-life care in your ICU, how often would you (or a member of your ICU team) contact the GP to tell them that their patient had died?

Always

Page 7

Q7 When/if you make contact with a patient's GP, which method(s) of communication do you use (more than one if applicable)?

Phone call

Page 8

Q8 What do you think are the main factors discouraging you from contacting a GP when their patient is in your ICU?

Out of hours communication difficult

Page 9

Q9 Please comment on the following statement: "Effective communication between ICU doctors and GPs is likely to benefit ICU patients after their hospital discharge"

Agree

Page 10

Q10 Please comment on the following statement: "Effective communication between ICU doctors and GPs is likely to benefit the relatives of ICU patients after their hospital discharge"

Neutral

Page 11

Q11 Do you participate in any outpatient clinic following up patients who have had a previous ICU stay with critical illness

No

#2

COMPLETE

Collector: Web Link 2 (Web Link)
Started: Tuesday, June 07, 2016 9:23:34 AM
Last Modified: Tuesday, June 07, 2016 9:27:33 AM
Time Spent: 00:03:59
IP Address: 207.219.153.170

Page 1

Q1 Which of the following best describes your current professional position?

Consultant with >/=50% clinical duties in ICU

Page 2

Q2 In which type of hospital do you conduct most of your intensive care work?

University-affiliated hospital

Page 3

Q3 When managing patients in your ICU, how often would you (or a member of your ICU team) make direct contact, for any purpose, with the patient's GP?

Occasionally

Page 4

Q4 If you (or a member of your ICU team) make contact with a patient's GP, what is the purpose of this communication?

To find out details leading to the patient's current illness	**Occasionally**
To find out about patient's background medical and/or social history	**Often**
To find out about patient's regular medications and/or allergies	**Often**
To inform the GP that their patient has been admitted to ICU	**Never**
To inform the GP about details of the patient's ICU stay (eg. diagnosis, length of stay and/or illness severity)	**Occasionally**

Page 5

Q5 When managing patients in your ICU, how often would you (or a member of your ICU team) contact the GP either during or after ICU admission to give them details about their patient's ICU stay (eg. diagnosis, length of stay, illness severity)?

Occasionally

Page 6

Q6 When managing end-of-life care in your ICU, how often would you (or a member of your ICU team) contact the GP to tell them that their patient had died?

Never

Page 7

Q7 When/if you make contact with a patient's GP, which method(s) of communication do you use (more than one if applicable)?

Phone call

Page 8

Q8 What do you think are the main factors discouraging you from contacting a GP when their patient is in your ICU?

Lack of insight from general practice in to ICU practice

Page 9

Q9 Please comment on the following statement: "Effective communication between ICU doctors and GPs is likely to benefit ICU patients after their hospital discharge"

Agree

Page 10

Q10 Please comment on the following statement: "Effective communication between ICU doctors and GPs is likely to benefit the relatives of ICU patients after their hospital discharge"

Neutral

Page 11

Q11 Do you participate in any outpatient clinic following up patients who have had a previous ICU stay with critical illness

No

#3

INCOMPLETE

Collector: Web Link 2 (Web Link)
Started: Tuesday, June 07, 2016 9:48:31 AM
Last Modified: Tuesday, June 07, 2016 9:48:45 AM
Time Spent: 00:00:14
IP Address: 95.45.28.135

Page 1

Q1 Which of the following best describes your current professional position?

Consultant with <50% clinical duties in ICU

Page 2

Q2 In which type of hospital do you conduct most of your intensive care work?

University-affiliated hospital

Page 3

Q3 When managing patients in your ICU, how often would you (or a member of your ICU team) make direct contact, for any purpose, with the patient's GP?

Respondent skipped this question

Page 4

Q4 If you (or a member of your ICU team) make contact with a patient's GP, what is the purpose of this communication?

Respondent skipped this question

Page 5

Q5 When managing patients in your ICU, how often would you (or a member of your ICU team) contact the GP either during or after ICU admission to give them details about their patient's ICU stay (eg. diagnosis, length of stay, illness severity)?

Respondent skipped this question

Page 6

Q6 When managing end-of-life care in your ICU, how often would you (or a member of your ICU team) contact the GP to tell them that their patient had died?

Respondent skipped this question

Page 7

Q7 When/if you make contact with a patient's GP, which method(s) of communication do you use (more than one if applicable)?

Respondent skipped this question

Page 8

Q8 What do you think are the main factors discouraging you from contacting a GP when their patient is in your ICU?

Respondent skipped this question

Page 9

Q9 Please comment on the following statement: "Effective communication between ICU doctors and GPs is likely to benefit ICU patients after their hospital discharge"

Respondent skipped this question

Page 10

Q10 Please comment on the following statement: "Effective communication between ICU doctors and GPs is likely to benefit the relatives of ICU patients after their hospital discharge"

Respondent skipped this question

Page 11

Q11 Do you participate in any outpatient clinic following up patients who have had a previous ICU stay with critical illness

Respondent skipped this question

#4

COMPLETE

Collector: Web Link 2 (Web Link)
Started: Tuesday, June 07, 2016 9:59:30 AM
Last Modified: Tuesday, June 07, 2016 10:03:20 AM
Time Spent: 00:03:50
IP Address: 86.131.71.223

Page 1

Q1 Which of the following best describes your current professional position?

Consultant with >/=50% clinical duties in ICU

Page 2

Q2 In which type of hospital do you conduct most of your intensive care work?

University-affiliated hospital

Page 3

Q3 When managing patients in your ICU, how often would you (or a member of your ICU team) make direct contact, for any purpose, with the patient's GP?

Often

Page 4

Q4 If you (or a member of your ICU team) make contact with a patient's GP, what is the purpose of this communication?

To find out details leading to the patient's current illness	**Sometimes**
To find out about patient's background medical and/or social history	**Sometimes**
To find out about patient's regular medications and/or allergies	**Occasionally**
To inform the GP that their patient has been admitted to ICU	**Never**
To inform the GP about details of the patient's ICU stay (eg. diagnosis, length of stay and/or illness severity)	**Always**

Page 5

Q5 When managing patients in your ICU, how often would you (or a member of your ICU team) contact the GP either during or after ICU admission to give them details about their patient's ICU stay (eg. diagnosis, length of stay, illness severity)?

Often

Page 6

Q6 When managing end-of-life care in your ICU, how often would you (or a member of your ICU team) contact the GP to tell them that their patient had died?

Always

Page 7

Q7 When/if you make contact with a patient's GP, which method(s) of communication do you use (more than one if applicable)?

Phone call,

Written letter

Page 8

Q8 What do you think are the main factors discouraging you from contacting a GP when their patient is in your ICU?

lack of clarity regarding their interest/information needs

Page 9

Q9 Please comment on the following statement: "Effective communication between ICU doctors and GPs is likely to benefit ICU patients after their hospital discharge"

Agree,
Please explain your answer:
Clarifying expected issues with rehabilitation, complications etc

Page 10

Q10 Please comment on the following statement: "Effective communication between ICU doctors and GPs is likely to benefit the relatives of ICU patients after their hospital discharge"

Agree,
Please explain your answer:
relatives may have questions regarding ICU stay or complications and the more information GP has the better

Page 11

Q11 Do you participate in any outpatient clinic following up patients who have had a previous ICU stay with critical illness

No

#5

COMPLETE

Collector: Web Link 2 (Web Link)
Started: Tuesday, June 07, 2016 11:06:18 AM
Last Modified: Tuesday, June 07, 2016 11:09:28 AM
Time Spent: 00:03:10
IP Address: 213.233.150.8

Page 1

Q1 Which of the following best describes your current professional position?

Consultant with <50% clinical duties in ICU

Page 2

Q2 In which type of hospital do you conduct most of your intensive care work?

University-affiliated hospital

Page 3

Q3 When managing patients in your ICU, how often would you (or a member of your ICU team) make direct contact, for any purpose, with the patient's GP?

Sometimes

Page 4

Q4 If you (or a member of your ICU team) make contact with a patient's GP, what is the purpose of this communication?

To find out details leading to the patient's current illness	**Sometimes**
To find out about patient's background medical and/or social history	**Sometimes**
To find out about patient's regular medications and/or allergies	**Sometimes**
To inform the GP that their patient has been admitted to ICU	**Never**
To inform the GP about details of the patient's ICU stay (eg. diagnosis, length of stay and/or illness severity)	**Occasionally**

Page 5

Q5 When managing patients in your ICU, how often would you (or a member of your ICU team) contact the GP either during or after ICU admission to give them details about their patient's ICU stay (eg. diagnosis, length of stay, illness severity)?

Occasionally

Page 6

Q6 When managing end-of-life care in your ICU, how often would you (or a member of your ICU team) contact the GP to tell them that their patient had died?

Never

Page 7

Q7 When/if you make contact with a patient's GP, which method(s) of communication do you use (more than one if applicable)?

Phone call,

Written letter

Page 8

Q8 What do you think are the main factors discouraging you from contacting a GP when their patient is in your ICU?

None

Page 9

Q9 Please comment on the following statement: "Effective communication between ICU doctors and GPs is likely to benefit ICU patients after their hospital discharge"

Neutral

Page 10

Q10 Please comment on the following statement: "Effective communication between ICU doctors and GPs is likely to benefit the relatives of ICU patients after their hospital discharge"

Disagree

Page 11

Q11 Do you participate in any outpatient clinic following up patients who have had a previous ICU stay with critical illness

No

#6

COMPLETE

Collector: Web Link 2 (Web Link)
Started: Tuesday, June 07, 2016 12:15:13 PM
Last Modified: Tuesday, June 07, 2016 12:18:58 PM
Time Spent: 00:03:45
IP Address: 213.233.150.21

Page 1

Q1 Which of the following best describes your current professional position?

Consultant with <50% clinical duties in ICU

Page 2

Q2 In which type of hospital do you conduct most of your intensive care work?

University-affiliated hospital

Page 3

Q3 When managing patients in your ICU, how often would you (or a member of your ICU team) make direct contact, for any purpose, with the patient's GP?

Often

Page 4

Q4 If you (or a member of your ICU team) make contact with a patient's GP, what is the purpose of this communication?

To find out details leading to the patient's current illness	**Sometimes**
To find out about patient's background medical and/or social history	**Often**
To find out about patient's regular medications and/or allergies	**Often**
To inform the GP that their patient has been admitted to ICU	**Occasionally**
To inform the GP about details of the patient's ICU stay (eg. diagnosis, length of stay and/or illness severity)	**Sometimes**

Page 5

Q5 When managing patients in your ICU, how often would you (or a member of your ICU team) contact the GP either during or after ICU admission to give them details about their patient's ICU stay (eg. diagnosis, length of stay, illness severity)?

Often

Page 6

Q6 When managing end-of-life care in your ICU, how often would you (or a member of your ICU team) contact the GP to tell them that their patient had died?

Often

Page 7

Q7 When/if you make contact with a patient's GP, which method(s) of communication do you use (more than one if applicable)?

Phone call

Page 8

Q8 What do you think are the main factors discouraging you from contacting a GP when their patient is in your ICU?

None

Page 9

Q9 Please comment on the following statement: "Effective communication between ICU doctors and GPs is likely to benefit ICU patients after their hospital discharge"

Agree,
Please explain your answer:
Has to be good

Page 10

Q10 Please comment on the following statement: "Effective communication between ICU doctors and GPs is likely to benefit the relatives of ICU patients after their hospital discharge"

Agree,
Please explain your answer:
Has to be good

Page 11

Q11 Do you participate in any outpatient clinic following up patients who have had a previous ICU stay with critical illness

No

#7

COMPLETE

Collector: Web Link 2 (Web Link)
Started: Tuesday, June 07, 2016 1:37:20 PM
Last Modified: Tuesday, June 07, 2016 1:42:06 PM
Time Spent: 00:04:46
IP Address: 185.32.93.254

Page 1

Q1 Which of the following best describes your current professional position?

Consultant with >/=50% clinical duties in ICU

Page 2

Q2 In which type of hospital do you conduct most of your intensive care work?

University-affiliated hospital

Page 3

Q3 When managing patients in your ICU, how often would you (or a member of your ICU team) make direct contact, for any purpose, with the patient's GP?

Occasionally

Page 4

Q4 If you (or a member of your ICU team) make contact with a patient's GP, what is the purpose of this communication?

To find out details leading to the patient's current illness	**Never**
To find out about patient's background medical and/or social history	**Never**
To find out about patient's regular medications and/or allergies	**Never**
To inform the GP that their patient has been admitted to ICU	**Occasionally**
To inform the GP about details of the patient's ICU stay (eg. diagnosis, length of stay and/or illness severity)	**Occasionally**
Other (please specify)	**To inform them of the patient's death**

Page 5

Q5 When managing patients in your ICU, how often would you (or a member of your ICU team) contact the GP either during or after ICU admission to give them details about their patient's ICU stay (eg. diagnosis, length of stay, illness severity)?

Occasionally

Page 6

Q6 When managing end-of-life care in your ICU, how often would you (or a member of your ICU team) contact the GP to tell them that their patient had died?

Always

Page 7

Q7 When/if you make contact with a patient's GP, which method(s) of communication do you use (more than one if applicable)?

Phone call

Page 8

Q8 What do you think are the main factors discouraging you from contacting a GP when their patient is in your ICU?

Usually done by primary admitting physician/ surgeon- however likely does not include illness severity eyc

Page 9

Q9 Please comment on the following statement: "Effective communication between ICU doctors and GPs is likely to benefit ICU patients after their hospital discharge"

Agree

Page 10

Q10 Please comment on the following statement: "Effective communication between ICU doctors and GPs is likely to benefit the relatives of ICU patients after their hospital discharge"

Agree

Page 11

Q11 Do you participate in any outpatient clinic following up patients who have had a previous ICU stay with critical illness

No

#8

COMPLETE

Collector: Web Link 2 (Web Link)
Started: Tuesday, June 07, 2016 3:20:35 PM
Last Modified: Tuesday, June 07, 2016 3:23:09 PM
Time Spent: 00:02:34
IP Address: 178.167.254.189

Page 1

Q1 Which of the following best describes your current professional position?

Consultant with <50% clinical duties in ICU

Page 2

Q2 In which type of hospital do you conduct most of your intensive care work?

University-affiliated hospital

Page 3

Q3 When managing patients in your ICU, how often would you (or a member of your ICU team) make direct contact, for any purpose, with the patient's GP?

Occasionally

Page 4

Q4 If you (or a member of your ICU team) make contact with a patient's GP, what is the purpose of this communication?

To find out details leading to the patient's current illness	**Sometimes**
To find out about patient's background medical and/or social history	**Sometimes**
To find out about patient's regular medications and/or allergies	**Sometimes**
To inform the GP that their patient has been admitted to ICU	**Never**
To inform the GP about details of the patient's ICU stay (eg. diagnosis, length of stay and/or illness severity)	**Never**

Page 5

Q5 When managing patients in your ICU, how often would you (or a member of your ICU team) contact the GP either during or after ICU admission to give them details about their patient's ICU stay (eg. diagnosis, length of stay, illness severity)?

Occasionally

Page 6

Q6 When managing end-of-life care in your ICU, how often would you (or a member of your ICU team) contact the GP to tell them that their patient had died?

Occasionally

Page 7

Q7 When/if you make contact with a patient's GP, which method(s) of communication do you use (more than one if applicable)?

Phone call

Page 8

Q8 What do you think are the main factors discouraging you from contacting a GP when their patient is in your ICU?

ICU team's relationship is largely with the referring consultant / team in the hospital

Page 9

Q9 Please comment on the following statement: "Effective communication between ICU doctors and GPs is likely to benefit ICU patients after their hospital discharge"

Neutral

Page 10

Q10 Please comment on the following statement: "Effective communication between ICU doctors and GPs is likely to benefit the relatives of ICU patients after their hospital discharge"

Neutral

Page 11

Q11 Do you participate in any outpatient clinic following up patients who have had a previous ICU stay with critical illness

No

#9

COMPLETE

Collector: Web Link 2 (Web Link)
Started: Tuesday, June 07, 2016 4:11:52 PM
Last Modified: Tuesday, June 07, 2016 4:19:32 PM
Time Spent: 00:07:40
IP Address: 176.61.8.197

Page 1

Q1 Which of the following best describes your current professional position?

Consultant with >/=50% clinical duties in ICU

Page 2

Q2 In which type of hospital do you conduct most of your intensive care work?

University-affiliated hospital

Page 3

Q3 When managing patients in your ICU, how often would you (or a member of your ICU team) make direct contact, for any purpose, with the patient's GP?

Occasionally

Page 4

Q4 If you (or a member of your ICU team) make contact with a patient's GP, what is the purpose of this communication?

To find out details leading to the patient's current illness	**Often**
To find out about patient's background medical and/or social history	**Always**
To find out about patient's regular medications and/or allergies	**Often**
To inform the GP that their patient has been admitted to ICU	**Occasionally**
To inform the GP about details of the patient's ICU stay (eg. diagnosis, length of stay and/or illness severity)	**Occasionally**

Page 5

Q5 When managing patients in your ICU, how often would you (or a member of your ICU team) contact the GP either during or after ICU admission to give them details about their patient's ICU stay (eg. diagnosis, length of stay, illness severity)?

Never

Page 6

Q6 When managing end-of-life care in your ICU, how often would you (or a member of your ICU team) contact the GP to tell them that their patient had died?

Never

Page 7

Q7 When/if you make contact with a patient's GP, which method(s) of communication do you use (more than one if applicable)?

I do not make contact with the GP

Page 8

Q8 What do you think are the main factors discouraging you from contacting a GP when their patient is in your ICU?

I can usually glean the necessary medical history form patients / relatives and or chart review.

Page 9

Q9 Please comment on the following statement: "Effective communication between ICU doctors and GPs is likely to benefit ICU patients after their hospital discharge"

Neutral,
Please explain your answer:
The primary team that care for patients after ICU discharge are in a better position to communicate with GPs regarding patient prognosis and residual disability.

Page 10

Q10 Please comment on the following statement: "Effective communication between ICU doctors and GPs is likely to benefit the relatives of ICU patients after their hospital discharge"

Neutral,
Please explain your answer:
This is the responsibility of the primary team, who have more experience in this area than I do, and are better qualified to perform this task. The primary team also has the capacity to follow patients and their relatives in out patient visits after discharge, and so has more opportunity to pursue patient contact.

Page 11

Q11 Do you participate in any outpatient clinic following up patients who have had a previous ICU stay with critical illness

No

#10

COMPLETE

Collector: Web Link 2 (Web Link)
Started: Tuesday, June 07, 2016 6:33:32 PM
Last Modified: Tuesday, June 07, 2016 6:56:38 PM
Time Spent: 00:23:06
IP Address: 84.203.136.190

Page 1

Q1 Which of the following best describes your current professional position?

Consultant with >/=50% clinical duties in ICU

Page 2

Q2 In which type of hospital do you conduct most of your intensive care work?

University-affiliated hospital

Page 3

Q3 When managing patients in your ICU, how often would you (or a member of your ICU team) make direct contact, for any purpose, with the patient's GP?

Sometimes

Page 4

Q4 If you (or a member of your ICU team) make contact with a patient's GP, what is the purpose of this communication?

To find out details leading to the patient's current illness	**Sometimes**
To find out about patient's background medical and/or social history	**Sometimes**
To find out about patient's regular medications and/or allergies	**Sometimes**
To inform the GP that their patient has been admitted to ICU	**Never**
Other (please specify)	**We don't make direct contact with the GP normally but we do a routine Critical Care discharge summary which is primarily directied at the hospital team taking over clinical care but is available for transmission to GP when patient is being discharged from hospital.**

Page 5

Q5 When managing patients in your ICU, how often would you (or a member of your ICU team) contact the GP either during or after ICU admission to give them details about their patient's ICU stay (eg. diagnosis, length of stay, illness severity)?

Never

Page 6

Q6 When managing end-of-life care in your ICU, how often would you (or a member of your ICU team) contact the GP to tell them that their patient had died?

Never

Page 7

Q7 When/if you make contact with a patient's GP, which method(s) of communication do you use (more than one if applicable)?

Phone call,

Written letter

Page 8

Q8 What do you think are the main factors discouraging you from contacting a GP when their patient is in your ICU?

Mainly because the GP is seen as two links away (at a minimum) in the chain of communication. We tend to be very attentive to communication with the referring team and largely tend to rely on the referring / admitting team in turn to make appropriate contact with GP - esp at time of hospital discharge or at the time of dying or death. Indeed the comment above re 'written contact with GP' refers to an indirect communication with the GP - the letter is actually written to the referring clinician.

Page 9

Q9 Please comment on the following statement: "Effective communication between ICU doctors and GPs is likely to benefit ICU patients after their hospital discharge"

Agree,
Please explain your answer:
It would likely improve the GP's understanding of what the patient has been through (and perhaps the likelihood of a post traumatic stress-like disorder). Might also beter explain some of the medications that the patient goes home on e.g. beta blockers, ACE inhibitors, diuretics....

Page 10

Q10 Please comment on the following statement: "Effective communication between ICU doctors and GPs is likely to benefit the relatives of ICU patients after their hospital discharge"

Agree,
Please explain your answer:
Otherwise the relatives (with no disrespect to them) may be the primary means of the GP having an insight into the critical care course

Page 11

Q11 Do you participate in any outpatient clinic following up patients who have had a previous ICU stay with critical illness

No

#11

COMPLETE

Collector: Web Link 2 (Web Link)
Started: Tuesday, June 07, 2016 7:56:16 PM
Last Modified: Tuesday, June 07, 2016 8:27:03 PM
Time Spent: 00:30:47
IP Address: 62.64.183.194

Page 1

Q1 Which of the following best describes your current professional position?

Consultant with >/=50% clinical duties in ICU

Page 2

Q2 In which type of hospital do you conduct most of your intensive care work?

University-affiliated hospital

Page 3

Q3 When managing patients in your ICU, how often would you (or a member of your ICU team) make direct contact, for any purpose, with the patient's GP?

Often

Page 4

Q4 If you (or a member of your ICU team) make contact with a patient's GP, what is the purpose of this communication?

To find out details leading to the patient's current illness	**Sometimes**
To find out about patient's background medical and/or social history	**Sometimes**
To find out about patient's regular medications and/or allergies	**Occasionally**
To inform the GP that their patient has been admitted to ICU	**Occasionally**
To inform the GP about details of the patient's ICU stay (eg. diagnosis, length of stay and/or illness severity)	**Always**

Page 5

Q5 When managing patients in your ICU, how often would you (or a member of your ICU team) contact the GP either during or after ICU admission to give them details about their patient's ICU stay (eg. diagnosis, length of stay, illness severity)?

Always

Page 6

Q6 When managing end-of-life care in your ICU, how often would you (or a member of your ICU team) contact the GP to tell them that their patient had died?

Always

Page 7

Q7 When/if you make contact with a patient's GP, which method(s) of communication do you use (more than one if applicable)?

Phone call,

Written letter

Page 8

Q8 What do you think are the main factors discouraging you from contacting a GP when their patient is in your ICU?

Time

Page 9

Q9 Please comment on the following statement: "Effective communication between ICU doctors and GPs is likely to benefit ICU patients after their hospital discharge"

Strongly agree,
Please explain your answer:
Information informs patient's long term care, chronic disorders, post ICU syndrome. Helps GP deal with questions from patient or family. Informs future limitations of treatment or dnacpr decisions.

Page 10

Q10 Please comment on the following statement: "Effective communication between ICU doctors and GPs is likely to benefit the relatives of ICU patients after their hospital discharge"

Strongly agree,
Please explain your answer:
Having informed GP to be aware of issues, answer questions and be aware of stress, social issues and ongoing care needs all supports family in their care of patient. Or indeed after the death of a relative in ICU.

Page 11

Q11 Do you participate in any outpatient clinic following up patients who have had a previous ICU stay with critical illness

Yes

#12

COMPLETE

Collector: Web Link 2 (Web Link)
Started: Tuesday, June 07, 2016 8:38:36 PM
Last Modified: Tuesday, June 07, 2016 8:40:30 PM
Time Spent: 00:01:54
IP Address: 37.228.228.118

Page 1

Q1 Which of the following best describes your current professional position?

Consultant with <50% clinical duties in ICU

Page 2

Q2 In which type of hospital do you conduct most of your intensive care work?

University-affiliated hospital

Page 3

Q3 When managing patients in your ICU, how often would you (or a member of your ICU team) make direct contact, for any purpose, with the patient's GP?

Never

Page 4

Q4 If you (or a member of your ICU team) make contact with a patient's GP, what is the purpose of this communication?

Respondent skipped this question

Page 5

Q5 When managing patients in your ICU, how often would you (or a member of your ICU team) contact the GP either during or after ICU admission to give them details about their patient's ICU stay (eg. diagnosis, length of stay, illness severity)?

Respondent skipped this question

Page 6

Q6 When managing end-of-life care in your ICU, how often would you (or a member of your ICU team) contact the GP to tell them that their patient had died?

Respondent skipped this question

Page 7

Q7 When/if you make contact with a patient's GP, which method(s) of communication do you use (more than one if applicable)?

Respondent skipped this question

Page 8

Q8 What do you think are the main factors discouraging you from contacting a GP when their patient is in your ICU?

GP difficult to contact ,time pressures, GP may not be that familiar with patient

Page 9

Q9 Please comment on the following statement: "Effective communication between ICU doctors and GPs is likely to benefit ICU patients after their hospital discharge"

Strongly agree

Page 10

Q10 Please comment on the following statement: "Effective communication between ICU doctors and GPs is likely to benefit the relatives of ICU patients after their hospital discharge"

Strongly agree

Page 11

Q11 Do you participate in any outpatient clinic following up patients who have had a previous ICU stay with critical illness

No

#13

COMPLETE

Collector: Web Link 2 (Web Link)
Started: Wednesday, June 08, 2016 8:02:53 AM
Last Modified: Wednesday, June 08, 2016 8:05:50 AM
Time Spent: 00:02:57
IP Address: 87.100.19.225

Page 1

Q1 Which of the following best describes your current professional position?

Consultant with >/=50% clinical duties in ICU

Page 2

Q2 In which type of hospital do you conduct most of your intensive care work?

University-affiliated hospital

Page 3

Q3 When managing patients in your ICU, how often would you (or a member of your ICU team) make direct contact, for any purpose, with the patient's GP?

Never

Page 4

Q4 If you (or a member of your ICU team) make contact with a patient's GP, what is the purpose of this communication?

Respondent skipped this question

Page 5

Q5 When managing patients in your ICU, how often would you (or a member of your ICU team) contact the GP either during or after ICU admission to give them details about their patient's ICU stay (eg. diagnosis, length of stay, illness severity)?

Respondent skipped this question

Page 6

Q6 When managing end-of-life care in your ICU, how often would you (or a member of your ICU team) contact the GP to tell them that their patient had died?

Respondent skipped this question

Page 7

Q7 When/if you make contact with a patient's GP, which method(s) of communication do you use (more than one if applicable)?

Respondent skipped this question

Page 8

Q8 What do you think are the main factors discouraging you from contacting a GP when their patient is in your ICU?

Belief that the primary team responsible for communication with GP

Page 9

Q9 Please comment on the following statement: "Effective communication between ICU doctors and GPs is likely to benefit ICU patients after their hospital discharge"

Agree

Page 10

Q10 Please comment on the following statement: "Effective communication between ICU doctors and GPs is likely to benefit the relatives of ICU patients after their hospital discharge"

Agree

Page 11

Q11 Do you participate in any outpatient clinic following up patients who have had a previous ICU stay with critical illness

No

#14

COMPLETE

Collector: Web Link 2 (Web Link)
Started: Wednesday, June 08, 2016 9:59:29 AM
Last Modified: Wednesday, June 08, 2016 10:02:31 AM
Time Spent: 00:03:02
IP Address: 137.191.231.131

Page 1

Q1 Which of the following best describes your current professional position?

Consultant with >/=50% clinical duties in ICU

Page 2

Q2 In which type of hospital do you conduct most of your intensive care work?

University-affiliated hospital

Page 3

Q3 When managing patients in your ICU, how often would you (or a member of your ICU team) make direct contact, for any purpose, with the patient's GP?

Occasionally

Page 4

Q4 If you (or a member of your ICU team) make contact with a patient's GP, what is the purpose of this communication?

To find out details leading to the patient's current illness	**Never**
To find out about patient's background medical and/or social history	**Occasionally**
To find out about patient's regular medications and/or allergies	**Never**
To inform the GP that their patient has been admitted to ICU	**Never**
To inform the GP about details of the patient's ICU stay (eg. diagnosis, length of stay and/or illness severity)	**Occasionally**

Page 5

Q5 When managing patients in your ICU, how often would you (or a member of your ICU team) contact the GP either during or after ICU admission to give them details about their patient's ICU stay (eg. diagnosis, length of stay, illness severity)?

Occasionally

Page 6

Q6 When managing end-of-life care in your ICU, how often would you (or a member of your ICU team) contact the GP to tell them that their patient had died?

Always

Page 7

Q7 When/if you make contact with a patient's GP, which method(s) of communication do you use (more than one if applicable)?

Phone call,

Written letter

Page 8

Q8 What do you think are the main factors discouraging you from contacting a GP when their patient is in your ICU?

Competing interests taking up time

Page 9

Q9 Please comment on the following statement: "Effective communication between ICU doctors and GPs is likely to benefit ICU patients after their hospital discharge"

Agree

Page 10

Q10 Please comment on the following statement: "Effective communication between ICU doctors and GPs is likely to benefit the relatives of ICU patients after their hospital discharge"

Agree

Page 11

Q11 Do you participate in any outpatient clinic following up patients who have had a previous ICU stay with critical illness

Yes

#15

COMPLETE

Collector: Web Link 2 (Web Link)
Started: Wednesday, June 08, 2016 10:12:01 AM
Last Modified: Wednesday, June 08, 2016 10:16:32 AM
Time Spent: 00:04:31
IP Address: 82.132.229.216

Page 1

Q1 Which of the following best describes your current professional position?

Consultant with >/=50% clinical duties in ICU

Page 2

Q2 In which type of hospital do you conduct most of your intensive care work?

University-affiliated hospital

Page 3

Q3 When managing patients in your ICU, how often would you (or a member of your ICU team) make direct contact, for any purpose, with the patient's GP?

Sometimes

Page 4

Q4 If you (or a member of your ICU team) make contact with a patient's GP, what is the purpose of this communication?

To find out details leading to the patient's current illness	**Occasionally**
To find out about patient's background medical and/or social history	**Sometimes**
To find out about patient's regular medications and/or allergies	**Often**
To inform the GP that their patient has been admitted to ICU	**Occasionally**
To inform the GP about details of the patient's ICU stay (eg. diagnosis, length of stay and/or illness severity)	**Occasionally**

Page 5

Q5 When managing patients in your ICU, how often would you (or a member of your ICU team) contact the GP either during or after ICU admission to give them details about their patient's ICU stay (eg. diagnosis, length of stay, illness severity)?

Sometimes

Page 6

Q6 When managing end-of-life care in your ICU, how often would you (or a member of your ICU team) contact the GP to tell them that their patient had died?

Always

Page 7

Q7 When/if you make contact with a patient's GP, which method(s) of communication do you use (more than one if applicable)?

Phone call

Page 8

Q8 What do you think are the main factors discouraging you from contacting a GP when their patient is in your ICU?

Lack of interest from gps

Page 9

Q9 Please comment on the following statement: "Effective communication between ICU doctors and GPs is likely to benefit ICU patients after their hospital discharge"

Disagree

Page 10

Q10 Please comment on the following statement: "Effective communication between ICU doctors and GPs is likely to benefit the relatives of ICU patients after their hospital discharge"

Agree

Page 11

Q11 Do you participate in any outpatient clinic following up patients who have had a previous ICU stay with critical illness

No

#16

COMPLETE

Collector: Web Link 2 (Web Link)
Started: Wednesday, June 08, 2016 3:50:18 PM
Last Modified: Wednesday, June 08, 2016 3:52:40 PM
Time Spent: 00:02:22
IP Address: 137.191.232.3

Page 1

Q1 Which of the following best describes your current professional position?

Consultant with >/=50% clinical duties in ICU

Page 2

Q2 In which type of hospital do you conduct most of your intensive care work?

University-affiliated hospital

Page 3

Q3 When managing patients in your ICU, how often would you (or a member of your ICU team) make direct contact, for any purpose, with the patient's GP?

Occasionally

Page 4

Q4 If you (or a member of your ICU team) make contact with a patient's GP, what is the purpose of this communication?

To find out details leading to the patient's current illness	**Occasionally**
To find out about patient's background medical and/or social history	**Never**
To find out about patient's regular medications and/or allergies	**Never**
To inform the GP that their patient has been admitted to ICU	**Occasionally**
To inform the GP about details of the patient's ICU stay (eg. diagnosis, length of stay and/or illness severity)	**Occasionally**

Page 5

Q5 When managing patients in your ICU, how often would you (or a member of your ICU team) contact the GP either during or after ICU admission to give them details about their patient's ICU stay (eg. diagnosis, length of stay, illness severity)?

Always

Page 6

Q6 When managing end-of-life care in your ICU, how often would you (or a member of your ICU team) contact the GP to tell them that their patient had died?

Always

Page 7

Q7 When/if you make contact with a patient's GP, which method(s) of communication do you use (more than one if applicable)?

Phone call,

Written letter

Page 8

Q8 What do you think are the main factors discouraging you from contacting a GP when their patient is in your ICU?

Unknown GP

Page 9

Q9 Please comment on the following statement: "Effective communication between ICU doctors and GPs is likely to benefit ICU patients after their hospital discharge"

Agree

Page 10

Q10 Please comment on the following statement: "Effective communication between ICU doctors and GPs is likely to benefit the relatives of ICU patients after their hospital discharge"

Neutral

Page 11

Q11 Do you participate in any outpatient clinic following up patients who have had a previous ICU stay with critical illness

Yes

#17

COMPLETE

Collector: Web Link 2 (Web Link)
Started: Wednesday, June 08, 2016 9:02:16 PM
Last Modified: Wednesday, June 08, 2016 9:13:54 PM
Time Spent: 00:11:38
IP Address: 86.20.169.166

Page 1

Q1 Which of the following best describes your current professional position?

Consultant with >/=50% clinical duties in ICU

Page 2

Q2 In which type of hospital do you conduct most of your intensive care work?

University-affiliated hospital

Page 3

Q3 When managing patients in your ICU, how often would you (or a member of your ICU team) make direct contact, for any purpose, with the patient's GP?

Sometimes

Page 4

Q4 If you (or a member of your ICU team) make contact with a patient's GP, what is the purpose of this communication?

To inform the GP that their patient has been admitted to ICU	**Sometimes**
To inform the GP about details of the patient's ICU stay (eg. diagnosis, length of stay and/or illness severity)	**Sometimes**
Other (please specify)	**All PICU patients admitted via our retrieval service have a discharge letter documenting their stay sent to their GP. All elective or unplanned admissions from within our institution have a discharge letter sent by their primary team to the GP on discharge. this will include some details of their PICU stay.**

Page 5

Q5 When managing patients in your ICU, how often would you (or a member of your ICU team) contact the GP either during or after ICU admission to give them details about their patient's ICU stay (eg. diagnosis, length of stay, illness severity)?

Sometimes

Page 6

Q6 When managing end-of-life care in your ICU, how often would you (or a member of your ICU team) contact the GP to tell them that their patient had died?

Always

Page 7

Q7 When/if you make contact with a patient's GP, which method(s) of communication do you use (more than one if applicable)?

Written letter

Page 8

Q8 What do you think are the main factors discouraging you from contacting a GP when their patient is in your ICU?

Our patients have a letter directly sent to their GP by PICU if they were retrieved to the PICU or if they died while in PICU. Otherwise all admission from within the institution have a discharge sent to GP by primary team which should include some details of their PICU stay.

Page 9

Q9 Please comment on the following statement: "Effective communication between ICU doctors and GPs is likely to benefit ICU patients after their hospital discharge"

Agree,
Please explain your answer:
It may help to facilitate better understanding of underlying pathology particularly in complex patients. It may also act as a red flag in in conditions such as asthma. It can also help in providing parental and family support post PICU discharge, particularly if there have been decisions regarding escalation or escalation of therapy. It is also useful where a child has died and the family may need support from the GP

Page 10

Q10 Please comment on the following statement: "Effective communication between ICU doctors and GPs is likely to benefit the relatives of ICU patients after their hospital discharge"

Agree

Page 11

**Q11** Do you participate in any outpatient clinic following up patients who have had a previous ICU stay with critical illness	**No**

#18

COMPLETE

Collector: Web Link 3 (Web Link)
Started: Wednesday, June 15, 2016 7:57:22 AM
Last Modified: Wednesday, June 15, 2016 7:59:47 AM
Time Spent: 00:02:25
IP Address: 82.141.205.26

Page 1

Q1 Which of the following best describes your current professional position?

Consultant with <50% clinical duties in ICU

Page 2

Q2 In which type of hospital do you conduct most of your intensive care work?

University-affiliated hospital

Page 3

Q3 When managing patients in your ICU, how often would you (or a member of your ICU team) make direct contact, for any purpose, with the patient's GP?

Occasionally

Page 4

Q4 If you (or a member of your ICU team) make contact with a patient's GP, what is the purpose of this communication?

To find out details leading to the patient's current illness	**Occasionally**
To find out about patient's background medical and/or social history	**Occasionally**
To find out about patient's regular medications and/or allergies	**Occasionally**
To inform the GP that their patient has been admitted to ICU	**Occasionally**
To inform the GP about details of the patient's ICU stay (eg. diagnosis, length of stay and/or illness severity)	**Occasionally**

Page 5

Q5 When managing patients in your ICU, how often would you (or a member of your ICU team) contact the GP either during or after ICU admission to give them details about their patient's ICU stay (eg. diagnosis, length of stay, illness severity)?

Occasionally

Page 6

Q6 When managing end-of-life care in your ICU, how often would you (or a member of your ICU team) contact the GP to tell them that their patient had died?

Occasionally

Page 7

Q7 When/if you make contact with a patient's GP, which method(s) of communication do you use (more than one if applicable)?

Phone call

Page 8

Q8 What do you think are the main factors discouraging you from contacting a GP when their patient is in your ICU?

None

Page 9

Q9 Please comment on the following statement: "Effective communication between ICU doctors and GPs is likely to benefit ICU patients after their hospital discharge"

Neutral

Page 10

Q10 Please comment on the following statement: "Effective communication between ICU doctors and GPs is likely to benefit the relatives of ICU patients after their hospital discharge"

Agree

Page 11

Q11 Do you participate in any outpatient clinic following up patients who have had a previous ICU stay with critical illness

No

#19

COMPLETE

Collector: Web Link 3 (Web Link)
Started: Wednesday, June 15, 2016 8:02:27 AM
Last Modified: Wednesday, June 15, 2016 8:05:22 AM
Time Spent: 00:02:55
IP Address: 137.191.238.101

Page 1

Q1 Which of the following best describes your current professional position?

Other (please specify):
Clinical director

Page 2

Q2 In which type of hospital do you conduct most of your intensive care work?

University-affiliated hospital

Page 3

Q3 When managing patients in your ICU, how often would you (or a member of your ICU team) make direct contact, for any purpose, with the patient's GP?

Sometimes

Page 4

Q4 If you (or a member of your ICU team) make contact with a patient's GP, what is the purpose of this communication?

To find out details leading to the patient's current illness	**Often**
To find out about patient's background medical and/or social history	**Often**
To find out about patient's regular medications and/or allergies	**Often**
To inform the GP that their patient has been admitted to ICU	**Occasionally**
To inform the GP about details of the patient's ICU stay (eg. diagnosis, length of stay and/or illness severity)	**Never**

Page 5

Q5 When managing patients in your ICU, how often would you (or a member of your ICU team) contact the GP either during or after ICU admission to give them details about their patient's ICU stay (eg. diagnosis, length of stay, illness severity)?

Never

Page 6

Q6 When managing end-of-life care in your ICU, how often would you (or a member of your ICU team) contact the GP to tell them that their patient had died?

Never

Page 7

Q7 When/if you make contact with a patient's GP, which method(s) of communication do you use (more than one if applicable)?

Phone call

Page 8

Q8 What do you think are the main factors discouraging you from contacting a GP when their patient is in your ICU?

Unsure if they wish to be contacted

Page 9

Q9 Please comment on the following statement: "Effective communication between ICU doctors and GPs is likely to benefit ICU patients after their hospital discharge"

Strongly agree

Page 10

Q10 Please comment on the following statement: "Effective communication between ICU doctors and GPs is likely to benefit the relatives of ICU patients after their hospital discharge"

Strongly agree

Page 11

Q11 Do you participate in any outpatient clinic following up patients who have had a previous ICU stay with critical illness

No

#20

COMPLETE

Collector: Web Link 3 (Web Link)
Started: Wednesday, June 15, 2016 8:34:11 AM
Last Modified: Wednesday, June 15, 2016 9:09:39 AM
Time Spent: 00:35:28
IP Address: 95.83.254.108

Page 1

Q1 Which of the following best describes your current professional position?

Consultant with >/=50% clinical duties in ICU

Page 2

Q2 In which type of hospital do you conduct most of your intensive care work?

University-affiliated hospital

Page 3

Q3 When managing patients in your ICU, how often would you (or a member of your ICU team) make direct contact, for any purpose, with the patient's GP?

Occasionally

Page 4

Q4 If you (or a member of your ICU team) make contact with a patient's GP, what is the purpose of this communication?

To find out details leading to the patient's current illness	**Occasionally**
To find out about patient's background medical and/or social history	**Occasionally**
To find out about patient's regular medications and/or allergies	**Never**
To inform the GP that their patient has been admitted to ICU	**Occasionally**
To inform the GP about details of the patient's ICU stay (eg. diagnosis, length of stay and/or illness severity)	**Occasionally**
Other (please specify)	**Most nicu patients have never seen gp so informed on discharge**

Page 5

Q5 When managing patients in your ICU, how often would you (or a member of your ICU team) contact the GP either during or after ICU admission to give them details about their patient's ICU stay (eg. diagnosis, length of stay, illness severity)?

Often

Page 6

Q6 When managing end-of-life care in your ICU, how often would you (or a member of your ICU team) contact the GP to tell them that their patient had died?

Always

Page 7

Q7 When/if you make contact with a patient's GP, which method(s) of communication do you use (more than one if applicable)?

Phone call,

Written letter

Page 8

Q8 What do you think are the main factors discouraging you from contacting a GP when their patient is in your ICU?

They wish to know on discharge as new born infants and also all follow up except vaccination with neonatologists

Page 9

Q9 Please comment on the following statement: "Effective communication between ICU doctors and GPs is likely to benefit ICU patients after their hospital discharge"

Agree

Page 10

Q10 Please comment on the following statement: "Effective communication between ICU doctors and GPs is likely to benefit the relatives of ICU patients after their hospital discharge"

Strongly agree

Page 11

Q11 Do you participate in any outpatient clinic following up patients who have had a previous ICU stay with critical illness

Yes

#21

COMPLETE

Collector: Web Link 3 (Web Link)
Started: Wednesday, June 15, 2016 9:58:32 AM
Last Modified: Wednesday, June 15, 2016 10:00:14 AM
Time Spent: 00:01:42
IP Address: 137.191.238.101

Page 1

Q1 Which of the following best describes your current professional position?

Consultant with >/=50% clinical duties in ICU

Page 2

Q2 In which type of hospital do you conduct most of your intensive care work?

University-affiliated hospital

Page 3

Q3 When managing patients in your ICU, how often would you (or a member of your ICU team) make direct contact, for any purpose, with the patient's GP?

Sometimes

Page 4

Q4 If you (or a member of your ICU team) make contact with a patient's GP, what is the purpose of this communication?

To find out details leading to the patient's current illness	**Always**
To find out about patient's background medical and/or social history	**Always**

Page 5

Q5 When managing patients in your ICU, how often would you (or a member of your ICU team) contact the GP either during or after ICU admission to give them details about their patient's ICU stay (eg. diagnosis, length of stay, illness severity)?

Never

Page 6

Q6 When managing end-of-life care in your ICU, how often would you (or a member of your ICU team) contact the GP to tell them that their patient had died?

Never

Page 7

Q7 When/if you make contact with a patient's GP, which method(s) of communication do you use (more than one if applicable)?

Phone call

Page 8

Q8 What do you think are the main factors discouraging you from contacting a GP when their patient is in your ICU?

I dont think the have anything to contribute

Page 9

Q9 Please comment on the following statement: "Effective communication between ICU doctors and GPs is likely to benefit ICU patients after their hospital discharge"

Neutral

Page 10

Q10 Please comment on the following statement: "Effective communication between ICU doctors and GPs is likely to benefit the relatives of ICU patients after their hospital discharge"

Neutral

Page 11

Q11 Do you participate in any outpatient clinic following up patients who have had a previous ICU stay with critical illness

No

#22

COMPLETE

Collector: Web Link 3 (Web Link)
Started: Wednesday, June 15, 2016 10:01:15 AM
Last Modified: Wednesday, June 15, 2016 10:03:47 AM
Time Spent: 00:02:32
IP Address: 89.100.143.21

Page 1

Q1 Which of the following best describes your current professional position?

Consultant with >/=50% clinical duties in ICU

Page 2

Q2 In which type of hospital do you conduct most of your intensive care work?

University-affiliated hospital

Page 3

Q3 When managing patients in your ICU, how often would you (or a member of your ICU team) make direct contact, for any purpose, with the patient's GP?

Often

Page 4

Q4 If you (or a member of your ICU team) make contact with a patient's GP, what is the purpose of this communication?

To find out details leading to the patient's current illness	**Always**
To find out about patient's background medical and/or social history	**Always**
To find out about patient's regular medications and/or allergies	**Always**
To inform the GP that their patient has been admitted to ICU	**Sometimes**
To inform the GP about details of the patient's ICU stay (eg. diagnosis, length of stay and/or illness severity)	**Sometimes**

Page 5

Q5 When managing patients in your ICU, how often would you (or a member of your ICU team) contact the GP either during or after ICU admission to give them details about their patient's ICU stay (eg. diagnosis, length of stay, illness severity)?

Never

Page 6

Q6 When managing end-of-life care in your ICU, how often would you (or a member of your ICU team) contact the GP to tell them that their patient had died?

Occasionally

Page 7

Q7 When/if you make contact with a patient's GP, which method(s) of communication do you use (more than one if applicable)?

Phone call,

Written letter

Page 8

Q8 What do you think are the main factors discouraging you from contacting a GP when their patient is in your ICU?

TIME

Page 9

Q9 Please comment on the following statement: "Effective communication between ICU doctors and GPs is likely to benefit ICU patients after their hospital discharge"

Agree

Page 10

Q10 Please comment on the following statement: "Effective communication between ICU doctors and GPs is likely to benefit the relatives of ICU patients after their hospital discharge"

Neutral

Page 11

Q11 Do you participate in any outpatient clinic following up patients who have had a previous ICU stay with critical illness

No

#23

COMPLETE

Collector: Web Link 3 (Web Link)
Started: Wednesday, June 15, 2016 10:22:31 AM
Last Modified: Wednesday, June 15, 2016 10:25:39 AM
Time Spent: 00:03:08
IP Address: 137.191.226.226

Page 1

Q1 Which of the following best describes your current professional position?

Consultant with <50% clinical duties in ICU

Page 2

Q2 In which type of hospital do you conduct most of your intensive care work?

University-affiliated hospital

Page 3

Q3 When managing patients in your ICU, how often would you (or a member of your ICU team) make direct contact, for any purpose, with the patient's GP?

Sometimes

Page 4

Q4 If you (or a member of your ICU team) make contact with a patient's GP, what is the purpose of this communication?

To find out details leading to the patient's current illness	**Occasionally**
To find out about patient's background medical and/or social history	**Occasionally**
To find out about patient's regular medications and/or allergies	**Occasionally**
To inform the GP that their patient has been admitted to ICU	**Occasionally**
To inform the GP about details of the patient's ICU stay (eg. diagnosis, length of stay and/or illness severity)	**Occasionally**

Page 5

Q5 When managing patients in your ICU, how often would you (or a member of your ICU team) contact the GP either during or after ICU admission to give them details about their patient's ICU stay (eg. diagnosis, length of stay, illness severity)?

Occasionally

Page 6

Q6 When managing end-of-life care in your ICU, how often would you (or a member of your ICU team) contact the GP to tell them that their patient had died?

Occasionally

Page 7

Q7 When/if you make contact with a patient's GP, which method(s) of communication do you use (more than one if applicable)?

Phone call

Page 8

Q8 What do you think are the main factors discouraging you from contacting a GP when their patient is in your ICU?

none, GP name & phone contact ascertained at ICU admission

Page 9

Q9 Please comment on the following statement: "Effective communication between ICU doctors and GPs is likely to benefit ICU patients after their hospital discharge"

Agree

Page 10

Q10 Please comment on the following statement: "Effective communication between ICU doctors and GPs is likely to benefit the relatives of ICU patients after their hospital discharge"

Agree

Page 11

Q11 Do you participate in any outpatient clinic following up patients who have had a previous ICU stay with critical illness

No

#24

COMPLETE

Collector: Web Link 3 (Web Link)
Started: Wednesday, June 15, 2016 12:54:22 PM
Last Modified: Wednesday, June 15, 2016 12:58:54 PM
Time Spent: 00:04:32
IP Address: 137.191.238.101

Page 1

Q1 Which of the following best describes your current professional position?

Consultant with >/=50% clinical duties in ICU

Page 2

Q2 In which type of hospital do you conduct most of your intensive care work?

University-affiliated hospital

Page 3

Q3 When managing patients in your ICU, how often would you (or a member of your ICU team) make direct contact, for any purpose, with the patient's GP?

Sometimes

Page 4

Q4 If you (or a member of your ICU team) make contact with a patient's GP, what is the purpose of this communication?

To find out details leading to the patient's current illness	**Sometimes**
To find out about patient's background medical and/or social history	**Often**
To find out about patient's regular medications and/or allergies	**Often**
To inform the GP that their patient has been admitted to ICU	**Never**
To inform the GP about details of the patient's ICU stay (eg. diagnosis, length of stay and/or illness severity)	**Never**

Page 5

Q5 When managing patients in your ICU, how often would you (or a member of your ICU team) contact the GP either during or after ICU admission to give them details about their patient's ICU stay (eg. diagnosis, length of stay, illness severity)?

Never

Page 6

Q6 When managing end-of-life care in your ICU, how often would you (or a member of your ICU team) contact the GP to tell them that their patient had died?

Never

Page 7

Q7 When/if you make contact with a patient's GP, which method(s) of communication do you use (more than one if applicable)?

Phone call

Page 8

Q8 What do you think are the main factors discouraging you from contacting a GP when their patient is in your ICU?

A combination of not having the time and not thinking about it as a useful resource

Page 9

Q9 Please comment on the following statement: "Effective communication between ICU doctors and GPs is likely to benefit ICU patients after their hospital discharge"

Agree,
Please explain your answer:
certainly some possible benefit as GPs are the doctors that will see patients most frequently and would be able to explain the significance of aspects of the ICU illness to the pateints

Page 10

Q10 Please comment on the following statement: "Effective communication between ICU doctors and GPs is likely to benefit the relatives of ICU patients after their hospital discharge"

Neutral,
Please explain your answer:
unsure about this. perhaps GP could help relatives understand the nature of patient's illness and what is being done and why

Page 11

Q11 Do you participate in any outpatient clinic following up patients who have had a previous ICU stay with critical illness

No

#25

COMPLETE

Collector: Web Link 3 (Web Link)
Started: Thursday, June 16, 2016 1:47:21 AM
Last Modified: Thursday, June 16, 2016 1:50:53 AM
Time Spent: 00:03:32
IP Address: 178.167.254.14

Page 1

Q1 Which of the following best describes your current professional position?

Consultant with >/=50% clinical duties in ICU

Page 2

Q2 In which type of hospital do you conduct most of your intensive care work?

University-affiliated hospital

Page 3

Q3 When managing patients in your ICU, how often would you (or a member of your ICU team) make direct contact, for any purpose, with the patient's GP?

Occasionally

Page 4

Q4 If you (or a member of your ICU team) make contact with a patient's GP, what is the purpose of this communication?

To find out details leading to the patient's current illness	**Occasionally**
To find out about patient's background medical and/or social history	**Occasionally**
To find out about patient's regular medications and/or allergies	**Often**
To inform the GP that their patient has been admitted to ICU	**Never**
To inform the GP about details of the patient's ICU stay (eg. diagnosis, length of stay and/or illness severity)	**Never**

Page 5

Q5 When managing patients in your ICU, how often would you (or a member of your ICU team) contact the GP either during or after ICU admission to give them details about their patient's ICU stay (eg. diagnosis, length of stay, illness severity)?

Never

Page 6

Q6 When managing end-of-life care in your ICU, how often would you (or a member of your ICU team) contact the GP to tell them that their patient had died?

Never

Page 7

Q7 When/if you make contact with a patient's GP, which method(s) of communication do you use (more than one if applicable)?

Phone call

Page 8

Q8 What do you think are the main factors discouraging you from contacting a GP when their patient is in your ICU?

No routine culture of doing so

Page 9

Q9 Please comment on the following statement: "Effective communication between ICU doctors and GPs is likely to benefit ICU patients after their hospital discharge"

Agree

Page 10

Q10 Please comment on the following statement: "Effective communication between ICU doctors and GPs is likely to benefit the relatives of ICU patients after their hospital discharge"

Agree

Page 11

Q11 Do you participate in any outpatient clinic following up patients who have had a previous ICU stay with critical illness

No

#26

COMPLETE

Collector: Web Link 3 (Web Link)
Started: Thursday, June 16, 2016 6:30:52 AM
Last Modified: Thursday, June 16, 2016 6:35:46 AM
Time Spent: 00:04:54
IP Address: 213.233.132.154

Page 1

Q1 Which of the following best describes your current professional position?

Consultant with >/=50% clinical duties in ICU

Page 2

Q2 In which type of hospital do you conduct most of your intensive care work?

University-affiliated hospital

Page 3

Q3 When managing patients in your ICU, how often would you (or a member of your ICU team) make direct contact, for any purpose, with the patient's GP?

Sometimes

Page 4

Q4 If you (or a member of your ICU team) make contact with a patient's GP, what is the purpose of this communication?

To find out details leading to the patient's current illness	**Sometimes**
To find out about patient's background medical and/or social history	**Sometimes**
To find out about patient's regular medications and/or allergies	**Sometimes**
To inform the GP that their patient has been admitted to ICU	**Never**
To inform the GP about details of the patient's ICU stay (eg. diagnosis, length of stay and/or illness severity)	**Never**

Page 5

Q5 When managing patients in your ICU, how often would you (or a member of your ICU team) contact the GP either during or after ICU admission to give them details about their patient's ICU stay (eg. diagnosis, length of stay, illness severity)?

Never

Page 6

Q6 When managing end-of-life care in your ICU, how often would you (or a member of your ICU team) contact the GP to tell them that their patient had died?

Occasionally

Page 7

Q7 When/if you make contact with a patient's GP, which method(s) of communication do you use (more than one if applicable)?

Phone call

Page 8

Q8 What do you think are the main factors discouraging you from contacting a GP when their patient is in your ICU?

Not viewing the patient journey as a whole from hospital to community

Page 9

Q9 Please comment on the following statement: "Effective communication between ICU doctors and GPs is likely to benefit ICU patients after their hospital discharge"

Strongly agree

Page 10

Q10 Please comment on the following statement: "Effective communication between ICU doctors and GPs is likely to benefit the relatives of ICU patients after their hospital discharge"

Strongly agree

Page 11

Q11 Do you participate in any outpatient clinic following up patients who have had a previous ICU stay with critical illness

No

#27

COMPLETE

Collector: Web Link 3 (Web Link)
Started: Thursday, June 16, 2016 6:35:28 AM
Last Modified: Thursday, June 16, 2016 6:41:24 AM
Time Spent: 00:05:56
IP Address: 185.51.73.75

Page 1

Q1 Which of the following best describes your current professional position?

Consultant with <50% clinical duties in ICU

Page 2

Q2 In which type of hospital do you conduct most of your intensive care work?

University-affiliated hospital

Page 3

Q3 When managing patients in your ICU, how often would you (or a member of your ICU team) make direct contact, for any purpose, with the patient's GP?

Occasionally

Page 4

Q4 If you (or a member of your ICU team) make contact with a patient's GP, what is the purpose of this communication?

To find out details leading to the patient's current illness	**Often**
To find out about patient's background medical and/or social history	**Often**
To find out about patient's regular medications and/or allergies	**Often**
To inform the GP that their patient has been admitted to ICU	**Never**
To inform the GP about details of the patient's ICU stay (eg. diagnosis, length of stay and/or illness severity)	**Never**

Page 5

Q5 When managing patients in your ICU, how often would you (or a member of your ICU team) contact the GP either during or after ICU admission to give them details about their patient's ICU stay (eg. diagnosis, length of stay, illness severity)?

Never

Page 6

Q6 When managing end-of-life care in your ICU, how often would you (or a member of your ICU team) contact the GP to tell them that their patient had died?

Never

Page 7

Q7 When/if you make contact with a patient's GP, which method(s) of communication do you use (more than one if applicable)?

Phone call

Page 8

Q8 What do you think are the main factors discouraging you from contacting a GP when their patient is in your ICU?

Work overload on both sides, unsure about benefits of keeping a GP informed about their patient in ITU.

Page 9

Q9 Please comment on the following statement: "Effective communication between ICU doctors and GPs is likely to benefit ICU patients after their hospital discharge"

Neutral,
Please explain your answer:
ITU patients most often move on to the wards where they are taken care of by their primary teams. There is an assumption that the primary team carries responsibility of communicating with GPs at the time of discharge from hospital.

Page 10

Q10 Please comment on the following statement: "Effective communication between ICU doctors and GPs is likely to benefit the relatives of ICU patients after their hospital discharge"

Agree,
Please explain your answer:
Again, not sure what is the evidence behind it. Long term consequences of ITU stay are managed further on the wards post ITU discharge and the primary team-ITU communication is more important here.

Page 11

Q11 Do you participate in any outpatient clinic following up patients who have had a previous ICU stay with critical illness

No

#28

COMPLETE

Collector: Web Link 3 (Web Link)
Started: Thursday, June 16, 2016 8:48:30 AM
Last Modified: Thursday, June 16, 2016 8:51:29 AM
Time Spent: 00:02:59
IP Address: 83.50.104.55

Page 1

Q1 Which of the following best describes your current professional position?

Consultant with >/=50% clinical duties in ICU

Page 2

Q2 In which type of hospital do you conduct most of your intensive care work?

University-affiliated hospital

Page 3

Q3 When managing patients in your ICU, how often would you (or a member of your ICU team) make direct contact, for any purpose, with the patient's GP?

Occasionally

Page 4

Q4 If you (or a member of your ICU team) make contact with a patient's GP, what is the purpose of this communication?

To find out details leading to the patient's current illness	**Occasionally**
To find out about patient's background medical and/or social history	**Occasionally**
To find out about patient's regular medications and/or allergies	**Occasionally**
To inform the GP that their patient has been admitted to ICU	**Never**
To inform the GP about details of the patient's ICU stay (eg. diagnosis, length of stay and/or illness severity)	**Never**

Page 5

Q5 When managing patients in your ICU, how often would you (or a member of your ICU team) contact the GP either during or after ICU admission to give them details about their patient's ICU stay (eg. diagnosis, length of stay, illness severity)?

Never

Page 6

Q6 When managing end-of-life care in your ICU, how often would you (or a member of your ICU team) contact the GP to tell them that their patient had died?

Occasionally

Page 7

Q7 When/if you make contact with a patient's GP, which method(s) of communication do you use (more than one if applicable)?

Phone call

Page 8

Q8 What do you think are the main factors discouraging you from contacting a GP when their patient is in your ICU?

Lack of time

Page 9

Q9 Please comment on the following statement: "Effective communication between ICU doctors and GPs is likely to benefit ICU patients after their hospital discharge"

Agree

Page 10

Q10 Please comment on the following statement: "Effective communication between ICU doctors and GPs is likely to benefit the relatives of ICU patients after their hospital discharge"

Agree

Page 11

Q11 Do you participate in any outpatient clinic following up patients who have had a previous ICU stay with critical illness

No

#29

COMPLETE

Collector: Web Link 3 (Web Link)
Started: Thursday, June 16, 2016 9:38:09 AM
Last Modified: Thursday, June 16, 2016 9:43:49 AM
Time Spent: 00:05:40
IP Address: 84.203.136.190

Page 1

Q1 Which of the following best describes your current professional position?

Consultant with >/=50% clinical duties in ICU

Page 2

Q2 In which type of hospital do you conduct most of your intensive care work?

University-affiliated hospital

Page 3

Q3 When managing patients in your ICU, how often would you (or a member of your ICU team) make direct contact, for any purpose, with the patient's GP?

Occasionally

Page 4

Q4 If you (or a member of your ICU team) make contact with a patient's GP, what is the purpose of this communication?

To find out details leading to the patient's current illness	**Occasionally**
To find out about patient's background medical and/or social history	**Occasionally**
To find out about patient's regular medications and/or allergies	**Sometimes**
To inform the GP that their patient has been admitted to ICU	**Never**
To inform the GP about details of the patient's ICU stay (eg. diagnosis, length of stay and/or illness severity)	**Never**

Page 5

Q5 When managing patients in your ICU, how often would you (or a member of your ICU team) contact the GP either during or after ICU admission to give them details about their patient's ICU stay (eg. diagnosis, length of stay, illness severity)?

Never

Page 6

Q6 When managing end-of-life care in your ICU, how often would you (or a member of your ICU team) contact the GP to tell them that their patient had died?

Occasionally

Page 7

Q7 When/if you make contact with a patient's GP, which method(s) of communication do you use (more than one if applicable)?

Phone call

Page 8

Q8 What do you think are the main factors discouraging you from contacting a GP when their patient is in your ICU?

Too much time required to inform every GP of their patient's admission to ICU. Also unsure of the GP's interest in this knowledge.

Page 9

Q9 Please comment on the following statement: "Effective communication between ICU doctors and GPs is likely to benefit ICU patients after their hospital discharge"

Neutral,
Please explain your answer:
Not sure what a GP will do differently with this knowledge. As they are usually extremely busy this information is likely to go unused.

Page 10

Q10 Please comment on the following statement: "Effective communication between ICU doctors and GPs is likely to benefit the relatives of ICU patients after their hospital discharge"

Agree

Page 11

Q11 Do you participate in any outpatient clinic following up patients who have had a previous ICU stay with critical illness

No

#30

COMPLETE

Collector: Web Link 3 (Web Link)
Started: Thursday, June 16, 2016 10:08:31 AM
Last Modified: Thursday, June 16, 2016 10:09:35 AM
Time Spent: 00:01:04
IP Address: 193.1.229.2

Page 1

Q1 Which of the following best describes your current professional position?

Consultant with <50% clinical duties in ICU

Page 2

Q2 In which type of hospital do you conduct most of your intensive care work?

University-affiliated hospital

Page 3

Q3 When managing patients in your ICU, how often would you (or a member of your ICU team) make direct contact, for any purpose, with the patient's GP?

Never

Page 4

Q4 If you (or a member of your ICU team) make contact with a patient's GP, what is the purpose of this communication?

Respondent skipped this question

Page 5

Q5 When managing patients in your ICU, how often would you (or a member of your ICU team) contact the GP either during or after ICU admission to give them details about their patient's ICU stay (eg. diagnosis, length of stay, illness severity)?

Respondent skipped this question

Page 6

Q6 When managing end-of-life care in your ICU, how often would you (or a member of your ICU team) contact the GP to tell them that their patient had died?

Respondent skipped this question

Page 7

Q7 When/if you make contact with a patient's GP, which method(s) of communication do you use (more than one if applicable)?

Respondent skipped this question

Page 8

Q8 What do you think are the main factors discouraging you from contacting a GP when their patient is in your ICU?

lack of time

Page 9

Q9 Please comment on the following statement: "Effective communication between ICU doctors and GPs is likely to benefit ICU patients after their hospital discharge"

Strongly agree

Page 10

Q10 Please comment on the following statement: "Effective communication between ICU doctors and GPs is likely to benefit the relatives of ICU patients after their hospital discharge"

Strongly agree

Page 11

Q11 Do you participate in any outpatient clinic following up patients who have had a previous ICU stay with critical illness

No

#31

COMPLETE

Collector: Web Link 3 (Web Link)
Started: Thursday, June 16, 2016 11:09:04 AM
Last Modified: Thursday, June 16, 2016 11:13:46 AM
Time Spent: 00:04:42
IP Address: 109.255.8.167

Page 1

Q1 Which of the following best describes your current professional position?

Consultant with <50% clinical duties in ICU

Page 2

Q2 In which type of hospital do you conduct most of your intensive care work?

University-affiliated hospital

Page 3

Q3 When managing patients in your ICU, how often would you (or a member of your ICU team) make direct contact, for any purpose, with the patient's GP?

Sometimes

Page 4

Q4 If you (or a member of your ICU team) make contact with a patient's GP, what is the purpose of this communication?

To find out details leading to the patient's current illness	**Sometimes**
To find out about patient's background medical and/or social history	**Often**
To find out about patient's regular medications and/or allergies	**Often**
To inform the GP that their patient has been admitted to ICU	**Never**
To inform the GP about details of the patient's ICU stay (eg. diagnosis, length of stay and/or illness severity)	**Never**

Page 5

Q5 When managing patients in your ICU, how often would you (or a member of your ICU team) contact the GP either during or after ICU admission to give them details about their patient's ICU stay (eg. diagnosis, length of stay, illness severity)?

Never

Page 6

Q6 When managing end-of-life care in your ICU, how often would you (or a member of your ICU team) contact the GP to tell them that their patient had died?

Never

Page 7

Q7 When/if you make contact with a patient's GP, which method(s) of communication do you use (more than one if applicable)?

Phone call

Page 8

Q8 What do you think are the main factors discouraging you from contacting a GP when their patient is in your ICU?

Discharge letter written by the primary admission team hopefully mentions about ICU admission

Page 9

Q9 Please comment on the following statement: "Effective communication between ICU doctors and GPs is likely to benefit ICU patients after their hospital discharge"

Agree

Page 10

Q10 Please comment on the following statement: "Effective communication between ICU doctors and GPs is likely to benefit the relatives of ICU patients after their hospital discharge"

Neutral

Page 11

Q11 Do you participate in any outpatient clinic following up patients who have had a previous ICU stay with critical illness

No

#32

COMPLETE

Collector: Web Link 3 (Web Link)
Started: Thursday, June 16, 2016 1:16:06 PM
Last Modified: Thursday, June 16, 2016 1:19:32 PM
Time Spent: 00:03:26
IP Address: 213.233.147.99

Page 1

Q1 Which of the following best describes your current professional position?

Consultant with <50% clinical duties in ICU

Page 2

Q2 In which type of hospital do you conduct most of your intensive care work?

University-affiliated hospital

Page 3

Q3 When managing patients in your ICU, how often would you (or a member of your ICU team) make direct contact, for any purpose, with the patient's GP?

Occasionally

Page 4

Q4 If you (or a member of your ICU team) make contact with a patient's GP, what is the purpose of this communication?

To find out details leading to the patient's current illness	**Occasionally**
To find out about patient's background medical and/or social history	**Often**
To find out about patient's regular medications and/or allergies	**Never**
To inform the GP that their patient has been admitted to ICU	**Occasionally**
To inform the GP about details of the patient's ICU stay (eg. diagnosis, length of stay and/or illness severity)	**Occasionally**

Page 5

Q5 When managing patients in your ICU, how often would you (or a member of your ICU team) contact the GP either during or after ICU admission to give them details about their patient's ICU stay (eg. diagnosis, length of stay, illness severity)?

Occasionally

Page 6

Q6 When managing end-of-life care in your ICU, how often would you (or a member of your ICU team) contact the GP to tell them that their patient had died?

Occasionally

Page 7

Q7 When/if you make contact with a patient's GP, which method(s) of communication do you use (more than one if applicable)?

Phone call

Page 8

Q8 What do you think are the main factors discouraging you from contacting a GP when their patient is in your ICU?

None in particular

Page 9

Q9 Please comment on the following statement: "Effective communication between ICU doctors and GPs is likely to benefit ICU patients after their hospital discharge"

Agree,
Please explain your answer:
Awareness by GP of nature of illness

Page 10

Q10 Please comment on the following statement: "Effective communication between ICU doctors and GPs is likely to benefit the relatives of ICU patients after their hospital discharge"

Agree,
Please explain your answer:
Gives perspective

Page 11

Q11 Do you participate in any outpatient clinic following up patients who have had a previous ICU stay with critical illness

No

#33

INCOMPLETE

Collector: Web Link 3 (Web Link)
Started: Thursday, June 16, 2016 2:41:04 PM
Last Modified: Thursday, June 16, 2016 2:42:49 PM
Time Spent: 00:01:45
IP Address: 89.101.141.162

Page 1

Q1 Which of the following best describes your current professional position?

Consultant with >/=50% clinical duties in ICU

Page 2

Q2 In which type of hospital do you conduct most of your intensive care work?

University-affiliated hospital

Page 3

Q3 When managing patients in your ICU, how often would you (or a member of your ICU team) make direct contact, for any purpose, with the patient's GP?

Occasionally

Page 4

Q4 If you (or a member of your ICU team) make contact with a patient's GP, what is the purpose of this communication?

To find out details leading to the patient's current illness	**Occasionally**
To find out about patient's background medical and/or social history	**Occasionally**
To find out about patient's regular medications and/or allergies	**Occasionally**
To inform the GP that their patient has been admitted to ICU	**Never**
To inform the GP about details of the patient's ICU stay (eg. diagnosis, length of stay and/or illness severity)	**Never**

Page 5

Q5 When managing patients in your ICU, how often would you (or a member of your ICU team) contact the GP either during or after ICU admission to give them details about their patient's ICU stay (eg. diagnosis, length of stay, illness severity)?

Occasionally

Page 6

Q6 When managing end-of-life care in your ICU, how often would you (or a member of your ICU team) contact the GP to tell them that their patient had died?

Never

Page 7

Q7 When/if you make contact with a patient's GP, which method(s) of communication do you use (more than one if applicable)?

Phone call

Page 8

Q8 What do you think are the main factors discouraging you from contacting a GP when their patient is in your ICU?

Respondent skipped this question

Page 9

Q9 Please comment on the following statement: "Effective communication between ICU doctors and GPs is likely to benefit ICU patients after their hospital discharge"

Respondent skipped this question

Page 10

Q10 Please comment on the following statement: "Effective communication between ICU doctors and GPs is likely to benefit the relatives of ICU patients after their hospital discharge"

Respondent skipped this question

Page 11

Q11 Do you participate in any outpatient clinic following up patients who have had a previous ICU stay with critical illness

Respondent skipped this question

#34

COMPLETE

Collector: Web Link 3 (Web Link)
Started: Thursday, June 16, 2016 4:02:13 PM
Last Modified: Thursday, June 16, 2016 4:04:57 PM
Time Spent: 00:02:44
IP Address: 137.191.240.37

Page 1

Q1 Which of the following best describes your current professional position?

Consultant with <50% clinical duties in ICU

Page 2

Q2 In which type of hospital do you conduct most of your intensive care work?

University-affiliated hospital

Page 3

Q3 When managing patients in your ICU, how often would you (or a member of your ICU team) make direct contact, for any purpose, with the patient's GP?

Occasionally

Page 4

Q4 If you (or a member of your ICU team) make contact with a patient's GP, what is the purpose of this communication?

To find out details leading to the patient's current illness	**Never**
To find out about patient's background medical and/or social history	**Never**
To find out about patient's regular medications and/or allergies	**Occasionally**
To inform the GP that their patient has been admitted to ICU	**Never**
To inform the GP about details of the patient's ICU stay (eg. diagnosis, length of stay and/or illness severity)	**Never**

Page 5

Q5 When managing patients in your ICU, how often would you (or a member of your ICU team) contact the GP either during or after ICU admission to give them details about their patient's ICU stay (eg. diagnosis, length of stay, illness severity)?

Never

Page 6

Q6 When managing end-of-life care in your ICU, how often would you (or a member of your ICU team) contact the GP to tell them that their patient had died?

Never

Page 7

Q7 When/if you make contact with a patient's GP, which method(s) of communication do you use (more than one if applicable)?

Phone call

Page 8

Q8 What do you think are the main factors discouraging you from contacting a GP when their patient is in your ICU?

Time. Ease of communication (would be more likely to do if email available)

Page 9

Q9 Please comment on the following statement: "Effective communication between ICU doctors and GPs is likely to benefit ICU patients after their hospital discharge"

Agree

Page 10

Q10 Please comment on the following statement: "Effective communication between ICU doctors and GPs is likely to benefit the relatives of ICU patients after their hospital discharge"

Strongly agree

Page 11

Q11 Do you participate in any outpatient clinic following up patients who have had a previous ICU stay with critical illness

No

#35

COMPLETE

Collector: Web Link 3 (Web Link)
Started: Thursday, June 16, 2016 5:38:00 PM
Last Modified: Thursday, June 16, 2016 5:44:12 PM
Time Spent: 00:06:12
IP Address: 89.101.132.226

Page 1

Q1 Which of the following best describes your current professional position?

Consultant with <50% clinical duties in ICU

Page 2

Q2 In which type of hospital do you conduct most of your intensive care work?

University-affiliated hospital

Page 3

Q3 When managing patients in your ICU, how often would you (or a member of your ICU team) make direct contact, for any purpose, with the patient's GP?

Occasionally

Page 4

Q4 If you (or a member of your ICU team) make contact with a patient's GP, what is the purpose of this communication?

To find out details leading to the patient's current illness	**Occasionally**
To find out about patient's background medical and/or social history	**Occasionally**
To find out about patient's regular medications and/or allergies	**Sometimes**
To inform the GP that their patient has been admitted to ICU	**Never**
To inform the GP about details of the patient's ICU stay (eg. diagnosis, length of stay and/or illness severity)	**Never**

Page 5

Q5 When managing patients in your ICU, how often would you (or a member of your ICU team) contact the GP either during or after ICU admission to give them details about their patient's ICU stay (eg. diagnosis, length of stay, illness severity)?

Never

Page 6

Q6 When managing end-of-life care in your ICU, how often would you (or a member of your ICU team) contact the GP to tell them that their patient had died?

Never

Page 7

Q7 When/if you make contact with a patient's GP, which method(s) of communication do you use (more than one if applicable)?

Phone call

Page 8

Q8 What do you think are the main factors discouraging you from contacting a GP when their patient is in your ICU?

Icu workload

Page 9

Q9 Please comment on the following statement: "Effective communication between ICU doctors and GPs is likely to benefit ICU patients after their hospital discharge"

Neutral,
Please explain your answer:
It is rare for patients to transition directly to the community before a stay in the hospital ward. The primary team should liaise with the GP and that is important for continuity. As a courtesy to the GP, they should be automatically contacted if their patient dies in Icu but it is an often overlooked detail

Page 10

Q10 Please comment on the following statement: "Effective communication between ICU doctors and GPs is likely to benefit the relatives of ICU patients after their hospital discharge"

Neutral

Page 11

Q11 Do you participate in any outpatient clinic following up patients who have had a previous ICU stay with critical illness

No

#36

COMPLETE

Collector: Web Link 3 (Web Link)
Started: Thursday, June 16, 2016 6:32:27 PM
Last Modified: Thursday, June 16, 2016 6:34:13 PM
Time Spent: 00:01:46
IP Address: 37.228.228.245

Page 1

Q1 Which of the following best describes your current professional position?

Consultant with >/=50% clinical duties in ICU

Page 2

Q2 In which type of hospital do you conduct most of your intensive care work?

University-affiliated hospital

Page 3

Q3 When managing patients in your ICU, how often would you (or a member of your ICU team) make direct contact, for any purpose, with the patient's GP?

Sometimes

Page 4

Q4 If you (or a member of your ICU team) make contact with a patient's GP, what is the purpose of this communication?

To find out details leading to the patient's current illness	**Often**
To find out about patient's background medical and/or social history	**Sometimes**
To find out about patient's regular medications and/or allergies	**Occasionally**
To inform the GP that their patient has been admitted to ICU	**Never**
To inform the GP about details of the patient's ICU stay (eg. diagnosis, length of stay and/or illness severity)	**Never**

Page 5

Q5 When managing patients in your ICU, how often would you (or a member of your ICU team) contact the GP either during or after ICU admission to give them details about their patient's ICU stay (eg. diagnosis, length of stay, illness severity)?

Never

Page 6

Q6 When managing end-of-life care in your ICU, how often would you (or a member of your ICU team) contact the GP to tell them that their patient had died?

Never

Page 7

Q7 When/if you make contact with a patient's GP, which method(s) of communication do you use (more than one if applicable)?

Phone call

Page 8

Q8 What do you think are the main factors discouraging you from contacting a GP when their patient is in your ICU?

Interest

Page 9

Q9 Please comment on the following statement: "Effective communication between ICU doctors and GPs is likely to benefit ICU patients after their hospital discharge"

Neutral

Page 10

Q10 Please comment on the following statement: "Effective communication between ICU doctors and GPs is likely to benefit the relatives of ICU patients after their hospital discharge"

Neutral

Page 11

Q11 Do you participate in any outpatient clinic following up patients who have had a previous ICU stay with critical illness

No

#37

COMPLETE

Collector: Web Link 3 (Web Link)
Started: Friday, June 17, 2016 11:37:10 AM
Last Modified: Friday, June 17, 2016 11:42:37 AM
Time Spent: 00:05:27
IP Address: 89.101.132.13

Page 1

Q1 Which of the following best describes your current professional position?

Consultant with >/=50% clinical duties in ICU

Page 2

Q2 In which type of hospital do you conduct most of your intensive care work?

University-affiliated hospital

Page 3

Q3 When managing patients in your ICU, how often would you (or a member of your ICU team) make direct contact, for any purpose, with the patient's GP?

Occasionally

Page 4

Q4 If you (or a member of your ICU team) make contact with a patient's GP, what is the purpose of this communication?

To find out details leading to the patient's current illness	**Sometimes**
To find out about patient's background medical and/or social history	**Sometimes**
To find out about patient's regular medications and/or allergies	**Never**
To inform the GP that their patient has been admitted to ICU	**Sometimes**
To inform the GP about details of the patient's ICU stay (eg. diagnosis, length of stay and/or illness severity)	**Sometimes**

Page 5

Q5 When managing patients in your ICU, how often would you (or a member of your ICU team) contact the GP either during or after ICU admission to give them details about their patient's ICU stay (eg. diagnosis, length of stay, illness severity)?

Occasionally

Page 6

Q6 When managing end-of-life care in your ICU, how often would you (or a member of your ICU team) contact the GP to tell them that their patient had died?

Occasionally

Page 7

Q7 When/if you make contact with a patient's GP, which method(s) of communication do you use (more than one if applicable)?

Phone call

Page 8

Q8 What do you think are the main factors discouraging you from contacting a GP when their patient is in your ICU?

Ease of communicstion

Page 9

Q9 Please comment on the following statement: "Effective communication between ICU doctors and GPs is likely to benefit ICU patients after their hospital discharge"

Strongly agree,
Please explain your answer:
Often relevant in end of life decision making is my main reason to ring. Quality of life/what their wishes would have been

Page 10

Q10 Please comment on the following statement: "Effective communication between ICU doctors and GPs is likely to benefit the relatives of ICU patients after their hospital discharge"

Strongly agree,
Please explain your answer:
Having GP on board is very helpful. Families have better (longer) established and more trusting relationship with Gp and family perceive that GP would have good insight into their relatives wishes

Page 11

Q11 Do you participate in any outpatient clinic following up patients who have had a previous ICU stay with critical illness

No

#38

COMPLETE

Collector: Web Link 3 (Web Link)
Started: Friday, June 17, 2016 6:26:50 PM
Last Modified: Friday, June 17, 2016 6:32:06 PM
Time Spent: 00:05:16
IP Address: 193.178.96.131

Page 1

Q1 Which of the following best describes your current professional position?

Consultant with <50% clinical duties in ICU

Page 2

Q2 In which type of hospital do you conduct most of your intensive care work?

University-affiliated hospital

Page 3

Q3 When managing patients in your ICU, how often would you (or a member of your ICU team) make direct contact, for any purpose, with the patient's GP?

Occasionally

Page 4

Q4 If you (or a member of your ICU team) make contact with a patient's GP, what is the purpose of this communication?

To find out details leading to the patient's current illness	**Sometimes**
To find out about patient's background medical and/or social history	**Sometimes**
To find out about patient's regular medications and/or allergies	**Occasionally**
To inform the GP that their patient has been admitted to ICU	**Occasionally**
To inform the GP about details of the patient's ICU stay (eg. diagnosis, length of stay and/or illness severity)	**Never**

Page 5

Q5 When managing patients in your ICU, how often would you (or a member of your ICU team) contact the GP either during or after ICU admission to give them details about their patient's ICU stay (eg. diagnosis, length of stay, illness severity)?

Never

Page 6

Q6 When managing end-of-life care in your ICU, how often would you (or a member of your ICU team) contact the GP to tell them that their patient had died?

Never

Page 7

Q7 When/if you make contact with a patient's GP, which method(s) of communication do you use (more than one if applicable)?

Phone call

Page 8

Q8 What do you think are the main factors discouraging you from contacting a GP when their patient is in your ICU?

Unsure what GP would contribute that family or primary care doctor cannot tell us

Page 9

Q9 Please comment on the following statement: "Effective communication between ICU doctors and GPs is likely to benefit ICU patients after their hospital discharge"

Agree

Page 10

Q10 Please comment on the following statement: "Effective communication between ICU doctors and GPs is likely to benefit the relatives of ICU patients after their hospital discharge"

Agree

Page 11

Q11 Do you participate in any outpatient clinic following up patients who have had a previous ICU stay with critical illness

No

#39

COMPLETE

Collector: Web Link 3 (Web Link)
Started: Saturday, June 18, 2016 8:36:48 AM
Last Modified: Saturday, June 18, 2016 8:43:17 AM
Time Spent: 00:06:29
IP Address: 185.51.73.252

Page 1

Q1 Which of the following best describes your current professional position?

Consultant with <50% clinical duties in ICU

Page 2

Q2 In which type of hospital do you conduct most of your intensive care work?

University-affiliated hospital

Page 3

Q3 When managing patients in your ICU, how often would you (or a member of your ICU team) make direct contact, for any purpose, with the patient's GP?

Always

Page 4

Q4 If you (or a member of your ICU team) make contact with a patient's GP, what is the purpose of this communication?

To find out details leading to the patient's current illness	**Sometimes**
To find out about patient's background medical and/or social history	**Always**
To find out about patient's regular medications and/or allergies	**Always**
To inform the GP that their patient has been admitted to ICU	**Never**
To inform the GP about details of the patient's ICU stay (eg. diagnosis, length of stay and/or illness severity)	**Never**
Other (please specify)	**The contact is made by the ICU PHARMACIST. She will get up to date medication list**

Page 5

Q5 When managing patients in your ICU, how often would you (or a member of your ICU team) contact the GP either during or after ICU admission to give them details about their patient's ICU stay (eg. diagnosis, length of stay, illness severity)?

Never

Page 6

Q6 When managing end-of-life care in your ICU, how often would you (or a member of your ICU team) contact the GP to tell them that their patient had died?

Occasionally

Page 7

Q7 When/if you make contact with a patient's GP, which method(s) of communication do you use (more than one if applicable)?

Phone call

Page 8

Q8 What do you think are the main factors discouraging you from contacting a GP when their patient is in your ICU?

If sufficient background information are available or if patient hasn't attended GP in recent past

Page 9

Q9 Please comment on the following statement: "Effective communication between ICU doctors and GPs is likely to benefit ICU patients after their hospital discharge"

Agree,
Please explain your answer:
Primary teams communication to GP at discharge is unlikely to include appropriate ICU stay details

Page 10

Q10 Please comment on the following statement: "Effective communication between ICU doctors and GPs is likely to benefit the relatives of ICU patients after their hospital discharge"

Agree,
Please explain your answer:
The GP may be able to answer questions about their relatives stay

Page 11

**Q11** Do you participate in any outpatient clinic following up patients who have had a previous ICU stay with critical illness	**No**

#40

COMPLETE

Collector: Web Link 3 (Web Link)
Started: Sunday, June 19, 2016 9:38:25 PM
Last Modified: Sunday, June 19, 2016 9:43:17 PM
Time Spent: 00:04:52
IP Address: 86.40.40.74

Page 1

Q1 Which of the following best describes your current professional position?

Consultant with <50% clinical duties in ICU

Page 2

Q2 In which type of hospital do you conduct most of your intensive care work?

University-affiliated hospital

Page 3

Q3 When managing patients in your ICU, how often would you (or a member of your ICU team) make direct contact, for any purpose, with the patient's GP?

Sometimes

Page 4

Q4 If you (or a member of your ICU team) make contact with a patient's GP, what is the purpose of this communication?

To find out details leading to the patient's current illness	**Sometimes**
To find out about patient's background medical and/or social history	**Sometimes**
To find out about patient's regular medications and/or allergies	**Often**
To inform the GP that their patient has been admitted to ICU	**Never**
To inform the GP about details of the patient's ICU stay (eg. diagnosis, length of stay and/or illness severity)	**Occasionally**
Other (please specify)	**re 4 and 5 above...occasional contact inititated by GP also**

Page 5

Q5 When managing patients in your ICU, how often would you (or a member of your ICU team) contact the GP either during or after ICU admission to give them details about their patient's ICU stay (eg. diagnosis, length of stay, illness severity)?

Occasionally

Page 6

Q6 When managing end-of-life care in your ICU, how often would you (or a member of your ICU team) contact the GP to tell them that their patient had died?

Occasionally

Page 7

Q7 When/if you make contact with a patient's GP, which method(s) of communication do you use (more than one if applicable)?

Phone call

Page 8

Q8 What do you think are the main factors discouraging you from contacting a GP when their patient is in your ICU?

time, not often felt necessary

Page 9

Q9 Please comment on the following statement: "Effective communication between ICU doctors and GPs is likely to benefit ICU patients after their hospital discharge"

Agree,
Please explain your answer:
Likely to aid in recovery from ICU (as opposed to recovery from primary problem itself)

Page 10

Q10 Please comment on the following statement: "Effective communication between ICU doctors and GPs is likely to benefit the relatives of ICU patients after their hospital discharge"

Agree,
Please explain your answer:
Likely to aid in recovery from ICU (as opposed to recovery from primary problem itself)....and possibly expectations for future

Page 11

**Q11** Do you participate in any outpatient clinic following up patients who have had a previous ICU stay with critical illness	**No**

#41

COMPLETE

Collector: Web Link 3 (Web Link)
Started: Monday, June 20, 2016 10:29:19 AM
Last Modified: Monday, June 20, 2016 10:31:37 AM
Time Spent: 00:02:18
IP Address: 109.78.150.179

Page 1

Q1 Which of the following best describes your current professional position?

Consultant with <50% clinical duties in ICU

Page 2

Q2 In which type of hospital do you conduct most of your intensive care work?

University-affiliated hospital

Page 3

Q3 When managing patients in your ICU, how often would you (or a member of your ICU team) make direct contact, for any purpose, with the patient's GP?

Occasionally

Page 4

Q4 If you (or a member of your ICU team) make contact with a patient's GP, what is the purpose of this communication?

To find out details leading to the patient's current illness	**Often**
To find out about patient's background medical and/or social history	**Never**
To find out about patient's regular medications and/or allergies	**Often**
To inform the GP that their patient has been admitted to ICU	**Never**
To inform the GP about details of the patient's ICU stay (eg. diagnosis, length of stay and/or illness severity)	**Sometimes**

Page 5

Q5 When managing patients in your ICU, how often would you (or a member of your ICU team) contact the GP either during or after ICU admission to give them details about their patient's ICU stay (eg. diagnosis, length of stay, illness severity)?

Occasionally

Page 6

Q6 When managing end-of-life care in your ICU, how often would you (or a member of your ICU team) contact the GP to tell them that their patient had died?

Occasionally

Page 7

Q7 When/if you make contact with a patient's GP, which method(s) of communication do you use (more than one if applicable)?

Phone call

Page 8

Q8 What do you think are the main factors discouraging you from contacting a GP when their patient is in your ICU?

Busy dealing with shared care teams and discussions with family of patient

Page 9

Q9 Please comment on the following statement: "Effective communication between ICU doctors and GPs is likely to benefit ICU patients after their hospital discharge"

Agree

Page 10

Q10 Please comment on the following statement: "Effective communication between ICU doctors and GPs is likely to benefit the relatives of ICU patients after their hospital discharge"

Agree

Page 11

Q11 Do you participate in any outpatient clinic following up patients who have had a previous ICU stay with critical illness

No

#42

COMPLETE

Collector: Web Link 3 (Web Link)
Started: Monday, June 20, 2016 5:49:30 PM
Last Modified: Monday, June 20, 2016 5:51:31 PM
Time Spent: 00:02:01
IP Address: 89.101.155.80

Page 1

Q1 Which of the following best describes your current professional position?

Consultant with <50% clinical duties in ICU

Page 2

Q2 In which type of hospital do you conduct most of your intensive care work?

University-affiliated hospital

Page 3

Q3 When managing patients in your ICU, how often would you (or a member of your ICU team) make direct contact, for any purpose, with the patient's GP?

Sometimes

Page 4

Q4 If you (or a member of your ICU team) make contact with a patient's GP, what is the purpose of this communication?

To find out details leading to the patient's current illness	**Often**
To find out about patient's background medical and/or social history	**Sometimes**
To find out about patient's regular medications and/or allergies	**Sometimes**
To inform the GP that their patient has been admitted to ICU	**Sometimes**
To inform the GP about details of the patient's ICU stay (eg. diagnosis, length of stay and/or illness severity)	**Often**

Page 5

Q5 When managing patients in your ICU, how often would you (or a member of your ICU team) contact the GP either during or after ICU admission to give them details about their patient's ICU stay (eg. diagnosis, length of stay, illness severity)?

Sometimes

Page 6

Q6 When managing end-of-life care in your ICU, how often would you (or a member of your ICU team) contact the GP to tell them that their patient had died?

Sometimes

Page 7

Q7 When/if you make contact with a patient's GP, which method(s) of communication do you use (more than one if applicable)?

Written letter

Page 8

Q8 What do you think are the main factors discouraging you from contacting a GP when their patient is in your ICU?

Time constraints

Page 9

Q9 Please comment on the following statement: "Effective communication between ICU doctors and GPs is likely to benefit ICU patients after their hospital discharge"

Strongly agree

Page 10

Q10 Please comment on the following statement: "Effective communication between ICU doctors and GPs is likely to benefit the relatives of ICU patients after their hospital discharge"

Strongly agree

Page 11

Q11 Do you participate in any outpatient clinic following up patients who have had a previous ICU stay with critical illness

Yes

#43

COMPLETE

Collector: Web Link 3 (Web Link)
Started: Monday, June 20, 2016 8:08:13 PM
Last Modified: Monday, June 20, 2016 8:15:11 PM
Time Spent: 00:06:58
IP Address: 95.44.202.12

Page 1

Q1 Which of the following best describes your current professional position?

Consultant with >/=50% clinical duties in ICU

Page 2

Q2 In which type of hospital do you conduct most of your intensive care work?

University-affiliated hospital

Page 3

Q3 When managing patients in your ICU, how often would you (or a member of your ICU team) make direct contact, for any purpose, with the patient's GP?

Sometimes

Page 4

Q4 If you (or a member of your ICU team) make contact with a patient's GP, what is the purpose of this communication?

To find out details leading to the patient's current illness	**Sometimes**
To find out about patient's background medical and/or social history	**Sometimes**
To find out about patient's regular medications and/or allergies	**Sometimes**
To inform the GP that their patient has been admitted to ICU	**Occasionally**
To inform the GP about details of the patient's ICU stay (eg. diagnosis, length of stay and/or illness severity)	**Never**

Page 5

Q5 When managing patients in your ICU, how often would you (or a member of your ICU team) contact the GP either during or after ICU admission to give them details about their patient's ICU stay (eg. diagnosis, length of stay, illness severity)?

Never

Page 6

Q6 When managing end-of-life care in your ICU, how often would you (or a member of your ICU team) contact the GP to tell them that their patient had died?

Occasionally

Page 7

Q7 When/if you make contact with a patient's GP, which method(s) of communication do you use (more than one if applicable)?

Phone call

Page 8

Q8 What do you think are the main factors discouraging you from contacting a GP when their patient is in your ICU?

It can be difficult to speak directly to a patients GP, there are office hours restrictions and other factors which make it difficult. Being honest I believe it is more the duty of the admitting team to address this aspect of the patients care.

Page 9

Q9 Please comment on the following statement: "Effective communication between ICU doctors and GPs is likely to benefit ICU patients after their hospital discharge"

Strongly agree

Page 10

Q10 Please comment on the following statement: "Effective communication between ICU doctors and GPs is likely to benefit the relatives of ICU patients after their hospital discharge"

Agree,

Please explain your answer:

There can by sequelae in terms of prolonged weakness, tracheostomy issues, HO or other ongoing difficulties that may be a significant factor in a patients longer term recovery. Therefore it is important the GP does get a very detailed handover from the medical staff within the hospital, this may not necessarily be the ICU team though. The admitting medical or surgical team do OPD follow up and are better placed to address the issues described.

Page 11

Q11 Do you participate in any outpatient clinic following up patients who have had a previous ICU stay with critical illness

No

#44

COMPLETE

Collector: Web Link 3 (Web Link)
Started: Tuesday, June 21, 2016 9:50:58 AM
Last Modified: Tuesday, June 21, 2016 9:55:15 AM
Time Spent: 00:04:17
IP Address: 95.83.254.232

Page 1

Q1 Which of the following best describes your current professional position?

Consultant with <50% clinical duties in ICU

Page 2

Q2 In which type of hospital do you conduct most of your intensive care work?

University-affiliated hospital

Page 3

Q3 When managing patients in your ICU, how often would you (or a member of your ICU team) make direct contact, for any purpose, with the patient's GP?

Occasionally

Page 4

Q4 If you (or a member of your ICU team) make contact with a patient's GP, what is the purpose of this communication?

To find out details leading to the patient's current illness	**Sometimes**
To find out about patient's background medical and/or social history	**Often**
To find out about patient's regular medications and/or allergies	**Occasionally**
To inform the GP that their patient has been admitted to ICU	**Never**
To inform the GP about details of the patient's ICU stay (eg. diagnosis, length of stay and/or illness severity)	**Never**

Page 5

Q5 When managing patients in your ICU, how often would you (or a member of your ICU team) contact the GP either during or after ICU admission to give them details about their patient's ICU stay (eg. diagnosis, length of stay, illness severity)?

Never

Page 6

Q6 When managing end-of-life care in your ICU, how often would you (or a member of your ICU team) contact the GP to tell them that their patient had died?

Occasionally

Page 7

Q7 When/if you make contact with a patient's GP, which method(s) of communication do you use (more than one if applicable)?

Phone call

Page 8

Q8 What do you think are the main factors discouraging you from contacting a GP when their patient is in your ICU?

Not discouraged if i think it will be helpful in current pt management

Page 9

Q9 Please comment on the following statement: "Effective communication between ICU doctors and GPs is likely to benefit ICU patients after their hospital discharge"

Neutral

Page 10

Q10 Please comment on the following statement: "Effective communication between ICU doctors and GPs is likely to benefit the relatives of ICU patients after their hospital discharge"

Neutral

Page 11

Q11 Do you participate in any outpatient clinic following up patients who have had a previous ICU stay with critical illness

No

#45

COMPLETE

Collector: Web Link 3 (Web Link)
Started: Tuesday, June 21, 2016 9:48:15 AM
Last Modified: Tuesday, June 21, 2016 9:56:19 AM
Time Spent: 00:08:04
IP Address: 213.233.132.177

Page 1

Q1 Which of the following best describes your current professional position?

Consultant with <50% clinical duties in ICU

Page 2

Q2 In which type of hospital do you conduct most of your intensive care work?

University-affiliated hospital

Page 3

Q3 When managing patients in your ICU, how often would you (or a member of your ICU team) make direct contact, for any purpose, with the patient's GP?

Occasionally

Page 4

Q4 If you (or a member of your ICU team) make contact with a patient's GP, what is the purpose of this communication?

To find out details leading to the patient's current illness	**Sometimes**
To find out about patient's background medical and/or social history	**Often**
To find out about patient's regular medications and/or allergies	**Often**
To inform the GP that their patient has been admitted to ICU	**Never**
To inform the GP about details of the patient's ICU stay (eg. diagnosis, length of stay and/or illness severity)	**Never**

Page 5

Q5 When managing patients in your ICU, how often would you (or a member of your ICU team) contact the GP either during or after ICU admission to give them details about their patient's ICU stay (eg. diagnosis, length of stay, illness severity)?

Never

Page 6

Q6 When managing end-of-life care in your ICU, how often would you (or a member of your ICU team) contact the GP to tell them that their patient had died?

Occasionally

Page 7

Q7 When/if you make contact with a patient's GP, which method(s) of communication do you use (more than one if applicable)?

Phone call

Page 8

Q8 What do you think are the main factors discouraging you from contacting a GP when their patient is in your ICU?

Hard to contact and usually adds little to existing knowledge.

Page 9

Q9 Please comment on the following statement: "Effective communication between ICU doctors and GPs is likely to benefit ICU patients after their hospital discharge"

Agree,
Please explain your answer:
GP understanding the severity of illness and prognosis ought to help.

Page 10

Q10 Please comment on the following statement: "Effective communication between ICU doctors and GPs is likely to benefit the relatives of ICU patients after their hospital discharge"

Agree,
Please explain your answer:
Relatives often really struggle with an incapacitated relative. Planning for and coping with this will need GP input.

Page 11

Q11 Do you participate in any outpatient clinic following up patients who have had a previous ICU stay with critical illness

No

#46

COMPLETE

Collector: Web Link 3 (Web Link)
Started: Tuesday, June 21, 2016 10:39:20 AM
Last Modified: Tuesday, June 21, 2016 10:41:59 AM
Time Spent: 00:02:39
IP Address: 80.111.90.68

Page 1

Q1 Which of the following best describes your current professional position?

Consultant with >/=50% clinical duties in ICU

Page 2

Q2 In which type of hospital do you conduct most of your intensive care work?

University-affiliated hospital

Page 3

Q3 When managing patients in your ICU, how often would you (or a member of your ICU team) make direct contact, for any purpose, with the patient's GP?

Sometimes

Page 4

Q4 If you (or a member of your ICU team) make contact with a patient's GP, what is the purpose of this communication?

To find out details leading to the patient's current illness	**Occasionally**
To find out about patient's background medical and/or social history	**Occasionally**
To find out about patient's regular medications and/or allergies	**Never**
To inform the GP that their patient has been admitted to ICU	**Often**
To inform the GP about details of the patient's ICU stay (eg. diagnosis, length of stay and/or illness severity)	**Often**

Page 5

Q5 When managing patients in your ICU, how often would you (or a member of your ICU team) contact the GP either during or after ICU admission to give them details about their patient's ICU stay (eg. diagnosis, length of stay, illness severity)?

Sometimes

Page 6

Q6 When managing end-of-life care in your ICU, how often would you (or a member of your ICU team) contact the GP to tell them that their patient had died?

Often

Page 7

Q7 When/if you make contact with a patient's GP, which method(s) of communication do you use (more than one if applicable)?

Phone call

Page 8

Q8 What do you think are the main factors discouraging you from contacting a GP when their patient is in your ICU?

Time

Page 9

Q9 Please comment on the following statement: "Effective communication between ICU doctors and GPs is likely to benefit ICU patients after their hospital discharge"

Neutral

Page 10

Q10 Please comment on the following statement: "Effective communication between ICU doctors and GPs is likely to benefit the relatives of ICU patients after their hospital discharge"

Neutral

Page 11

Q11 Do you participate in any outpatient clinic following up patients who have had a previous ICU stay with critical illness

Yes

#47

COMPLETE

Collector: Web Link 3 (Web Link)
Started: Tuesday, June 21, 2016 11:18:42 AM
Last Modified: Tuesday, June 21, 2016 11:20:06 AM
Time Spent: 00:01:24
IP Address: 95.83.254.63

Page 1

Q1 Which of the following best describes your current professional position?

Consultant with <50% clinical duties in ICU

Page 2

Q2 In which type of hospital do you conduct most of your intensive care work?

University-affiliated hospital

Page 3

Q3 When managing patients in your ICU, how often would you (or a member of your ICU team) make direct contact, for any purpose, with the patient's GP?

Never

Page 4

Q4 If you (or a member of your ICU team) make contact with a patient's GP, what is the purpose of this communication?

Respondent skipped this question

Page 5

Q5 When managing patients in your ICU, how often would you (or a member of your ICU team) contact the GP either during or after ICU admission to give them details about their patient's ICU stay (eg. diagnosis, length of stay, illness severity)?

Respondent skipped this question

Page 6

Q6 When managing end-of-life care in your ICU, how often would you (or a member of your ICU team) contact the GP to tell them that their patient had died?

Respondent skipped this question

Page 7

Q7 When/if you make contact with a patient's GP, which method(s) of communication do you use (more than one if applicable)?

Respondent skipped this question

Page 8

Q8 What do you think are the main factors discouraging you from contacting a GP when their patient is in your ICU?

Time

Page 9

Q9 Please comment on the following statement: "Effective communication between ICU doctors and GPs is likely to benefit ICU patients after their hospital discharge"

Agree

Page 10

Q10 Please comment on the following statement: "Effective communication between ICU doctors and GPs is likely to benefit the relatives of ICU patients after their hospital discharge"

Agree

Page 11

Q11 Do you participate in any outpatient clinic following up patients who have had a previous ICU stay with critical illness

No

#48

COMPLETE

Collector: Web Link 3 (Web Link)
Started: Tuesday, June 21, 2016 12:07:27 PM
Last Modified: Tuesday, June 21, 2016 12:10:32 PM
Time Spent: 00:03:05
IP Address: 137.191.232.40

Page 1

Q1 Which of the following best describes your current professional position?

Consultant with >/=50% clinical duties in ICU

Page 2

Q2 In which type of hospital do you conduct most of your intensive care work?

University-affiliated hospital

Page 3

Q3 When managing patients in your ICU, how often would you (or a member of your ICU team) make direct contact, for any purpose, with the patient's GP?

Sometimes

Page 4

Q4 If you (or a member of your ICU team) make contact with a patient's GP, what is the purpose of this communication?

To find out details leading to the patient's current illness	**Never**
To find out about patient's background medical and/or social history	**Occasionally**
To find out about patient's regular medications and/or allergies	**Sometimes**
To inform the GP that their patient has been admitted to ICU	**Sometimes**
To inform the GP about details of the patient's ICU stay (eg. diagnosis, length of stay and/or illness severity)	**Often**
Other (please specify)	**NICU so most patietns admitted form labour ward; always fax a letter to GP followed up with more detialed letter if a baby dies in NICU**

Page 5

**Q5** When managing patients in your ICU, how often would you (or a member of your ICU team) contact the GP either during or after ICU admission to give them details about their patient's ICU stay (eg. diagnosis, length of stay, illness severity)?	**Often**

Page 6

**Q6** When managing end-of-life care in your ICU, how often would you (or a member of your ICU team) contact the GP to tell them that their patient had died?	**Always**

Page 7

**Q7** When/if you make contact with a patient's GP, which method(s) of communication do you use (more than one if applicable)?	**Phone call,** **Written letter**

Page 8

Q8 What do you think are the main factors discouraging you from contacting a GP when their patient is in your ICU?

ease of access we have found faxing useful alternative to phoning

Page 9

**Q9** Please comment on the following statement: "Effective communication between ICU doctors and GPs is likely to benefit ICU patients after their hospital discharge"	**Agree**

Page 10

**Q10** Please comment on the following statement: "Effective communication between ICU doctors and GPs is likely to benefit the relatives of ICU patients after their hospital discharge"	**Agree**

Page 11

**Q11** Do you participate in any outpatient clinic following up patients who have had a previous ICU stay with critical illness	**Yes**

#49

COMPLETE

Collector: Web Link 3 (Web Link)
Started: Tuesday, June 21, 2016 12:27:41 PM
Last Modified: Tuesday, June 21, 2016 12:31:07 PM
Time Spent: 00:03:26
IP Address: 137.191.246.146

Page 1

Q1 Which of the following best describes your current professional position?

Consultant with <50% clinical duties in ICU

Page 2

Q2 In which type of hospital do you conduct most of your intensive care work?

University-affiliated hospital

Page 3

Q3 When managing patients in your ICU, how often would you (or a member of your ICU team) make direct contact, for any purpose, with the patient's GP?

Occasionally

Page 4

Q4 If you (or a member of your ICU team) make contact with a patient's GP, what is the purpose of this communication?

To find out details leading to the patient's current illness	**Sometimes**
To find out about patient's background medical and/or social history	**Often**
To find out about patient's regular medications and/or allergies	**Often**
To inform the GP that their patient has been admitted to ICU	**Never**
To inform the GP about details of the patient's ICU stay (eg. diagnosis, length of stay and/or illness severity)	**Never**

Page 5

Q5 When managing patients in your ICU, how often would you (or a member of your ICU team) contact the GP either during or after ICU admission to give them details about their patient's ICU stay (eg. diagnosis, length of stay, illness severity)?

Never

Page 6

Q6 When managing end-of-life care in your ICU, how often would you (or a member of your ICU team) contact the GP to tell them that their patient had died?

Never

Page 7

Q7 When/if you make contact with a patient's GP, which method(s) of communication do you use (more than one if applicable)?

Phone call

Page 8

Q8 What do you think are the main factors discouraging you from contacting a GP when their patient is in your ICU?

Primary team communicate with the GP

Page 9

Q9 Please comment on the following statement: "Effective communication between ICU doctors and GPs is likely to benefit ICU patients after their hospital discharge"

Strongly agree

Page 10

Q10 Please comment on the following statement: "Effective communication between ICU doctors and GPs is likely to benefit the relatives of ICU patients after their hospital discharge"

Strongly agree

Page 11

Q11 Do you participate in any outpatient clinic following up patients who have had a previous ICU stay with critical illness

No

#50

COMPLETE

Collector: Web Link 3 (Web Link)
Started: Tuesday, June 21, 2016 10:46:47 AM
Last Modified: Tuesday, June 21, 2016 1:09:33 PM
Time Spent: 02:22:46
IP Address: 149.5.32.5

Page 1

Q1 Which of the following best describes your current professional position?

Consultant with >/=50% clinical duties in ICU

Page 2

Q2 In which type of hospital do you conduct most of your intensive care work?

University-affiliated hospital

Page 3

Q3 When managing patients in your ICU, how often would you (or a member of your ICU team) make direct contact, for any purpose, with the patient's GP?

Occasionally

Page 4

Q4 If you (or a member of your ICU team) make contact with a patient's GP, what is the purpose of this communication?

To find out details leading to the patient's current illness	**Occasionally**
To find out about patient's background medical and/or social history	**Never**
To find out about patient's regular medications and/or allergies	**Never**
To inform the GP that their patient has been admitted to ICU	**Never**
To inform the GP about details of the patient's ICU stay (eg. diagnosis, length of stay and/or illness severity)	**Never**

Page 5

Q5 When managing patients in your ICU, how often would you (or a member of your ICU team) contact the GP either during or after ICU admission to give them details about their patient's ICU stay (eg. diagnosis, length of stay, illness severity)?

Never

Page 6

Q6 When managing end-of-life care in your ICU, how often would you (or a member of your ICU team) contact the GP to tell them that their patient had died?

Never

Page 7

Q7 When/if you make contact with a patient's GP, which method(s) of communication do you use (more than one if applicable)?

Phone call

Page 8

Q8 What do you think are the main factors discouraging you from contacting a GP when their patient is in your ICU?

Majority of inofmrtion has already been gathered by primary medical / surgical team / pharmacy. All other liaison are conducted with he next of kin. Little additional relevant information gained by direct ICU liaison with GP

Page 9

Q9 Please comment on the following statement: "Effective communication between ICU doctors and GPs is likely to benefit ICU patients after their hospital discharge"

Disagree

Page 10

Q10 Please comment on the following statement: "Effective communication between ICU doctors and GPs is likely to benefit the relatives of ICU patients after their hospital discharge"

Strongly disagree

Page 11

Q11 Do you participate in any outpatient clinic following up patients who have had a previous ICU stay with critical illness

No

#51

COMPLETE

Collector: Web Link 3 (Web Link)
Started: Tuesday, June 21, 2016 5:01:56 PM
Last Modified: Tuesday, June 21, 2016 5:14:54 PM
Time Spent: 00:12:58
IP Address: 92.251.255.11

Page 1

Q1 Which of the following best describes your current professional position?

Consultant with <50% clinical duties in ICU

Page 2

Q2 In which type of hospital do you conduct most of your intensive care work?

University-affiliated hospital

Page 3

Q3 When managing patients in your ICU, how often would you (or a member of your ICU team) make direct contact, for any purpose, with the patient's GP?

Often

Page 4

Q4 If you (or a member of your ICU team) make contact with a patient's GP, what is the purpose of this communication?

To find out details leading to the patient's current illness	**Often**
To find out about patient's background medical and/or social history	**Always**
To find out about patient's regular medications and/or allergies	**Always**
To inform the GP that their patient has been admitted to ICU	**Never**
To inform the GP about details of the patient's ICU stay (eg. diagnosis, length of stay and/or illness severity)	**Never**

Page 5

Q5 When managing patients in your ICU, how often would you (or a member of your ICU team) contact the GP either during or after ICU admission to give them details about their patient's ICU stay (eg. diagnosis, length of stay, illness severity)?

Never

Page 6

Q6 When managing end-of-life care in your ICU, how often would you (or a member of your ICU team) contact the GP to tell them that their patient had died?

Never

Page 7

Q7 When/if you make contact with a patient's GP, which method(s) of communication do you use (more than one if applicable)?

Phone call

Page 8

Q8 What do you think are the main factors discouraging you from contacting a GP when their patient is in your ICU?

Difficulty in communication

Page 9

Q9 Please comment on the following statement: "Effective communication between ICU doctors and GPs is likely to benefit ICU patients after their hospital discharge"

Agree,
Please explain your answer:
These pt's have higher risks of morbidity and mortality after hospital discharge than pt's who have not required ICU care.

Page 10

Q10 Please comment on the following statement: "Effective communication between ICU doctors and GPs is likely to benefit the relatives of ICU patients after their hospital discharge"

Agree,
Please explain your answer:
As per answer for #9

Page 11

Q11 Do you participate in any outpatient clinic following up patients who have had a previous ICU stay with critical illness

No

#52

COMPLETE

Collector: Web Link 3 (Web Link)
Started: Tuesday, June 21, 2016 5:23:03 PM
Last Modified: Tuesday, June 21, 2016 5:27:31 PM
Time Spent: 00:04:28
IP Address: 137.191.238.101

Page 1

Q1 Which of the following best describes your current professional position?

Consultant with >/=50% clinical duties in ICU

Page 2

Q2 In which type of hospital do you conduct most of your intensive care work?

University-affiliated hospital

Page 3

Q3 When managing patients in your ICU, how often would you (or a member of your ICU team) make direct contact, for any purpose, with the patient's GP?

Occasionally

Page 4

Q4 If you (or a member of your ICU team) make contact with a patient's GP, what is the purpose of this communication?

To find out details leading to the patient's current illness	**Occasionally**
To find out about patient's background medical and/or social history	**Occasionally**
To find out about patient's regular medications and/or allergies	**Occasionally**
To inform the GP that their patient has been admitted to ICU	**Never**
To inform the GP about details of the patient's ICU stay (eg. diagnosis, length of stay and/or illness severity)	**Never**

Page 5

Q5 When managing patients in your ICU, how often would you (or a member of your ICU team) contact the GP either during or after ICU admission to give them details about their patient's ICU stay (eg. diagnosis, length of stay, illness severity)?

Never

Page 6

Q6 When managing end-of-life care in your ICU, how often would you (or a member of your ICU team) contact the GP to tell them that their patient had died?

Occasionally

Page 7

Q7 When/if you make contact with a patient's GP, which method(s) of communication do you use (more than one if applicable)?

Phone call

Page 8

Q8 What do you think are the main factors discouraging you from contacting a GP when their patient is in your ICU?

Time constraints, lack of/misleading information re GP contact, and not clear that my call was useful to myself or GP

Page 9

Q9 Please comment on the following statement: "Effective communication between ICU doctors and GPs is likely to benefit ICU patients after their hospital discharge"

Strongly agree

Page 10

Q10 Please comment on the following statement: "Effective communication between ICU doctors and GPs is likely to benefit the relatives of ICU patients after their hospital discharge"

Strongly agree

Page 11

Q11 Do you participate in any outpatient clinic following up patients who have had a previous ICU stay with critical illness

No

#53

INCOMPLETE

Collector: Web Link 3 (Web Link)
Started: Tuesday, June 21, 2016 5:45:29 PM
Last Modified: Tuesday, June 21, 2016 5:48:11 PM
Time Spent: 00:02:42
IP Address: 109.78.49.225

Page 1

Q1 Which of the following best describes your current professional position?

Consultant with <50% clinical duties in ICU

Page 2

Q2 In which type of hospital do you conduct most of your intensive care work?

University-affiliated hospital

Page 3

Q3 When managing patients in your ICU, how often would you (or a member of your ICU team) make direct contact, for any purpose, with the patient's GP?

Occasionally

Page 4

Q4 If you (or a member of your ICU team) make contact with a patient's GP, what is the purpose of this communication?

To find out details leading to the patient's current illness	**Occasionally**
To find out about patient's background medical and/or social history	**Occasionally**
To find out about patient's regular medications and/or allergies	**Occasionally**
To inform the GP that their patient has been admitted to ICU	**Never**
To inform the GP about details of the patient's ICU stay (eg. diagnosis, length of stay and/or illness severity)	**Never**

Page 5

Q5 When managing patients in your ICU, how often would you (or a member of your ICU team) contact the GP either during or after ICU admission to give them details about their patient's ICU stay (eg. diagnosis, length of stay, illness severity)?

Never

Page 6

Q6 When managing end-of-life care in your ICU, how often would you (or a member of your ICU team) contact the GP to tell them that their patient had died?

Never

Page 7

Q7 When/if you make contact with a patient's GP, which method(s) of communication do you use (more than one if applicable)?

Phone call

Page 8

Q8 What do you think are the main factors discouraging you from contacting a GP when their patient is in your ICU?

no particular reason to other than to determine medications and pharmacy usually do this.

Page 9

Q9 Please comment on the following statement: "Effective communication between ICU doctors and GPs is likely to benefit ICU patients after their hospital discharge"

Strongly agree,

Please explain your answer:

In Australia we always sent discharge summaries to the GPs but In Australia we had adequate staffing levels and a full time secretary.

Page 10

Q10 Please comment on the following statement: "Effective communication between ICU doctors and GPs is likely to benefit the relatives of ICU patients after their hospital discharge"

Agree

Page 11

Q11 Do you participate in any outpatient clinic following up patients who have had a previous ICU stay with critical illness

No

#54

COMPLETE

Collector: Web Link 3 (Web Link)
Started: Tuesday, June 21, 2016 7:29:54 PM
Last Modified: Tuesday, June 21, 2016 7:31:10 PM
Time Spent: 00:01:16
IP Address: 37.228.232.251

Page 1

Q1 Which of the following best describes your current professional position?

Consultant with <50% clinical duties in ICU

Page 2

Q2 In which type of hospital do you conduct most of your intensive care work?

University-affiliated hospital

Page 3

Q3 When managing patients in your ICU, how often would you (or a member of your ICU team) make direct contact, for any purpose, with the patient's GP?

Never

Page 4

Q4 If you (or a member of your ICU team) make contact with a patient's GP, what is the purpose of this communication?

Respondent skipped this question

Page 5

Q5 When managing patients in your ICU, how often would you (or a member of your ICU team) contact the GP either during or after ICU admission to give them details about their patient's ICU stay (eg. diagnosis, length of stay, illness severity)?

Respondent skipped this question

Page 6

Q6 When managing end-of-life care in your ICU, how often would you (or a member of your ICU team) contact the GP to tell them that their patient had died?

Respondent skipped this question

Page 7

Q7 When/if you make contact with a patient's GP, which method(s) of communication do you use (more than one if applicable)?

Respondent skipped this question

Page 8

Q8 What do you think are the main factors discouraging you from contacting a GP when their patient is in your ICU?

Time, relevance

Page 9

Q9 Please comment on the following statement: "Effective communication between ICU doctors and GPs is likely to benefit ICU patients after their hospital discharge"

Neutral

Page 10

Q10 Please comment on the following statement: "Effective communication between ICU doctors and GPs is likely to benefit the relatives of ICU patients after their hospital discharge"

Neutral

Page 11

Q11 Do you participate in any outpatient clinic following up patients who have had a previous ICU stay with critical illness

Yes

#55

COMPLETE

Collector: Web Link 3 (Web Link)
Started: Tuesday, June 21, 2016 8:21:32 PM
Last Modified: Tuesday, June 21, 2016 8:26:42 PM
Time Spent: 00:05:10
IP Address: 79.97.69.200

Page 1

Q1 Which of the following best describes your current professional position?

Consultant with <50% clinical duties in ICU

Page 2

Q2 In which type of hospital do you conduct most of your intensive care work?

University-affiliated hospital

Page 3

Q3 When managing patients in your ICU, how often would you (or a member of your ICU team) make direct contact, for any purpose, with the patient's GP?

Sometimes

Page 4

Q4 If you (or a member of your ICU team) make contact with a patient's GP, what is the purpose of this communication?

To find out details leading to the patient's current illness	**Sometimes**
To find out about patient's background medical and/or social history	**Often**
To find out about patient's regular medications and/or allergies	**Often**
To inform the GP that their patient has been admitted to ICU	**Never**
To inform the GP about details of the patient's ICU stay (eg. diagnosis, length of stay and/or illness severity)	**Never**

Page 5

Q5 When managing patients in your ICU, how often would you (or a member of your ICU team) contact the GP either during or after ICU admission to give them details about their patient's ICU stay (eg. diagnosis, length of stay, illness severity)?

Never

Page 6

Q6 When managing end-of-life care in your ICU, how often would you (or a member of your ICU team) contact the GP to tell them that their patient had died?

Occasionally

Page 7

Q7 When/if you make contact with a patient's GP, which method(s) of communication do you use (more than one if applicable)?

Phone call

Page 8

Q8 What do you think are the main factors discouraging you from contacting a GP when their patient is in your ICU?

Time

Page 9

Q9 Please comment on the following statement: "Effective communication between ICU doctors and GPs is likely to benefit ICU patients after their hospital discharge"

Strongly agree,
Please explain your answer:
Patients may display symptoms of post ICU syndrome and awareness woud facilitate diagnosis. Patient may require ongoing community support for rehabilitation

Page 10

Q10 Please comment on the following statement: "Effective communication between ICU doctors and GPs is likely to benefit the relatives of ICU patients after their hospital discharge"

Strongly agree,
Please explain your answer:
Relatives often are traumatised by the ICU stay and awareness would facilitate diagnosis and support

Page 11

Q11 Do you participate in any outpatient clinic following up patients who have had a previous ICU stay with critical illness

No

#56

COMPLETE

Collector: Web Link 3 (Web Link)
Started: Friday, June 24, 2016 7:41:15 AM
Last Modified: Friday, June 24, 2016 7:45:22 AM
Time Spent: 00:04:07
IP Address: 176.12.107.139

Page 1

Q1 Which of the following best describes your current professional position?

Consultant with <50% clinical duties in ICU

Page 2

Q2 In which type of hospital do you conduct most of your intensive care work?

University-affiliated hospital

Page 3

Q3 When managing patients in your ICU, how often would you (or a member of your ICU team) make direct contact, for any purpose, with the patient's GP?

Sometimes

Page 4

Q4 If you (or a member of your ICU team) make contact with a patient's GP, what is the purpose of this communication?

To find out details leading to the patient's current illness	**Sometimes**
To find out about patient's background medical and/or social history	**Often**
To find out about patient's regular medications and/or allergies	**Sometimes**
To inform the GP that their patient has been admitted to ICU	**Occasionally**
To inform the GP about details of the patient's ICU stay (eg. diagnosis, length of stay and/or illness severity)	**Occasionally**

Page 5

Q5 When managing patients in your ICU, how often would you (or a member of your ICU team) contact the GP either during or after ICU admission to give them details about their patient's ICU stay (eg. diagnosis, length of stay, illness severity)?

Occasionally

Page 6

Q6 When managing end-of-life care in your ICU, how often would you (or a member of your ICU team) contact the GP to tell them that their patient had died?

Always

Page 7

Q7 When/if you make contact with a patient's GP, which method(s) of communication do you use (more than one if applicable)?

Phone call

Page 8

Q8 What do you think are the main factors discouraging you from contacting a GP when their patient is in your ICU?

They are hard to contact especially outside of office hours

Page 9

Q9 Please comment on the following statement: "Effective communication between ICU doctors and GPs is likely to benefit ICU patients after their hospital discharge"

Strongly agree

Page 10

Q10 Please comment on the following statement: "Effective communication between ICU doctors and GPs is likely to benefit the relatives of ICU patients after their hospital discharge"

Strongly agree

Page 11

Q11 Do you participate in any outpatient clinic following up patients who have had a previous ICU stay with critical illness

No

#57

COMPLETE

Collector: Web Link 3 (Web Link)
Started: Sunday, June 26, 2016 1:28:52 PM
Last Modified: Sunday, June 26, 2016 1:34:18 PM
Time Spent: 00:05:26
IP Address: 46.7.217.6

Page 1

Q1 Which of the following best describes your current professional position?

Consultant with <50% clinical duties in ICU

Page 2

Q2 In which type of hospital do you conduct most of your intensive care work?

University-affiliated hospital

Page 3

Q3 When managing patients in your ICU, how often would you (or a member of your ICU team) make direct contact, for any purpose, with the patient's GP?

Occasionally

Page 4

Q4 If you (or a member of your ICU team) make contact with a patient's GP, what is the purpose of this communication?

To find out details leading to the patient's current illness	**Often**
To find out about patient's background medical and/or social history	**Always**
To find out about patient's regular medications and/or allergies	**Often**
To inform the GP that their patient has been admitted to ICU	**Occasionally**
To inform the GP about details of the patient's ICU stay (eg. diagnosis, length of stay and/or illness severity)	**Occasionally**

Page 5

Q5 When managing patients in your ICU, how often would you (or a member of your ICU team) contact the GP either during or after ICU admission to give them details about their patient's ICU stay (eg. diagnosis, length of stay, illness severity)?

Occasionally

Page 6

Q6 When managing end-of-life care in your ICU, how often would you (or a member of your ICU team) contact the GP to tell them that their patient had died?

Never

Page 7

Q7 When/if you make contact with a patient's GP, which method(s) of communication do you use (more than one if applicable)?

Phone call,

Written letter

Page 8

Q8 What do you think are the main factors discouraging you from contacting a GP when their patient is in your ICU?

not part of routine practice unless information needed; difficulty speaking directly to GP;

Page 9

Q9 Please comment on the following statement: "Effective communication between ICU doctors and GPs is likely to benefit ICU patients after their hospital discharge"

Agree

Page 10

Q10 Please comment on the following statement: "Effective communication between ICU doctors and GPs is likely to benefit the relatives of ICU patients after their hospital discharge"

Agree,
Please explain your answer:
understanding of events which occured

Page 11

Q11 Do you participate in any outpatient clinic following up patients who have had a previous ICU stay with critical illness

No

#58

COMPLETE

Collector: Web Link 3 (Web Link)
Started: Monday, June 27, 2016 11:03:16 AM
Last Modified: Monday, June 27, 2016 11:14:06 AM
Time Spent: 00:10:50
IP Address: 88.87.177.190

Page 1

Q1 Which of the following best describes your current professional position?

Consultant with <50% clinical duties in ICU

Page 2

Q2 In which type of hospital do you conduct most of your intensive care work?

University-affiliated hospital

Page 3

Q3 When managing patients in your ICU, how often would you (or a member of your ICU team) make direct contact, for any purpose, with the patient's GP?

Occasionally

Page 4

Q4 If you (or a member of your ICU team) make contact with a patient's GP, what is the purpose of this communication?

To find out details leading to the patient's current illness	**Occasionally**
To find out about patient's background medical and/or social history	**Occasionally**
To find out about patient's regular medications and/or allergies	**Occasionally**
To inform the GP that their patient has been admitted to ICU	**Never**
To inform the GP about details of the patient's ICU stay (eg. diagnosis, length of stay and/or illness severity)	**Never**
Other (please specify)	**To inform the GP that a patient has died**

Page 5

Q5 When managing patients in your ICU, how often would you (or a member of your ICU team) contact the GP either during or after ICU admission to give them details about their patient's ICU stay (eg. diagnosis, length of stay, illness severity)?

Occasionally

Page 6

Q6 When managing end-of-life care in your ICU, how often would you (or a member of your ICU team) contact the GP to tell them that their patient had died?

Sometimes

Page 7

Q7 When/if you make contact with a patient's GP, which method(s) of communication do you use (more than one if applicable)?

Phone call

Page 8

Q8 What do you think are the main factors discouraging you from contacting a GP when their patient is in your ICU?

We need to ring switch to put us through - we don't have a list of GPs and their e-mails which would be helpful. Also it should be a tick box on our discharge form - we don't have a discharge letter yet. Once the electronic system is in things should improve.

Page 9

Q9 Please comment on the following statement: "Effective communication between ICU doctors and GPs is likely to benefit ICU patients after their hospital discharge"

Strongly agree

Page 10

Q10 Please comment on the following statement: "Effective communication between ICU doctors and GPs is likely to benefit the relatives of ICU patients after their hospital discharge"

Strongly agree

Page 11

Q11 Do you participate in any outpatient clinic following up patients who have had a previous ICU stay with critical illness

No

#59

INCOMPLETE

Collector: Web Link 3 (Web Link)
Started: Monday, June 27, 2016 1:54:56 PM
Last Modified: Monday, June 27, 2016 1:57:30 PM
Time Spent: 00:02:34
IP Address: 137.191.240.200

Page 1

Q1 Which of the following best describes your current professional position?

Consultant with <50% clinical duties in ICU

Page 2

Q2 In which type of hospital do you conduct most of your intensive care work?

University-affiliated hospital

Page 3

Q3 When managing patients in your ICU, how often would you (or a member of your ICU team) make direct contact, for any purpose, with the patient's GP?

Never

Page 4

Q4 If you (or a member of your ICU team) make contact with a patient's GP, what is the purpose of this communication?

To find out details leading to the patient's current illness	**Always**
To find out about patient's background medical and/or social history	**Never**
To find out about patient's regular medications and/or allergies	**Never**
To inform the GP that their patient has been admitted to ICU	**Never**
To inform the GP about details of the patient's ICU stay (eg. diagnosis, length of stay and/or illness severity)	**Never**

Page 5

Q5 When managing patients in your ICU, how often would you (or a member of your ICU team) contact the GP either during or after ICU admission to give them details about their patient's ICU stay (eg. diagnosis, length of stay, illness severity)?

Respondent skipped this question

Page 6

Q6 When managing end-of-life care in your ICU, how often would you (or a member of your ICU team) contact the GP to tell them that their patient had died?

Respondent skipped this question

Page 7

Q7 When/if you make contact with a patient's GP, which method(s) of communication do you use (more than one if applicable)?

Respondent skipped this question

Page 8

Q8 What do you think are the main factors discouraging you from contacting a GP when their patient is in your ICU?

irrelevance

Page 9

Q9 Please comment on the following statement: "Effective communication between ICU doctors and GPs is likely to benefit ICU patients after their hospital discharge"

Respondent skipped this question

Page 10

Q10 Please comment on the following statement: "Effective communication between ICU doctors and GPs is likely to benefit the relatives of ICU patients after their hospital discharge"

Respondent skipped this question

Page 11

Q11 Do you participate in any outpatient clinic following up patients who have had a previous ICU stay with critical illness

Respondent skipped this question

#60

COMPLETE

Collector: Web Link 3 (Web Link)
Started: Tuesday, June 28, 2016 7:11:23 AM
Last Modified: Tuesday, June 28, 2016 7:23:43 AM
Time Spent: 00:12:20
IP Address: 178.167.254.166

Page 1

Q1 Which of the following best describes your current professional position?

Consultant with >/=50% clinical duties in ICU

Page 2

Q2 In which type of hospital do you conduct most of your intensive care work?

University-affiliated hospital

Page 3

Q3 When managing patients in your ICU, how often would you (or a member of your ICU team) make direct contact, for any purpose, with the patient's GP?

Occasionally

Page 4

Q4 If you (or a member of your ICU team) make contact with a patient's GP, what is the purpose of this communication?

To find out details leading to the patient's current illness	**Occasionally**
To find out about patient's background medical and/or social history	**Occasionally**
To find out about patient's regular medications and/or allergies	**Occasionally**
To inform the GP that their patient has been admitted to ICU	**Never**
To inform the GP about details of the patient's ICU stay (eg. diagnosis, length of stay and/or illness severity)	**Never**

Page 5

Q5 When managing patients in your ICU, how often would you (or a member of your ICU team) contact the GP either during or after ICU admission to give them details about their patient's ICU stay (eg. diagnosis, length of stay, illness severity)?

Never

Page 6

Q6 When managing end-of-life care in your ICU, how often would you (or a member of your ICU team) contact the GP to tell them that their patient had died?

Never

Page 7

Q7 When/if you make contact with a patient's GP, which method(s) of communication do you use (more than one if applicable)?

Phone call

Page 8

Q8 What do you think are the main factors discouraging you from contacting a GP when their patient is in your ICU?

We have so little contact in general with GPs. And we gain information about the patient's background and clinical condition from the Out-Patient Clinics instead.

Page 9

Q9 Please comment on the following statement: "Effective communication between ICU doctors and GPs is likely to benefit ICU patients after their hospital discharge"

Agree,
Please explain your answer:
The GPs receive a discharge summary from the primary team after the patient being discharged from the hospital. Ideally it should contain a summary of the ICU stay as well. But I agree it would be beneficial to share information from multiple angles (primary team as well as ICU team).

Page 10

Q10 Please comment on the following statement: "Effective communication between ICU doctors and GPs is likely to benefit the relatives of ICU patients after their hospital discharge"

Agree

Page 11

Q11 Do you participate in any outpatient clinic following up patients who have had a previous ICU stay with critical illness

No

#61

COMPLETE

Collector: Web Link 3 (Web Link)
Started: Monday, July 11, 2016 10:01:25 PM
Last Modified: Monday, July 11, 2016 10:05:36 PM
Time Spent: 00:04:11
IP Address: 46.233.116.31

Page 1

Q1 Which of the following best describes your current professional position?

Consultant with <50% clinical duties in ICU

Page 2

Q2 In which type of hospital do you conduct most of your intensive care work?

Non university-affiliated hospital

Page 3

Q3 When managing patients in your ICU, how often would you (or a member of your ICU team) make direct contact, for any purpose, with the patient's GP?

Sometimes

Page 4

Q4 If you (or a member of your ICU team) make contact with a patient's GP, what is the purpose of this communication?

To find out details leading to the patient's current illness	**Sometimes**
To find out about patient's background medical and/or social history	**Often**
To find out about patient's regular medications and/or allergies	**Always**
To inform the GP that their patient has been admitted to ICU	**Sometimes**
To inform the GP about details of the patient's ICU stay (eg. diagnosis, length of stay and/or illness severity)	**Occasionally**

Page 5

Q5 When managing patients in your ICU, how often would you (or a member of your ICU team) contact the GP either during or after ICU admission to give them details about their patient's ICU stay (eg. diagnosis, length of stay, illness severity)?

Sometimes

Page 6

Q6 When managing end-of-life care in your ICU, how often would you (or a member of your ICU team) contact the GP to tell them that their patient had died?

Occasionally

Page 7

Q7 When/if you make contact with a patient's GP, which method(s) of communication do you use (more than one if applicable)?

Phone call

Page 8

Q8 What do you think are the main factors discouraging you from contacting a GP when their patient is in your ICU?

time

Page 9

Q9 Please comment on the following statement: "Effective communication between ICU doctors and GPs is likely to benefit ICU patients after their hospital discharge"

Strongly agree

Page 10

Q10 Please comment on the following statement: "Effective communication between ICU doctors and GPs is likely to benefit the relatives of ICU patients after their hospital discharge"

Strongly agree

Page 11

Q11 Do you participate in any outpatient clinic following up patients who have had a previous ICU stay with critical illness

No

#62

COMPLETE

Collector: Web Link 3 (Web Link)
Started: Monday, July 11, 2016 10:28:23 PM
Last Modified: Monday, July 11, 2016 10:34:29 PM
Time Spent: 00:06:06
IP Address: 78.17.14.236

Page 1

Q1 Which of the following best describes your current professional position?

Consultant with <50% clinical duties in ICU

Page 2

Q2 In which type of hospital do you conduct most of your intensive care work?

University-affiliated hospital

Page 3

Q3 When managing patients in your ICU, how often would you (or a member of your ICU team) make direct contact, for any purpose, with the patient's GP?

Occasionally

Page 4

Q4 If you (or a member of your ICU team) make contact with a patient's GP, what is the purpose of this communication?

To find out details leading to the patient's current illness	**Occasionally**
To find out about patient's background medical and/or social history	**Occasionally**
To find out about patient's regular medications and/or allergies	**Occasionally**
To inform the GP that their patient has been admitted to ICU	**Never**
To inform the GP about details of the patient's ICU stay (eg. diagnosis, length of stay and/or illness severity)	**Never**

Page 5

Q5 When managing patients in your ICU, how often would you (or a member of your ICU team) contact the GP either during or after ICU admission to give them details about their patient's ICU stay (eg. diagnosis, length of stay, illness severity)?

Never

Page 6

Q6 When managing end-of-life care in your ICU, how often would you (or a member of your ICU team) contact the GP to tell them that their patient had died?

Occasionally

Page 7

Q7 When/if you make contact with a patient's GP, which method(s) of communication do you use (more than one if applicable)?

Phone call

Page 8

Q8 What do you think are the main factors discouraging you from contacting a GP when their patient is in your ICU?

Information usually available from old notes or admitting physician/surgeon

Page 9

Q9 Please comment on the following statement: "Effective communication between ICU doctors and GPs is likely to benefit ICU patients after their hospital discharge"

Agree,
Please explain your answer:
Admitting team involved in ICU care in our unit and would normally be the ones arranging discharge from hospital and follow up, not usually us as the ICU team

Page 10

Q10 Please comment on the following statement: "Effective communication between ICU doctors and GPs is likely to benefit the relatives of ICU patients after their hospital discharge"

Agree,
Please explain your answer:
More likely to have contact with families than GP during ICU stay and prognosis and likely course of recovery often discussed with relatives

Page 11

Q11 Do you participate in any outpatient clinic following up patients who have had a previous ICU stay with critical illness

No

#63

COMPLETE

Collector: Web Link 3 (Web Link)
Started: Tuesday, July 12, 2016 4:11:41 AM
Last Modified: Tuesday, July 12, 2016 4:19:19 AM
Time Spent: 00:07:38
IP Address: 188.141.18.194

Page 1

Q1 Which of the following best describes your current professional position?

Consultant with <50% clinical duties in ICU

Page 2

Q2 In which type of hospital do you conduct most of your intensive care work?

Non university-affiliated hospital

Page 3

Q3 When managing patients in your ICU, how often would you (or a member of your ICU team) make direct contact, for any purpose, with the patient's GP?

Sometimes

Page 4

Q4 If you (or a member of your ICU team) make contact with a patient's GP, what is the purpose of this communication?

To find out details leading to the patient's current illness	**Occasionally**
To find out about patient's background medical and/or social history	**Often**
To find out about patient's regular medications and/or allergies	**Always**
To inform the GP that their patient has been admitted to ICU	**Occasionally**
To inform the GP about details of the patient's ICU stay (eg. diagnosis, length of stay and/or illness severity)	**Sometimes**

Page 5

Q5 When managing patients in your ICU, how often would you (or a member of your ICU team) contact the GP either during or after ICU admission to give them details about their patient's ICU stay (eg. diagnosis, length of stay, illness severity)?

Occasionally

Page 6

Q6 When managing end-of-life care in your ICU, how often would you (or a member of your ICU team) contact the GP to tell them that their patient had died?

Occasionally

Page 7

Q7 When/if you make contact with a patient's GP, which method(s) of communication do you use (more than one if applicable)?

Phone call

Page 8

Q8 What do you think are the main factors discouraging you from contacting a GP when their patient is in your ICU?

Anaesthetist majority of the times is a secondary team member. Primary consultant under whom patient is admitted will contact the GP

Page 9

Q9 Please comment on the following statement: "Effective communication between ICU doctors and GPs is likely to benefit ICU patients after their hospital discharge"

Strongly agree

Page 10

Q10 Please comment on the following statement: "Effective communication between ICU doctors and GPs is likely to benefit the relatives of ICU patients after their hospital discharge"

Strongly agree

Page 11

Q11 Do you participate in any outpatient clinic following up patients who have had a previous ICU stay with critical illness

No

#64

COMPLETE

Collector: Web Link 3 (Web Link)
Started: Monday, July 18, 2016 2:45:44 PM
Last Modified: Monday, July 18, 2016 2:48:22 PM
Time Spent: 00:02:38
IP Address: 78.17.28.250

Page 1

Q1 Which of the following best describes your current professional position?

Consultant with >/=50% clinical duties in ICU

Page 2

Q2 In which type of hospital do you conduct most of your intensive care work?

University-affiliated hospital

Page 3

Q3 When managing patients in your ICU, how often would you (or a member of your ICU team) make direct contact, for any purpose, with the patient's GP?

Occasionally

Page 4

Q4 If you (or a member of your ICU team) make contact with a patient's GP, what is the purpose of this communication?

To find out details leading to the patient's current illness	**Occasionally**
To find out about patient's background medical and/or social history	**Often**
To find out about patient's regular medications and/or allergies	**Often**
To inform the GP that their patient has been admitted to ICU	**Never**
To inform the GP about details of the patient's ICU stay (eg. diagnosis, length of stay and/or illness severity)	**Never**

Page 5

Q5 When managing patients in your ICU, how often would you (or a member of your ICU team) contact the GP either during or after ICU admission to give them details about their patient's ICU stay (eg. diagnosis, length of stay, illness severity)?

Never

Page 6

Q6 When managing end-of-life care in your ICU, how often would you (or a member of your ICU team) contact the GP to tell them that their patient had died?

Never

Page 7

Q7 When/if you make contact with a patient's GP, which method(s) of communication do you use (more than one if applicable)?

Phone call

Page 8

Q8 What do you think are the main factors discouraging you from contacting a GP when their patient is in your ICU?

Lack of perceived need

Page 9

Q9 Please comment on the following statement: "Effective communication between ICU doctors and GPs is likely to benefit ICU patients after their hospital discharge"

Neutral

Page 10

Q10 Please comment on the following statement: "Effective communication between ICU doctors and GPs is likely to benefit the relatives of ICU patients after their hospital discharge"

Agree

Page 11

Q11 Do you participate in any outpatient clinic following up patients who have had a previous ICU stay with critical illness

No

#65

COMPLETE

Collector: Web Link 2 (Web Link)
Started: Wednesday, February 01, 2017 8:17:08 AM
Last Modified: Wednesday, February 01, 2017 8:37:19 AM
Time Spent: 00:20:11
IP Address: 78.19.175.133

Page 1

Q1 Which of the following best describes your current professional position?

Consultant with <50% clinical duties in ICU

Page 2

Q2 In which type of hospital do you conduct most of your intensive care work?

University-affiliated hospital

Page 3

Q3 When managing patients in your ICU, how often would you (or a member of your ICU team) make direct contact, for any purpose, with the patient's GP?

Occasionally

Page 4

Q4 If you (or a member of your ICU team) make contact with a patient's GP, what is the purpose of this communication?

To find out details leading to the patient's current illness	**Occasionally**
To find out about patient's background medical and/or social history	**Occasionally**
To find out about patient's regular medications and/or allergies	**Occasionally**
To inform the GP that their patient has been admitted to ICU	**Never**
To inform the GP about details of the patient's ICU stay (eg. diagnosis, length of stay and/or illness severity)	**Never**

Page 5

Q5 When managing patients in your ICU, how often would you (or a member of your ICU team) contact the GP either during or after ICU admission to give them details about their patient's ICU stay (eg. diagnosis, length of stay, illness severity)?

Never

Page 6

Q6 When managing end-of-life care in your ICU, how often would you (or a member of your ICU team) contact the GP to tell them that their patient had died?

Never

Page 7

Q7 When/if you make contact with a patient's GP, which method(s) of communication do you use (more than one if applicable)?

I do not make contact with the GP

Page 8

Q8 What do you think are the main factors discouraging you from contacting a GP when their patient is in your ICU?

no time!!

Page 9

Q9 Please comment on the following statement: "Effective communication between ICU doctors and GPs is likely to benefit ICU patients after their hospital discharge"

Strongly agree

Page 10

Q10 Please comment on the following statement: "Effective communication between ICU doctors and GPs is likely to benefit the relatives of ICU patients after their hospital discharge"

Strongly agree

Page 11

Q11 Do you participate in any outpatient clinic following up patients who have had a previous ICU stay with critical illness

No
