# Supplementary material for: Information sharing between intensive care and primary care after an episode of critical illness; A mixed methods analysis
Source: PLoS One. 2019 Feb 28;14(2):e0212438. doi: 10.1371/journal.pone.0212438 (PMC6394993; doi:10.1371/journal.pone.0212438)
Supplement: S2 Dataset — (PDF) [file pone.0212438.s004.pdf]

#1

COMPLETE

Collector: Web Link 1 (Web Link)
Started: Monday, June 20, 2016 7:11:21 PM
Last Modified: Monday, June 20, 2016 7:16:43 PM
Time Spent: 00:05:22
IP Address: 83.71.22.8

Page 1

Q1 Which of the following best describes your GP practice?

Rural practice (outside city)

Page 2

Q2 Please indicate how long you have been working as a general practitioner.

>20years

Page 3

Q3 In which county do you conduct most of your GP work?

Limerick

Page 4

Q4 Please comment on the following statements regarding communication you receive after your patients are discharged from hospital.

I receive details of the patient’s admission	**Always**
I receive details of their admission within 30days of patient’s discharge	**Always**
The details I receive include whether patient was admitted to the ICU or not	**Rarely**

Page 5

Q5 If you receive information about your patient's ICU stay, by which method(s) would you receive this information?

The ICU/anaesthetic staff phone me directly	**Never**
In a discharge summary or letter from ICU medical team	**Never**
In a discharge summary or letter from other non-ICU medical/surgical team	**Rarely**
I contact the hospital myself to find out	**Rarely**
The patient's relatives tell me	**Often**
The patient tells me after hospital discharge	**Often**

Page 6

Q6 If you received information about your patient's stay in ICU, how often would it include details about the following aspects of their critical illness?

Shock	**Rarely**
Respiratory failure and mechanical ventilation	**Occasionally**
Acute kidney injury requiring acute dialysis	**Occasionally**
Acute encephalopathy / Delirium	**Rarely**
ARDS (acute respiratory distress syndrome)	**Never**
Neuromuscular weakness	**Never**
Tracheostomy insertion	**Occasionally**
The duration of patient's stay in ICU	**Often**

Page 7

**Q7** If, during a recent hospital stay, your patient was in ICU, would you record this ICU admission in the medical/surgical history section of their notes?	**No**

Page 8

Q8 You receive a discharge summary in the post about your patient who was recently discharged from hospital after a severe illness. The summary confirms that they were in ICU during the hospital stay. Please comment on the following statements about the patient’s follow-up care:

Because of the patient’s ICU admission, I would make contact with them, even if the discharge summary did not request specific follow-up	**Strongly disagree**
If the patient did not self-present to my surgery for follow-up, the ICU admission would prompt me to schedule a consultation with them	**Disagree**
If the patient did not self-present to my surgery for follow-up, the ICU admission would prompt me to schedule a consultation with the patient and a close relative	**Disagree**

Page 9

Q9 Have you ever attended an educational meeting at which you learned about the long term complications of critical illness?

No,
If Yes, what was the event? If No, would you attend one if it was available?:
Probably not- we already have too many meetings and too litle time- that is also the reason why I would not schedule a follow up- we do not have the time

Page 10

Q10 Are you aware of any published Guidelines about the rehabilitation of patients following hospital discharge after ICU admission with critical illness?

No

Page 11

Q11 Do you think it would benefit your patient and/or their family members if you received details about their ICU admission?

Yes,
If Yes, what the possible benefits?:
More awareness about possible complications later especially from a psychological perspective

#2

COMPLETE

Collector: Web Link 1 (Web Link)
Started: Monday, June 20, 2016 8:13:27 PM
Last Modified: Monday, June 20, 2016 8:16:52 PM
Time Spent: 00:03:25
IP Address: 109.79.79.18

Page 1

Q1 Which of the following best describes your GP practice?

Rural practice (outside city)

Page 2

Q2 Please indicate how long you have been working as a general practitioner.

>20years

Page 3

Q3 In which county do you conduct most of your GP work?

Kildare

Page 4

Q4 Please comment on the following statements regarding communication you receive after your patients are discharged from hospital.

I receive details of the patient's admission **Often**

I receive details of their admission within 30days of patient's discharge **Often**

The details I receive include whether patient was admitted to the ICU or not **Occasionally**

Page 5

Q5 If you receive information about your patient's ICU stay, by which method(s) would you receive this information?

The ICU/anaesthetic staff phone me directly	**Never**
In a discharge summary or letter from ICU medical team	**Never**
I contact the hospital myself to find out	**Rarely**
The patient's relatives tell me	**Often**
The patient tells me after hospital discharge	**Often**

Page 6

Q6 If you received information about your patient's stay in ICU, how often would it include details about the following aspects of their critical illness?

Shock	**Never**
Respiratory failure and mechanical ventilation	**Never**
Acute kidney injury requiring acute dialysis	**Often**
Acute encephalopathy / Delirium	**Rarely**
ARDS (acute respiratory distress syndrome)	**Often**
Neuromuscular weakness	**Never**
Tracheostomy insertion	**Rarely**
The duration of patient's stay in ICU	**Rarely**

Page 7

Q7 If, during a recent hospital stay, your patient was in ICU, would you record this ICU admission in the medical/surgical history section of their notes? **No**

Page 8

Q8 You receive a discharge summary in the post about your patient who was recently discharged from hospital after a severe illness. The summary confirms that they were in ICU during the hospital stay. Please comment on the following statements about the patient's follow-up care:

Because of the patient's ICU admission, I would make contact with them, even if the discharge summary did not request specific follow-up	**Neutral**
If the patient did not self-present to my surgery for follow-up, the ICU admission would prompt me to schedule a consultation with them	**Agree**
If the patient did not self-present to my surgery for follow-up, the ICU admission would prompt me to schedule a consultation with the patient and a close relative	**Disagree**

Page 9

Q9 Have you ever attended an educational meeting at which you learned about the long term complications of critical illness?

No

Page 10

Q10 Are you aware of any published Guidelines about the rehabilitation of patients following hospital discharge after ICU admission with critical illness?

No

Page 11

Q11 Do you think it would benefit your patient and/or their family members if you received details about their ICU admission?

Yes,
If Yes, what the possible benefits?:
Timely notification would enable discharge reviews at the practice, which in turn would enable more effective support and tailored follow up

#3

COMPLETE

**Collector:**	Web Link 1 (Web Link)
**Started:**	Monday, June 20, 2016 8:36:55 PM
**Last Modified:**	Monday, June 20, 2016 8:43:58 PM
**Time Spent:**	00:07:03
**IP Address:**	37.203.199.5

Page 1

Q1 Which of the following best describes your GP practice?

Rural practice (outside city)

Page 2

Q2 Please indicate how long you have been working as a general practitioner.

>20years

Page 3

Q3 In which county do you conduct most of your GP work?

Tipperary South

Page 4

Q4 Please comment on the following statements regarding communication you receive after your patients are discharged from hospital.

I receive details of the patient's admission	**Often**
I receive details of their admission within 30days of patient's discharge	**Often**
The details I receive include whether patient was admitted to the ICU or not	**Rarely**

Page 5

Q5 If you receive information about your patient’s ICU stay, by which method(s) would you receive this information?

The ICU/anaesthetic staff phone me directly	**Never**
In a discharge summary or letter from ICU medical team	**Never**
In a discharge summary or letter from other non-ICU medical/surgical team	**Occasionally**
I contact the hospital myself to find out	**Occasionally**
The patient’s relatives tell me	**Often**
The patient tells me after hospital discharge	**Always**

Page 6

Q6 If you received information about your patient’s stay in ICU, how often would it include details about the following aspects of their critical illness?

Shock	**Never**
Respiratory failure and mechanical ventilation	**Rarely**
Acute kidney injury requiring acute dialysis	**Rarely**
Acute encephalopathy / Delirium	**Rarely**
ARDS (acute respiratory distress syndrome)	**Rarely**
Neuromuscular weakness	**Never**
Tracheostomy insertion	**Never**
The duration of patient’s stay in ICU	**Never**

Page 7

**Q7** If, during a recent hospital stay, your patient was in ICU, would you record this ICU admission in the medical/surgical history section of their notes?	**Yes**

Page 8

Q8 You receive a discharge summary in the post about your patient who was recently discharged from hospital after a severe illness. The summary confirms that they were in ICU during the hospital stay. Please comment on the following statements about the patient's follow-up care:

Because of the patient's ICU admission, I would make contact with them, even if the discharge summary did not request specific follow-up	**Strongly agree**
If the patient did not self-present to my surgery for follow-up, the ICU admission would prompt me to schedule a consultation with them	**Strongly agree**
If the patient did not self-present to my surgery for follow-up, the ICU admission would prompt me to schedule a consultation with the patient and a close relative	**Neutral**

Page 9

Q9 Have you ever attended an educational meeting at which you learned about the long term complications of critical illness?

No,
If Yes, what was the event? If No, would you attend one if it was available?:
Yes

Page 10

Q10 Are you aware of any published Guidelines about the rehabilitation of patients following hospital discharge after ICU admission with critical illness?

Yes,
If Yes, what is the Guideline?:
NICE

Page 11

Q11 Do you think it would benefit your patient and/or their family members if you received details about their ICU admission?

Yes,
If Yes, what the possible benefits?:
Ability to hone in on particular rehab issues and to discuss course of critical illness with patient when they are well enough to comprehend it fully

#4

COMPLETE

Collector: Web Link 1 (Web Link)
Started: Monday, June 20, 2016 10:08:09 PM
Last Modified: Monday, June 20, 2016 10:11:03 PM
Time Spent: 00:02:54
IP Address: 78.18.21.228

Page 1

Q1 Which of the following best describes your GP practice?

Urban practice (Dublin, Cork, Galway, Limerick)

Page 2

Q2 Please indicate how long you have been working as a general practitioner.

>20years

Page 3

Q3 In which county do you conduct most of your GP work?

Dublin

Page 4

Q4 Please comment on the following statements regarding communication you receive after your patients are discharged from hospital.

I receive details of the patient’s admission	**Often**
I receive details of their admission within 30days of patient’s discharge	**Often**
The details I receive include whether patient was admitted to the ICU or not	**Often**

Page 5

Q5 If you receive information about your patient's ICU stay, by which method(s) would you receive this information?

The ICU/anaesthetic staff phone me directly	**Never**
In a discharge summary or letter from ICU medical team	**Never**
In a discharge summary or letter from other non-ICU medical/surgical team	**Often**
I contact the hospital myself to find out	**Occasionally**
The patient's relatives tell me	**Occasionally**
The patient tells me after hospital discharge	**Occasionally**

Page 6

Q6 If you received information about your patient's stay in ICU, how often would it include details about the following aspects of their critical illness?

Shock	**Rarely**
Respiratory failure and mechanical ventilation	**Rarely**
Acute kidney injury requiring acute dialysis	**Often**
Acute encephalopathy / Delirium	**Rarely**
ARDS (acute respiratory distress syndrome)	**Rarely**
Neuromuscular weakness	**Rarely**
Tracheostomy insertion	**Rarely**
The duration of patient's stay in ICU	**Occasionally**

Page 7

Q7 If, during a recent hospital stay, your patient was in ICU, would you record this ICU admission in the medical/surgical history section of their notes? **Yes**

Page 8

Q8 You receive a discharge summary in the post about your patient who was recently discharged from hospital after a severe illness. The summary confirms that they were in ICU during the hospital stay. Please comment on the following statements about the patient's follow-up care:

Because of the patient's ICU admission, I would make contact with them, even if the discharge summary did not request specific follow-up	**Neutral**
If the patient did not self-present to my surgery for follow-up, the ICU admission would prompt me to schedule a consultation with them	**Agree**
If the patient did not self-present to my surgery for follow-up, the ICU admission would prompt me to schedule a consultation with the patient and a close relative	**Disagree**

Page 9

Q9 Have you ever attended an educational meeting at which you learned about the long term complications of critical illness?

No

Page 10

Q10 Are you aware of any published Guidelines about the rehabilitation of patients following hospital discharge after ICU admission with critical illness?

No

Page 11

Q11 Do you think it would benefit your patient and/or their family members if you received details about their ICU admission?

Yes

#5

COMPLETE

Collector: Web Link 1 (Web Link)
Started: Tuesday, June 21, 2016 9:34:05 AM
Last Modified: Tuesday, June 21, 2016 9:36:47 AM
Time Spent: 00:02:42
IP Address: 82.141.235.1

Page 1

Q1 Which of the following best describes your GP practice?

Rural practice (outside city)

Page 2

Q2 Please indicate how long you have been working as a general practitioner.

>20years

Page 3

Q3 In which county do you conduct most of your GP work?

meath

Page 4

Q4 Please comment on the following statements regarding communication you receive after your patients are discharged from hospital.

I receive details of the patient's admission **Often**

I receive details of their admission within 30days of patient's discharge **Occasionally**

The details I receive include whether patient was admitted to the ICU or not **Rarely**

Page 5

Q5 If you receive information about your patient's ICU stay, by which method(s) would you receive this information?

The ICU/anaesthetic staff phone me directly	**Never**
In a discharge summary or letter from ICU medical team	**Never**
In a discharge summary or letter from other non-ICU medical/surgical team	**Never**
I contact the hospital myself to find out	**Occasionally**
The patient's relatives tell me	**Occasionally**
The patient tells me after hospital discharge	**Often**

Page 6

Q6 If you received information about your patient's stay in ICU, how often would it include details about the following aspects of their critical illness?

Shock	**Never**
Respiratory failure and mechanical ventilation	**Never**
Acute kidney injury requiring acute dialysis	**Never**
Acute encephalopathy / Delirium	**Never**
ARDS (acute respiratory distress syndrome)	**Never**
Neuromuscular weakness	**Never**
Tracheostomy insertion	**Never**
The duration of patient's stay in ICU	**Never**

Page 7

Q7 If, during a recent hospital stay, your patient was in ICU, would you record this ICU admission in the medical/surgical history section of their notes? **No**

Page 8

Q8 You receive a discharge summary in the post about your patient who was recently discharged from hospital after a severe illness. The summary confirms that they were in ICU during the hospital stay. Please comment on the following statements about the patient's follow-up care:

Because of the patient's ICU admission, I would make contact with them, even if the discharge summary did not request specific follow-up	**Disagree**
If the patient did not self-present to my surgery for follow-up, the ICU admission would prompt me to schedule a consultation with them	**Disagree**
If the patient did not self-present to my surgery for follow-up, the ICU admission would prompt me to schedule a consultation with the patient and a close relative	**Disagree**

Page 9

Q9 Have you ever attended an educational meeting at which you learned about the long term complications of critical illness?

No

Page 10

Q10 Are you aware of any published Guidelines about the rehabilitation of patients following hospital discharge after ICU admission with critical illness?

No

Page 11

Q11 Do you think it would benefit your patient and/or their family members if you received details about their ICU admission?

No

#6

COMPLETE

Collector: Web Link 1 (Web Link)
Started: Tuesday, June 21, 2016 11:55:12 AM
Last Modified: Tuesday, June 21, 2016 12:00:30 PM
Time Spent: 00:05:18
IP Address: 213.233.150.28

Page 1

Q1 Which of the following best describes your GP practice?

Urban practice (Dublin, Cork, Galway, Limerick)

Page 2

Q2 Please indicate how long you have been working as a general practitioner.

10 years to 20 years

Page 3

Q3 In which county do you conduct most of your GP work?

Dublin

Page 4

Q4 Please comment on the following statements regarding communication you receive after your patients are discharged from hospital.

I receive details of the patient's admission	**Often**
I receive details of their admission within 30days of patient's discharge	**Often**
The details I receive include whether patient was admitted to the ICU or not	**Occasionally**

Page 5

Q5 If you receive information about your patient's ICU stay, by which method(s) would you receive this information?

The ICU/anaesthetic staff phone me directly	**Never**
In a discharge summary or letter from ICU medical team	**Occasionally**
In a discharge summary or letter from other non-ICU medical/surgical team	**Often**
I contact the hospital myself to find out	**Occasionally**
The patient's relatives tell me	**Often**
The patient tells me after hospital discharge	**Often**

Page 6

Q6 If you received information about your patient's stay in ICU, how often would it include details about the following aspects of their critical illness?

Shock	**Occasionally**
Respiratory failure and mechanical ventilation	**Occasionally**
Acute kidney injury requiring acute dialysis	**Occasionally**
Acute encephalopathy / Delirium	**Occasionally**
ARDS (acute respiratory distress syndrome)	**Occasionally**
Neuromuscular weakness	**Occasionally**
Tracheostomy insertion	**Often**
The duration of patient's stay in ICU	**Occasionally**

Page 7

Q7 If, during a recent hospital stay, your patient was in ICU, would you record this ICU admission in the medical/surgical history section of their notes?

No

Page 8

Q8 You receive a discharge summary in the post about your patient who was recently discharged from hospital after a severe illness. The summary confirms that they were in ICU during the hospital stay. Please comment on the following statements about the patient's follow-up care:

Because of the patient's ICU admission, I would make contact with them, even if the discharge summary did not request specific follow-up	**Agree**
If the patient did not self-present to my surgery for follow-up, the ICU admission would prompt me to schedule a consultation with them	**Neutral**
If the patient did not self-present to my surgery for follow-up, the ICU admission would prompt me to schedule a consultation with the patient and a close relative	**Neutral**

Page 9

Q9 Have you ever attended an educational meeting at which you learned about the long term complications of critical illness?

No

Page 10

Q10 Are you aware of any published Guidelines about the rehabilitation of patients following hospital discharge after ICU admission with critical illness?

No

Page 11

Q11 Do you think it would benefit your patient and/or their family members if you received details about their ICU admission?

Yes

#7

COMPLETE

Collector: Web Link 1 (Web Link)
Started: Tuesday, June 21, 2016 8:43:02 PM
Last Modified: Tuesday, June 21, 2016 8:46:57 PM
Time Spent: 00:03:55
IP Address: 93.107.106.23

Page 1

Q1 Which of the following best describes your GP practice?

Urban practice (Dublin, Cork, Galway, Limerick)

Page 2

Q2 Please indicate how long you have been working as a general practitioner.

10 years to 20 years

Page 3

Q3 In which county do you conduct most of your GP work?

Dublin

Page 4

Q4 Please comment on the following statements regarding communication you receive after your patients are discharged from hospital.

I receive details of the patient’s admission **Often**

I receive details of their admission within 30days of patient’s discharge **Often**

The details I receive include whether patient was admitted to the ICU or not **Occasionally**

Page 5

Q5 If you receive information about your patient's ICU stay, by which method(s) would you receive this information?

The ICU/anaesthetic staff phone me directly	**Never**
In a discharge summary or letter from ICU medical team	**Never**
In a discharge summary or letter from other non-ICU medical/surgical team	**Occasionally**
I contact the hospital myself to find out	**Never**
The patient's relatives tell me	**Often**
The patient tells me after hospital discharge	**Often**

Page 6

Q6 If you received information about your patient's stay in ICU, how often would it include details about the following aspects of their critical illness?

Shock	**Never**
Respiratory failure and mechanical ventilation	**Rarely**
Acute kidney injury requiring acute dialysis	**Rarely**
Acute encephalopathy / Delirium	**Never**
ARDS (acute respiratory distress syndrome)	**Never**
Neuromuscular weakness	**Never**
Tracheostomy insertion	**Never**
The duration of patient's stay in ICU	**Never**

Page 7

Q7 If, during a recent hospital stay, your patient was in ICU, would you record this ICU admission in the medical/surgical history section of their notes? **Yes**

Page 8

Q8 You receive a discharge summary in the post about your patient who was recently discharged from hospital after a severe illness. The summary confirms that they were in ICU during the hospital stay. Please comment on the following statements about the patient’s follow-up care:

Because of the patient’s ICU admission, I would make contact with them, even if the discharge summary did not request specific follow-up	**Disagree**
If the patient did not self-present to my surgery for follow-up, the ICU admission would prompt me to schedule a consultation with them	**Disagree**
If the patient did not self-present to my surgery for follow-up, the ICU admission would prompt me to schedule a consultation with the patient and a close relative	**Strongly disagree**

Page 9

Q9 Have you ever attended an educational meeting at which you learned about the long term complications of critical illness?

No

Page 10

Q10 Are you aware of any published Guidelines about the rehabilitation of patients following hospital discharge after ICU admission with critical illness?

No

Page 11

Q11 Do you think it would benefit your patient and/or their family members if you received details about their ICU admission?

Yes

#8

COMPLETE

Collector: Web Link 1 (Web Link)
Started: Wednesday, June 22, 2016 9:03:26 AM
Last Modified: Wednesday, June 22, 2016 9:05:51 AM
Time Spent: 00:02:25
IP Address: 86.44.36.8

Page 1

Q1 Which of the following best describes your GP practice?

Urban practice (Dublin, Cork, Galway, Limerick)

Page 2

Q2 Please indicate how long you have been working as a general practitioner.

<10years

Page 3

Q3 In which county do you conduct most of your GP work?

Dublin

Page 4

Q4 Please comment on the following statements regarding communication you receive after your patients are discharged from hospital.

I receive details of the patient’s admission	**Often**
I receive details of their admission within 30days of patient’s discharge	**Often**
The details I receive include whether patient was admitted to the ICU or not	**Often**

Page 5

Q5 If you receive information about your patient’s ICU stay, by which method(s) would you receive this information?

The ICU/anaesthetic staff phone me directly	**Never**
In a discharge summary or letter from ICU medical team	**Never**
In a discharge summary or letter from other non-ICU medical/surgical team	**Often**
I contact the hospital myself to find out	**Occasionally**
The patient’s relatives tell me	**Often**
The patient tells me after hospital discharge	**Often**

Page 6

Q6 If you received information about your patient’s stay in ICU, how often would it include details about the following aspects of their critical illness?

Shock	**Often**
Respiratory failure and mechanical ventilation	**Often**
Acute kidney injury requiring acute dialysis	**Often**
Acute encephalopathy / Delirium	**Often**
ARDS (acute respiratory distress syndrome)	**Rarely**
Neuromuscular weakness	**Rarely**
Tracheostomy insertion	**Often**
The duration of patient’s stay in ICU	**Rarely**

Page 7

Q7 If, during a recent hospital stay, your patient was in ICU, would you record this ICU admission in the medical/surgical history section of their notes? **Yes**

Page 8

Q8 You receive a discharge summary in the post about your patient who was recently discharged from hospital after a severe illness. The summary confirms that they were in ICU during the hospital stay. Please comment on the following statements about the patient's follow-up care:

Because of the patient's ICU admission, I would make contact with them, even if the discharge summary did not request specific follow-up	**Agree**
If the patient did not self-present to my surgery for follow-up, the ICU admission would prompt me to schedule a consultation with them	**Agree**
If the patient did not self-present to my surgery for follow-up, the ICU admission would prompt me to schedule a consultation with the patient and a close relative	**Neutral**

Page 9

Q9 Have you ever attended an educational meeting at which you learned about the long term complications of critical illness?

No

Page 10

Q10 Are you aware of any published Guidelines about the rehabilitation of patients following hospital discharge after ICU admission with critical illness?

No

Page 11

Q11 Do you think it would benefit your patient and/or their family members if you received details about their ICU admission?

Yes

#9

COMPLETE

Collector: Web Link 1 (Web Link)
Started: Thursday, June 23, 2016 8:35:20 AM
Last Modified: Thursday, June 23, 2016 8:38:09 AM
Time Spent: 00:02:49
IP Address: 86.43.125.61

Page 1

Q1 Which of the following best describes your GP practice?

Rural practice (outside city)

Page 2

Q2 Please indicate how long you have been working as a general practitioner.

>20years

Page 3

Q3 In which county do you conduct most of your GP work?

louth

Page 4

Q4 Please comment on the following statements regarding communication you receive after your patients are discharged from hospital.

I receive details of the patient's admission **Often**

I receive details of their admission within 30days of patient's discharge **Often**

The details I receive include whether patient was admitted to the ICU or not **Occasionally**

Page 5

Q5 If you receive information about your patient’s ICU stay, by which method(s) would you receive this information?

The ICU/anaesthetic staff phone me directly	**Rarely**
In a discharge summary or letter from ICU medical team	**Rarely**
In a discharge summary or letter from other non-ICU medical/surgical team	**Often**
I contact the hospital myself to find out	**Occasionally**
The patient’s relatives tell me	**Occasionally**
The patient tells me after hospital discharge	**Occasionally**

Page 6

Q6 If you received information about your patient’s stay in ICU, how often would it include details about the following aspects of their critical illness?

Shock	**Often**
Respiratory failure and mechanical ventilation	**Always**
Acute kidney injury requiring acute dialysis	**Always**
Acute encephalopathy / Delirium	**Always**
ARDS (acute respiratory distress syndrome)	**Always**
Neuromuscular weakness	**Always**
Tracheostomy insertion	**Always**
The duration of patient’s stay in ICU	**Occasionally**

Page 7

Q7 If, during a recent hospital stay, your patient was in ICU, would you record this ICU admission in the medical/surgical history section of their notes?

No

Page 8

Q8 You receive a discharge summary in the post about your patient who was recently discharged from hospital after a severe illness. The summary confirms that they were in ICU during the hospital stay. Please comment on the following statements about the patient's follow-up care:

Because of the patient's ICU admission, I would make contact with them, even if the discharge summary did not request specific follow-up	**Disagree**
If the patient did not self-present to my surgery for follow-up, the ICU admission would prompt me to schedule a consultation with them	**Disagree**
If the patient did not self-present to my surgery for follow-up, the ICU admission would prompt me to schedule a consultation with the patient and a close relative	**Disagree**

Page 9

Q9 Have you ever attended an educational meeting at which you learned about the long term complications of critical illness?

No

Page 10

Q10 Are you aware of any published Guidelines about the rehabilitation of patients following hospital discharge after ICU admission with critical illness?

No

Page 11

Q11 Do you think it would benefit your patient and/or their family members if you received details about their ICU admission?

Yes

#10

COMPLETE

Collector: Web Link 1 (Web Link)
Started: Thursday, June 23, 2016 9:22:58 AM
Last Modified: Thursday, June 23, 2016 9:25:09 AM
Time Spent: 00:02:11
IP Address: 83.71.21.9

Page 1

Q1 Which of the following best describes your GP practice?

Rural practice (outside city)

Page 2

Q2 Please indicate how long you have been working as a general practitioner.

10 years to 20 years

Page 3

Q3 In which county do you conduct most of your GP work?

wicklow

Page 4

Q4 Please comment on the following statements regarding communication you receive after your patients are discharged from hospital.

I receive details of the patient’s admission	**Occasionally**
I receive details of their admission within 30days of patient’s discharge	**Often**
The details I receive include whether patient was admitted to the ICU or not	**Often**

Page 5

Q5 If you receive information about your patient's ICU stay, by which method(s) would you receive this information?

The ICU/anaesthetic staff phone me directly	**Never**
In a discharge summary or letter from ICU medical team	**Rarely**
In a discharge summary or letter from other non-ICU medical/surgical team	**Occasionally**
I contact the hospital myself to find out	**Occasionally**
The patient's relatives tell me	**Often**
The patient tells me after hospital discharge	**Often**

Page 6

Q6 If you received information about your patient's stay in ICU, how often would it include details about the following aspects of their critical illness?

Shock	**Rarely**
Respiratory failure and mechanical ventilation	**Rarely**
Acute kidney injury requiring acute dialysis	**Occasionally**
Acute encephalopathy / Delirium	**Occasionally**
ARDS (acute respiratory distress syndrome)	**Rarely**
Neuromuscular weakness	**Never**
Tracheostomy insertion	**Never**
The duration of patient's stay in ICU	**Rarely**

Page 7

Q7 If, during a recent hospital stay, your patient was in ICU, would you record this ICU admission in the medical/surgical history section of their notes? **Yes**

Page 8

Q8 You receive a discharge summary in the post about your patient who was recently discharged from hospital after a severe illness. The summary confirms that they were in ICU during the hospital stay. Please comment on the following statements about the patient's follow-up care:

Because of the patient's ICU admission, I would make contact with them, even if the discharge summary did not request specific follow-up	**Disagree**
If the patient did not self-present to my surgery for follow-up, the ICU admission would prompt me to schedule a consultation with them	**Disagree**
If the patient did not self-present to my surgery for follow-up, the ICU admission would prompt me to schedule a consultation with the patient and a close relative	**Neutral**

Page 9

Q9 Have you ever attended an educational meeting at which you learned about the long term complications of critical illness?

No,
If Yes, what was the event? If No, would you attend one if it was available?:
yes

Page 10

Q10 Are you aware of any published Guidelines about the rehabilitation of patients following hospital discharge after ICU admission with critical illness?

No

Page 11

Q11 Do you think it would benefit your patient and/or their family members if you received details about their ICU admission?

Yes

#11

COMPLETE

Collector: Web Link 1 (Web Link)
Started: Thursday, June 23, 2016 1:36:02 PM
Last Modified: Thursday, June 23, 2016 1:40:43 PM
Time Spent: 00:04:41
IP Address: 86.44.238.167

Page 1

Q1 Which of the following best describes your GP practice?

Rural practice (outside city)

Page 2

Q2 Please indicate how long you have been working as a general practitioner.

10 years to 20 years

Page 3

Q3 In which county do you conduct most of your GP work?

Ireland

Page 4

Q4 Please comment on the following statements regarding communication you receive after your patients are discharged from hospital.

I receive details of the patient's admission	**Rarely**
I receive details of their admission within 30days of patient's discharge	**Occasionally**
The details I receive include whether patient was admitted to the ICU or not	**Rarely**

Page 5

Q5 If you receive information about your patient's ICU stay, by which method(s) would you receive this information?

The ICU/anaesthetic staff phone me directly	**Never**
In a discharge summary or letter from ICU medical team	**Never**
In a discharge summary or letter from other non-ICU medical/surgical team	**Occasionally**
The patient's relatives tell me	**Occasionally**
The patient tells me after hospital discharge	**Often**

Page 6

Q6 If you received information about your patient's stay in ICU, how often would it include details about the following aspects of their critical illness?

Shock	**Never**
Respiratory failure and mechanical ventilation	**Never**
Acute kidney injury requiring acute dialysis	**Never**
Acute encephalopathy / Delirium	**Never**
ARDS (acute respiratory distress syndrome)	**Never**
Neuromuscular weakness	**Never**
Tracheostomy insertion	**Never**
The duration of patient's stay in ICU	**Never**

Page 7

Q7 If, during a recent hospital stay, your patient was in ICU, would you record this ICU admission in the medical/surgical history section of their notes? **Yes**

Page 8

Q8 You receive a discharge summary in the post about your patient who was recently discharged from hospital after a severe illness. The summary confirms that they were in ICU during the hospital stay. Please comment on the following statements about the patient's follow-up care:

Because of the patient's ICU admission, I would make contact with them, even if the discharge summary did not request specific follow-up	**Disagree**
If the patient did not self-present to my surgery for follow-up, the ICU admission would prompt me to schedule a consultation with them	**Agree**
If the patient did not self-present to my surgery for follow-up, the ICU admission would prompt me to schedule a consultation with the patient and a close relative	**Disagree**

Page 9

Q9 Have you ever attended an educational meeting at which you learned about the long term complications of critical illness?

No

Page 10

Q10 Are you aware of any published Guidelines about the rehabilitation of patients following hospital discharge after ICU admission with critical illness?

No

Page 11

Q11 Do you think it would benefit your patient and/or their family members if you received details about their ICU admission?

Yes

#12

COMPLETE

Collector: Web Link 1 (Web Link)
Started: Thursday, June 23, 2016 3:17:30 PM
Last Modified: Thursday, June 23, 2016 3:20:35 PM
Time Spent: 00:03:05
IP Address: 86.45.65.225

Page 1

Q1 Which of the following best describes your GP practice?

Rural practice (outside city)

Page 2

Q2 Please indicate how long you have been working as a general practitioner.

10 years to 20 years

Page 3

Q3 In which county do you conduct most of your GP work?

Sligo

Page 4

Q4 Please comment on the following statements regarding communication you receive after your patients are discharged from hospital.

I receive details of the patient’s admission	**Often**
I receive details of their admission within 30days of patient’s discharge	**Often**
The details I receive include whether patient was admitted to the ICU or not	**Often**

Page 5

Q5 If you receive information about your patient's ICU stay, by which method(s) would you receive this information?

The ICU/anaesthetic staff phone me directly	**Never**
In a discharge summary or letter from ICU medical team	**Never**
In a discharge summary or letter from other non-ICU medical/surgical team	**Often**
I contact the hospital myself to find out	**Rarely**
The patient's relatives tell me	**Often**
The patient tells me after hospital discharge	**Often**

Page 6

Q6 If you received information about your patient's stay in ICU, how often would it include details about the following aspects of their critical illness?

Shock	**Rarely**
Respiratory failure and mechanical ventilation	**Often**
Acute kidney injury requiring acute dialysis	**Rarely**
Acute encephalopathy / Delirium	**Rarely**
ARDS (acute respiratory distress syndrome)	**Occasionally**
Neuromuscular weakness	**Never**
Tracheostomy insertion	**Never**
The duration of patient's stay in ICU	**Rarely**

Page 7

**Q7** If, during a recent hospital stay, your patient was in ICU, would you record this ICU admission in the medical/surgical history section of their notes?	**Yes**

Page 8

Q8 You receive a discharge summary in the post about your patient who was recently discharged from hospital after a severe illness. The summary confirms that they were in ICU during the hospital stay. Please comment on the following statements about the patient's follow-up care:

Because of the patient's ICU admission, I would make contact with them, even if the discharge summary did not request specific follow-up	**Disagree**
If the patient did not self-present to my surgery for follow-up, the ICU admission would prompt me to schedule a consultation with them	**Disagree**
If the patient did not self-present to my surgery for follow-up, the ICU admission would prompt me to schedule a consultation with the patient and a close relative	**Disagree**

Page 9

Q9 Have you ever attended an educational meeting at which you learned about the long term complications of critical illness?

No,
If Yes, what was the event? If No, would you attend one if it was available?:
Possibly

Page 10

Q10 Are you aware of any published Guidelines about the rehabilitation of patients following hospital discharge after ICU admission with critical illness?

No

Page 11

Q11 Do you think it would benefit your patient and/or their family members if you received details about their ICU admission?

Yes

#13

INCOMPLETE

Collector: Web Link 1 (Web Link)
Started: Friday, June 24, 2016 1:12:54 PM
Last Modified: Friday, June 24, 2016 1:14:36 PM
Time Spent: 00:01:42
IP Address: 92.61.200.19

Page 1

Q1 Which of the following best describes your GP practice?

Rural practice (outside city)

Page 2

Q2 Please indicate how long you have been working as a general practitioner.

>20years

Page 3

Q3 In which county do you conduct most of your GP work?

Longford

Page 4

Q4 Please comment on the following statements regarding communication you receive after your patients are discharged from hospital.

I receive details of the patient's admission	**Often**
I receive details of their admission within 30days of patient's discharge	**Often**
The details I receive include whether patient was admitted to the ICU or not	**Occasionally**

Page 5

Q5 If you receive information about your patient’s ICU stay, by which method(s) would you receive this information?

The ICU/anaesthetic staff phone me directly	**Never**
In a discharge summary or letter from ICU medical team	**Never**
In a discharge summary or letter from other non-ICU medical/surgical team	**Often**
The patient’s relatives tell me	**Often**
The patient tells me after hospital discharge	**Often**

Page 6

Q6 If you received information about your patient’s stay in ICU, how often would it include details about the following aspects of their critical illness?

Shock	**Occasionally**
Respiratory failure and mechanical ventilation	**Occasionally**
Acute kidney injury requiring acute dialysis	**Occasionally**
Acute encephalopathy / Delirium	**Occasionally**
ARDS (acute respiratory distress syndrome)	**Occasionally**
Neuromuscular weakness	**Occasionally**

Page 7

Q7 If, during a recent hospital stay, your patient was in ICU, would you record this ICU admission in the medical/surgical history section of their notes?

Respondent skipped this question

Page 8

Q8 You receive a discharge summary in the post about your patient who was recently discharged from hospital after a severe illness. The summary confirms that they were in ICU during the hospital stay. Please comment on the following statements about the patient’s follow-up care:

Respondent skipped this question

Page 9

Q9 Have you ever attended an educational meeting at which you learned about the long term complications of critical illness?

Respondent skipped this question

Page 10

Q10 Are you aware of any published Guidelines about the rehabilitation of patients following hospital discharge after ICU admission with critical illness?

Respondent skipped this question

Page 11

Q11 Do you think it would benefit your patient and/or their family members if you received details about their ICU admission?

Respondent skipped this question

#14

COMPLETE

Collector: Web Link 1 (Web Link)
Started: Friday, June 24, 2016 1:50:11 PM
Last Modified: Friday, June 24, 2016 1:53:28 PM
Time Spent: 00:03:17
IP Address: 86.47.56.245

Page 1

Q1 Which of the following best describes your GP practice?

Urban practice (Dublin, Cork, Galway, Limerick)

Page 2

Q2 Please indicate how long you have been working as a general practitioner.

<10years

Page 3

Q3 In which county do you conduct most of your GP work?

Dublin

Page 4

Q4 Please comment on the following statements regarding communication you receive after your patients are discharged from hospital.

I receive details of the patient’s admission	**Often**
I receive details of their admission within 30days of patient’s discharge	**Occasionally**
The details I receive include whether patient was admitted to the ICU or not	**Occasionally**

Page 5

Q5 If you receive information about your patient's ICU stay, by which method(s) would you receive this information?

The ICU/anaesthetic staff phone me directly	**Never**
In a discharge summary or letter from other non-ICU medical/surgical team	**Often**
I contact the hospital myself to find out	**Never**
The patient's relatives tell me	**Occasionally**
The patient tells me after hospital discharge	**Never**

Page 6

Q6 If you received information about your patient's stay in ICU, how often would it include details about the following aspects of their critical illness?

Shock	**Never**
Respiratory failure and mechanical ventilation	**Often**
Acute kidney injury requiring acute dialysis	**Occasionally**
Acute encephalopathy / Delirium	**Never**
ARDS (acute respiratory distress syndrome)	**Never**
Neuromuscular weakness	**Never**
Tracheostomy insertion	**Rarely**
The duration of patient's stay in ICU	**Often**

Page 7

Q7 If, during a recent hospital stay, your patient was in ICU, would you record this ICU admission in the medical/surgical history section of their notes?

Yes

Page 8

Q8 You receive a discharge summary in the post about your patient who was recently discharged from hospital after a severe illness. The summary confirms that they were in ICU during the hospital stay. Please comment on the following statements about the patient's follow-up care:

Because of the patient's ICU admission, I would make contact with them, even if the discharge summary did not request specific follow-up	**Agree**
If the patient did not self-present to my surgery for follow-up, the ICU admission would prompt me to schedule a consultation with them	**Agree**
If the patient did not self-present to my surgery for follow-up, the ICU admission would prompt me to schedule a consultation with the patient and a close relative	**Neutral**

Page 9

Q9 Have you ever attended an educational meeting at which you learned about the long term complications of critical illness?

No

Page 10

Q10 Are you aware of any published Guidelines about the rehabilitation of patients following hospital discharge after ICU admission with critical illness?

No

Page 11

Q11 Do you think it would benefit your patient and/or their family members if you received details about their ICU admission?

Yes

#15

COMPLETE

Collector: Web Link 1 (Web Link)
Started: Saturday, June 25, 2016 10:49:53 PM
Last Modified: Saturday, June 25, 2016 10:54:58 PM
Time Spent: 00:05:05
IP Address: 86.41.65.143

Page 1

Q1 Which of the following best describes your GP practice?

Rural practice (outside city)

Page 2

Q2 Please indicate how long you have been working as a general practitioner.

10 years to 20 years

Page 3

Q3 In which county do you conduct most of your GP work?

Cork

Page 4

Q4 Please comment on the following statements regarding communication you receive after your patients are discharged from hospital.

I receive details of the patient's admission	**Often**
I receive details of their admission within 30days of patient's discharge	**Often**
The details I receive include whether patient was admitted to the ICU or not	**Occasionally**

Page 5

Q5 If you receive information about your patient's ICU stay, by which method(s) would you receive this information?

The ICU/anaesthetic staff phone me directly	**Never**
In a discharge summary or letter from ICU medical team	**Never**
In a discharge summary or letter from other non-ICU medical/surgical team	**Occasionally**
I contact the hospital myself to find out	**Never**
The patient's relatives tell me	**Often**
The patient tells me after hospital discharge	**Occasionally**

Page 6

Q6 If you received information about your patient's stay in ICU, how often would it include details about the following aspects of their critical illness?

Shock	**Never**
Respiratory failure and mechanical ventilation	**Occasionally**
Acute kidney injury requiring acute dialysis	**Occasionally**
Acute encephalopathy / Delirium	**Rarely**
ARDS (acute respiratory distress syndrome)	**Rarely**
Neuromuscular weakness	**Rarely**
Tracheostomy insertion	**Rarely**
The duration of patient's stay in ICU	**Never**

Page 7

Q7 If, during a recent hospital stay, your patient was in ICU, would you record this ICU admission in the medical/surgical history section of their notes? **No**

Page 8

Q8 You receive a discharge summary in the post about your patient who was recently discharged from hospital after a severe illness. The summary confirms that they were in ICU during the hospital stay. Please comment on the following statements about the patient's follow-up care:

Because of the patient's ICU admission, I would make contact with them, even if the discharge summary did not request specific follow-up	**Neutral**
If the patient did not self-present to my surgery for follow-up, the ICU admission would prompt me to schedule a consultation with them	**Neutral**
If the patient did not self-present to my surgery for follow-up, the ICU admission would prompt me to schedule a consultation with the patient and a close relative	**Neutral**

Page 9

Q9 Have you ever attended an educational meeting at which you learned about the long term complications of critical illness?

No

Page 10

Q10 Are you aware of any published Guidelines about the rehabilitation of patients following hospital discharge after ICU admission with critical illness?

No

Page 11

Q11 Do you think it would benefit your patient and/or their family members if you received details about their ICU admission?

Yes,
If Yes, what the possible benefits?:
Awareness of possible stress issues for the patient

#16

COMPLETE

Collector: Web Link 1 (Web Link)
Started: Sunday, June 26, 2016 9:12:38 AM
Last Modified: Sunday, June 26, 2016 9:19:12 AM
Time Spent: 00:06:34
IP Address: 78.16.49.91

Page 1

Q1 Which of the following best describes your GP practice?

Rural practice (outside city)

Page 2

Q2 Please indicate how long you have been working as a general practitioner.

>20years

Page 3

Q3 In which county do you conduct most of your GP work?

Monaghan

Page 4

Q4 Please comment on the following statements regarding communication you receive after your patients are discharged from hospital.

I receive details of the patient's admission	**Often**
I receive details of their admission within 30days of patient's discharge	**Often**
The details I receive include whether patient was admitted to the ICU or not	**Never**

Page 5

Q5 If you receive information about your patient's ICU stay, by which method(s) would you receive this information?

The ICU/anaesthetic staff phone me directly	**Never**
In a discharge summary or letter from ICU medical team	**Never**
In a discharge summary or letter from other non-ICU medical/surgical team	**Never**
I contact the hospital myself to find out	**Occasionally**
The patient's relatives tell me	**Often**
The patient tells me after hospital discharge	**Often**

Page 6

Q6 If you received information about your patient's stay in ICU, how often would it include details about the following aspects of their critical illness?

Shock	**Never**
Respiratory failure and mechanical ventilation	**Never**
Acute kidney injury requiring acute dialysis	**Never**
Acute encephalopathy / Delirium	**Never**
ARDS (acute respiratory distress syndrome)	**Never**
Neuromuscular weakness	**Never**
Tracheostomy insertion	**Never**
The duration of patient's stay in ICU	**Never**

Page 7

**Q7** If, during a recent hospital stay, your patient was in ICU, would you record this ICU admission in the medical/surgical history section of their notes?	**Yes**

Page 8

Q8 You receive a discharge summary in the post about your patient who was recently discharged from hospital after a severe illness. The summary confirms that they were in ICU during the hospital stay. Please comment on the following statements about the patient's follow-up care:

Because of the patient's ICU admission, I would make contact with them, even if the discharge summary did not request specific follow-up	**Neutral**
If the patient did not self-present to my surgery for follow-up, the ICU admission would prompt me to schedule a consultation with them	**Neutral**
If the patient did not self-present to my surgery for follow-up, the ICU admission would prompt me to schedule a consultation with the patient and a close relative	**Neutral**

Page 9

Q9 Have you ever attended an educational meeting at which you learned about the long term complications of critical illness?

No

Page 10

Q10 Are you aware of any published Guidelines about the rehabilitation of patients following hospital discharge after ICU admission with critical illness?

No

Page 11

Q11 Do you think it would benefit your patient and/or their family members if you received details about their ICU admission?

Yes

#17

COMPLETE

Collector: Web Link 1 (Web Link)
Started: Monday, June 27, 2016 11:38:58 AM
Last Modified: Monday, June 27, 2016 11:42:47 AM
Time Spent: 00:03:49
IP Address: 86.47.54.184

Page 1

Q1 Which of the following best describes your GP practice?

Rural practice (outside city)

Page 2

Q2 Please indicate how long you have been working as a general practitioner.

10 years to 20 years

Page 3

Q3 In which county do you conduct most of your GP work?

Ireland

Page 4

Q4 Please comment on the following statements regarding communication you receive after your patients are discharged from hospital.

I receive details of the patient's admission	**Rarely**
I receive details of their admission within 30days of patient's discharge	**Rarely**
The details I receive include whether patient was admitted to the ICU or not	**Rarely**

Page 5

Q5 If you receive information about your patient's ICU stay, by which method(s) would you receive this information?

The ICU/anaesthetic staff phone me directly	**Rarely**
In a discharge summary or letter from ICU medical team	**Never**
In a discharge summary or letter from other non-ICU medical/surgical team	**Occasionally**
I contact the hospital myself to find out	**Never**
The patient's relatives tell me	**Often**
The patient tells me after hospital discharge	**Often**

Page 6

Q6 If you received information about your patient's stay in ICU, how often would it include details about the following aspects of their critical illness?

Shock	**Often**
Respiratory failure and mechanical ventilation	**Often**
Acute kidney injury requiring acute dialysis	**Often**
Acute encephalopathy / Delirium	**Often**
ARDS (acute respiratory distress syndrome)	**Occasionally**
Neuromuscular weakness	**Rarely**
Tracheostomy insertion	**Rarely**
The duration of patient's stay in ICU	**Often**

Page 7

Q7 If, during a recent hospital stay, your patient was in ICU, would you record this ICU admission in the medical/surgical history section of their notes? **Yes**

Page 8

Q8 You receive a discharge summary in the post about your patient who was recently discharged from hospital after a severe illness. The summary confirms that they were in ICU during the hospital stay. Please comment on the following statements about the patient's follow-up care:

Because of the patient's ICU admission, I would make contact with them, even if the discharge summary did not request specific follow-up	**Agree**
If the patient did not self-present to my surgery for follow-up, the ICU admission would prompt me to schedule a consultation with them	**Disagree**
If the patient did not self-present to my surgery for follow-up, the ICU admission would prompt me to schedule a consultation with the patient and a close relative	**Strongly disagree**

Page 9

Q9 Have you ever attended an educational meeting at which you learned about the long term complications of critical illness?

No

Page 10

Q10 Are you aware of any published Guidelines about the rehabilitation of patients following hospital discharge after ICU admission with critical illness?

No

Page 11

Q11 Do you think it would benefit your patient and/or their family members if you received details about their ICU admission?

Yes

#18

COMPLETE

Collector: Web Link 1 (Web Link)
Started: Monday, June 27, 2016 12:58:16 PM
Last Modified: Monday, June 27, 2016 1:02:51 PM
Time Spent: 00:04:35
IP Address: 217.114.169.241

Page 1

Q1 Which of the following best describes your GP practice?

Urban practice (Dublin, Cork, Galway, Limerick)

Page 2

Q2 Please indicate how long you have been working as a general practitioner.

>20years

Page 3

Q3 In which county do you conduct most of your GP work?

Dublin

Page 4

Q4 Please comment on the following statements regarding communication you receive after your patients are discharged from hospital.

I receive details of the patient’s admission	**Often**
I receive details of their admission within 30days of patient’s discharge	**Often**
The details I receive include whether patient was admitted to the ICU or not	**Often**

Page 5

Q5 If you receive information about your patient's ICU stay, by which method(s) would you receive this information?

The ICU/anaesthetic staff phone me directly	**Never**
In a discharge summary or letter from ICU medical team	**Never**
In a discharge summary or letter from other non-ICU medical/surgical team	**Often**
I contact the hospital myself to find out	**Never**
The patient's relatives tell me	**Occasionally**
The patient tells me after hospital discharge	**Often**

Page 6

Q6 If you received information about your patient's stay in ICU, how often would it include details about the following aspects of their critical illness?

Shock	**Never**
Respiratory failure and mechanical ventilation	**Occasionally**
Acute kidney injury requiring acute dialysis	**Often**
Acute encephalopathy / Delirium	**Occasionally**
ARDS (acute respiratory distress syndrome)	**Occasionally**
Neuromuscular weakness	**Occasionally**
Tracheostomy insertion	**Occasionally**
The duration of patient's stay in ICU	**Occasionally**

Page 7

Q7 If, during a recent hospital stay, your patient was in ICU, would you record this ICU admission in the medical/surgical history section of their notes? **Yes**

Page 8

Q8 You receive a discharge summary in the post about your patient who was recently discharged from hospital after a severe illness. The summary confirms that they were in ICU during the hospital stay. Please comment on the following statements about the patient's follow-up care:

Because of the patient's ICU admission, I would make contact with them, even if the discharge summary did not request specific follow-up	**Neutral**
If the patient did not self-present to my surgery for follow-up, the ICU admission would prompt me to schedule a consultation with them	**Disagree**
If the patient did not self-present to my surgery for follow-up, the ICU admission would prompt me to schedule a consultation with the patient and a close relative	**Disagree**

Page 9

Q9 Have you ever attended an educational meeting at which you learned about the long term complications of critical illness?

No

Page 10

Q10 Are you aware of any published Guidelines about the rehabilitation of patients following hospital discharge after ICU admission with critical illness?

No

Page 11

Q11 Do you think it would benefit your patient and/or their family members if you received details about their ICU admission?

Yes

#19

COMPLETE

Collector: Web Link 1 (Web Link)
Started: Monday, June 27, 2016 2:13:45 PM
Last Modified: Monday, June 27, 2016 2:17:28 PM
Time Spent: 00:03:43
IP Address: 82.141.234.104

Page 1

Q1 Which of the following best describes your GP practice?

Rural practice (outside city)

Page 2

Q2 Please indicate how long you have been working as a general practitioner.

>20years

Page 3

Q3 In which county do you conduct most of your GP work?

Tipperary

Page 4

Q4 Please comment on the following statements regarding communication you receive after your patients are discharged from hospital.

I receive details of the patient's admission	**Often**
I receive details of their admission within 30days of patient's discharge	**Often**
The details I receive include whether patient was admitted to the ICU or not	**Occasionally**

Page 5

Q5 If you receive information about your patient's ICU stay, by which method(s) would you receive this information?

The ICU/anaesthetic staff phone me directly	**Never**
In a discharge summary or letter from ICU medical team	**Never**
In a discharge summary or letter from other non-ICU medical/surgical team	**Occasionally**
I contact the hospital myself to find out	**Occasionally**
The patient's relatives tell me	**Often**
The patient tells me after hospital discharge	**Often**

Page 6

Q6 If you received information about your patient's stay in ICU, how often would it include details about the following aspects of their critical illness?

Shock	**Never**
Respiratory failure and mechanical ventilation	**Never**
Acute kidney injury requiring acute dialysis	**Never**
Acute encephalopathy / Delirium	**Never**
ARDS (acute respiratory distress syndrome)	**Never**
Neuromuscular weakness	**Never**
Tracheostomy insertion	**Never**
The duration of patient's stay in ICU	**Never**

Page 7

Q7 If, during a recent hospital stay, your patient was in ICU, would you record this ICU admission in the medical/surgical history section of their notes? **No**

Page 8

Q8 You receive a discharge summary in the post about your patient who was recently discharged from hospital after a severe illness. The summary confirms that they were in ICU during the hospital stay. Please comment on the following statements about the patient's follow-up care:

Because of the patient's ICU admission, I would make contact with them, even if the discharge summary did not request specific follow-up	**Neutral**
If the patient did not self-present to my surgery for follow-up, the ICU admission would prompt me to schedule a consultation with them	**Neutral**
If the patient did not self-present to my surgery for follow-up, the ICU admission would prompt me to schedule a consultation with the patient and a close relative	**Neutral**

Page 9

Q9 Have you ever attended an educational meeting at which you learned about the long term complications of critical illness?

No

Page 10

Q10 Are you aware of any published Guidelines about the rehabilitation of patients following hospital discharge after ICU admission with critical illness?

No

Page 11

Q11 Do you think it would benefit your patient and/or their family members if you received details about their ICU admission?

Yes

#20

COMPLETE

Collector: Web Link 1 (Web Link)
Started: Monday, June 27, 2016 5:47:04 PM
Last Modified: Monday, June 27, 2016 5:53:20 PM
Time Spent: 00:06:16
IP Address: 213.233.147.129

Page 1

Q1 Which of the following best describes your GP practice?

Urban practice (Dublin, Cork, Galway, Limerick)

Page 2

Q2 Please indicate how long you have been working as a general practitioner.

10 years to 20 years

Page 3

Q3 In which county do you conduct most of your GP work?

Dublin

Page 4

Q4 Please comment on the following statements regarding communication you receive after your patients are discharged from hospital.

I receive details of the patient’s admission	**Often**
I receive details of their admission within 30days of patient’s discharge	**Often**
The details I receive include whether patient was admitted to the ICU or not	**Always**

Page 5

Q5 If you receive information about your patient's ICU stay, by which method(s) would you receive this information?

The ICU/anaesthetic staff phone me directly	**Never**
In a discharge summary or letter from ICU medical team	**Never**
In a discharge summary or letter from other non-ICU medical/surgical team	**Often**
I contact the hospital myself to find out	**Rarely**
The patient's relatives tell me	**Occasionally**
The patient tells me after hospital discharge	**Occasionally**

Page 6

Q6 If you received information about your patient's stay in ICU, how often would it include details about the following aspects of their critical illness?

Shock	**Always**
Respiratory failure and mechanical ventilation	**Always**
Acute kidney injury requiring acute dialysis	**Always**
Acute encephalopathy / Delirium	**Always**
ARDS (acute respiratory distress syndrome)	**Always**
Neuromuscular weakness	**Occasionally**
Tracheostomy insertion	**Always**
The duration of patient's stay in ICU	**Occasionally**

Page 7

Q7 If, during a recent hospital stay, your patient was in ICU, would you record this ICU admission in the medical/surgical history section of their notes?

No

Page 8

Q8 You receive a discharge summary in the post about your patient who was recently discharged from hospital after a severe illness. The summary confirms that they were in ICU during the hospital stay. Please comment on the following statements about the patient's follow-up care:

Because of the patient's ICU admission, I would make contact with them, even if the discharge summary did not request specific follow-up	**Disagree**
If the patient did not self-present to my surgery for follow-up, the ICU admission would prompt me to schedule a consultation with them	**Disagree**
If the patient did not self-present to my surgery for follow-up, the ICU admission would prompt me to schedule a consultation with the patient and a close relative	**Disagree**

Page 9

Q9 Have you ever attended an educational meeting at which you learned about the long term complications of critical illness?

No

Page 10

Q10 Are you aware of any published Guidelines about the rehabilitation of patients following hospital discharge after ICU admission with critical illness?

No

Page 11

Q11 Do you think it would benefit your patient and/or their family members if you received details about their ICU admission?

Yes,
If Yes, what the possible benefits?:
Need to know for follow up

#21

COMPLETE

Collector: Web Link 1 (Web Link)
Started: Monday, June 27, 2016 8:37:03 PM
Last Modified: Monday, June 27, 2016 8:40:04 PM
Time Spent: 00:03:01
IP Address: 37.228.228.63

Page 1

Q1 Which of the following best describes your GP practice?

Urban practice (Dublin, Cork, Galway, Limerick)

Page 2

Q2 Please indicate how long you have been working as a general practitioner.

10 years to 20 years

Page 3

Q3 In which county do you conduct most of your GP work?

Dublin

Page 4

Q4 Please comment on the following statements regarding communication you receive after your patients are discharged from hospital.

I receive details of the patient’s admission **Occasionally**

I receive details of their admission within 30days of patient’s discharge **Occasionally**

The details I receive include whether patient was admitted to the ICU or not **Occasionally**

Page 5

Q5 If you receive information about your patient's ICU stay, by which method(s) would you receive this information?

The ICU/anaesthetic staff phone me directly	**Never**
In a discharge summary or letter from ICU medical team	**Never**
In a discharge summary or letter from other non-ICU medical/surgical team	**Often**
I contact the hospital myself to find out	**Occasionally**
The patient's relatives tell me	**Often**
The patient tells me after hospital discharge	**Often**

Page 6

Q6 If you received information about your patient's stay in ICU, how often would it include details about the following aspects of their critical illness?

Shock	**Never**
Respiratory failure and mechanical ventilation	**Occasionally**
Acute kidney injury requiring acute dialysis	**Occasionally**
Acute encephalopathy / Delirium	**Never**
ARDS (acute respiratory distress syndrome)	**Occasionally**
Neuromuscular weakness	**Never**
Tracheostomy insertion	**Occasionally**
The duration of patient's stay in ICU	**Occasionally**

Page 7

Q7 If, during a recent hospital stay, your patient was in ICU, would you record this ICU admission in the medical/surgical history section of their notes? **Yes**

Page 8

Q8 You receive a discharge summary in the post about your patient who was recently discharged from hospital after a severe illness. The summary confirms that they were in ICU during the hospital stay. Please comment on the following statements about the patient’s follow-up care:

Because of the patient’s ICU admission, I would make contact with them, even if the discharge summary did not request specific follow-up	**Agree**
If the patient did not self-present to my surgery for follow-up, the ICU admission would prompt me to schedule a consultation with them	**Neutral**
If the patient did not self-present to my surgery for follow-up, the ICU admission would prompt me to schedule a consultation with the patient and a close relative	**Neutral**

Page 9

Q9 Have you ever attended an educational meeting at which you learned about the long term complications of critical illness?

No,
If Yes, what was the event? If No, would you attend one if it was available?:
Yes

Page 10

Q10 Are you aware of any published Guidelines about the rehabilitation of patients following hospital discharge after ICU admission with critical illness?

No

Page 11

Q11 Do you think it would benefit your patient and/or their family members if you received details about their ICU admission?

Yes

#22

COMPLETE

Collector: Web Link 1 (Web Link)
Started: Monday, June 27, 2016 8:45:38 PM
Last Modified: Monday, June 27, 2016 8:48:56 PM
Time Spent: 00:03:18
IP Address: 80.111.199.170

Page 1

Q1 Which of the following best describes your GP practice?

Urban practice (Dublin, Cork, Galway, Limerick)

Page 2

Q2 Please indicate how long you have been working as a general practitioner.

<10years

Page 3

Q3 In which county do you conduct most of your GP work?

Dublin

Page 4

Q4 Please comment on the following statements regarding communication you receive after your patients are discharged from hospital.

I receive details of the patient's admission	**Often**
I receive details of their admission within 30days of patient's discharge	**Often**
The details I receive include whether patient was admitted to the ICU or not	**Often**

Page 5

Q5 If you receive information about your patient's ICU stay, by which method(s) would you receive this information?

The ICU/anaesthetic staff phone me directly	**Never**
In a discharge summary or letter from ICU medical team	**Never**
In a discharge summary or letter from other non-ICU medical/surgical team	**Rarely**
I contact the hospital myself to find out	**Never**
The patient's relatives tell me	**Occasionally**
The patient tells me after hospital discharge	**Never**

Page 6

Q6 If you received information about your patient's stay in ICU, how often would it include details about the following aspects of their critical illness?

Shock	**Rarely**
Respiratory failure and mechanical ventilation	**Rarely**
Acute kidney injury requiring acute dialysis	**Rarely**
Acute encephalopathy / Delirium	**Never**
ARDS (acute respiratory distress syndrome)	**Rarely**
Neuromuscular weakness	**Never**
Tracheostomy insertion	**Never**
The duration of patient's stay in ICU	**Occasionally**

Page 7

Q7 If, during a recent hospital stay, your patient was in ICU, would you record this ICU admission in the medical/surgical history section of their notes?

Yes

Page 8

Q8 You receive a discharge summary in the post about your patient who was recently discharged from hospital after a severe illness. The summary confirms that they were in ICU during the hospital stay. Please comment on the following statements about the patient's follow-up care:

Because of the patient's ICU admission, I would make contact with them, even if the discharge summary did not request specific follow-up	**Agree**
If the patient did not self-present to my surgery for follow-up, the ICU admission would prompt me to schedule a consultation with them	**Agree**
If the patient did not self-present to my surgery for follow-up, the ICU admission would prompt me to schedule a consultation with the patient and a close relative	**Agree**

Page 9

Q9 Have you ever attended an educational meeting at which you learned about the long term complications of critical illness?

No

Page 10

Q10 Are you aware of any published Guidelines about the rehabilitation of patients following hospital discharge after ICU admission with critical illness?

No

Page 11

Q11 Do you think it would benefit your patient and/or their family members if you received details about their ICU admission?

Yes

#23

COMPLETE

Collector: Web Link 1 (Web Link)
Started: Monday, June 27, 2016 10:10:50 PM
Last Modified: Monday, June 27, 2016 10:14:09 PM
Time Spent: 00:03:19
IP Address: 86.45.87.53

Page 1

Q1 Which of the following best describes your GP practice?

Urban practice (Dublin, Cork, Galway, Limerick)

Page 2

Q2 Please indicate how long you have been working as a general practitioner.

<10years

Page 3

Q3 In which county do you conduct most of your GP work?

Dublin

Page 4

Q4 Please comment on the following statements regarding communication you receive after your patients are discharged from hospital.

I receive details of the patient’s admission	**Often**
I receive details of their admission within 30days of patient’s discharge	**Often**
The details I receive include whether patient was admitted to the ICU or not	**Occasionally**

Page 5

Q5 If you receive information about your patient’s ICU stay, by which method(s) would you receive this information?

The ICU/anaesthetic staff phone me directly	**Never**
In a discharge summary or letter from ICU medical team	**Never**
In a discharge summary or letter from other non-ICU medical/surgical team	**Always**
I contact the hospital myself to find out	**Rarely**
The patient’s relatives tell me	**Often**
The patient tells me after hospital discharge	**Often**

Page 6

Q6 If you received information about your patient’s stay in ICU, how often would it include details about the following aspects of their critical illness?

Shock	**Rarely**
Respiratory failure and mechanical ventilation	**Often**
Acute kidney injury requiring acute dialysis	**Often**
Acute encephalopathy / Delirium	**Occasionally**
ARDS (acute respiratory distress syndrome)	**Often**
Neuromuscular weakness	**Occasionally**
Tracheostomy insertion	**Occasionally**
The duration of patient’s stay in ICU	**Occasionally**

Page 7

Q7 If, during a recent hospital stay, your patient was in ICU, would you record this ICU admission in the medical/surgical history section of their notes? **Yes**

Page 8

Q8 You receive a discharge summary in the post about your patient who was recently discharged from hospital after a severe illness. The summary confirms that they were in ICU during the hospital stay. Please comment on the following statements about the patient's follow-up care:

Because of the patient's ICU admission, I would make contact with them, even if the discharge summary did not request specific follow-up	**Neutral**
If the patient did not self-present to my surgery for follow-up, the ICU admission would prompt me to schedule a consultation with them	**Neutral**
If the patient did not self-present to my surgery for follow-up, the ICU admission would prompt me to schedule a consultation with the patient and a close relative	**Neutral**

Page 9

Q9 Have you ever attended an educational meeting at which you learned about the long term complications of critical illness?

No

Page 10

Q10 Are you aware of any published Guidelines about the rehabilitation of patients following hospital discharge after ICU admission with critical illness?

No

Page 11

Q11 Do you think it would benefit your patient and/or their family members if you received details about their ICU admission?

Yes

#24

COMPLETE

Collector: Web Link 1 (Web Link)
Started: Tuesday, June 28, 2016 7:43:03 AM
Last Modified: Tuesday, June 28, 2016 7:46:45 AM
Time Spent: 00:03:42
IP Address: 86.40.16.32

Page 1

Q1 Which of the following best describes your GP practice?

Urban practice (Dublin, Cork, Galway, Limerick)

Page 2

Q2 Please indicate how long you have been working as a general practitioner.

<10years

Page 3

Q3 In which county do you conduct most of your GP work?

Dublin

Page 4

Q4 Please comment on the following statements regarding communication you receive after your patients are discharged from hospital.

I receive details of the patient's admission	**Always**
I receive details of their admission within 30days of patient's discharge	**Often**
The details I receive include whether patient was admitted to the ICU or not	**Occasionally**

Page 5

Q5 If you receive information about your patient's ICU stay, by which method(s) would you receive this information?

The ICU/anaesthetic staff phone me directly	**Never**
In a discharge summary or letter from ICU medical team	**Never**
In a discharge summary or letter from other non-ICU medical/surgical team	**Occasionally**
I contact the hospital myself to find out	**Never**
The patient's relatives tell me	**Often**
The patient tells me after hospital discharge	**Often**

Page 6

Q6 If you received information about your patient's stay in ICU, how often would it include details about the following aspects of their critical illness?

Shock	**Often**
Respiratory failure and mechanical ventilation	**Often**
Acute kidney injury requiring acute dialysis	**Often**
Acute encephalopathy / Delirium	**Often**
ARDS (acute respiratory distress syndrome)	**Often**
Neuromuscular weakness	**Rarely**
Tracheostomy insertion	**Often**
The duration of patient's stay in ICU	**Rarely**

Page 7

Q7 If, during a recent hospital stay, your patient was in ICU, would you record this ICU admission in the medical/surgical history section of their notes?

No

Page 8

Q8 You receive a discharge summary in the post about your patient who was recently discharged from hospital after a severe illness. The summary confirms that they were in ICU during the hospital stay. Please comment on the following statements about the patient's follow-up care:

Because of the patient's ICU admission, I would make contact with them, even if the discharge summary did not request specific follow-up	**Strongly disagree**
If the patient did not self-present to my surgery for follow-up, the ICU admission would prompt me to schedule a consultation with them	**Disagree**
If the patient did not self-present to my surgery for follow-up, the ICU admission would prompt me to schedule a consultation with the patient and a close relative	**Disagree**

Page 9

Q9 Have you ever attended an educational meeting at which you learned about the long term complications of critical illness? — **No**

Page 10

Q10 Are you aware of any published Guidelines about the rehabilitation of patients following hospital discharge after ICU admission with critical illness? — **No**

Page 11

Q11 Do you think it would benefit your patient and/or their family members if you received details about their ICU admission? — **Yes**

#25

COMPLETE

Collector: Web Link 1 (Web Link)
Started: Tuesday, June 28, 2016 3:09:26 PM
Last Modified: Tuesday, June 28, 2016 3:14:34 PM
Time Spent: 00:05:08
IP Address: 93.89.240.73

Page 1

Q1 Which of the following best describes your GP practice?

Rural practice (outside city)

Page 2

Q2 Please indicate how long you have been working as a general practitioner.

<10years

Page 3

Q3 In which county do you conduct most of your GP work?

Clare

Page 4

Q4 Please comment on the following statements regarding communication you receive after your patients are discharged from hospital.

I receive details of the patient’s admission	**Often**
I receive details of their admission within 30days of patient’s discharge	**Often**
The details I receive include whether patient was admitted to the ICU or not	**Always**

Page 5

Q5 If you receive information about your patient's ICU stay, by which method(s) would you receive this information?

The ICU/anaesthetic staff phone me directly	**Never**
In a discharge summary or letter from ICU medical team	**Never**
In a discharge summary or letter from other non-ICU medical/surgical team	**Often**
I contact the hospital myself to find out	**Often**
The patient's relatives tell me	**Always**
The patient tells me after hospital discharge	**Often**

Page 6

Q6 If you received information about your patient's stay in ICU, how often would it include details about the following aspects of their critical illness?

Shock	**Rarely**
Respiratory failure and mechanical ventilation	**Occasionally**
Acute kidney injury requiring acute dialysis	**Occasionally**
Acute encephalopathy / Delirium	**Occasionally**
ARDS (acute respiratory distress syndrome)	**Occasionally**
Neuromuscular weakness	**Rarely**
Tracheostomy insertion	**Occasionally**
The duration of patient's stay in ICU	**Rarely**

Page 7

Q7 If, during a recent hospital stay, your patient was in ICU, would you record this ICU admission in the medical/surgical history section of their notes?

No

Page 8

Q8 You receive a discharge summary in the post about your patient who was recently discharged from hospital after a severe illness. The summary confirms that they were in ICU during the hospital stay. Please comment on the following statements about the patient's follow-up care:

Because of the patient's ICU admission, I would make contact with them, even if the discharge summary did not request specific follow-up	**Agree**
If the patient did not self-present to my surgery for follow-up, the ICU admission would prompt me to schedule a consultation with them	**Neutral**
If the patient did not self-present to my surgery for follow-up, the ICU admission would prompt me to schedule a consultation with the patient and a close relative	**Neutral**

Page 9

Q9 Have you ever attended an educational meeting at which you learned about the long term complications of critical illness?

Yes,
If Yes, what was the event? If No, would you attend one if it was available?:
during my training asAneasthetic SHO and Registrar we had ongoing lectures/educational meetings and the topic was covered once

Page 10

Q10 Are you aware of any published Guidelines about the rehabilitation of patients following hospital discharge after ICU admission with critical illness?

No

Page 11

Q11 Do you think it would benefit your patient and/or their family members if you received details about their ICU admission?

Yes

#26

COMPLETE

Collector: Web Link 1 (Web Link)
Started: Tuesday, June 28, 2016 5:38:41 PM
Last Modified: Tuesday, June 28, 2016 5:46:30 PM
Time Spent: 00:07:49
IP Address: 78.17.119.31

Page 1

Q1 Which of the following best describes your GP practice?

Urban practice (Dublin, Cork, Galway, Limerick)

Page 2

Q2 Please indicate how long you have been working as a general practitioner.

10 years to 20 years

Page 3

Q3 In which county do you conduct most of your GP work?

Co.Dublin

Page 4

Q4 Please comment on the following statements regarding communication you receive after your patients are discharged from hospital.

I receive details of the patient's admission **Never**

I receive details of their admission within 30days of patient's discharge **Rarely**

The details I receive include whether patient was admitted to the ICU or not **Always**

Page 5

Q5 If you receive information about your patient's ICU stay, by which method(s) would you receive this information?

The ICU/anaesthetic staff phone me directly	**Occasionally**
In a discharge summary or letter from ICU medical team	**Often**
In a discharge summary or letter from other non-ICU medical/surgical team	**Often**
I contact the hospital myself to find out	**Always**
The patient's relatives tell me	**Often**
The patient tells me after hospital discharge	**Always**

Page 6

Q6 If you received information about your patient's stay in ICU, how often would it include details about the following aspects of their critical illness?

Shock	**Rarely**
Respiratory failure and mechanical ventilation	**Occasionally**
Acute kidney injury requiring acute dialysis	**Rarely**
Acute encephalopathy / Delirium	**Rarely**
ARDS (acute respiratory distress syndrome)	**Occasionally**
Neuromuscular weakness	**Often**
Tracheostomy insertion	**Never**
The duration of patient's stay in ICU	**Always**

Page 7

Q7 If, during a recent hospital stay, your patient was in ICU, would you record this ICU admission in the medical/surgical history section of their notes? **Yes**

Page 8

Q8 You receive a discharge summary in the post about your patient who was recently discharged from hospital after a severe illness. The summary confirms that they were in ICU during the hospital stay. Please comment on the following statements about the patient's follow-up care:

Because of the patient's ICU admission, I would make contact with them, even if the discharge summary did not request specific follow-up	**Agree**
If the patient did not self-present to my surgery for follow-up, the ICU admission would prompt me to schedule a consultation with them	**Strongly agree**
If the patient did not self-present to my surgery for follow-up, the ICU admission would prompt me to schedule a consultation with the patient and a close relative	**Neutral**

Page 9

Q9 Have you ever attended an educational meeting at which you learned about the long term complications of critical illness?

Yes

Page 10

Q10 Are you aware of any published Guidelines about the rehabilitation of patients following hospital discharge after ICU admission with critical illness?

Yes

Page 11

Q11 Do you think it would benefit your patient and/or their family members if you received details about their ICU admission?

Yes

#27

COMPLETE

Collector: Web Link 1 (Web Link)
Started: Tuesday, June 28, 2016 8:31:09 PM
Last Modified: Tuesday, June 28, 2016 8:35:00 PM
Time Spent: 00:03:51
IP Address: 51.171.103.162

Page 1

Q1 Which of the following best describes your GP practice?

Urban practice (Dublin, Cork, Galway, Limerick)

Page 2

Q2 Please indicate how long you have been working as a general practitioner.

10 years to 20 years

Page 3

Q3 In which county do you conduct most of your GP work?

Galway

Page 4

Q4 Please comment on the following statements regarding communication you receive after your patients are discharged from hospital.

I receive details of the patient’s admission	**Often**
I receive details of their admission within 30days of patient’s discharge	**Often**
The details I receive include whether patient was admitted to the ICU or not	**Occasionally**

Page 5

Q5 If you receive information about your patient's ICU stay, by which method(s) would you receive this information?

The ICU/anaesthetic staff phone me directly	**Never**
In a discharge summary or letter from ICU medical team	**Rarely**
In a discharge summary or letter from other non-ICU medical/surgical team	**Often**
I contact the hospital myself to find out	**Rarely**
The patient's relatives tell me	**Occasionally**
The patient tells me after hospital discharge	**Occasionally**

Page 6

Q6 If you received information about your patient's stay in ICU, how often would it include details about the following aspects of their critical illness?

Shock	**Often**
Respiratory failure and mechanical ventilation	**Often**
Acute kidney injury requiring acute dialysis	**Often**
Acute encephalopathy / Delirium	**Often**
ARDS (acute respiratory distress syndrome)	**Often**
Neuromuscular weakness	**Occasionally**
Tracheostomy insertion	**Often**
The duration of patient's stay in ICU	**Often**

Page 7

Q7 If, during a recent hospital stay, your patient was in ICU, would you record this ICU admission in the medical/surgical history section of their notes? **Yes**

Page 8

Q8 You receive a discharge summary in the post about your patient who was recently discharged from hospital after a severe illness. The summary confirms that they were in ICU during the hospital stay. Please comment on the following statements about the patient's follow-up care:

Because of the patient's ICU admission, I would make contact with them, even if the discharge summary did not request specific follow-up	**Neutral**
If the patient did not self-present to my surgery for follow-up, the ICU admission would prompt me to schedule a consultation with them	**Strongly disagree**
If the patient did not self-present to my surgery for follow-up, the ICU admission would prompt me to schedule a consultation with the patient and a close relative	**Neutral**

Page 9

Q9 Have you ever attended an educational meeting at which you learned about the long term complications of critical illness? **No**

Page 10

Q10 Are you aware of any published Guidelines about the rehabilitation of patients following hospital discharge after ICU admission with critical illness? **No**

Page 11

Q11 Do you think it would benefit your patient and/or their family members if you received details about their ICU admission? **Yes**

#28

COMPLETE

Collector: Web Link 1 (Web Link)
Started: Thursday, June 30, 2016 9:56:08 AM
Last Modified: Thursday, June 30, 2016 9:58:41 AM
Time Spent: 00:02:33
IP Address: 137.43.223.117

Page 1

Q1 Which of the following best describes your GP practice?

Urban practice (Dublin, Cork, Galway, Limerick)

Page 2

Q2 Please indicate how long you have been working as a general practitioner.

<10years

Page 3

Q3 In which county do you conduct most of your GP work?

Dublin

Page 4

Q4 Please comment on the following statements regarding communication you receive after your patients are discharged from hospital.

I receive details of the patient’s admission	**Often**
I receive details of their admission within 30days of patient’s discharge	**Often**
The details I receive include whether patient was admitted to the ICU or not	**Often**

Page 5

Q5 If you receive information about your patient's ICU stay, by which method(s) would you receive this information?

The ICU/anaesthetic staff phone me directly	**Never**
In a discharge summary or letter from ICU medical team	**Never**
In a discharge summary or letter from other non-ICU medical/surgical team	**Always**
I contact the hospital myself to find out	**Never**
The patient's relatives tell me	**Occasionally**
The patient tells me after hospital discharge	**Occasionally**

Page 6

Q6 If you received information about your patient's stay in ICU, how often would it include details about the following aspects of their critical illness?

Shock	**Occasionally**
Respiratory failure and mechanical ventilation	**Occasionally**
Acute kidney injury requiring acute dialysis	**Occasionally**
Acute encephalopathy / Delirium	**Rarely**
ARDS (acute respiratory distress syndrome)	**Rarely**
Neuromuscular weakness	**Rarely**
Tracheostomy insertion	**Occasionally**
The duration of patient's stay in ICU	**Occasionally**

Page 7

Q7 If, during a recent hospital stay, your patient was in ICU, would you record this ICU admission in the medical/surgical history section of their notes? **No**

Page 8

Q8 You receive a discharge summary in the post about your patient who was recently discharged from hospital after a severe illness. The summary confirms that they were in ICU during the hospital stay. Please comment on the following statements about the patient’s follow-up care:

Because of the patient’s ICU admission, I would make contact with them, even if the discharge summary did not request specific follow-up	**Neutral**
If the patient did not self-present to my surgery for follow-up, the ICU admission would prompt me to schedule a consultation with them	**Neutral**
If the patient did not self-present to my surgery for follow-up, the ICU admission would prompt me to schedule a consultation with the patient and a close relative	**Neutral**

Page 9

Q9 Have you ever attended an educational meeting at which you learned about the long term complications of critical illness?

No

Page 10

Q10 Are you aware of any published Guidelines about the rehabilitation of patients following hospital discharge after ICU admission with critical illness?

No

Page 11

Q11 Do you think it would benefit your patient and/or their family members if you received details about their ICU admission?

Yes

#29

COMPLETE

Collector: Web Link 1 (Web Link)
Started: Saturday, July 02, 2016 7:21:41 AM
Last Modified: Saturday, July 02, 2016 7:26:47 AM
Time Spent: 00:05:06
IP Address: 86.44.27.52

Page 1

Q1 Which of the following best describes your GP practice?

Urban practice (Dublin, Cork, Galway, Limerick)

Page 2

Q2 Please indicate how long you have been working as a general practitioner.

>20years

Page 3

Q3 In which county do you conduct most of your GP work?

Dublin

Page 4

Q4 Please comment on the following statements regarding communication you receive after your patients are discharged from hospital.

I receive details of the patient’s admission	**Often**
I receive details of their admission within 30days of patient’s discharge	**Often**
The details I receive include whether patient was admitted to the ICU or not	**Rarely**

Page 5

Q5 If you receive information about your patient’s ICU stay, by which method(s) would you receive this information?

The ICU/anaesthetic staff phone me directly	**Never**
In a discharge summary or letter from ICU medical team	**Never**
In a discharge summary or letter from other non-ICU medical/surgical team	**Often**
I contact the hospital myself to find out	**Never**
The patient’s relatives tell me	**Occasionally**
The patient tells me after hospital discharge	**Occasionally**

Page 6

Q6 If you received information about your patient’s stay in ICU, how often would it include details about the following aspects of their critical illness?

Shock	**Occasionally**
Respiratory failure and mechanical ventilation	**Often**
Acute kidney injury requiring acute dialysis	**Often**
Acute encephalopathy / Delirium	**Often**
ARDS (acute respiratory distress syndrome)	**Rarely**
Neuromuscular weakness	**Rarely**
Tracheostomy insertion	**Often**
The duration of patient’s stay in ICU	**Never**

Page 7

Q7 If, during a recent hospital stay, your patient was in ICU, would you record this ICU admission in the medical/surgical history section of their notes?

No

Page 8

Q8 You receive a discharge summary in the post about your patient who was recently discharged from hospital after a severe illness. The summary confirms that they were in ICU during the hospital stay. Please comment on the following statements about the patient's follow-up care:

Because of the patient's ICU admission, I would make contact with them, even if the discharge summary did not request specific follow-up	**Disagree**
If the patient did not self-present to my surgery for follow-up, the ICU admission would prompt me to schedule a consultation with them	**Disagree**
If the patient did not self-present to my surgery for follow-up, the ICU admission would prompt me to schedule a consultation with the patient and a close relative	**Disagree**

Page 9

Q9 Have you ever attended an educational meeting at which you learned about the long term complications of critical illness?

No,
If Yes, what was the event? If No, would you attend one if it was available?:
Maybe

Page 10

Q10 Are you aware of any published Guidelines about the rehabilitation of patients following hospital discharge after ICU admission with critical illness?

No

Page 11

Q11 Do you think it would benefit your patient and/or their family members if you received details about their ICU admission?

Yes,
If Yes, what the possible benefits?:
Better management of physical/psychological problems

#30

COMPLETE

Collector: Web Link 1 (Web Link)
Started: Friday, July 08, 2016 2:32:01 PM
Last Modified: Friday, July 08, 2016 2:35:06 PM
Time Spent: 00:03:05
IP Address: 185.11.67.132

Page 1

Q1 Which of the following best describes your GP practice?

Rural practice (outside city)

Page 2

Q2 Please indicate how long you have been working as a general practitioner.

10 years to 20 years

Page 3

Q3 In which county do you conduct most of your GP work?

Limerick

Page 4

Q4 Please comment on the following statements regarding communication you receive after your patients are discharged from hospital.

I receive details of the patient's admission	**Often**
I receive details of their admission within 30days of patient's discharge	**Often**
The details I receive include whether patient was admitted to the ICU or not	**Occasionally**

Page 5

Q5 If you receive information about your patient's ICU stay, by which method(s) would you receive this information?

The ICU/anaesthetic staff phone me directly	**Rarely**
In a discharge summary or letter from ICU medical team	**Rarely**
In a discharge summary or letter from other non-ICU medical/surgical team	**Occasionally**
I contact the hospital myself to find out	**Rarely**
The patient's relatives tell me	**Occasionally**
The patient tells me after hospital discharge	**Occasionally**

Page 6

Q6 If you received information about your patient's stay in ICU, how often would it include details about the following aspects of their critical illness?

Shock	**Rarely**
Respiratory failure and mechanical ventilation	**Occasionally**
Acute kidney injury requiring acute dialysis	**Often**
Acute encephalopathy / Delirium	**Often**
ARDS (acute respiratory distress syndrome)	**Occasionally**
Neuromuscular weakness	**Occasionally**
Tracheostomy insertion	**Often**
The duration of patient's stay in ICU	**Rarely**

Page 7

Q7 If, during a recent hospital stay, your patient was in ICU, would you record this ICU admission in the medical/surgical history section of their notes?

Yes

Page 8

Q8 You receive a discharge summary in the post about your patient who was recently discharged from hospital after a severe illness. The summary confirms that they were in ICU during the hospital stay. Please comment on the following statements about the patient's follow-up care:

Because of the patient's ICU admission, I would make contact with them, even if the discharge summary did not request specific follow-up	**Neutral**
If the patient did not self-present to my surgery for follow-up, the ICU admission would prompt me to schedule a consultation with them	**Neutral**
If the patient did not self-present to my surgery for follow-up, the ICU admission would prompt me to schedule a consultation with the patient and a close relative	**Neutral**

Page 9

Q9 Have you ever attended an educational meeting at which you learned about the long term complications of critical illness?

No,
If Yes, what was the event? If No, would you attend one if it was available?:
yes

Page 10

Q10 Are you aware of any published Guidelines about the rehabilitation of patients following hospital discharge after ICU admission with critical illness?

No

Page 11

Q11 Do you think it would benefit your patient and/or their family members if you received details about their ICU admission?

Yes,
If Yes, what the possible benefits?:
it may prompt review and follow up

#31

COMPLETE

Collector: Web Link - Manual Entry 1 (Web Link)
Started: Tuesday, September 27, 2016 12:34:32 PM
Last Modified: Tuesday, September 27, 2016 12:42:08 PM
Time Spent: 00:07:36
IP Address: 86.145.61.199

Page 1

Q1 Which of the following best describes your GP practice?

Rural practice (outside city)

Page 2

Q2 Please indicate how long you have been working as a general practitioner.

>20years

Page 3

Q3 In which county do you conduct most of your GP work?

Wicklow

Page 4

Q4 Please comment on the following statements regarding communication you receive after your patients are discharged from hospital.

I receive details of their admission within 30days of patient's discharge	**Often**
The details I receive include whether patient was admitted to the ICU or not	**Occasionally**

Page 5

Q5 If you receive information about your patient’s ICU stay, by which method(s) would you receive this information?

The ICU/anaesthetic staff phone me directly	**Never**
In a discharge summary or letter from ICU medical team	**Never**
In a discharge summary or letter from other non-ICU medical/surgical team	**Often**
I contact the hospital myself to find out	**Rarely**
The patient’s relatives tell me	**Often**
The patient tells me after hospital discharge	**Often**

Page 6

Q6 If you received information about your patient’s stay in ICU, how often would it include details about the following aspects of their critical illness?

Shock	**Never**
Respiratory failure and mechanical ventilation	**Occasionally**
Acute kidney injury requiring acute dialysis	**Occasionally**
Acute encephalopathy / Delirium	**Occasionally**
ARDS (acute respiratory distress syndrome)	**Occasionally**
Neuromuscular weakness	**Occasionally**
Tracheostomy insertion	**Occasionally**
The duration of patient’s stay in ICU	**Occasionally**

Page 7

Q7 If, during a recent hospital stay, your patient was in ICU, would you record this ICU admission in the medical/surgical history section of their notes? **No**

Page 8

Q8 You receive a discharge summary in the post about your patient who was recently discharged from hospital after a severe illness. The summary confirms that they were in ICU during the hospital stay. Please comment on the following statements about the patient's follow-up care:

Because of the patient's ICU admission, I would make contact with them, even if the discharge summary did not request specific follow-up	**Agree**
If the patient did not self-present to my surgery for follow-up, the ICU admission would prompt me to schedule a consultation with them	**Disagree**
If the patient did not self-present to my surgery for follow-up, the ICU admission would prompt me to schedule a consultation with the patient and a close relative	**Disagree**

Page 9

Q9 Have you ever attended an educational meeting at which you learned about the long term complications of critical illness?

No

Page 10

Q10 Are you aware of any published Guidelines about the rehabilitation of patients following hospital discharge after ICU admission with critical illness?

No

Page 11

Q11 Do you think it would benefit your patient and/or their family members if you received details about their ICU admission?

Yes,
If Yes, what the possible benefits?:
I would know what they went through and what problems will anticipate

#32

COMPLETE

Collector: Web Link - Manual Entry 1 (Web Link)
Started: Tuesday, September 27, 2016 12:42:14 PM
Last Modified: Tuesday, September 27, 2016 12:45:51 PM
Time Spent: 00:03:37
IP Address: 86.145.61.199

Page 1

Q1 Which of the following best describes your GP practice?

Rural practice (outside city)

Page 2

Q2 Please indicate how long you have been working as a general practitioner.

>20years

Page 3

Q3 In which county do you conduct most of your GP work?

Tipperary

Page 4

Q4 Please comment on the following statements regarding communication you receive after your patients are discharged from hospital.

I receive details of the patient's admission	**Always**
I receive details of their admission within 30days of patient's discharge	**Often**
The details I receive include whether patient was admitted to the ICU or not	**Rarely**

Page 5

Q5 If you receive information about your patient's ICU stay, by which method(s) would you receive this information?

The ICU/anaesthetic staff phone me directly	**Never**
In a discharge summary or letter from ICU medical team	**Rarely**
In a discharge summary or letter from other non-ICU medical/surgical team	**Occasionally**
I contact the hospital myself to find out	**Rarely**
The patient's relatives tell me	**Often**
The patient tells me after hospital discharge	**Occasionally**

Page 6

Q6 If you received information about your patient's stay in ICU, how often would it include details about the following aspects of their critical illness?

Shock	**Rarely**
Respiratory failure and mechanical ventilation	**Often**
Acute kidney injury requiring acute dialysis	**Often**
Acute encephalopathy / Delirium	**Occasionally**
ARDS (acute respiratory distress syndrome)	**Rarely**
Neuromuscular weakness	**Rarely**
Tracheostomy insertion	**Rarely**
The duration of patient's stay in ICU	**Often**

Page 7

Q7 If, during a recent hospital stay, your patient was in ICU, would you record this ICU admission in the medical/surgical history section of their notes?

No

Page 8

Q8 You receive a discharge summary in the post about your patient who was recently discharged from hospital after a severe illness. The summary confirms that they were in ICU during the hospital stay. Please comment on the following statements about the patient's follow-up care:

Because of the patient's ICU admission, I would make contact with them, even if the discharge summary did not request specific follow-up	**Neutral**
If the patient did not self-present to my surgery for follow-up, the ICU admission would prompt me to schedule a consultation with them	**Agree**
If the patient did not self-present to my surgery for follow-up, the ICU admission would prompt me to schedule a consultation with the patient and a close relative	**Disagree**

Page 9

Q9 Have you ever attended an educational meeting at which you learned about the long term complications of critical illness?

No

Page 10

Q10 Are you aware of any published Guidelines about the rehabilitation of patients following hospital discharge after ICU admission with critical illness?

No

Page 11

Q11 Do you think it would benefit your patient and/or their family members if you received details about their ICU admission?

Yes,
If Yes, what the possible benefits?:
Critically ill patients and their family often have questions concerning prognosis, complications following an episode of critical illness

#33

COMPLETE

Collector: Web Link - Manual Entry 1 (Web Link)
Started: Tuesday, September 27, 2016 12:46:15 PM
Last Modified: Tuesday, September 27, 2016 12:47:43 PM
Time Spent: 00:01:28
IP Address: 86.145.61.199

Page 1

Q1 Which of the following best describes your GP practice?

Rural practice (outside city)

Page 2

Q2 Please indicate how long you have been working as a general practitioner.

<10years

Page 3

Q3 In which county do you conduct most of your GP work?

Monaghan

Page 4

Q4 Please comment on the following statements regarding communication you receive after your patients are discharged from hospital.

I receive details of the patient's admission	**Often**
I receive details of their admission within 30days of patient's discharge	**Often**
The details I receive include whether patient was admitted to the ICU or not	**Occasionally**

Page 5

Q5 If you receive information about your patient's ICU stay, by which method(s) would you receive this information?

The ICU/anaesthetic staff phone me directly	**Never**
In a discharge summary or letter from ICU medical team	**Never**
In a discharge summary or letter from other non-ICU medical/surgical team	**Often**
I contact the hospital myself to find out	**Never**
The patient's relatives tell me	**Often**
The patient tells me after hospital discharge	**Often**

Page 6

Q6 If you received information about your patient's stay in ICU, how often would it include details about the following aspects of their critical illness?

Shock	**Often**
Respiratory failure and mechanical ventilation	**Occasionally**
Acute kidney injury requiring acute dialysis	**Occasionally**
Acute encephalopathy / Delirium	**Occasionally**
ARDS (acute respiratory distress syndrome)	**Occasionally**
Neuromuscular weakness	**Never**
Tracheostomy insertion	**Never**
The duration of patient's stay in ICU	**Occasionally**

Page 7

Q7 If, during a recent hospital stay, your patient was in ICU, would you record this ICU admission in the medical/surgical history section of their notes?

Yes

Page 8

Q8 You receive a discharge summary in the post about your patient who was recently discharged from hospital after a severe illness. The summary confirms that they were in ICU during the hospital stay. Please comment on the following statements about the patient's follow-up care:

Because of the patient's ICU admission, I would make contact with them, even if the discharge summary did not request specific follow-up	**Strongly disagree**
If the patient did not self-present to my surgery for follow-up, the ICU admission would prompt me to schedule a consultation with them	**Strongly agree**
If the patient did not self-present to my surgery for follow-up, the ICU admission would prompt me to schedule a consultation with the patient and a close relative	**Neutral**

Page 9

Q9 Have you ever attended an educational meeting at which you learned about the long term complications of critical illness?

No

Page 10

Q10 Are you aware of any published Guidelines about the rehabilitation of patients following hospital discharge after ICU admission with critical illness?

No

Page 11

Q11 Do you think it would benefit your patient and/or their family members if you received details about their ICU admission?

Yes

#34

COMPLETE

Collector: Web Link - Manual Entry 1 (Web Link)
Started: Tuesday, September 27, 2016 12:47:48 PM
Last Modified: Tuesday, September 27, 2016 12:49:58 PM
Time Spent: 00:02:10
IP Address: 86.145.61.199

Page 1

Q1 Which of the following best describes your GP practice?

Urban practice (Dublin, Cork, Galway, Limerick)

Page 2

Q2 Please indicate how long you have been working as a general practitioner.

<10years

Page 3

Q3 In which county do you conduct most of your GP work?

Limerick

Page 4

Q4 Please comment on the following statements regarding communication you receive after your patients are discharged from hospital.

I receive details of the patient’s admission	**Often**
I receive details of their admission within 30days of patient’s discharge	**Often**
The details I receive include whether patient was admitted to the ICU or not	**Occasionally**

Page 5

Q5 If you receive information about your patient's ICU stay, by which method(s) would you receive this information?

The ICU/anaesthetic staff phone me directly	**Never**
In a discharge summary or letter from ICU medical team	**Never**
In a discharge summary or letter from other non-ICU medical/surgical team	**Often**
I contact the hospital myself to find out	**Rarely**
The patient's relatives tell me	**Occasionally**
The patient tells me after hospital discharge	**Occasionally**

Page 6

Q6 If you received information about your patient's stay in ICU, how often would it include details about the following aspects of their critical illness?

Shock	**Occasionally**
Respiratory failure and mechanical ventilation	**Occasionally**
Acute kidney injury requiring acute dialysis	**Occasionally**
Acute encephalopathy / Delirium	**Occasionally**
ARDS (acute respiratory distress syndrome)	**Occasionally**
Neuromuscular weakness	**Rarely**
Tracheostomy insertion	**Occasionally**
The duration of patient's stay in ICU	**Occasionally**

Page 7

Q7 If, during a recent hospital stay, your patient was in ICU, would you record this ICU admission in the medical/surgical history section of their notes? **Yes**

Page 8

Q8 You receive a discharge summary in the post about your patient who was recently discharged from hospital after a severe illness. The summary confirms that they were in ICU during the hospital stay. Please comment on the following statements about the patient's follow-up care:

Because of the patient's ICU admission, I would make contact with them, even if the discharge summary did not request specific follow-up	**Disagree**
If the patient did not self-present to my surgery for follow-up, the ICU admission would prompt me to schedule a consultation with them	**Neutral**
If the patient did not self-present to my surgery for follow-up, the ICU admission would prompt me to schedule a consultation with the patient and a close relative	**Disagree**

Page 9

Q9 Have you ever attended an educational meeting at which you learned about the long term complications of critical illness?

Yes

Page 10

Q10 Are you aware of any published Guidelines about the rehabilitation of patients following hospital discharge after ICU admission with critical illness?

No

Page 11

Q11 Do you think it would benefit your patient and/or their family members if you received details about their ICU admission?

Yes

#35

COMPLETE

Collector: Web Link - Manual Entry 1 (Web Link)
Started: Tuesday, September 27, 2016 12:50:00 PM
Last Modified: Tuesday, September 27, 2016 12:52:13 PM
Time Spent: 00:02:13
IP Address: 86.145.61.199

Page 1

Q1 Which of the following best describes your GP practice?

Urban practice (Dublin, Cork, Galway, Limerick)

Page 2

Q2 Please indicate how long you have been working as a general practitioner.

<10years

Page 3

Q3 In which county do you conduct most of your GP work?

Cork

Page 4

Q4 Please comment on the following statements regarding communication you receive after your patients are discharged from hospital.

I receive details of the patient’s admission	**Often**
I receive details of their admission within 30days of patient’s discharge	**Often**
The details I receive include whether patient was admitted to the ICU or not	**Occasionally**

Page 5

Q5 If you receive information about your patient’s ICU stay, by which method(s) would you receive this information?

The ICU/anaesthetic staff phone me directly	**Never**
In a discharge summary or letter from ICU medical team	**Never**
In a discharge summary or letter from other non-ICU medical/surgical team	**Often**
I contact the hospital myself to find out	**Occasionally**
The patient’s relatives tell me	**Always**
The patient tells me after hospital discharge	**Always**

Page 6

Q6 If you received information about your patient’s stay in ICU, how often would it include details about the following aspects of their critical illness?

Shock	**Never**
Respiratory failure and mechanical ventilation	**Occasionally**
Acute kidney injury requiring acute dialysis	**Occasionally**
Acute encephalopathy / Delirium	**Never**
ARDS (acute respiratory distress syndrome)	**Never**
Neuromuscular weakness	**Never**
Tracheostomy insertion	**Never**
The duration of patient’s stay in ICU	**Never**

Page 7

Q7 If, during a recent hospital stay, your patient was in ICU, would you record this ICU admission in the medical/surgical history section of their notes? **Yes**

Page 8

Q8 You receive a discharge summary in the post about your patient who was recently discharged from hospital after a severe illness. The summary confirms that they were in ICU during the hospital stay. Please comment on the following statements about the patient's follow-up care:

Because of the patient's ICU admission, I would make contact with them, even if the discharge summary did not request specific follow-up	**Strongly disagree**
If the patient did not self-present to my surgery for follow-up, the ICU admission would prompt me to schedule a consultation with them	**Strongly agree**
If the patient did not self-present to my surgery for follow-up, the ICU admission would prompt me to schedule a consultation with the patient and a close relative	**Agree**

Page 9

Q9 Have you ever attended an educational meeting at which you learned about the long term complications of critical illness?

No,
If Yes, what was the event? If No, would you attend one if it was available?:
I would
attend

Page 10

Q10 Are you aware of any published Guidelines about the rehabilitation of patients following hospital discharge after ICU admission with critical illness?

No

Page 11

Q11 Do you think it would benefit your patient and/or their family members if you received details about their ICU admission?

Yes,
If Yes, what the possible
benefits?:
great understanding and forward planning, eg increasing support..etc

#36

COMPLETE

Collector: Web Link - Manual Entry 1 (Web Link)
Started: Tuesday, September 27, 2016 12:52:44 PM
Last Modified: Tuesday, September 27, 2016 12:54:15 PM
Time Spent: 00:01:31
IP Address: 86.145.61.199

Page 1

Q1 Which of the following best describes your GP practice?

Rural practice (outside city)

Page 2

Q2 Please indicate how long you have been working as a general practitioner.

<10years

Page 3

Q3 In which county do you conduct most of your GP work?

Cork

Page 4

Q4 Please comment on the following statements regarding communication you receive after your patients are discharged from hospital.

I receive details of the patient’s admission	**Always**
I receive details of their admission within 30days of patient’s discharge	**Occasionally**
The details I receive include whether patient was admitted to the ICU or not	**Often**

Page 5

Q5 If you receive information about your patient's ICU stay, by which method(s) would you receive this information?

The ICU/anaesthetic staff phone me directly	**Never**
In a discharge summary or letter from ICU medical team	**Never**
In a discharge summary or letter from other non-ICU medical/surgical team	**Occasionally**
I contact the hospital myself to find out	**Often**
The patient's relatives tell me	**Often**
The patient tells me after hospital discharge	**Often**

Page 6

Q6 If you received information about your patient's stay in ICU, how often would it include details about the following aspects of their critical illness?

Shock	**Occasionally**
Respiratory failure and mechanical ventilation	**Occasionally**
Acute kidney injury requiring acute dialysis	**Occasionally**
Acute encephalopathy / Delirium	**Rarely**
ARDS (acute respiratory distress syndrome)	**Occasionally**
Neuromuscular weakness	**Rarely**
Tracheostomy insertion	**Occasionally**
The duration of patient's stay in ICU	**Occasionally**

Page 7

Q7 If, during a recent hospital stay, your patient was in ICU, would you record this ICU admission in the medical/surgical history section of their notes?

No

Page 8

Q8 You receive a discharge summary in the post about your patient who was recently discharged from hospital after a severe illness. The summary confirms that they were in ICU during the hospital stay. Please comment on the following statements about the patient's follow-up care:

Because of the patient's ICU admission, I would make contact with them, even if the discharge summary did not request specific follow-up	**Disagree**
If the patient did not self-present to my surgery for follow-up, the ICU admission would prompt me to schedule a consultation with them	**Agree**
If the patient did not self-present to my surgery for follow-up, the ICU admission would prompt me to schedule a consultation with the patient and a close relative	**Neutral**

Page 9

Q9 Have you ever attended an educational meeting at which you learned about the long term complications of critical illness?

No

Page 10

Q10 Are you aware of any published Guidelines about the rehabilitation of patients following hospital discharge after ICU admission with critical illness?

No

Page 11

Q11 Do you think it would benefit your patient and/or their family members if you received details about their ICU admission?

Yes,
If Yes, what the possible benefits?:
absolutely

#37

COMPLETE

Collector: Web Link - Manual Entry 1 (Web Link)
Started: Tuesday, September 27, 2016 12:54:39 PM
Last Modified: Tuesday, September 27, 2016 12:55:44 PM
Time Spent: 00:01:05
IP Address: 86.145.61.199

Page 1

Q1 Which of the following best describes your GP practice?

Rural practice (outside city)

Page 2

Q2 Please indicate how long you have been working as a general practitioner.

10 years to 20 years

Page 3

Q3 In which county do you conduct most of your GP work?

cork

Page 4

Q4 Please comment on the following statements regarding communication you receive after your patients are discharged from hospital.

I receive details of the patient's admission	**Often**
I receive details of their admission within 30days of patient's discharge	**Often**
The details I receive include whether patient was admitted to the ICU or not	**Often**

Page 5

Q5 If you receive information about your patient’s ICU stay, by which method(s) would you receive this information?

The ICU/anaesthetic staff phone me directly	**Never**
In a discharge summary or letter from ICU medical team	**Never**
In a discharge summary or letter from other non-ICU medical/surgical team	**Never**
I contact the hospital myself to find out	**Occasionally**
The patient’s relatives tell me	**Often**
The patient tells me after hospital discharge	**Occasionally**

Page 6

Q6 If you received information about your patient’s stay in ICU, how often would it include details about the following aspects of their critical illness?

Shock	**Occasionally**
Respiratory failure and mechanical ventilation	**Occasionally**
Acute kidney injury requiring acute dialysis	**Occasionally**
Acute encephalopathy / Delirium	**Occasionally**
ARDS (acute respiratory distress syndrome)	**Occasionally**
Neuromuscular weakness	**Occasionally**
Tracheostomy insertion	**Occasionally**
The duration of patient’s stay in ICU	**Occasionally**

Page 7

Q7 If, during a recent hospital stay, your patient was in ICU, would you record this ICU admission in the medical/surgical history section of their notes? **Yes**

Page 8

Q8 You receive a discharge summary in the post about your patient who was recently discharged from hospital after a severe illness. The summary confirms that they were in ICU during the hospital stay. Please comment on the following statements about the patient's follow-up care:

Because of the patient's ICU admission, I would make contact with them, even if the discharge summary did not request specific follow-up	**Neutral**
If the patient did not self-present to my surgery for follow-up, the ICU admission would prompt me to schedule a consultation with them	**Neutral**
If the patient did not self-present to my surgery for follow-up, the ICU admission would prompt me to schedule a consultation with the patient and a close relative	**Agree**

Page 9

Q9 Have you ever attended an educational meeting at which you learned about the long term complications of critical illness?

No

Page 10

Q10 Are you aware of any published Guidelines about the rehabilitation of patients following hospital discharge after ICU admission with critical illness?

No

Page 11

Q11 Do you think it would benefit your patient and/or their family members if you received details about their ICU admission?

Yes

#38

COMPLETE

Collector: Web Link - Manual Entry 1 (Web Link)
Started: Tuesday, September 27, 2016 12:56:12 PM
Last Modified: Tuesday, September 27, 2016 12:58:40 PM
Time Spent: 00:02:28
IP Address: 86.145.61.199

Page 1

Q1 Which of the following best describes your GP practice?

Rural practice (outside city)

Page 2

Q2 Please indicate how long you have been working as a general practitioner.

>20years

Page 3

Q3 In which county do you conduct most of your GP work?

Clare

Page 4

Q4 Please comment on the following statements regarding communication you receive after your patients are discharged from hospital.

I receive details of the patient's admission	**Often**
I receive details of their admission within 30days of patient's discharge	**Often**
The details I receive include whether patient was admitted to the ICU or not	**Occasionally**

Page 5

Q5 If you receive information about your patient's ICU stay, by which method(s) would you receive this information?

The ICU/anaesthetic staff phone me directly	**Never**
In a discharge summary or letter from ICU medical team	**Rarely**
In a discharge summary or letter from other non-ICU medical/surgical team	**Often**
I contact the hospital myself to find out	**Never**
The patient's relatives tell me	**Occasionally**
The patient tells me after hospital discharge	**Occasionally**

Page 6

Q6 If you received information about your patient's stay in ICU, how often would it include details about the following aspects of their critical illness?

Shock	**Occasionally**
Respiratory failure and mechanical ventilation	**Occasionally**
Acute kidney injury requiring acute dialysis	**Occasionally**
Acute encephalopathy / Delirium	**Rarely**
ARDS (acute respiratory distress syndrome)	**Occasionally**
Neuromuscular weakness	**Rarely**
Tracheostomy insertion	**Occasionally**
The duration of patient's stay in ICU	**Occasionally**

Page 7

Q7 If, during a recent hospital stay, your patient was in ICU, would you record this ICU admission in the medical/surgical history section of their notes? **No**

Page 8

Q8 You receive a discharge summary in the post about your patient who was recently discharged from hospital after a severe illness. The summary confirms that they were in ICU during the hospital stay. Please comment on the following statements about the patient's follow-up care:

Because of the patient's ICU admission, I would make contact with them, even if the discharge summary did not request specific follow-up	**Neutral**
If the patient did not self-present to my surgery for follow-up, the ICU admission would prompt me to schedule a consultation with them	**Neutral**
If the patient did not self-present to my surgery for follow-up, the ICU admission would prompt me to schedule a consultation with the patient and a close relative	**Neutral**

Page 9

Q9 Have you ever attended an educational meeting at which you learned about the long term complications of critical illness?

No,
If Yes, what was the event? If No, would you attend one if it was available?:
yes

Page 10

Q10 Are you aware of any published Guidelines about the rehabilitation of patients following hospital discharge after ICU admission with critical illness?

No

Page 11

Q11 Do you think it would benefit your patient and/or their family members if you received details about their ICU admission?

Yes,
If Yes, what the possible benefits?:
Better insight into severity of illness and better management going forward

#39

COMPLETE

Collector: Web Link - Manual Entry 1 (Web Link)
Started: Tuesday, September 27, 2016 12:59:02 PM
Last Modified: Tuesday, September 27, 2016 1:00:14 PM
Time Spent: 00:01:12
IP Address: 86.145.61.199

Page 1

Q1 Which of the following best describes your GP practice?

Rural practice (outside city)

Page 2

Q2 Please indicate how long you have been working as a general practitioner.

10 years to 20 years

Page 3

Q3 In which county do you conduct most of your GP work?

Kerry

Page 4

Q4 Please comment on the following statements regarding communication you receive after your patients are discharged from hospital.

I receive details of the patient’s admission	**Often**
I receive details of their admission within 30days of patient’s discharge	**Often**
The details I receive include whether patient was admitted to the ICU or not	**Occasionally**

Page 5

Q5 If you receive information about your patient’s ICU stay, by which method(s) would you receive this information?

In a discharge summary or letter from ICU medical team	**Often**
The patient’s relatives tell me	**Often**

Page 6

Q6 If you received information about your patient’s stay in ICU, how often would it include details about the following aspects of their critical illness?

Shock	**Occasionally**
Respiratory failure and mechanical ventilation	**Often**
Acute kidney injury requiring acute dialysis	**Often**
Acute encephalopathy / Delirium	**Occasionally**
ARDS (acute respiratory distress syndrome)	**Rarely**
Neuromuscular weakness	**Occasionally**
Tracheostomy insertion	**Rarely**
The duration of patient’s stay in ICU	**Rarely**

Page 7

Q7 If, during a recent hospital stay, your patient was in ICU, would you record this ICU admission in the medical/surgical history section of their notes? **No**

Page 8

Q8 You receive a discharge summary in the post about your patient who was recently discharged from hospital after a severe illness. The summary confirms that they were in ICU during the hospital stay. Please comment on the following statements about the patient’s follow-up care:

Because of the patient’s ICU admission, I would make contact with them, even if the discharge summary did not request specific follow-up	**Agree**
If the patient did not self-present to my surgery for follow-up, the ICU admission would prompt me to schedule a consultation with them	**Disagree**
If the patient did not self-present to my surgery for follow-up, the ICU admission would prompt me to schedule a consultation with the patient and a close relative	**Disagree**

Page 9

Q9 Have you ever attended an educational meeting at which you learned about the long term complications of critical illness? **No**

Page 10

Q10 Are you aware of any published Guidelines about the rehabilitation of patients following hospital discharge after ICU admission with critical illness?

No

Page 11

Q11 Do you think it would benefit your patient and/or their family members if you received details about their ICU admission?

Yes

#40

COMPLETE

Collector: Web Link - Manual Entry 1 (Web Link)
Started: Tuesday, September 27, 2016 1:00:56 PM
Last Modified: Tuesday, September 27, 2016 1:10:34 PM
Time Spent: 00:09:38
IP Address: 86.145.61.199

Page 1

Q1 Which of the following best describes your GP practice?

Rural practice (outside city)

Page 2

Q2 Please indicate how long you have been working as a general practitioner.

<10years

Page 3

Q3 In which county do you conduct most of your GP work?

Leitrim

Page 4

Q4 Please comment on the following statements regarding communication you receive after your patients are discharged from hospital.

I receive details of the patient’s admission	**Always**
I receive details of their admission within 30days of patient’s discharge	**Often**
The details I receive include whether patient was admitted to the ICU or not	**Often**

Page 5

Q5 If you receive information about your patient's ICU stay, by which method(s) would you receive this information?

The ICU/anaesthetic staff phone me directly	**Rarely**
In a discharge summary or letter from ICU medical team	**Rarely**
In a discharge summary or letter from other non-ICU medical/surgical team	**Often**
I contact the hospital myself to find out	**Rarely**
The patient's relatives tell me	**Occasionally**
The patient tells me after hospital discharge	**Occasionally**

Page 6

Q6 If you received information about your patient's stay in ICU, how often would it include details about the following aspects of their critical illness?

Shock	**Often**
Respiratory failure and mechanical ventilation	**Often**
Acute kidney injury requiring acute dialysis	**Often**
Acute encephalopathy / Delirium	**Often**
Neuromuscular weakness	**Occasionally**
Tracheostomy insertion	**Often**
The duration of patient's stay in ICU	**Often**

Page 7

Q7 If, during a recent hospital stay, your patient was in ICU, would you record this ICU admission in the medical/surgical history section of their notes? **No**

Page 8

Q8 You receive a discharge summary in the post about your patient who was recently discharged from hospital after a severe illness. The summary confirms that they were in ICU during the hospital stay. Please comment on the following statements about the patient's follow-up care:

Because of the patient's ICU admission, I would make contact with them, even if the discharge summary did not request specific follow-up	**Agree**
If the patient did not self-present to my surgery for follow-up, the ICU admission would prompt me to schedule a consultation with them	**Strongly disagree**
If the patient did not self-present to my surgery for follow-up, the ICU admission would prompt me to schedule a consultation with the patient and a close relative	**Strongly disagree**

Page 9

Q9 Have you ever attended an educational meeting at which you learned about the long term complications of critical illness?

No

Page 10

Q10 Are you aware of any published Guidelines about the rehabilitation of patients following hospital discharge after ICU admission with critical illness?

No

Page 11

Q11 Do you think it would benefit your patient and/or their family members if you received details about their ICU admission?

Yes,
If Yes, what the possible benefits?:
not legible

#41

COMPLETE

Collector: Web Link - Manual Entry 1 (Web Link)
Started: Tuesday, September 27, 2016 1:11:01 PM
Last Modified: Tuesday, September 27, 2016 1:14:01 PM
Time Spent: 00:03:00
IP Address: 86.145.61.199

Page 1

Q1 Which of the following best describes your GP practice?

Urban practice (Dublin, Cork, Galway, Limerick)

Page 2

Q2 Please indicate how long you have been working as a general practitioner.

>20years

Page 3

Q3 In which county do you conduct most of your GP work?

Wexford

Page 4

Q4 Please comment on the following statements regarding communication you receive after your patients are discharged from hospital.

I receive details of the patient's admission	**Often**
I receive details of their admission within 30days of patient's discharge	**Rarely**
The details I receive include whether patient was admitted to the ICU or not	**Never**

Page 5

Q5 If you receive information about your patient's ICU stay, by which method(s) would you receive this information?

The ICU/anaesthetic staff phone me directly	**Never**
In a discharge summary or letter from ICU medical team	**Never**
In a discharge summary or letter from other non-ICU medical/surgical team	**Occasionally**
I contact the hospital myself to find out	**Often**
The patient's relatives tell me	**Often**
The patient tells me after hospital discharge	**Often**

Page 6

Q6 If you received information about your patient's stay in ICU, how often would it include details about the following aspects of their critical illness?

Shock	**Never**
Respiratory failure and mechanical ventilation	**Never**
Acute kidney injury requiring acute dialysis	**Never**
Acute encephalopathy / Delirium	**Never**
ARDS (acute respiratory distress syndrome)	**Never**
Neuromuscular weakness	**Never**
Tracheostomy insertion	**Often**
The duration of patient's stay in ICU	**Never**

Page 7

Q7 If, during a recent hospital stay, your patient was in ICU, would you record this ICU admission in the medical/surgical history section of their notes? **Yes**

Page 8

Q8 You receive a discharge summary in the post about your patient who was recently discharged from hospital after a severe illness. The summary confirms that they were in ICU during the hospital stay. Please comment on the following statements about the patient's follow-up care:

Because of the patient's ICU admission, I would make contact with them, even if the discharge summary did not request specific follow-up	**Strongly disagree**
If the patient did not self-present to my surgery for follow-up, the ICU admission would prompt me to schedule a consultation with them	**Strongly agree**
If the patient did not self-present to my surgery for follow-up, the ICU admission would prompt me to schedule a consultation with the patient and a close relative	**Strongly agree**

Page 9

Q9 Have you ever attended an educational meeting at which you learned about the long term complications of critical illness?

No

Page 10

Q10 Are you aware of any published Guidelines about the rehabilitation of patients following hospital discharge after ICU admission with critical illness?

No

Page 11

Q11 Do you think it would benefit your patient and/or their family members if you received details about their ICU admission?

Yes,
If Yes, what the possible benefits?:
complete medical notes facilitate the understanding of hospital care

#42

COMPLETE

Collector: Web Link - Manual Entry 1 (Web Link)
Started: Tuesday, September 27, 2016 1:14:22 PM
Last Modified: Tuesday, September 27, 2016 1:16:52 PM
Time Spent: 00:02:30
IP Address: 86.145.61.199

Page 1

Q1 Which of the following best describes your GP practice?

Rural practice (outside city)

Page 2

Q2 Please indicate how long you have been working as a general practitioner.

10 years to 20 years

Page 3

Q3 In which county do you conduct most of your GP work?

Wicklow

Page 4

Q4 Please comment on the following statements regarding communication you receive after your patients are discharged from hospital.

I receive details of the patient’s admission	**Rarely**
I receive details of their admission within 30days of patient’s discharge	**Rarely**
The details I receive include whether patient was admitted to the ICU or not	**Rarely**

Page 5

Q5 If you receive information about your patient's ICU stay, by which method(s) would you receive this information?

The ICU/anaesthetic staff phone me directly	**Rarely**
In a discharge summary or letter from ICU medical team	**Rarely**
In a discharge summary or letter from other non-ICU medical/surgical team	**Occasionally**
I contact the hospital myself to find out	**Occasionally**
The patient's relatives tell me	**Often**
The patient tells me after hospital discharge	**Often**

Page 6

Q6 If you received information about your patient's stay in ICU, how often would it include details about the following aspects of their critical illness?

Shock	**Rarely**
Respiratory failure and mechanical ventilation	**Rarely**
Acute kidney injury requiring acute dialysis	**Rarely**
Acute encephalopathy / Delirium	**Rarely**
ARDS (acute respiratory distress syndrome)	**Rarely**
Neuromuscular weakness	**Rarely**
Tracheostomy insertion	**Occasionally**
The duration of patient's stay in ICU	**Occasionally**

Page 7

Q7 If, during a recent hospital stay, your patient was in ICU, would you record this ICU admission in the medical/surgical history section of their notes? **Yes**

Page 8

Q8 You receive a discharge summary in the post about your patient who was recently discharged from hospital after a severe illness. The summary confirms that they were in ICU during the hospital stay. Please comment on the following statements about the patient's follow-up care:

Because of the patient's ICU admission, I would make contact with them, even if the discharge summary did not request specific follow-up	**Disagree**
If the patient did not self-present to my surgery for follow-up, the ICU admission would prompt me to schedule a consultation with them	**Neutral**
If the patient did not self-present to my surgery for follow-up, the ICU admission would prompt me to schedule a consultation with the patient and a close relative	**Neutral**

Page 9

Q9 Have you ever attended an educational meeting at which you learned about the long term complications of critical illness?

No

Page 10

Q10 Are you aware of any published Guidelines about the rehabilitation of patients following hospital discharge after ICU admission with critical illness?

No

Page 11

Q11 Do you think it would benefit your patient and/or their family members if you received details about their ICU admission?

Yes,
If Yes, what the possible benefits?:
We «50% of the time get d/c letters of any quality at all

#43

COMPLETE

Collector: Web Link - Manual Entry 1 (Web Link)
Started: Tuesday, September 27, 2016 1:17:11 PM
Last Modified: Tuesday, September 27, 2016 1:18:16 PM
Time Spent: 00:01:05
IP Address: 86.145.61.199

Page 1

Q1 Which of the following best describes your GP practice?

Rural practice (outside city)

Page 2

Q2 Please indicate how long you have been working as a general practitioner.

>20years

Page 3

Q3 In which county do you conduct most of your GP work?

-

Page 4

Q4 Please comment on the following statements regarding communication you receive after your patients are discharged from hospital.

I receive details of the patient's admission	**Always**
I receive details of their admission within 30days of patient's discharge	**Often**
The details I receive include whether patient was admitted to the ICU or not	**Often**

Page 5

Q5 If you receive information about your patient's ICU stay, by which method(s) would you receive this information?

The ICU/anaesthetic staff phone me directly	**Never**
In a discharge summary or letter from ICU medical team	**Never**
In a discharge summary or letter from other non-ICU medical/surgical team	**Often**
I contact the hospital myself to find out	**Rarely**
The patient's relatives tell me	**Often**
The patient tells me after hospital discharge	**Often**

Page 6

Q6 If you received information about your patient's stay in ICU, how often would it include details about the following aspects of their critical illness?

Shock	**Occasionally**
Respiratory failure and mechanical ventilation	**Often**
Acute kidney injury requiring acute dialysis	**Often**
Acute encephalopathy / Delirium	**Often**
ARDS (acute respiratory distress syndrome)	**Often**
Neuromuscular weakness	**Often**
Tracheostomy insertion	**Often**
The duration of patient's stay in ICU	**Often**

Page 7

Q7 If, during a recent hospital stay, your patient was in ICU, would you record this ICU admission in the medical/surgical history section of their notes? **Yes**

Page 8

Q8 You receive a discharge summary in the post about your patient who was recently discharged from hospital after a severe illness. The summary confirms that they were in ICU during the hospital stay. Please comment on the following statements about the patient's follow-up care:

Because of the patient's ICU admission, I would make contact with them, even if the discharge summary did not request specific follow-up	**Disagree**
If the patient did not self-present to my surgery for follow-up, the ICU admission would prompt me to schedule a consultation with them	**Disagree**
If the patient did not self-present to my surgery for follow-up, the ICU admission would prompt me to schedule a consultation with the patient and a close relative	**Disagree**

Page 9

Q9 Have you ever attended an educational meeting at which you learned about the long term complications of critical illness? **No**

Page 10

Q10 Are you aware of any published Guidelines about the rehabilitation of patients following hospital discharge after ICU admission with critical illness? **No**

Page 11

Q11 Do you think it would benefit your patient and/or their family members if you received details about their ICU admission? **Yes**

#44

COMPLETE

Collector: Web Link - Manual Entry 1 (Web Link)
Started: Tuesday, September 27, 2016 1:19:10 PM
Last Modified: Tuesday, September 27, 2016 1:20:37 PM
Time Spent: 00:01:27
IP Address: 86.145.61.199

Page 1

Q1 Which of the following best describes your GP practice?

Rural practice (outside city)

Page 2

Q2 Please indicate how long you have been working as a general practitioner.

10 years to 20 years

Page 3

Q3 In which county do you conduct most of your GP work?

Louth/Meath

Page 4

Q4 Please comment on the following statements regarding communication you receive after your patients are discharged from hospital.

I receive details of the patient’s admission	**Often**
I receive details of their admission within 30days of patient’s discharge	**Often**
The details I receive include whether patient was admitted to the ICU or not	**Occasionally**

Page 5

Q5 If you receive information about your patient's ICU stay, by which method(s) would you receive this information?

The ICU/anaesthetic staff phone me directly	**Never**
In a discharge summary or letter from ICU medical team	**Never**
In a discharge summary or letter from other non-ICU medical/surgical team	**Occasionally**
I contact the hospital myself to find out	**Never**
The patient's relatives tell me	**Often**
The patient tells me after hospital discharge	**Often**

Page 6

Q6 If you received information about your patient's stay in ICU, how often would it include details about the following aspects of their critical illness?

Shock	**Never**
Respiratory failure and mechanical ventilation	**Occasionally**
Acute kidney injury requiring acute dialysis	**Occasionally**
Acute encephalopathy / Delirium	**Occasionally**
ARDS (acute respiratory distress syndrome)	**Occasionally**
Neuromuscular weakness	**Occasionally**
Tracheostomy insertion	**Occasionally**
The duration of patient's stay in ICU	**Rarely**

Page 7

Q7 If, during a recent hospital stay, your patient was in ICU, would you record this ICU admission in the medical/surgical history section of their notes? **No**

Page 8

Q8 You receive a discharge summary in the post about your patient who was recently discharged from hospital after a severe illness. The summary confirms that they were in ICU during the hospital stay. Please comment on the following statements about the patient's follow-up care:

Because of the patient's ICU admission, I would make contact with them, even if the discharge summary did not request specific follow-up	**Agree**
If the patient did not self-present to my surgery for follow-up, the ICU admission would prompt me to schedule a consultation with them	**Disagree**
If the patient did not self-present to my surgery for follow-up, the ICU admission would prompt me to schedule a consultation with the patient and a close relative	**Disagree**

Page 9

Q9 Have you ever attended an educational meeting at which you learned about the long term complications of critical illness? **No**

Page 10

Q10 Are you aware of any published Guidelines about the rehabilitation of patients following hospital discharge after ICU admission with critical illness? **No**

Page 11

Q11 Do you think it would benefit your patient and/or their family members if you received details about their ICU admission? **Yes**

#45

COMPLETE

Collector: Web Link - Manual Entry 1 (Web Link)
Started: Tuesday, September 27, 2016 1:21:01 PM
Last Modified: Tuesday, September 27, 2016 1:22:20 PM
Time Spent: 00:01:19
IP Address: 86.145.61.199

Page 1

Q1 Which of the following best describes your GP practice?

Rural practice (outside city)

Page 2

Q2 Please indicate how long you have been working as a general practitioner.

<10years

Page 3

Q3 In which county do you conduct most of your GP work?

Meath

Page 4

Q4 Please comment on the following statements regarding communication you receive after your patients are discharged from hospital.

I receive details of the patient’s admission	**Often**
I receive details of their admission within 30days of patient’s discharge	**Often**
The details I receive include whether patient was admitted to the ICU or not	**Often**

Page 5

Q5 If you receive information about your patient's ICU stay, by which method(s) would you receive this information?

The ICU/anaesthetic staff phone me directly	**Never**
In a discharge summary or letter from ICU medical team	**Never**
In a discharge summary or letter from other non-ICU medical/surgical team	**Often**
I contact the hospital myself to find out	**Occasionally**
The patient's relatives tell me	**Often**
The patient tells me after hospital discharge	**Often**

Page 6

Q6 If you received information about your patient's stay in ICU, how often would it include details about the following aspects of their critical illness?

Shock	**Never**
Respiratory failure and mechanical ventilation	**Occasionally**
Acute kidney injury requiring acute dialysis	**Occasionally**
Acute encephalopathy / Delirium	**Never**
ARDS (acute respiratory distress syndrome)	**Never**
Neuromuscular weakness	**Never**
Tracheostomy insertion	**Never**
The duration of patient's stay in ICU	**Rarely**

Page 7

Q7 If, during a recent hospital stay, your patient was in ICU, would you record this ICU admission in the medical/surgical history section of their notes? **No**

Page 8

Q8 You receive a discharge summary in the post about your patient who was recently discharged from hospital after a severe illness. The summary confirms that they were in ICU during the hospital stay. Please comment on the following statements about the patient's follow-up care:

Because of the patient's ICU admission, I would make contact with them, even if the discharge summary did not request specific follow-up	**Disagree**
If the patient did not self-present to my surgery for follow-up, the ICU admission would prompt me to schedule a consultation with them	**Strongly disagree**
If the patient did not self-present to my surgery for follow-up, the ICU admission would prompt me to schedule a consultation with the patient and a close relative	**Strongly disagree**

Page 9

Q9 Have you ever attended an educational meeting at which you learned about the long term complications of critical illness?

No

Page 10

Q10 Are you aware of any published Guidelines about the rehabilitation of patients following hospital discharge after ICU admission with critical illness?

No

Page 11

Q11 Do you think it would benefit your patient and/or their family members if you received details about their ICU admission?

Yes

#46

COMPLETE

Collector: Web Link - Manual Entry 1 (Web Link)
Started: Tuesday, September 27, 2016 1:22:40 PM
Last Modified: Tuesday, September 27, 2016 1:24:47 PM
Time Spent: 00:02:07
IP Address: 86.145.61.199

Page 1

Q1 Which of the following best describes your GP practice?

Rural practice (outside city)

Page 2

Q2 Please indicate how long you have been working as a general practitioner.

>20years

Page 3

Q3 In which county do you conduct most of your GP work?

Louth

Page 4

Q4 Please comment on the following statements regarding communication you receive after your patients are discharged from hospital.

I receive details of the patient’s admission	**Often**
I receive details of their admission within 30days of patient’s discharge	**Often**
The details I receive include whether patient was admitted to the ICU or not	**Occasionally**

Page 5

Q5 If you receive information about your patient's ICU stay, by which method(s) would you receive this information?

The ICU/anaesthetic staff phone me directly	**Never**
In a discharge summary or letter from ICU medical team	**Never**
In a discharge summary or letter from other non-ICU medical/surgical team	**Often**
I contact the hospital myself to find out	**Never**
The patient's relatives tell me	**Often**
The patient tells me after hospital discharge	**Often**

Page 6

Q6 If you received information about your patient's stay in ICU, how often would it include details about the following aspects of their critical illness?

Shock	**Never**
Respiratory failure and mechanical ventilation	**Never**
Acute kidney injury requiring acute dialysis	**Never**
Acute encephalopathy / Delirium	**Never**
ARDS (acute respiratory distress syndrome)	**Never**
Neuromuscular weakness	**Never**
Tracheostomy insertion	**Never**
The duration of patient's stay in ICU	**Never**

Page 7

Q7 If, during a recent hospital stay, your patient was in ICU, would you record this ICU admission in the medical/surgical history section of their notes? **No**

Page 8

Q8 You receive a discharge summary in the post about your patient who was recently discharged from hospital after a severe illness. The summary confirms that they were in ICU during the hospital stay. Please comment on the following statements about the patient's follow-up care:

Because of the patient's ICU admission, I would make contact with them, even if the discharge summary did not request specific follow-up	**Agree**
If the patient did not self-present to my surgery for follow-up, the ICU admission would prompt me to schedule a consultation with them	**Disagree**
If the patient did not self-present to my surgery for follow-up, the ICU admission would prompt me to schedule a consultation with the patient and a close relative	**Disagree**

Page 9

Q9 Have you ever attended an educational meeting at which you learned about the long term complications of critical illness?

No

Page 10

Q10 Are you aware of any published Guidelines about the rehabilitation of patients following hospital discharge after ICU admission with critical illness?

No

Page 11

Q11 Do you think it would benefit your patient and/or their family members if you received details about their ICU admission?

Yes,
If Yes, what the possible benefits?:
more insight to the trauma they injured

#47

COMPLETE

Collector: Web Link - Manual Entry 1 (Web Link)
Started: Tuesday, September 27, 2016 1:58:38 PM
Last Modified: Tuesday, September 27, 2016 1:59:52 PM
Time Spent: 00:01:14
IP Address: 86.145.61.199

Page 1

Q1 Which of the following best describes your GP practice?

Rural practice (outside city)

Page 2

Q2 Please indicate how long you have been working as a general practitioner.

<10years

Page 3

Q3 In which county do you conduct most of your GP work?

Kildare

Page 4

Q4 Please comment on the following statements regarding communication you receive after your patients are discharged from hospital.

I receive details of the patient's admission	**Often**
I receive details of their admission within 30days of patient's discharge	**Occasionally**
The details I receive include whether patient was admitted to the ICU or not	**Rarely**

Page 5

Q5 If you receive information about your patient's ICU stay, by which method(s) would you receive this information?

The ICU/anaesthetic staff phone me directly	**Rarely**
In a discharge summary or letter from ICU medical team	**Rarely**
In a discharge summary or letter from other non-ICU medical/surgical team	**Occasionally**
I contact the hospital myself to find out	**Occasionally**
The patient's relatives tell me	**Occasionally**
The patient tells me after hospital discharge	**Often**

Page 6

Q6 If you received information about your patient's stay in ICU, how often would it include details about the following aspects of their critical illness?

Shock	**Rarely**
Respiratory failure and mechanical ventilation	**Rarely**
Acute kidney injury requiring acute dialysis	**Rarely**
Acute encephalopathy / Delirium	**Rarely**
ARDS (acute respiratory distress syndrome)	**Occasionally**
Neuromuscular weakness	**Occasionally**
Tracheostomy insertion	**Rarely**
The duration of patient's stay in ICU	**Rarely**

Page 7

Q7 If, during a recent hospital stay, your patient was in ICU, would you record this ICU admission in the medical/surgical history section of their notes? **Yes**

Page 8

Q8 You receive a discharge summary in the post about your patient who was recently discharged from hospital after a severe illness. The summary confirms that they were in ICU during the hospital stay. Please comment on the following statements about the patient’s follow-up care:

Because of the patient’s ICU admission, I would make contact with them, even if the discharge summary did not request specific follow-up	**Disagree**
If the patient did not self-present to my surgery for follow-up, the ICU admission would prompt me to schedule a consultation with them	**Agree**
If the patient did not self-present to my surgery for follow-up, the ICU admission would prompt me to schedule a consultation with the patient and a close relative	**Neutral**

Page 9

Q9 Have you ever attended an educational meeting at which you learned about the long term complications of critical illness?

No

Page 10

Q10 Are you aware of any published Guidelines about the rehabilitation of patients following hospital discharge after ICU admission with critical illness?

No

Page 11

Q11 Do you think it would benefit your patient and/or their family members if you received details about their ICU admission?

Yes

#48

COMPLETE

Collector: Web Link - Manual Entry 1 (Web Link)
Started: Tuesday, September 27, 2016 2:06:07 PM
Last Modified: Tuesday, September 27, 2016 2:07:00 PM
Time Spent: 00:00:53
IP Address: 86.145.61.199

Page 1

Q1 Which of the following best describes your GP practice?

Urban practice (Dublin, Cork, Galway, Limerick)

Page 2

Q2 Please indicate how long you have been working as a general practitioner.

>20years

Page 3

Q3 In which county do you conduct most of your GP work?

Dublin

Page 4

Q4 Please comment on the following statements regarding communication you receive after your patients are discharged from hospital.

The details I receive include whether patient was admitted to the ICU or not **Always**

Page 5

Q5 If you receive information about your patient's ICU stay, by which method(s) would you receive this information?

In a discharge summary or letter from other non-ICU medical/surgical team **Always**

Page 6

Q6 If you received information about your patient's stay in ICU, how often would it include details about the following aspects of their critical illness?

Shock	**Occasionally**
Respiratory failure and mechanical ventilation	**Occasionally**
Acute kidney injury requiring acute dialysis	**Occasionally**
Acute encephalopathy / Delirium	**Occasionally**
ARDS (acute respiratory distress syndrome)	**Occasionally**
Neuromuscular weakness	**Occasionally**
Tracheostomy insertion	**Occasionally**
The duration of patient's stay in ICU	**Occasionally**

Page 7

Q7 If, during a recent hospital stay, your patient was in ICU, would you record this ICU admission in the medical/surgical history section of their notes?

Yes

Page 8

Q8 You receive a discharge summary in the post about your patient who was recently discharged from hospital after a severe illness. The summary confirms that they were in ICU during the hospital stay. Please comment on the following statements about the patient's follow-up care:

Because of the patient's ICU admission, I would make contact with them, even if the discharge summary did not request specific follow-up	**Strongly agree**

Page 9

Q9 Have you ever attended an educational meeting at which you learned about the long term complications of critical illness?

No

Page 10

Q10 Are you aware of any published Guidelines about the rehabilitation of patients following hospital discharge after ICU admission with critical illness?

No

Page 11

**Q11** Do you think it would benefit your patient and/or their family members if you received details about their ICU admission?	**Yes**

#49

COMPLETE

Collector: Web Link - Manual Entry 1 (Web Link)
Started: Tuesday, September 27, 2016 2:07:20 PM
Last Modified: Tuesday, September 27, 2016 2:09:15 PM
Time Spent: 00:01:55
IP Address: 86.145.61.199

Page 1

Q1 Which of the following best describes your GP practice?

Urban practice (Dublin, Cork, Galway, Limerick)

Page 2

Q2 Please indicate how long you have been working as a general practitioner.

>20years

Page 3

Q3 In which county do you conduct most of your GP work?

Cork

Page 4

Q4 Please comment on the following statements regarding communication you receive after your patients are discharged from hospital.

I receive details of the patient's admission	**Often**
I receive details of their admission within 30days of patient's discharge	**Often**
The details I receive include whether patient was admitted to the ICU or not	**Often**

Page 5

Q5 If you receive information about your patient's ICU stay, by which method(s) would you receive this information?

The ICU/anaesthetic staff phone me directly	**Never**
In a discharge summary or letter from ICU medical team	**Never**
In a discharge summary or letter from other non-ICU medical/surgical team	**Often**
I contact the hospital myself to find out	**Never**
The patient's relatives tell me	**Often**
The patient tells me after hospital discharge	**Often**

Page 6

Q6 If you received information about your patient's stay in ICU, how often would it include details about the following aspects of their critical illness?

Shock	**Occasionally**
Respiratory failure and mechanical ventilation	**Often**
Acute kidney injury requiring acute dialysis	**Often**
Acute encephalopathy / Delirium	**Often**
ARDS (acute respiratory distress syndrome)	**Often**
Neuromuscular weakness	**Occasionally**
Tracheostomy insertion	**Often**
The duration of patient's stay in ICU	**Often**

Page 7

Q7 If, during a recent hospital stay, your patient was in ICU, would you record this ICU admission in the medical/surgical history section of their notes? **No**

Page 8

Q8 You receive a discharge summary in the post about your patient who was recently discharged from hospital after a severe illness. The summary confirms that they were in ICU during the hospital stay. Please comment on the following statements about the patient's follow-up care:

Because of the patient's ICU admission, I would make contact with them, even if the discharge summary did not request specific follow-up	**Neutral**
If the patient did not self-present to my surgery for follow-up, the ICU admission would prompt me to schedule a consultation with them	**Disagree**
If the patient did not self-present to my surgery for follow-up, the ICU admission would prompt me to schedule a consultation with the patient and a close relative	**Disagree**

Page 9

Q9 Have you ever attended an educational meeting at which you learned about the long term complications of critical illness?

No

Page 10

Q10 Are you aware of any published Guidelines about the rehabilitation of patients following hospital discharge after ICU admission with critical illness?

No

Page 11

Q11 Do you think it would benefit your patient and/or their family members if you received details about their ICU admission?

Yes,
If Yes, what the possible benefits?:
able to answer questions they have about their illness

#50

COMPLETE

Collector: Web Link - Manual Entry 1 (Web Link)
Started: Tuesday, September 27, 2016 2:09:36 PM
Last Modified: Tuesday, September 27, 2016 2:11:25 PM
Time Spent: 00:01:49
IP Address: 86.145.61.199

Page 1

Q1 Which of the following best describes your GP practice?

Urban practice (Dublin, Cork, Galway, Limerick)

Page 2

Q2 Please indicate how long you have been working as a general practitioner.

10 years to 20 years

Page 3

Q3 In which county do you conduct most of your GP work?

Cork

Page 4

Q4 Please comment on the following statements regarding communication you receive after your patients are discharged from hospital.

I receive details of the patient's admission	**Occasionally**
I receive details of their admission within 30days of patient's discharge	**Rarely**
The details I receive include whether patient was admitted to the ICU or not	**Never**

Page 5

Q5 If you receive information about your patient’s ICU stay, by which method(s) would you receive this information?

The ICU/anaesthetic staff phone me directly	**Never**
In a discharge summary or letter from ICU medical team	**Never**
In a discharge summary or letter from other non-ICU medical/surgical team	**Never**
I contact the hospital myself to find out	**Rarely**
The patient’s relatives tell me	**Often**
The patient tells me after hospital discharge	**Often**

Page 6

Q6 If you received information about your patient’s stay in ICU, how often would it include details about the following aspects of their critical illness?

Shock	**Occasionally**
Respiratory failure and mechanical ventilation	**Occasionally**
Acute kidney injury requiring acute dialysis	**Occasionally**
Acute encephalopathy / Delirium	**Occasionally**
ARDS (acute respiratory distress syndrome)	**Occasionally**
Neuromuscular weakness	**Occasionally**
Tracheostomy insertion	**Occasionally**
The duration of patient’s stay in ICU	**Occasionally**

Page 7

Q7 If, during a recent hospital stay, your patient was in ICU, would you record this ICU admission in the medical/surgical history section of their notes? **Yes**

Page 8

Q8 You receive a discharge summary in the post about your patient who was recently discharged from hospital after a severe illness. The summary confirms that they were in ICU during the hospital stay. Please comment on the following statements about the patient's follow-up care:

Because of the patient's ICU admission, I would make contact with them, even if the discharge summary did not request specific follow-up	**Strongly disagree**
If the patient did not self-present to my surgery for follow-up, the ICU admission would prompt me to schedule a consultation with them	**Strongly disagree**
If the patient did not self-present to my surgery for follow-up, the ICU admission would prompt me to schedule a consultation with the patient and a close relative	**Strongly disagree**

Page 9

Q9 Have you ever attended an educational meeting at which you learned about the long term complications of critical illness?

No

Page 10

Q10 Are you aware of any published Guidelines about the rehabilitation of patients following hospital discharge after ICU admission with critical illness?

No

Page 11

Q11 Do you think it would benefit your patient and/or their family members if you received details about their ICU admission?

Yes

#51

COMPLETE

Collector: Web Link - Manual Entry 1 (Web Link)
Started: Tuesday, September 27, 2016 2:11:47 PM
Last Modified: Tuesday, September 27, 2016 2:13:15 PM
Time Spent: 00:01:28
IP Address: 86.145.61.199

Page 1

Q1 Which of the following best describes your GP practice?

Rural practice (outside city)

Page 2

Q2 Please indicate how long you have been working as a general practitioner.

>20years

Page 3

Q3 In which county do you conduct most of your GP work?

Limerick

Page 4

Q4 Please comment on the following statements regarding communication you receive after your patients are discharged from hospital.

I receive details of the patient’s admission	**Often**
I receive details of their admission within 30days of patient’s discharge	**Occasionally**
The details I receive include whether patient was admitted to the ICU or not	**Occasionally**

Page 5

Q5 If you receive information about your patient’s ICU stay, by which method(s) would you receive this information?

The ICU/anaesthetic staff phone me directly	**Never**
In a discharge summary or letter from ICU medical team	**Never**
In a discharge summary or letter from other non-ICU medical/surgical team	**Often**
I contact the hospital myself to find out	**Occasionally**
The patient’s relatives tell me	**Often**
The patient tells me after hospital discharge	**Always**

Page 6

Q6 If you received information about your patient’s stay in ICU, how often would it include details about the following aspects of their critical illness?

Shock	**Never**
Respiratory failure and mechanical ventilation	**Occasionally**
Acute kidney injury requiring acute dialysis	**Often**
Acute encephalopathy / Delirium	**Rarely**
ARDS (acute respiratory distress syndrome)	**Rarely**
Neuromuscular weakness	**Never**
Tracheostomy insertion	**Rarely**
The duration of patient’s stay in ICU	**Occasionally**

Page 7

**Q7** If, during a recent hospital stay, your patient was in ICU, would you record this ICU admission in the medical/surgical history section of their notes?	**No**

Page 8

Q8 You receive a discharge summary in the post about your patient who was recently discharged from hospital after a severe illness. The summary confirms that they were in ICU during the hospital stay. Please comment on the following statements about the patient's follow-up care:

Because of the patient's ICU admission, I would make contact with them, even if the discharge summary did not request specific follow-up	**Disagree**
If the patient did not self-present to my surgery for follow-up, the ICU admission would prompt me to schedule a consultation with them	**Disagree**
If the patient did not self-present to my surgery for follow-up, the ICU admission would prompt me to schedule a consultation with the patient and a close relative	**Disagree**

Page 9

Q9 Have you ever attended an educational meeting at which you learned about the long term complications of critical illness?

No,
If Yes, what was the event? If No, would you attend one if it was available?:
yes

Page 10

Q10 Are you aware of any published Guidelines about the rehabilitation of patients following hospital discharge after ICU admission with critical illness?

No

Page 11

Q11 Do you think it would benefit your patient and/or their family members if you received details about their ICU admission?

Yes

#52

COMPLETE

Collector: Web Link - Manual Entry 1 (Web Link)
Started: Tuesday, September 27, 2016 2:14:02 PM
Last Modified: Tuesday, September 27, 2016 2:15:17 PM
Time Spent: 00:01:15
IP Address: 86.145.61.199

Page 1

Q1 Which of the following best describes your GP practice?

Rural practice (outside city)

Page 2

Q2 Please indicate how long you have been working as a general practitioner.

>20years

Page 3

Q3 In which county do you conduct most of your GP work?

Longford

Page 4

Q4 Please comment on the following statements regarding communication you receive after your patients are discharged from hospital.

I receive details of the patient’s admission	**Often**
I receive details of their admission within 30days of patient’s discharge	**Often**
The details I receive include whether patient was admitted to the ICU or not	**Rarely**

Page 5

Q5 If you receive information about your patient's ICU stay, by which method(s) would you receive this information?

The ICU/anaesthetic staff phone me directly	**Never**
In a discharge summary or letter from ICU medical team	**Rarely**
In a discharge summary or letter from other non-ICU medical/surgical team	**Rarely**
I contact the hospital myself to find out	**Never**
The patient's relatives tell me	**Occasionally**
The patient tells me after hospital discharge	**Occasionally**

Page 6

Q6 If you received information about your patient's stay in ICU, how often would it include details about the following aspects of their critical illness?

Shock	**Occasionally**
Respiratory failure and mechanical ventilation	**Occasionally**
Acute kidney injury requiring acute dialysis	**Occasionally**
Acute encephalopathy / Delirium	**Occasionally**
ARDS (acute respiratory distress syndrome)	**Occasionally**
Neuromuscular weakness	**Occasionally**
Tracheostomy insertion	**Occasionally**
The duration of patient's stay in ICU	**Occasionally**

Page 7

Q7 If, during a recent hospital stay, your patient was in ICU, would you record this ICU admission in the medical/surgical history section of their notes? **Yes**

Page 8

Q8 You receive a discharge summary in the post about your patient who was recently discharged from hospital after a severe illness. The summary confirms that they were in ICU during the hospital stay. Please comment on the following statements about the patient's follow-up care:

Because of the patient's ICU admission, I would make contact with them, even if the discharge summary did not request specific follow-up	**Disagree**
If the patient did not self-present to my surgery for follow-up, the ICU admission would prompt me to schedule a consultation with them	**Disagree**
If the patient did not self-present to my surgery for follow-up, the ICU admission would prompt me to schedule a consultation with the patient and a close relative	**Disagree**

Page 9

Q9 Have you ever attended an educational meeting at which you learned about the long term complications of critical illness?

No

Page 10

Q10 Are you aware of any published Guidelines about the rehabilitation of patients following hospital discharge after ICU admission with critical illness?

No

Page 11

Q11 Do you think it would benefit your patient and/or their family members if you received details about their ICU admission?

Yes

#53

COMPLETE

Collector: Web Link - Manual Entry 1 (Web Link)
Started: Tuesday, September 27, 2016 2:15:38 PM
Last Modified: Tuesday, September 27, 2016 2:16:43 PM
Time Spent: 00:01:05
IP Address: 86.145.61.199

Page 1

Q1 Which of the following best describes your GP practice?

Urban practice (Dublin, Cork, Galway, Limerick)

Page 2

Q2 Please indicate how long you have been working as a general practitioner.

>20years

Page 3

Q3 In which county do you conduct most of your GP work?

-

Page 4

Q4 Please comment on the following statements regarding communication you receive after your patients are discharged from hospital.

I receive details of the patient’s admission	**Often**
I receive details of their admission within 30days of patient’s discharge	**Often**
The details I receive include whether patient was admitted to the ICU or not	**Occasionally**

Page 5

Q5 If you receive information about your patient's ICU stay, by which method(s) would you receive this information?

The ICU/anaesthetic staff phone me directly	**Rarely**
In a discharge summary or letter from ICU medical team	**Never**
In a discharge summary or letter from other non-ICU medical/surgical team	**Rarely**
I contact the hospital myself to find out	**Often**
The patient's relatives tell me	**Often**
The patient tells me after hospital discharge	**Often**

Page 6

Q6 If you received information about your patient's stay in ICU, how often would it include details about the following aspects of their critical illness?

Shock	**Rarely**
Respiratory failure and mechanical ventilation	**Rarely**
Acute kidney injury requiring acute dialysis	**Rarely**
Acute encephalopathy / Delirium	**Rarely**
ARDS (acute respiratory distress syndrome)	**Rarely**
Neuromuscular weakness	**Never**
Tracheostomy insertion	**Never**
The duration of patient's stay in ICU	**Never**

Page 7

Q7 If, during a recent hospital stay, your patient was in ICU, would you record this ICU admission in the medical/surgical history section of their notes? **Yes**

Page 8

Q8 You receive a discharge summary in the post about your patient who was recently discharged from hospital after a severe illness. The summary confirms that they were in ICU during the hospital stay. Please comment on the following statements about the patient's follow-up care:

Because of the patient's ICU admission, I would make contact with them, even if the discharge summary did not request specific follow-up	**Disagree**
If the patient did not self-present to my surgery for follow-up, the ICU admission would prompt me to schedule a consultation with them	**Agree**
If the patient did not self-present to my surgery for follow-up, the ICU admission would prompt me to schedule a consultation with the patient and a close relative	**Agree**

Page 9

Q9 Have you ever attended an educational meeting at which you learned about the long term complications of critical illness?

Yes

Page 10

Q10 Are you aware of any published Guidelines about the rehabilitation of patients following hospital discharge after ICU admission with critical illness?

No

Page 11

Q11 Do you think it would benefit your patient and/or their family members if you received details about their ICU admission?

Yes

#54

COMPLETE

Collector: Web Link - Manual Entry 1 (Web Link)
Started: Tuesday, September 27, 2016 2:17:04 PM
Last Modified: Tuesday, September 27, 2016 2:18:13 PM
Time Spent: 00:01:09
IP Address: 86.145.61.199

Page 1

Q1 Which of the following best describes your GP practice?

Urban practice (Dublin, Cork, Galway, Limerick)

Page 2

Q2 Please indicate how long you have been working as a general practitioner.

10 years to 20 years

Page 3

Q3 In which county do you conduct most of your GP work?

Dublin

Page 4

Q4 Please comment on the following statements regarding communication you receive after your patients are discharged from hospital.

I receive details of the patient's admission	**Rarely**
I receive details of their admission within 30days of patient's discharge	**Occasionally**
The details I receive include whether patient was admitted to the ICU or not	**Occasionally**

Page 5

Q5 If you receive information about your patient's ICU stay, by which method(s) would you receive this information?

The ICU/anaesthetic staff phone me directly	**Never**
In a discharge summary or letter from ICU medical team	**Never**
In a discharge summary or letter from other non-ICU medical/surgical team	**Occasionally**
I contact the hospital myself to find out	**Rarely**
The patient's relatives tell me	**Occasionally**
The patient tells me after hospital discharge	**Often**

Page 6

Q6 If you received information about your patient's stay in ICU, how often would it include details about the following aspects of their critical illness?

Shock	**Never**
Respiratory failure and mechanical ventilation	**Never**
Acute kidney injury requiring acute dialysis	**Never**
Acute encephalopathy / Delirium	**Never**
ARDS (acute respiratory distress syndrome)	**Never**
Neuromuscular weakness	**Never**
Tracheostomy insertion	**Never**
The duration of patient's stay in ICU	**Never**

Page 7

**Q7** If, during a recent hospital stay, your patient was in ICU, would you record this ICU admission in the medical/surgical history section of their notes?	**Yes**

Page 8

Q8 You receive a discharge summary in the post about your patient who was recently discharged from hospital after a severe illness. The summary confirms that they were in ICU during the hospital stay. Please comment on the following statements about the patient's follow-up care:

Because of the patient's ICU admission, I would make contact with them, even if the discharge summary did not request specific follow-up	**Neutral**
If the patient did not self-present to my surgery for follow-up, the ICU admission would prompt me to schedule a consultation with them	**Agree**
If the patient did not self-present to my surgery for follow-up, the ICU admission would prompt me to schedule a consultation with the patient and a close relative	**Neutral**

Page 9

Q9 Have you ever attended an educational meeting at which you learned about the long term complications of critical illness?

No

Page 10

Q10 Are you aware of any published Guidelines about the rehabilitation of patients following hospital discharge after ICU admission with critical illness?

No

Page 11

Q11 Do you think it would benefit your patient and/or their family members if you received details about their ICU admission?

Yes

#55

COMPLETE

Collector: Web Link - Manual Entry 1 (Web Link)
Started: Tuesday, September 27, 2016 2:18:37 PM
Last Modified: Tuesday, September 27, 2016 2:20:04 PM
Time Spent: 00:01:27
IP Address: 86.145.61.199

Page 1

Q1 Which of the following best describes your GP practice?

Rural practice (outside city)

Page 2

Q2 Please indicate how long you have been working as a general practitioner.

>20years

Page 3

Q3 In which county do you conduct most of your GP work?

Kerry

Page 4

Q4 Please comment on the following statements regarding communication you receive after your patients are discharged from hospital.

I receive details of the patient’s admission	**Often**
I receive details of their admission within 30days of patient’s discharge	**Often**
The details I receive include whether patient was admitted to the ICU or not	**Rarely**

Page 5

Q5 If you receive information about your patient's ICU stay, by which method(s) would you receive this information?

The ICU/anaesthetic staff phone me directly	**Never**
In a discharge summary or letter from ICU medical team	**Never**
In a discharge summary or letter from other non-ICU medical/surgical team	**Rarely**
I contact the hospital myself to find out	**Rarely**
The patient's relatives tell me	**Rarely**
The patient tells me after hospital discharge	**Occasionally**

Page 6

Q6 If you received information about your patient's stay in ICU, how often would it include details about the following aspects of their critical illness?

Shock	**Rarely**
Respiratory failure and mechanical ventilation	**Rarely**
Acute kidney injury requiring acute dialysis	**Rarely**
Acute encephalopathy / Delirium	**Rarely**
ARDS (acute respiratory distress syndrome)	**Never**
Neuromuscular weakness	**Never**
Tracheostomy insertion	**Rarely**
The duration of patient's stay in ICU	**Rarely**

Page 7

Q7 If, during a recent hospital stay, your patient was in ICU, would you record this ICU admission in the medical/surgical history section of their notes? **No**

Page 8

Q8 You receive a discharge summary in the post about your patient who was recently discharged from hospital after a severe illness. The summary confirms that they were in ICU during the hospital stay. Please comment on the following statements about the patient's follow-up care:

Because of the patient's ICU admission, I would make contact with them, even if the discharge summary did not request specific follow-up	**Agree**
If the patient did not self-present to my surgery for follow-up, the ICU admission would prompt me to schedule a consultation with them	**Neutral**
If the patient did not self-present to my surgery for follow-up, the ICU admission would prompt me to schedule a consultation with the patient and a close relative	**Neutral**

Page 9

Q9 Have you ever attended an educational meeting at which you learned about the long term complications of critical illness?

No,
If Yes, what was the event? If No, would you attend one if it was available?:
yes

Page 10

Q10 Are you aware of any published Guidelines about the rehabilitation of patients following hospital discharge after ICU admission with critical illness?

No

Page 11

Q11 Do you think it would benefit your patient and/or their family members if you received details about their ICU admission?

Yes

#56

COMPLETE

Collector: Web Link - Manual Entry 1 (Web Link)
Started: Tuesday, September 27, 2016 2:20:22 PM
Last Modified: Tuesday, September 27, 2016 2:22:41 PM
Time Spent: 00:02:19
IP Address: 86.145.61.199

Page 1

Q1 Which of the following best describes your GP practice?

Urban practice (Dublin, Cork, Galway, Limerick)

Page 2

Q2 Please indicate how long you have been working as a general practitioner.

<10years

Page 3

Q3 In which county do you conduct most of your GP work?

Donegal

Page 4

Q4 Please comment on the following statements regarding communication you receive after your patients are discharged from hospital.

I receive details of their admission within 30days of patient’s discharge — **Always**

The details I receive include whether patient was admitted to the ICU or not — **Occasionally**

Page 5

Q5 If you receive information about your patient’s ICU stay, by which method(s) would you receive this information?

In a discharge summary or letter from other non-ICU medical/surgical team — **Always**

Page 6

Q6 If you received information about your patient’s stay in ICU, how often would it include details about the following aspects of their critical illness?

Shock	**Occasionally**
Respiratory failure and mechanical ventilation	**Occasionally**
Acute kidney injury requiring acute dialysis	**Occasionally**
Acute encephalopathy / Delirium	**Rarely**
ARDS (acute respiratory distress syndrome)	**Rarely**
Neuromuscular weakness	**Rarely**
Tracheostomy insertion	**Occasionally**
The duration of patient’s stay in ICU	**Often**

Page 7

Q7 If, during a recent hospital stay, your patient was in ICU, would you record this ICU admission in the medical/surgical history section of their notes?

No

Page 8

Q8 You receive a discharge summary in the post about your patient who was recently discharged from hospital after a severe illness. The summary confirms that they were in ICU during the hospital stay. Please comment on the following statements about the patient’s follow-up care:

Because of the patient’s ICU admission, I would make contact with them, even if the discharge summary did not request specific follow-up	**Neutral**
If the patient did not self-present to my surgery for follow-up, the ICU admission would prompt me to schedule a consultation with them	**Agree**
If the patient did not self-present to my surgery for follow-up, the ICU admission would prompt me to schedule a consultation with the patient and a close relative	**Disagree**

Page 9

Q9 Have you ever attended an educational meeting at which you learned about the long term complications of critical illness?

No

Page 10

Q10 Are you aware of any published Guidelines about the rehabilitation of patients following hospital discharge after ICU admission with critical illness?

No

Page 11

Q11 Do you think it would benefit your patient and/or their family members if you received details about their ICU admission?

Yes,
If Yes, what the possible benefits?:
assist the overall assessment and psychological input (short and long term)

#57

COMPLETE

Collector: Web Link - Manual Entry 1 (Web Link)
Started: Tuesday, September 27, 2016 2:23:00 PM
Last Modified: Tuesday, September 27, 2016 2:24:11 PM
Time Spent: 00:01:11
IP Address: 86.145.61.199

Page 1

Q1 Which of the following best describes your GP practice?

Rural practice (outside city)

Page 2

Q2 Please indicate how long you have been working as a general practitioner.

>20years

Page 3

Q3 In which county do you conduct most of your GP work?

Cork

Page 4

Q4 Please comment on the following statements regarding communication you receive after your patients are discharged from hospital.

I receive details of the patient’s admission	**Often**
I receive details of their admission within 30days of patient’s discharge	**Occasionally**
The details I receive include whether patient was admitted to the ICU or not	**Rarely**

Page 5

Q5 If you receive information about your patient’s ICU stay, by which method(s) would you receive this information?

The ICU/anaesthetic staff phone me directly	**Never**
In a discharge summary or letter from ICU medical team	**Never**
In a discharge summary or letter from other non-ICU medical/surgical team	**Rarely**
I contact the hospital myself to find out	**Rarely**
The patient’s relatives tell me	**Often**
The patient tells me after hospital discharge	**Often**

Page 6

Q6 If you received information about your patient’s stay in ICU, how often would it include details about the following aspects of their critical illness?

Shock	**Rarely**
Respiratory failure and mechanical ventilation	**Occasionally**
Acute kidney injury requiring acute dialysis	**Occasionally**
Acute encephalopathy / Delirium	**Never**
ARDS (acute respiratory distress syndrome)	**Rarely**
Neuromuscular weakness	**Never**
Tracheostomy insertion	**Rarely**
The duration of patient’s stay in ICU	**Rarely**

Page 7

Q7 If, during a recent hospital stay, your patient was in ICU, would you record this ICU admission in the medical/surgical history section of their notes? **No**

Page 8

Q8 You receive a discharge summary in the post about your patient who was recently discharged from hospital after a severe illness. The summary confirms that they were in ICU during the hospital stay. Please comment on the following statements about the patient's follow-up care:

Because of the patient's ICU admission, I would make contact with them, even if the discharge summary did not request specific follow-up	**Agree**
If the patient did not self-present to my surgery for follow-up, the ICU admission would prompt me to schedule a consultation with them	**Disagree**
If the patient did not self-present to my surgery for follow-up, the ICU admission would prompt me to schedule a consultation with the patient and a close relative	**Disagree**

Page 9

Q9 Have you ever attended an educational meeting at which you learned about the long term complications of critical illness?

No

Page 10

Q10 Are you aware of any published Guidelines about the rehabilitation of patients following hospital discharge after ICU admission with critical illness?

No

Page 11

Q11 Do you think it would benefit your patient and/or their family members if you received details about their ICU admission?

Yes

#58

COMPLETE

Collector: Web Link - Manual Entry 1 (Web Link)
Started: Tuesday, September 27, 2016 2:24:35 PM
Last Modified: Tuesday, September 27, 2016 2:25:46 PM
Time Spent: 00:01:11
IP Address: 86.145.61.199

Page 1

Q1 Which of the following best describes your GP practice?

Rural practice (outside city)

Page 2

Q2 Please indicate how long you have been working as a general practitioner.

10 years to 20 years

Page 3

Q3 In which county do you conduct most of your GP work?

Clare

Page 4

Q4 Please comment on the following statements regarding communication you receive after your patients are discharged from hospital.

I receive details of the patient’s admission	**Often**
I receive details of their admission within 30days of patient’s discharge	**Often**
The details I receive include whether patient was admitted to the ICU or not	**Occasionally**

Page 5

Q5 If you receive information about your patient's ICU stay, by which method(s) would you receive this information?

The ICU/anaesthetic staff phone me directly	**Rarely**
In a discharge summary or letter from ICU medical team	**Never**
In a discharge summary or letter from other non-ICU medical/surgical team	**Occasionally**
I contact the hospital myself to find out	**Rarely**
The patient's relatives tell me	**Often**
The patient tells me after hospital discharge	**Occasionally**

Page 6

Q6 If you received information about your patient's stay in ICU, how often would it include details about the following aspects of their critical illness?

Shock	**Rarely**
Respiratory failure and mechanical ventilation	**Rarely**
Acute kidney injury requiring acute dialysis	**Rarely**
Acute encephalopathy / Delirium	**Rarely**
ARDS (acute respiratory distress syndrome)	**Rarely**
Neuromuscular weakness	**Rarely**
Tracheostomy insertion	**Rarely**
The duration of patient's stay in ICU	**Occasionally**

Page 7

Q7 If, during a recent hospital stay, your patient was in ICU, would you record this ICU admission in the medical/surgical history section of their notes? **No**

Page 8

Q8 You receive a discharge summary in the post about your patient who was recently discharged from hospital after a severe illness. The summary confirms that they were in ICU during the hospital stay. Please comment on the following statements about the patient's follow-up care:

Because of the patient's ICU admission, I would make contact with them, even if the discharge summary did not request specific follow-up	**Disagree**
If the patient did not self-present to my surgery for follow-up, the ICU admission would prompt me to schedule a consultation with them	**Neutral**
If the patient did not self-present to my surgery for follow-up, the ICU admission would prompt me to schedule a consultation with the patient and a close relative	**Neutral**

Page 9

Q9 Have you ever attended an educational meeting at which you learned about the long term complications of critical illness?

No

Page 10

Q10 Are you aware of any published Guidelines about the rehabilitation of patients following hospital discharge after ICU admission with critical illness?

No

Page 11

Q11 Do you think it would benefit your patient and/or their family members if you received details about their ICU admission?

Yes

#59

COMPLETE

Collector: Web Link - Manual Entry 1 (Web Link)
Started: Tuesday, September 27, 2016 2:26:20 PM
Last Modified: Tuesday, September 27, 2016 2:27:28 PM
Time Spent: 00:01:08
IP Address: 86.145.61.199

Page 1

Q1 Which of the following best describes your GP practice?

Urban practice (Dublin, Cork, Galway, Limerick)

Page 2

Q2 Please indicate how long you have been working as a general practitioner.

>20years

Page 3

Q3 In which county do you conduct most of your GP work?

Cork

Page 4

Q4 Please comment on the following statements regarding communication you receive after your patients are discharged from hospital.

I receive details of the patient's admission	**Always**
I receive details of their admission within 30days of patient's discharge	**Often**
The details I receive include whether patient was admitted to the ICU or not	**Often**

Page 5

Q5 If you receive information about your patient's ICU stay, by which method(s) would you receive this information?

In a discharge summary or letter from ICU medical team	**Always**

Page 6

Q6 If you received information about your patient's stay in ICU, how often would it include details about the following aspects of their critical illness?

Shock	**Always**
Respiratory failure and mechanical ventilation	**Always**
Acute encephalopathy / Delirium	**Often**
ARDS (acute respiratory distress syndrome)	**Always**
Tracheostomy insertion	**Often**
The duration of patient's stay in ICU	**Often**

Page 7

Q7 If, during a recent hospital stay, your patient was in ICU, would you record this ICU admission in the medical/surgical history section of their notes?

No

Page 8

Q8 You receive a discharge summary in the post about your patient who was recently discharged from hospital after a severe illness. The summary confirms that they were in ICU during the hospital stay. Please comment on the following statements about the patient's follow-up care:

Because of the patient's ICU admission, I would make contact with them, even if the discharge summary did not request specific follow-up	**Agree**
If the patient did not self-present to my surgery for follow-up, the ICU admission would prompt me to schedule a consultation with them	**Neutral**
If the patient did not self-present to my surgery for follow-up, the ICU admission would prompt me to schedule a consultation with the patient and a close relative	**Neutral**

Page 9

Q9 Have you ever attended an educational meeting at which you learned about the long term complications of critical illness?

No

Page 10

Q10 Are you aware of any published Guidelines about the rehabilitation of patients following hospital discharge after ICU admission with critical illness?

No

Page 11

Q11 Do you think it would benefit your patient and/or their family members if you received details about their ICU admission?

Yes

#60

COMPLETE

Collector: Web Link - Manual Entry 1 (Web Link)
Started: Tuesday, September 27, 2016 2:27:49 PM
Last Modified: Tuesday, September 27, 2016 2:28:53 PM
Time Spent: 00:01:04
IP Address: 86.145.61.199

Page 1

Q1 Which of the following best describes your GP practice?

Rural practice (outside city)

Page 2

Q2 Please indicate how long you have been working as a general practitioner.

10 years to 20 years

Page 3

Q3 In which county do you conduct most of your GP work?

Galway

Page 4

Q4 Please comment on the following statements regarding communication you receive after your patients are discharged from hospital.

I receive details of the patient’s admission	**Often**
I receive details of their admission within 30days of patient’s discharge	**Often**
The details I receive include whether patient was admitted to the ICU or not	**Often**

Page 5

Q5 If you receive information about your patient's ICU stay, by which method(s) would you receive this information?

The ICU/anaesthetic staff phone me directly	**Never**
In a discharge summary or letter from ICU medical team	**Rarely**
In a discharge summary or letter from other non-ICU medical/surgical team	**Often**
I contact the hospital myself to find out	**Occasionally**
The patient's relatives tell me	**Often**
The patient tells me after hospital discharge	**Often**

Page 6

Q6 If you received information about your patient's stay in ICU, how often would it include details about the following aspects of their critical illness?

Shock	**Never**
Respiratory failure and mechanical ventilation	**Often**
Acute kidney injury requiring acute dialysis	**Often**
Acute encephalopathy / Delirium	**Occasionally**
ARDS (acute respiratory distress syndrome)	**Occasionally**
Neuromuscular weakness	**Occasionally**
Tracheostomy insertion	**Often**
The duration of patient's stay in ICU	**Often**

Page 7

Q7 If, during a recent hospital stay, your patient was in ICU, would you record this ICU admission in the medical/surgical history section of their notes? **Yes**

Page 8

Q8 You receive a discharge summary in the post about your patient who was recently discharged from hospital after a severe illness. The summary confirms that they were in ICU during the hospital stay. Please comment on the following statements about the patient's follow-up care:

Because of the patient's ICU admission, I would make contact with them, even if the discharge summary did not request specific follow-up	**Agree**
If the patient did not self-present to my surgery for follow-up, the ICU admission would prompt me to schedule a consultation with them	**Neutral**
If the patient did not self-present to my surgery for follow-up, the ICU admission would prompt me to schedule a consultation with the patient and a close relative	**Disagree**

Page 9

Q9 Have you ever attended an educational meeting at which you learned about the long term complications of critical illness?

No

Page 10

Q10 Are you aware of any published Guidelines about the rehabilitation of patients following hospital discharge after ICU admission with critical illness?

No

Page 11

Q11 Do you think it would benefit your patient and/or their family members if you received details about their ICU admission?

Yes

#61

COMPLETE

Collector: Web Link - Manual Entry 1 (Web Link)
Started: Tuesday, September 27, 2016 2:29:18 PM
Last Modified: Tuesday, September 27, 2016 2:32:32 PM
Time Spent: 00:03:14
IP Address: 86.145.61.199

Page 1

Q1 Which of the following best describes your GP practice?

Urban practice (Dublin, Cork, Galway, Limerick)

Page 2

Q2 Please indicate how long you have been working as a general practitioner.

>20years

Page 3

Q3 In which county do you conduct most of your GP work?

-

Page 4

Q4 Please comment on the following statements regarding communication you receive after your patients are discharged from hospital.

I receive details of the patient's admission	**Often**
I receive details of their admission within 30days of patient's discharge	**Often**
The details I receive include whether patient was admitted to the ICU or not	**Often**

Page 5

Q5 If you receive information about your patient's ICU stay, by which method(s) would you receive this information?

The ICU/anaesthetic staff phone me directly	**Never**
In a discharge summary or letter from ICU medical team	**Never**
In a discharge summary or letter from other non-ICU medical/surgical team	**Often**
I contact the hospital myself to find out	**Rarely**
The patient's relatives tell me	**Occasionally**
The patient tells me after hospital discharge	**Often**

Page 6

Q6 If you received information about your patient's stay in ICU, how often would it include details about the following aspects of their critical illness?

Shock	**Occasionally**
Respiratory failure and mechanical ventilation	**Often**
Acute kidney injury requiring acute dialysis	**Often**
Acute encephalopathy / Delirium	**Occasionally**
ARDS (acute respiratory distress syndrome)	**Occasionally**
Neuromuscular weakness	**Occasionally**
Tracheostomy insertion	**Occasionally**
The duration of patient's stay in ICU	**Often**

Page 7

Q7 If, during a recent hospital stay, your patient was in ICU, would you record this ICU admission in the medical/surgical history section of their notes?

No

Page 8

Q8 You receive a discharge summary in the post about your patient who was recently discharged from hospital after a severe illness. The summary confirms that they were in ICU during the hospital stay. Please comment on the following statements about the patient's follow-up care:

Because of the patient's ICU admission, I would make contact with them, even if the discharge summary did not request specific follow-up	**Disagree**
If the patient did not self-present to my surgery for follow-up, the ICU admission would prompt me to schedule a consultation with them	**Neutral**
If the patient did not self-present to my surgery for follow-up, the ICU admission would prompt me to schedule a consultation with the patient and a close relative	**Neutral**

Page 9

Q9 Have you ever attended an educational meeting at which you learned about the long term complications of critical illness?

No

Page 10

Q10 Are you aware of any published Guidelines about the rehabilitation of patients following hospital discharge after ICU admission with critical illness?

No

Page 11

Q11 Do you think it would benefit your patient and/or their family members if you received details about their ICU admission?

Yes,
If Yes, what the possible
benefits?:
Help provide better support, but already aware that distress patient and families need support

#62

COMPLETE

Collector: Web Link - Manual Entry 1 (Web Link)
Started: Tuesday, September 27, 2016 2:32:55 PM
Last Modified: Tuesday, September 27, 2016 2:35:18 PM
Time Spent: 00:02:23
IP Address: 86.145.61.199

Page 1

Q1 Which of the following best describes your GP practice?

Rural practice (outside city)

Page 2

Q2 Please indicate how long you have been working as a general practitioner.

>20years

Page 3

Q3 In which county do you conduct most of your GP work?

Louth

Page 4

Q4 Please comment on the following statements regarding communication you receive after your patients are discharged from hospital.

I receive details of the patient's admission	**Always**
I receive details of their admission within 30days of patient's discharge	**Often**
The details I receive include whether patient was admitted to the ICU or not	**Occasionally**

Page 5

Q5 If you receive information about your patient's ICU stay, by which method(s) would you receive this information?

The ICU/anaesthetic staff phone me directly	**Never**
In a discharge summary or letter from ICU medical team	**Never**
In a discharge summary or letter from other non-ICU medical/surgical team	**Always**
I contact the hospital myself to find out	**Never**
The patient's relatives tell me	**Occasionally**
The patient tells me after hospital discharge	**Occasionally**

Page 6

Q6 If you received information about your patient's stay in ICU, how often would it include details about the following aspects of their critical illness?

Shock	**Never**
Respiratory failure and mechanical ventilation	**Occasionally**
Acute kidney injury requiring acute dialysis	**Occasionally**
Acute encephalopathy / Delirium	**Occasionally**
ARDS (acute respiratory distress syndrome)	**Occasionally**
Neuromuscular weakness	**Occasionally**
Tracheostomy insertion	**Occasionally**
The duration of patient's stay in ICU	**Occasionally**

Page 7

Q7 If, during a recent hospital stay, your patient was in ICU, would you record this ICU admission in the medical/surgical history section of their notes? **Yes**

Page 8

Q8 You receive a discharge summary in the post about your patient who was recently discharged from hospital after a severe illness. The summary confirms that they were in ICU during the hospital stay. Please comment on the following statements about the patient's follow-up care:

Because of the patient's ICU admission, I would make contact with them, even if the discharge summary did not request specific follow-up	**Disagree**
If the patient did not self-present to my surgery for follow-up, the ICU admission would prompt me to schedule a consultation with them	**Neutral**
If the patient did not self-present to my surgery for follow-up, the ICU admission would prompt me to schedule a consultation with the patient and a close relative	**Neutral**

Page 9

Q9 Have you ever attended an educational meeting at which you learned about the long term complications of critical illness?

Yes,
If Yes, what was the event? If No, would you attend one if it was available?:
many

Page 10

Q10 Are you aware of any published Guidelines about the rehabilitation of patients following hospital discharge after ICU admission with critical illness?

No

Page 11

Q11 Do you think it would benefit your patient and/or their family members if you received details about their ICU admission?

Yes,
If Yes, what the possible benefits?:
obviously could preempt / early identification of d/cs

#63

COMPLETE

Collector: Web Link - Manual Entry 1 (Web Link)
Started: Tuesday, September 27, 2016 2:35:36 PM
Last Modified: Tuesday, September 27, 2016 2:36:48 PM
Time Spent: 00:01:12
IP Address: 86.145.61.199

Page 1

Q1 Which of the following best describes your GP practice?

Urban practice (Dublin, Cork, Galway, Limerick)

Page 2

Q2 Please indicate how long you have been working as a general practitioner.

10 years to 20 years

Page 3

Q3 In which county do you conduct most of your GP work?

Louth

Page 4

Q4 Please comment on the following statements regarding communication you receive after your patients are discharged from hospital.

I receive details of the patient’s admission	**Often**
I receive details of their admission within 30days of patient’s discharge	**Often**
The details I receive include whether patient was admitted to the ICU or not	**Occasionally**

Page 5

Q5 If you receive information about your patient's ICU stay, by which method(s) would you receive this information?

The ICU/anaesthetic staff phone me directly	**Never**
In a discharge summary or letter from ICU medical team	**Never**
In a discharge summary or letter from other non-ICU medical/surgical team	**Occasionally**
I contact the hospital myself to find out	**Never**
The patient's relatives tell me	**Rarely**
The patient tells me after hospital discharge	**Occasionally**

Page 6

Q6 If you received information about your patient's stay in ICU, how often would it include details about the following aspects of their critical illness?

Shock	**Rarely**
Respiratory failure and mechanical ventilation	**Never**
Acute kidney injury requiring acute dialysis	**Never**
Acute encephalopathy / Delirium	**Never**
ARDS (acute respiratory distress syndrome)	**Never**
Neuromuscular weakness	**Never**
Tracheostomy insertion	**Never**
The duration of patient's stay in ICU	**Never**

Page 7

Q7 If, during a recent hospital stay, your patient was in ICU, would you record this ICU admission in the medical/surgical history section of their notes? **No**

Page 8

Q8 You receive a discharge summary in the post about your patient who was recently discharged from hospital after a severe illness. The summary confirms that they were in ICU during the hospital stay. Please comment on the following statements about the patient's follow-up care:

Because of the patient's ICU admission, I would make contact with them, even if the discharge summary did not request specific follow-up	**Disagree**
If the patient did not self-present to my surgery for follow-up, the ICU admission would prompt me to schedule a consultation with them	**Agree**
If the patient did not self-present to my surgery for follow-up, the ICU admission would prompt me to schedule a consultation with the patient and a close relative	**Neutral**

Page 9

Q9 Have you ever attended an educational meeting at which you learned about the long term complications of critical illness?

No

Page 10

Q10 Are you aware of any published Guidelines about the rehabilitation of patients following hospital discharge after ICU admission with critical illness?

No

Page 11

Q11 Do you think it would benefit your patient and/or their family members if you received details about their ICU admission?

Yes

#64

COMPLETE

Collector: Web Link - Manual Entry 1 (Web Link)
Started: Tuesday, September 27, 2016 2:37:22 PM
Last Modified: Tuesday, September 27, 2016 2:38:30 PM
Time Spent: 00:01:08
IP Address: 86.145.61.199

Page 1

Q1 Which of the following best describes your GP practice?

Rural practice (outside city)

Page 2

Q2 Please indicate how long you have been working as a general practitioner.

<10years

Page 3

Q3 In which county do you conduct most of your GP work?

Waterford

Page 4

Q4 Please comment on the following statements regarding communication you receive after your patients are discharged from hospital.

I receive details of the patient’s admission	**Often**
I receive details of their admission within 30days of patient’s discharge	**Occasionally**
The details I receive include whether patient was admitted to the ICU or not	**Rarely**

Page 5

Q5 If you receive information about your patient's ICU stay, by which method(s) would you receive this information?

The ICU/anaesthetic staff phone me directly	**Never**
In a discharge summary or letter from ICU medical team	**Never**
In a discharge summary or letter from other non-ICU medical/surgical team	**Occasionally**
I contact the hospital myself to find out	**Rarely**
The patient's relatives tell me	**Often**
The patient tells me after hospital discharge	**Often**

Page 6

Q6 If you received information about your patient's stay in ICU, how often would it include details about the following aspects of their critical illness?

Shock	**Never**
Respiratory failure and mechanical ventilation	**Never**
Acute kidney injury requiring acute dialysis	**Never**
Acute encephalopathy / Delirium	**Never**
ARDS (acute respiratory distress syndrome)	**Never**
Neuromuscular weakness	**Never**
Tracheostomy insertion	**Never**
The duration of patient's stay in ICU	**Never**

Page 7

Q7 If, during a recent hospital stay, your patient was in ICU, would you record this ICU admission in the medical/surgical history section of their notes? **Yes**

Page 8

Q8 You receive a discharge summary in the post about your patient who was recently discharged from hospital after a severe illness. The summary confirms that they were in ICU during the hospital stay. Please comment on the following statements about the patient's follow-up care:

Because of the patient's ICU admission, I would make contact with them, even if the discharge summary did not request specific follow-up	**Disagree**
If the patient did not self-present to my surgery for follow-up, the ICU admission would prompt me to schedule a consultation with them	**Agree**
If the patient did not self-present to my surgery for follow-up, the ICU admission would prompt me to schedule a consultation with the patient and a close relative	**Neutral**

Page 9

Q9 Have you ever attended an educational meeting at which you learned about the long term complications of critical illness?

No

Page 10

Q10 Are you aware of any published Guidelines about the rehabilitation of patients following hospital discharge after ICU admission with critical illness?

No

Page 11

Q11 Do you think it would benefit your patient and/or their family members if you received details about their ICU admission?

Yes

#65

COMPLETE

Collector: Web Link - Manual Entry 1 (Web Link)
Started: Tuesday, September 27, 2016 2:38:54 PM
Last Modified: Tuesday, September 27, 2016 2:42:09 PM
Time Spent: 00:03:15
IP Address: 86.145.61.199

Page 1

Q1 Which of the following best describes your GP practice?

Urban practice (Dublin, Cork, Galway, Limerick)

Page 2

Q2 Please indicate how long you have been working as a general practitioner.

>20years

Page 3

Q3 In which county do you conduct most of your GP work?

Dublin

Page 4

Q4 Please comment on the following statements regarding communication you receive after your patients are discharged from hospital.

I receive details of the patient’s admission	**Always**
I receive details of their admission within 30days of patient’s discharge	**Always**
The details I receive include whether patient was admitted to the ICU or not	**Never**

Page 5

Q5 If you receive information about your patient's ICU stay, by which method(s) would you receive this information?

The ICU/anaesthetic staff phone me directly	**Never**
In a discharge summary or letter from ICU medical team	**Never**
In a discharge summary or letter from other non-ICU medical/surgical team	**Never**
The patient's relatives tell me	**Often**
The patient tells me after hospital discharge	**Always**

Page 6

Q6 If you received information about your patient's stay in ICU, how often would it include details about the following aspects of their critical illness?

Shock	**Never**
Respiratory failure and mechanical ventilation	**Never**
Acute kidney injury requiring acute dialysis	**Occasionally**
Acute encephalopathy / Delirium	**Occasionally**
ARDS (acute respiratory distress syndrome)	**Occasionally**
Neuromuscular weakness	**Never**
Tracheostomy insertion	**Rarely**
The duration of patient's stay in ICU	**Never**

Page 7

Q7 If, during a recent hospital stay, your patient was in ICU, would you record this ICU admission in the medical/surgical history section of their notes?

Yes

Page 8

Q8 You receive a discharge summary in the post about your patient who was recently discharged from hospital after a severe illness. The summary confirms that they were in ICU during the hospital stay. Please comment on the following statements about the patient's follow-up care:

Because of the patient's ICU admission, I would make contact with them, even if the discharge summary did not request specific follow-up	**Disagree**
If the patient did not self-present to my surgery for follow-up, the ICU admission would prompt me to schedule a consultation with them	**Agree**
If the patient did not self-present to my surgery for follow-up, the ICU admission would prompt me to schedule a consultation with the patient and a close relative	**Agree**

Page 9

Q9 Have you ever attended an educational meeting at which you learned about the long term complications of critical illness?

No,
If Yes, what was the event? If No, would you attend one if it was available?:
yes

Page 10

Q10 Are you aware of any published Guidelines about the rehabilitation of patients following hospital discharge after ICU admission with critical illness?

No

Page 11

Q11 Do you think it would benefit your patient and/or their family members if you received details about their ICU admission?

Yes,
If Yes, what the possible benefits?:
It defines how serious their illness was. I am more mindful they may need a higher level of support, fear of hospitalisation in the future.

#66

COMPLETE

Collector: Web Link - Manual Entry 2 (Web Link)
Started: Wednesday, September 28, 2016 9:20:57 AM
Last Modified: Wednesday, September 28, 2016 9:22:05 AM
Time Spent: 00:01:08
IP Address: 86.145.61.199

Page 1

Q1 Which of the following best describes your GP practice?

Rural practice (outside city)

Page 2

Q2 Please indicate how long you have been working as a general practitioner.

<10years

Page 3

Q3 In which county do you conduct most of your GP work?

Clare

Page 4

Q4 Please comment on the following statements regarding communication you receive after your patients are discharged from hospital.

The details I receive include whether patient was admitted to the ICU or not — **Always**

Page 5

Q5 If you receive information about your patient's ICU stay, by which method(s) would you receive this information?

In a discharge summary or letter from other non-ICU medical/surgical team — **Always**

Page 6

Q6 If you received information about your patient's stay in ICU, how often would it include details about the following aspects of their critical illness?

Acute kidney injury requiring acute dialysis	**Always**

Page 7

Q7 If, during a recent hospital stay, your patient was in ICU, would you record this ICU admission in the medical/surgical history section of their notes?

Yes

Page 8

Q8 You receive a discharge summary in the post about your patient who was recently discharged from hospital after a severe illness. The summary confirms that they were in ICU during the hospital stay. Please comment on the following statements about the patient's follow-up care:

Because of the patient's ICU admission, I would make contact with them, even if the discharge summary did not request specific follow-up	**Strongly disagree**
If the patient did not self-present to my surgery for follow-up, the ICU admission would prompt me to schedule a consultation with them	**Disagree**
If the patient did not self-present to my surgery for follow-up, the ICU admission would prompt me to schedule a consultation with the patient and a close relative	**Disagree**

Page 9

Q9 Have you ever attended an educational meeting at which you learned about the long term complications of critical illness?

No

Page 10

Q10 Are you aware of any published Guidelines about the rehabilitation of patients following hospital discharge after ICU admission with critical illness?

No

Page 11

Q11 Do you think it would benefit your patient and/or their family members if you received details about their ICU admission?

Yes

#67

COMPLETE

Collector: Web Link - Manual Entry 2 (Web Link)
Started: Wednesday, September 28, 2016 9:22:40 AM
Last Modified: Wednesday, September 28, 2016 9:27:29 AM
Time Spent: 00:04:49
IP Address: 86.145.61.199

Page 1

Q1 Which of the following best describes your GP practice?

Urban practice (Dublin, Cork, Galway, Limerick)

Page 2

Q2 Please indicate how long you have been working as a general practitioner.

>20years

Page 3

Q3 In which county do you conduct most of your GP work?

Dublin

Page 4

Q4 Please comment on the following statements regarding communication you receive after your patients are discharged from hospital.

I receive details of the patient's admission	**Often**
I receive details of their admission within 30days of patient's discharge	**Often**
The details I receive include whether patient was admitted to the ICU or not	**Rarely**

Page 5

Q5 If you receive information about your patient's ICU stay, by which method(s) would you receive this information?

The ICU/anaesthetic staff phone me directly	**Rarely**
In a discharge summary or letter from ICU medical team	**Never**
In a discharge summary or letter from other non-ICU medical/surgical team	**Occasionally**
The patient's relatives tell me	**Occasionally**
The patient tells me after hospital discharge	**Rarely**

Page 6

Q6 If you received information about your patient's stay in ICU, how often would it include details about the following aspects of their critical illness?

Shock	**Never**
Respiratory failure and mechanical ventilation	**Rarely**
Acute kidney injury requiring acute dialysis	**Never**
Acute encephalopathy / Delirium	**Rarely**
ARDS (acute respiratory distress syndrome)	**Rarely**
Neuromuscular weakness	**Never**
Tracheostomy insertion	**Never**
The duration of patient's stay in ICU	**Rarely**

Page 7

Q7 If, during a recent hospital stay, your patient was in ICU, would you record this ICU admission in the medical/surgical history section of their notes? **No**

Page 8

Q8 You receive a discharge summary in the post about your patient who was recently discharged from hospital after a severe illness. The summary confirms that they were in ICU during the hospital stay. Please comment on the following statements about the patient's follow-up care:

Because of the patient's ICU admission, I would make contact with them, even if the discharge summary did not request specific follow-up	**Agree**
If the patient did not self-present to my surgery for follow-up, the ICU admission would prompt me to schedule a consultation with them	**Strongly disagree**
If the patient did not self-present to my surgery for follow-up, the ICU admission would prompt me to schedule a consultation with the patient and a close relative	**Strongly disagree**

Page 9

Q9 Have you ever attended an educational meeting at which you learned about the long term complications of critical illness?

No

Page 10

Q10 Are you aware of any published Guidelines about the rehabilitation of patients following hospital discharge after ICU admission with critical illness?

No

Page 11

Q11 Do you think it would benefit your patient and/or their family members if you received details about their ICU admission?

Yes,
If Yes, what the possible benefits?:
Better understanding the severity of the illness + stress for them and for the family

#68

COMPLETE

Collector: Web Link - Manual Entry 2 (Web Link)
Started: Wednesday, September 28, 2016 9:27:52 AM
Last Modified: Wednesday, September 28, 2016 9:28:51 AM
Time Spent: 00:00:59
IP Address: 86.145.61.199

Page 1

Q1 Which of the following best describes your GP practice?

Rural practice (outside city)

Page 2

Q2 Please indicate how long you have been working as a general practitioner.

10 years to 20 years

Page 3

Q3 In which county do you conduct most of your GP work?

Tipperary

Page 4

Q4 Please comment on the following statements regarding communication you receive after your patients are discharged from hospital.

I receive details of the patient’s admission	**Never**
I receive details of their admission within 30days of patient’s discharge	**Often**
The details I receive include whether patient was admitted to the ICU or not	**Often**

Page 5

Q5 If you receive information about your patient's ICU stay, by which method(s) would you receive this information?

The ICU/anaesthetic staff phone me directly	**Rarely**
In a discharge summary or letter from ICU medical team	**Rarely**
In a discharge summary or letter from other non-ICU medical/surgical team	**Often**
I contact the hospital myself to find out	**Occasionally**
The patient's relatives tell me	**Often**
The patient tells me after hospital discharge	**Often**

Page 6

Q6 If you received information about your patient's stay in ICU, how often would it include details about the following aspects of their critical illness?

Shock	**Occasionally**
Respiratory failure and mechanical ventilation	**Occasionally**
Acute kidney injury requiring acute dialysis	**Occasionally**
Acute encephalopathy / Delirium	**Occasionally**
ARDS (acute respiratory distress syndrome)	**Occasionally**
Neuromuscular weakness	**Occasionally**
Tracheostomy insertion	**Occasionally**
The duration of patient's stay in ICU	**Occasionally**

Page 7

Q7 If, during a recent hospital stay, your patient was in ICU, would you record this ICU admission in the medical/surgical history section of their notes? **Yes**

Page 8

Q8 You receive a discharge summary in the post about your patient who was recently discharged from hospital after a severe illness. The summary confirms that they were in ICU during the hospital stay. Please comment on the following statements about the patient's follow-up care:

Because of the patient's ICU admission, I would make contact with them, even if the discharge summary did not request specific follow-up	**Disagree**
If the patient did not self-present to my surgery for follow-up, the ICU admission would prompt me to schedule a consultation with them	**Neutral**
If the patient did not self-present to my surgery for follow-up, the ICU admission would prompt me to schedule a consultation with the patient and a close relative	**Neutral**

Page 9

Q9 Have you ever attended an educational meeting at which you learned about the long term complications of critical illness?

No

Page 10

Q10 Are you aware of any published Guidelines about the rehabilitation of patients following hospital discharge after ICU admission with critical illness?

No

Page 11

Q11 Do you think it would benefit your patient and/or their family members if you received details about their ICU admission?

Yes

#69

COMPLETE

Collector: Web Link - Manual Entry 2 (Web Link)
Started: Wednesday, September 28, 2016 9:29:16 AM
Last Modified: Wednesday, September 28, 2016 9:31:12 AM
Time Spent: 00:01:56
IP Address: 86.145.61.199

Page 1

Q1 Which of the following best describes your GP practice?

Urban practice (Dublin, Cork, Galway, Limerick)

Page 2

Q2 Please indicate how long you have been working as a general practitioner.

>20years

Page 3

Q3 In which county do you conduct most of your GP work?

Dublin

Page 4

Q4 Please comment on the following statements regarding communication you receive after your patients are discharged from hospital.

I receive details of the patient's admission	**Often**
I receive details of their admission within 30days of patient's discharge	**Occasionally**
The details I receive include whether patient was admitted to the ICU or not	**Rarely**

Page 5

Q5 If you receive information about your patient’s ICU stay, by which method(s) would you receive this information?

The ICU/anaesthetic staff phone me directly	**Never**
In a discharge summary or letter from ICU medical team	**Never**
In a discharge summary or letter from other non-ICU medical/surgical team	**Occasionally**
I contact the hospital myself to find out	**Often**
The patient’s relatives tell me	**Often**
The patient tells me after hospital discharge	**Often**

Page 6

Q6 If you received information about your patient’s stay in ICU, how often would it include details about the following aspects of their critical illness?

Shock	**Never**
Respiratory failure and mechanical ventilation	**Often**
Acute kidney injury requiring acute dialysis	**Often**
Acute encephalopathy / Delirium	**Often**
ARDS (acute respiratory distress syndrome)	**Often**
Neuromuscular weakness	**Rarely**
Tracheostomy insertion	**Rarely**
The duration of patient’s stay in ICU	**Never**

Page 7

Q7 If, during a recent hospital stay, your patient was in ICU, would you record this ICU admission in the medical/surgical history section of their notes? **No**

Page 8

Q8 You receive a discharge summary in the post about your patient who was recently discharged from hospital after a severe illness. The summary confirms that they were in ICU during the hospital stay. Please comment on the following statements about the patient's follow-up care:

Because of the patient's ICU admission, I would make contact with them, even if the discharge summary did not request specific follow-up	**Strongly disagree**
If the patient did not self-present to my surgery for follow-up, the ICU admission would prompt me to schedule a consultation with them	**Strongly agree**
If the patient did not self-present to my surgery for follow-up, the ICU admission would prompt me to schedule a consultation with the patient and a close relative	**Agree**

Page 9

Q9 Have you ever attended an educational meeting at which you learned about the long term complications of critical illness?

No,
If Yes, what was the event? If No, would you attend one if it was available?:
Yes

Page 10

Q10 Are you aware of any published Guidelines about the rehabilitation of patients following hospital discharge after ICU admission with critical illness?

No

Page 11

Q11 Do you think it would benefit your patient and/or their family members if you received details about their ICU admission?

Yes,
If Yes, what the possible benefits?:
detail; communication; continuity of care; empathy

#70

COMPLETE

Collector: Web Link - Manual Entry 2 (Web Link)
Started: Wednesday, September 28, 2016 9:31:35 AM
Last Modified: Wednesday, September 28, 2016 9:32:43 AM
Time Spent: 00:01:08
IP Address: 86.145.61.199

Page 1

Q1 Which of the following best describes your GP practice?

Rural practice (outside city)

Page 2

Q2 Please indicate how long you have been working as a general practitioner.

10 years to 20 years

Page 3

Q3 In which county do you conduct most of your GP work?

Dublin

Page 4

Q4 Please comment on the following statements regarding communication you receive after your patients are discharged from hospital.

I receive details of the patient's admission	**Often**
I receive details of their admission within 30days of patient's discharge	**Often**
The details I receive include whether patient was admitted to the ICU or not	**Always**

Page 5

Q5 If you receive information about your patient's ICU stay, by which method(s) would you receive this information?

In a discharge summary or letter from other non-ICU medical/surgical team	**Often**
I contact the hospital myself to find out	**Always**
The patient tells me after hospital discharge	**Often**

Page 6

Q6 If you received information about your patient's stay in ICU, how often would it include details about the following aspects of their critical illness?

Shock	**Never**
Respiratory failure and mechanical ventilation	**Never**
Acute kidney injury requiring acute dialysis	**Never**
Acute encephalopathy / Delirium	**Never**
ARDS (acute respiratory distress syndrome)	**Never**
Neuromuscular weakness	**Never**
Tracheostomy insertion	**Never**
The duration of patient's stay in ICU	**Never**

Page 7

Q7 If, during a recent hospital stay, your patient was in ICU, would you record this ICU admission in the medical/surgical history section of their notes? **Yes**

Page 8

Q8 You receive a discharge summary in the post about your patient who was recently discharged from hospital after a severe illness. The summary confirms that they were in ICU during the hospital stay. Please comment on the following statements about the patient's follow-up care:

If the patient did not self-present to my surgery for follow-up, the ICU admission would prompt me to schedule a consultation with the patient and a close relative	**Strongly agree**

Page 9

Q9 Have you ever attended an educational meeting at which you learned about the long term complications of critical illness? **Yes**

Page 10

Q10 Are you aware of any published Guidelines about the rehabilitation of patients following hospital discharge after ICU admission with critical illness?

No

Page 11

Q11 Do you think it would benefit your patient and/or their family members if you received details about their ICU admission?

Yes

#71

COMPLETE

Collector: Web Link - Manual Entry 2 (Web Link)
Started: Wednesday, September 28, 2016 9:33:03 AM
Last Modified: Wednesday, September 28, 2016 9:34:29 AM
Time Spent: 00:01:26
IP Address: 86.145.61.199

Page 1

Q1 Which of the following best describes your GP practice?

Rural practice (outside city)

Page 2

Q2 Please indicate how long you have been working as a general practitioner.

10 years to 20 years

Page 3

Q3 In which county do you conduct most of your GP work?

-

Page 4

Q4 Please comment on the following statements regarding communication you receive after your patients are discharged from hospital.

I receive details of the patient's admission	**Often**
I receive details of their admission within 30days of patient's discharge	**Often**
The details I receive include whether patient was admitted to the ICU or not	**Occasionally**

Page 5

Q5 If you receive information about your patient's ICU stay, by which method(s) would you receive this information?

The ICU/anaesthetic staff phone me directly	**Never**
In a discharge summary or letter from ICU medical team	**Never**
In a discharge summary or letter from other non-ICU medical/surgical team	**Occasionally**
I contact the hospital myself to find out	**Rarely**
The patient's relatives tell me	**Often**
The patient tells me after hospital discharge	**Often**

Page 6

Q6 If you received information about your patient's stay in ICU, how often would it include details about the following aspects of their critical illness?

Respiratory failure and mechanical ventilation	**Occasionally**
Acute kidney injury requiring acute dialysis	**Occasionally**
Acute encephalopathy / Delirium	**Occasionally**
ARDS (acute respiratory distress syndrome)	**Occasionally**
Neuromuscular weakness	**Rarely**
Tracheostomy insertion	**Occasionally**
The duration of patient's stay in ICU	**Occasionally**

Page 7

Q7 If, during a recent hospital stay, your patient was in ICU, would you record this ICU admission in the medical/surgical history section of their notes? **Yes**

Page 8

Q8 You receive a discharge summary in the post about your patient who was recently discharged from hospital after a severe illness. The summary confirms that they were in ICU during the hospital stay. Please comment on the following statements about the patient's follow-up care:

Because of the patient's ICU admission, I would make contact with them, even if the discharge summary did not request specific follow-up	**Disagree**
If the patient did not self-present to my surgery for follow-up, the ICU admission would prompt me to schedule a consultation with them	**Neutral**
If the patient did not self-present to my surgery for follow-up, the ICU admission would prompt me to schedule a consultation with the patient and a close relative	**Neutral**

Page 9

Q9 Have you ever attended an educational meeting at which you learned about the long term complications of critical illness?

No

Page 10

Q10 Are you aware of any published Guidelines about the rehabilitation of patients following hospital discharge after ICU admission with critical illness?

No

Page 11

Q11 Do you think it would benefit your patient and/or their family members if you received details about their ICU admission?

Yes

#72

COMPLETE

Collector: Web Link - Manual Entry 2 (Web Link)
Started: Wednesday, September 28, 2016 9:34:48 AM
Last Modified: Wednesday, September 28, 2016 9:37:07 AM
Time Spent: 00:02:19
IP Address: 86.145.61.199

Page 1

Q1 Which of the following best describes your GP practice?

Rural practice (outside city)

Page 2

Q2 Please indicate how long you have been working as a general practitioner.

>20years

Page 3

Q3 In which county do you conduct most of your GP work?

-

Page 4

Q4 Please comment on the following statements regarding communication you receive after your patients are discharged from hospital.

I receive details of the patient's admission	**Often**
I receive details of their admission within 30days of patient's discharge	**Often**
The details I receive include whether patient was admitted to the ICU or not	**Occasionally**

Page 5

Q5 If you receive information about your patient's ICU stay, by which method(s) would you receive this information?

In a discharge summary or letter from other non-ICU medical/surgical team	**Occasionally**
The patient's relatives tell me	**Occasionally**
The patient tells me after hospital discharge	**Occasionally**

Page 6

Q6 If you received information about your patient's stay in ICU, how often would it include details about the following aspects of their critical illness?

Shock	**Rarely**
Respiratory failure and mechanical ventilation	**Rarely**
Acute kidney injury requiring acute dialysis	**Rarely**
Acute encephalopathy / Delirium	**Never**
ARDS (acute respiratory distress syndrome)	**Never**
Neuromuscular weakness	**Never**
Tracheostomy insertion	**Never**
The duration of patient's stay in ICU	**Never**

Page 7

Q7 If, during a recent hospital stay, your patient was in ICU, would you record this ICU admission in the medical/surgical history section of their notes? **Yes**

Page 8

Q8 You receive a discharge summary in the post about your patient who was recently discharged from hospital after a severe illness. The summary confirms that they were in ICU during the hospital stay. Please comment on the following statements about the patient's follow-up care:

Because of the patient's ICU admission, I would make contact with them, even if the discharge summary did not request specific follow-up	**Neutral**
If the patient did not self-present to my surgery for follow-up, the ICU admission would prompt me to schedule a consultation with them	**Disagree**
If the patient did not self-present to my surgery for follow-up, the ICU admission would prompt me to schedule a consultation with the patient and a close relative	**Strongly disagree**

Page 9

Q9 Have you ever attended an educational meeting at which you learned about the long term complications of critical illness? **No**

Page 10

Q10 Are you aware of any published Guidelines about the rehabilitation of patients following hospital discharge after ICU admission with critical illness?

No

Page 11

Q11 Do you think it would benefit your patient and/or their family members if you received details about their ICU admission?

Yes,
If Yes, what the possible
benefits?:
a conversation to review the experience could help its processing

#73

COMPLETE

Collector: Web Link - Manual Entry 2 (Web Link)
Started: Wednesday, September 28, 2016 9:37:09 AM
Last Modified: Wednesday, September 28, 2016 9:38:13 AM
Time Spent: 00:01:04
IP Address: 86.145.61.199

Page 1

Q1 Which of the following best describes your GP practice?

Urban practice (Dublin, Cork, Galway, Limerick)

Page 2

Q2 Please indicate how long you have been working as a general practitioner.

>20years

Page 3

Q3 In which county do you conduct most of your GP work?

Cork

Page 4

Q4 Please comment on the following statements regarding communication you receive after your patients are discharged from hospital.

I receive details of the patient’s admission	**Rarely**
I receive details of their admission within 30days of patient’s discharge	**Often**
The details I receive include whether patient was admitted to the ICU or not	**Often**

Page 5

Q5 If you receive information about your patient's ICU stay, by which method(s) would you receive this information?

The ICU/anaesthetic staff phone me directly	**Never**
In a discharge summary or letter from ICU medical team	**Never**
In a discharge summary or letter from other non-ICU medical/surgical team	**Never**
I contact the hospital myself to find out	**Never**
The patient's relatives tell me	**Never**
The patient tells me after hospital discharge	**Never**

Page 6

Q6 If you received information about your patient's stay in ICU, how often would it include details about the following aspects of their critical illness?

Shock	**Never**
Respiratory failure and mechanical ventilation	**Often**
Acute kidney injury requiring acute dialysis	**Occasionally**
Acute encephalopathy / Delirium	**Never**
ARDS (acute respiratory distress syndrome)	**Never**
Neuromuscular weakness	**Never**
Tracheostomy insertion	**Rarely**
The duration of patient's stay in ICU	**Often**

Page 7

Q7 If, during a recent hospital stay, your patient was in ICU, would you record this ICU admission in the medical/surgical history section of their notes? **Yes**

Page 8

Q8 You receive a discharge summary in the post about your patient who was recently discharged from hospital after a severe illness. The summary confirms that they were in ICU during the hospital stay. Please comment on the following statements about the patient's follow-up care:

Because of the patient's ICU admission, I would make contact with them, even if the discharge summary did not request specific follow-up	**Disagree**
If the patient did not self-present to my surgery for follow-up, the ICU admission would prompt me to schedule a consultation with them	**Disagree**
If the patient did not self-present to my surgery for follow-up, the ICU admission would prompt me to schedule a consultation with the patient and a close relative	**Neutral**

Page 9

Q9 Have you ever attended an educational meeting at which you learned about the long term complications of critical illness?

No

Page 10

Q10 Are you aware of any published Guidelines about the rehabilitation of patients following hospital discharge after ICU admission with critical illness?

No

Page 11

Q11 Do you think it would benefit your patient and/or their family members if you received details about their ICU admission?

Yes

#74

COMPLETE

Collector: Web Link - Manual Entry 2 (Web Link)
Started: Wednesday, September 28, 2016 9:38:32 AM
Last Modified: Wednesday, September 28, 2016 9:39:56 AM
Time Spent: 00:01:24
IP Address: 86.145.61.199

Page 1

Q1 Which of the following best describes your GP practice?

Rural practice (outside city)

Page 2

Q2 Please indicate how long you have been working as a general practitioner.

>20years

Page 3

Q3 In which county do you conduct most of your GP work?

Leitrim

Page 4

Q4 Please comment on the following statements regarding communication you receive after your patients are discharged from hospital.

I receive details of the patient's admission	**Always**
I receive details of their admission within 30days of patient's discharge	**Always**
The details I receive include whether patient was admitted to the ICU or not	**Always**

Page 5

Q5 If you receive information about your patient's ICU stay, by which method(s) would you receive this information?

The ICU/anaesthetic staff phone me directly	**Never**
In a discharge summary or letter from ICU medical team	**Rarely**
In a discharge summary or letter from other non-ICU medical/surgical team	**Often**
I contact the hospital myself to find out	**Never**
The patient's relatives tell me	**Rarely**
The patient tells me after hospital discharge	**Rarely**

Page 6

Q6 If you received information about your patient's stay in ICU, how often would it include details about the following aspects of their critical illness?

Respiratory failure and mechanical ventilation	**Often**
Acute kidney injury requiring acute dialysis	**Often**
Acute encephalopathy / Delirium	**Often**
ARDS (acute respiratory distress syndrome)	**Rarely**
Neuromuscular weakness	**Rarely**
Tracheostomy insertion	**Rarely**
The duration of patient's stay in ICU	**Often**

Page 7

Q7 If, during a recent hospital stay, your patient was in ICU, would you record this ICU admission in the medical/surgical history section of their notes? **No**

Page 8

Q8 You receive a discharge summary in the post about your patient who was recently discharged from hospital after a severe illness. The summary confirms that they were in ICU during the hospital stay. Please comment on the following statements about the patient's follow-up care:

Because of the patient's ICU admission, I would make contact with them, even if the discharge summary did not request specific follow-up	**Agree**
If the patient did not self-present to my surgery for follow-up, the ICU admission would prompt me to schedule a consultation with them	**Disagree**
If the patient did not self-present to my surgery for follow-up, the ICU admission would prompt me to schedule a consultation with the patient and a close relative	**Disagree**

Page 9

Q9 Have you ever attended an educational meeting at which you learned about the long term complications of critical illness?

No

Page 10

Q10 Are you aware of any published Guidelines about the rehabilitation of patients following hospital discharge after ICU admission with critical illness?

No

Page 11

Q11 Do you think it would benefit your patient and/or their family members if you received details about their ICU admission?

Yes

#75

COMPLETE

Collector: Web Link - Manual Entry 2 (Web Link)
Started: Wednesday, September 28, 2016 9:40:16 AM
Last Modified: Wednesday, September 28, 2016 9:41:16 AM
Time Spent: 00:01:00
IP Address: 86.145.61.199

Page 1

Q1 Which of the following best describes your GP practice?

Rural practice (outside city)

Page 2

Q2 Please indicate how long you have been working as a general practitioner.

>20years

Page 3

Q3 In which county do you conduct most of your GP work?

Mayo

Page 4

Q4 Please comment on the following statements regarding communication you receive after your patients are discharged from hospital.

I receive details of the patient's admission	**Often**
I receive details of their admission within 30days of patient's discharge	**Occasionally**
The details I receive include whether patient was admitted to the ICU or not	**Rarely**

Page 5

Q5 If you receive information about your patient's ICU stay, by which method(s) would you receive this information?

In a discharge summary or letter from other non-ICU medical/surgical team	**Often**
I contact the hospital myself to find out	**Never**
The patient's relatives tell me	**Often**
The patient tells me after hospital discharge	**Rarely**

Page 6

Q6 If you received information about your patient's stay in ICU, how often would it include details about the following aspects of their critical illness?

Shock	**Never**
Respiratory failure and mechanical ventilation	**Never**
Acute kidney injury requiring acute dialysis	**Never**
Acute encephalopathy / Delirium	**Never**
ARDS (acute respiratory distress syndrome)	**Never**
Neuromuscular weakness	**Never**
Tracheostomy insertion	**Never**
The duration of patient's stay in ICU	**Rarely**

Page 7

**Q7** If, during a recent hospital stay, your patient was in ICU, would you record this ICU admission in the medical/surgical history section of their notes?	**No**

Page 8

Q8 You receive a discharge summary in the post about your patient who was recently discharged from hospital after a severe illness. The summary confirms that they were in ICU during the hospital stay. Please comment on the following statements about the patient's follow-up care:

Because of the patient's ICU admission, I would make contact with them, even if the discharge summary did not request specific follow-up	**Disagree**
If the patient did not self-present to my surgery for follow-up, the ICU admission would prompt me to schedule a consultation with them	**Disagree**
If the patient did not self-present to my surgery for follow-up, the ICU admission would prompt me to schedule a consultation with the patient and a close relative	**Disagree**

Page 9

Q9 Have you ever attended an educational meeting at which you learned about the long term complications of critical illness?

No

Page 10

Q10 Are you aware of any published Guidelines about the rehabilitation of patients following hospital discharge after ICU admission with critical illness?

No

Page 11

Q11 Do you think it would benefit your patient and/or their family members if you received details about their ICU admission?

Yes

#76

COMPLETE

Collector: Web Link - Manual Entry 2 (Web Link)
Started: Wednesday, September 28, 2016 9:41:39 AM
Last Modified: Wednesday, September 28, 2016 9:42:35 AM
Time Spent: 00:00:56
IP Address: 86.145.61.199

Page 1

Q1 Which of the following best describes your GP practice?

Rural practice (outside city)

Page 2

Q2 Please indicate how long you have been working as a general practitioner.

>20years

Page 3

Q3 In which county do you conduct most of your GP work?

Waterford

Page 4

Q4 Please comment on the following statements regarding communication you receive after your patients are discharged from hospital.

I receive details of the patient’s admission	**Occasionally**
I receive details of their admission within 30days of patient’s discharge	**Occasionally**
The details I receive include whether patient was admitted to the ICU or not	**Often**

Page 5

Q5 If you receive information about your patient's ICU stay, by which method(s) would you receive this information?

The ICU/anaesthetic staff phone me directly	**Never**
In a discharge summary or letter from ICU medical team	**Never**
In a discharge summary or letter from other non-ICU medical/surgical team	**Often**
I contact the hospital myself to find out	**Occasionally**
The patient's relatives tell me	**Often**
The patient tells me after hospital discharge	**Often**

Page 6

Q6 If you received information about your patient's stay in ICU, how often would it include details about the following aspects of their critical illness?

Shock	**Occasionally**
Respiratory failure and mechanical ventilation	**Occasionally**
Acute kidney injury requiring acute dialysis	**Occasionally**
Acute encephalopathy / Delirium	**Occasionally**
ARDS (acute respiratory distress syndrome)	**Occasionally**
Neuromuscular weakness	**Occasionally**
Tracheostomy insertion	**Occasionally**
The duration of patient's stay in ICU	**Occasionally**

Page 7

Q7 If, during a recent hospital stay, your patient was in ICU, would you record this ICU admission in the medical/surgical history section of their notes? **Yes**

Page 8

Q8 You receive a discharge summary in the post about your patient who was recently discharged from hospital after a severe illness. The summary confirms that they were in ICU during the hospital stay. Please comment on the following statements about the patient's follow-up care:

Because of the patient's ICU admission, I would make contact with them, even if the discharge summary did not request specific follow-up	**Agree**
If the patient did not self-present to my surgery for follow-up, the ICU admission would prompt me to schedule a consultation with them	**Agree**
If the patient did not self-present to my surgery for follow-up, the ICU admission would prompt me to schedule a consultation with the patient and a close relative	**Neutral**

Page 9

Q9 Have you ever attended an educational meeting at which you learned about the long term complications of critical illness?

No

Page 10

Q10 Are you aware of any published Guidelines about the rehabilitation of patients following hospital discharge after ICU admission with critical illness?

No

Page 11

Q11 Do you think it would benefit your patient and/or their family members if you received details about their ICU admission?

Yes

#77

COMPLETE

Collector: Web Link - Manual Entry 2 (Web Link)
Started: Wednesday, September 28, 2016 9:42:53 AM
Last Modified: Wednesday, September 28, 2016 9:46:05 AM
Time Spent: 00:03:12
IP Address: 86.145.61.199

Page 1

Q1 Which of the following best describes your GP practice?

Urban practice (Dublin, Cork, Galway, Limerick)

Page 2

Q2 Please indicate how long you have been working as a general practitioner.

10 years to 20 years

Page 3

Q3 In which county do you conduct most of your GP work?

Dublin

Page 4

Q4 Please comment on the following statements regarding communication you receive after your patients are discharged from hospital.

I receive details of the patient’s admission	**Often**
I receive details of their admission within 30days of patient’s discharge	**Often**
The details I receive include whether patient was admitted to the ICU or not	**Often**

Page 5

Q5 If you receive information about your patient's ICU stay, by which method(s) would you receive this information?

The ICU/anaesthetic staff phone me directly	**Never**
In a discharge summary or letter from ICU medical team	**Never**
In a discharge summary or letter from other non-ICU medical/surgical team	**Often**
I contact the hospital myself to find out	**Occasionally**
The patient's relatives tell me	**Often**
The patient tells me after hospital discharge	**Occasionally**

Page 6

Q6 If you received information about your patient's stay in ICU, how often would it include details about the following aspects of their critical illness?

Shock	**Occasionally**
Respiratory failure and mechanical ventilation	**Often**
Acute kidney injury requiring acute dialysis	**Often**
Acute encephalopathy / Delirium	**Often**
ARDS (acute respiratory distress syndrome)	**Often**
Neuromuscular weakness	**Occasionally**
Tracheostomy insertion	**Often**
The duration of patient's stay in ICU	**Occasionally**

Page 7

Q7 If, during a recent hospital stay, your patient was in ICU, would you record this ICU admission in the medical/surgical history section of their notes? **Yes**

Page 8

Q8 You receive a discharge summary in the post about your patient who was recently discharged from hospital after a severe illness. The summary confirms that they were in ICU during the hospital stay. Please comment on the following statements about the patient's follow-up care:

Because of the patient's ICU admission, I would make contact with them, even if the discharge summary did not request specific follow-up	**Disagree**
If the patient did not self-present to my surgery for follow-up, the ICU admission would prompt me to schedule a consultation with them	**Disagree**
If the patient did not self-present to my surgery for follow-up, the ICU admission would prompt me to schedule a consultation with the patient and a close relative	**Disagree**

Page 9

Q9 Have you ever attended an educational meeting at which you learned about the long term complications of critical illness?

No

Page 10

Q10 Are you aware of any published Guidelines about the rehabilitation of patients following hospital discharge after ICU admission with critical illness?

No

Page 11

Q11 Do you think it would benefit your patient and/or their family members if you received details about their ICU admission?

Yes,
If Yes, what the possible benefits?:
Improved awareness of significant illness, improved monitoring and follow up for physical and psychological problems

#78

COMPLETE

Collector: Web Link - Manual Entry 3 (Web Link)
Started: Wednesday, September 28, 2016 9:48:15 AM
Last Modified: Wednesday, September 28, 2016 9:49:29 AM
Time Spent: 00:01:14
IP Address: 86.145.61.199

Page 1

Q1 Which of the following best describes your GP practice?

Rural practice (outside city)

Page 2

Q2 Please indicate how long you have been working as a general practitioner.

>20years

Page 3

Q3 In which county do you conduct most of your GP work?

Cavan

Page 4

Q4 Please comment on the following statements regarding communication you receive after your patients are discharged from hospital.

I receive details of the patient's admission	**Often**
I receive details of their admission within 30days of patient's discharge	**Often**
The details I receive include whether patient was admitted to the ICU or not	**Always**

Page 5

Q5 If you receive information about your patient's ICU stay, by which method(s) would you receive this information?

The ICU/anaesthetic staff phone me directly	**Never**
In a discharge summary or letter from ICU medical team	**Never**
In a discharge summary or letter from other non-ICU medical/surgical team	**Often**
I contact the hospital myself to find out	**Rarely**
The patient's relatives tell me	**Occasionally**
The patient tells me after hospital discharge	**Always**

Page 6

Q6 If you received information about your patient's stay in ICU, how often would it include details about the following aspects of their critical illness?

Shock	**Rarely**
Respiratory failure and mechanical ventilation	**Always**
Acute kidney injury requiring acute dialysis	**Always**
ARDS (acute respiratory distress syndrome)	**Always**
Neuromuscular weakness	**Rarely**
Tracheostomy insertion	**Always**
The duration of patient's stay in ICU	**Always**

Page 7

Q7 If, during a recent hospital stay, your patient was in ICU, would you record this ICU admission in the medical/surgical history section of their notes?

Yes

Page 8

Q8 You receive a discharge summary in the post about your patient who was recently discharged from hospital after a severe illness. The summary confirms that they were in ICU during the hospital stay. Please comment on the following statements about the patient's follow-up care:

Because of the patient's ICU admission, I would make contact with them, even if the discharge summary did not request specific follow-up	**Strongly disagree**
If the patient did not self-present to my surgery for follow-up, the ICU admission would prompt me to schedule a consultation with them	**Strongly agree**
If the patient did not self-present to my surgery for follow-up, the ICU admission would prompt me to schedule a consultation with the patient and a close relative	**Agree**

Page 9

Q9 Have you ever attended an educational meeting at which you learned about the long term complications of critical illness?

No

Page 10

Q10 Are you aware of any published Guidelines about the rehabilitation of patients following hospital discharge after ICU admission with critical illness?

No

Page 11

Q11 Do you think it would benefit your patient and/or their family members if you received details about their ICU admission?

Yes

#79

COMPLETE

Collector: Web Link - Manual Entry 3 (Web Link)
Started: Wednesday, September 28, 2016 9:50:06 AM
Last Modified: Wednesday, September 28, 2016 9:51:26 AM
Time Spent: 00:01:20
IP Address: 86.145.61.199

Page 1

Q1 Which of the following best describes your GP practice?

Urban practice (Dublin, Cork, Galway, Limerick)

Page 2

Q2 Please indicate how long you have been working as a general practitioner.

10 years to 20 years

Page 3

Q3 In which county do you conduct most of your GP work?

Dublin

Page 4

Q4 Please comment on the following statements regarding communication you receive after your patients are discharged from hospital.

I receive details of the patient’s admission	**Often**
I receive details of their admission within 30days of patient’s discharge	**Often**
The details I receive include whether patient was admitted to the ICU or not	**Always**

Page 5

Q5 If you receive information about your patient’s ICU stay, by which method(s) would you receive this information?

The ICU/anaesthetic staff phone me directly	**Never**
In a discharge summary or letter from ICU medical team	**Never**
In a discharge summary or letter from other non-ICU medical/surgical team	**Often**
I contact the hospital myself to find out	**Rarely**
The patient’s relatives tell me	**Always**
The patient tells me after hospital discharge	**Always**

Page 6

Q6 If you received information about your patient’s stay in ICU, how often would it include details about the following aspects of their critical illness?

Shock	**Often**
Respiratory failure and mechanical ventilation	**Always**
Acute kidney injury requiring acute dialysis	**Often**
Acute encephalopathy / Delirium	**Occasionally**
ARDS (acute respiratory distress syndrome)	**Rarely**
Neuromuscular weakness	**Rarely**
Tracheostomy insertion	**Never**
The duration of patient’s stay in ICU	**Often**

Page 7

Q7 If, during a recent hospital stay, your patient was in ICU, would you record this ICU admission in the medical/surgical history section of their notes? **No**

Page 8

Q8 You receive a discharge summary in the post about your patient who was recently discharged from hospital after a severe illness. The summary confirms that they were in ICU during the hospital stay. Please comment on the following statements about the patient's follow-up care:

Because of the patient's ICU admission, I would make contact with them, even if the discharge summary did not request specific follow-up	**Disagree**
If the patient did not self-present to my surgery for follow-up, the ICU admission would prompt me to schedule a consultation with them	**Agree**
If the patient did not self-present to my surgery for follow-up, the ICU admission would prompt me to schedule a consultation with the patient and a close relative	**Neutral**

Page 9

Q9 Have you ever attended an educational meeting at which you learned about the long term complications of critical illness?

No

Page 10

Q10 Are you aware of any published Guidelines about the rehabilitation of patients following hospital discharge after ICU admission with critical illness?

No

Page 11

Q11 Do you think it would benefit your patient and/or their family members if you received details about their ICU admission?

Yes

#80

COMPLETE

Collector: Web Link - Manual Entry 3 (Web Link)
Started: Wednesday, September 28, 2016 9:51:44 AM
Last Modified: Wednesday, September 28, 2016 9:52:45 AM
Time Spent: 00:01:01
IP Address: 86.145.61.199

Page 1

Q1 Which of the following best describes your GP practice? **Rural practice (outside city)**

Page 2

Q2 Please indicate how long you have been working as a general practitioner. **>20years**

Page 3

Q3 In which county do you conduct most of your GP work?

Limerick

Page 4

Q4 Please comment on the following statements regarding communication you receive after your patients are discharged from hospital.

I receive details of the patient's admission **Often**

I receive details of their admission within 30days of patient's discharge **Often**

Page 5

Q5 If you receive information about your patient's ICU stay, by which method(s) would you receive this information?

In a discharge summary or letter from other non-ICU medical/surgical team **Always**

Page 6

Q6 If you received information about your patient’s stay in ICU, how often would it include details about the following aspects of their critical illness?

Shock	**Always**
Respiratory failure and mechanical ventilation	**Always**
Acute kidney injury requiring acute dialysis	**Always**
Acute encephalopathy / Delirium	**Always**
ARDS (acute respiratory distress syndrome)	**Always**
Neuromuscular weakness	**Always**
Tracheostomy insertion	**Always**
The duration of patient’s stay in ICU	**Always**

Page 7

Q7 If, during a recent hospital stay, your patient was in ICU, would you record this ICU admission in the medical/surgical history section of their notes?

Yes

Page 8

Q8 You receive a discharge summary in the post about your patient who was recently discharged from hospital after a severe illness. The summary confirms that they were in ICU during the hospital stay. Please comment on the following statements about the patient’s follow-up care:

Because of the patient’s ICU admission, I would make contact with them, even if the discharge summary did not request specific follow-up	**Neutral**
If the patient did not self-present to my surgery for follow-up, the ICU admission would prompt me to schedule a consultation with them	**Neutral**
If the patient did not self-present to my surgery for follow-up, the ICU admission would prompt me to schedule a consultation with the patient and a close relative	**Neutral**

Page 9

Q9 Have you ever attended an educational meeting at which you learned about the long term complications of critical illness?

No,
If Yes, what was the event? If No, would you attend one if it was available?:
Yes

Page 10

Q10 Are you aware of any published Guidelines about the rehabilitation of patients following hospital discharge after ICU admission with critical illness?

No

Page 11

Q11 Do you think it would benefit your patient and/or their family members if you received details about their ICU admission?

Yes,
If Yes, what the possible benefits?:
ongoing
care

#81

COMPLETE

Collector: Web Link - Manual Entry 3 (Web Link)
Started: Wednesday, September 28, 2016 9:53:15 AM
Last Modified: Wednesday, September 28, 2016 9:54:59 AM
Time Spent: 00:01:44
IP Address: 86.145.61.199

Page 1

Q1 Which of the following best describes your GP practice?

Rural practice (outside city)

Page 2

Q2 Please indicate how long you have been working as a general practitioner.

10 years to 20 years

Page 3

Q3 In which county do you conduct most of your GP work?

-

Page 4

Q4 Please comment on the following statements regarding communication you receive after your patients are discharged from hospital.

I receive details of the patient's admission	**Often**
I receive details of their admission within 30days of patient's discharge	**Often**
The details I receive include whether patient was admitted to the ICU or not	**Often**

Page 5

Q5 If you receive information about your patient's ICU stay, by which method(s) would you receive this information?

The ICU/anaesthetic staff phone me directly	**Rarely**
In a discharge summary or letter from ICU medical team	**Never**
In a discharge summary or letter from other non-ICU medical/surgical team	**Often**
I contact the hospital myself to find out	**Occasionally**
The patient's relatives tell me	**Occasionally**
The patient tells me after hospital discharge	**Often**

Page 6

Q6 If you received information about your patient's stay in ICU, how often would it include details about the following aspects of their critical illness?

Shock	**Rarely**
Respiratory failure and mechanical ventilation	**Always**
Acute kidney injury requiring acute dialysis	**Always**
Neuromuscular weakness	**Occasionally**
The duration of patient's stay in ICU	**Rarely**

Page 7

Q7 If, during a recent hospital stay, your patient was in ICU, would you record this ICU admission in the medical/surgical history section of their notes? **Yes**

Page 8

Q8 You receive a discharge summary in the post about your patient who was recently discharged from hospital after a severe illness. The summary confirms that they were in ICU during the hospital stay. Please comment on the following statements about the patient's follow-up care:

Because of the patient's ICU admission, I would make contact with them, even if the discharge summary did not request specific follow-up	**Disagree**
If the patient did not self-present to my surgery for follow-up, the ICU admission would prompt me to schedule a consultation with them	**Neutral**
If the patient did not self-present to my surgery for follow-up, the ICU admission would prompt me to schedule a consultation with the patient and a close relative	**Neutral**

Page 9

Q9 Have you ever attended an educational meeting at which you learned about the long term complications of critical illness?

No

Page 10

Q10 Are you aware of any published Guidelines about the rehabilitation of patients following hospital discharge after ICU admission with critical illness?

No

Page 11

Q11 Do you think it would benefit your patient and/or their family members if you received details about their ICU admission?

Yes

#82

COMPLETE

Collector: Web Link - Manual Entry 3 (Web Link)
Started: Wednesday, September 28, 2016 9:55:17 AM
Last Modified: Wednesday, September 28, 2016 9:56:15 AM
Time Spent: 00:00:58
IP Address: 86.145.61.199

Page 1

Q1 Which of the following best describes your GP practice?

Urban practice (Dublin, Cork, Galway, Limerick)

Page 2

Q2 Please indicate how long you have been working as a general practitioner.

>20years

Page 3

Q3 In which county do you conduct most of your GP work?

-

Page 4

Q4 Please comment on the following statements regarding communication you receive after your patients are discharged from hospital.

I receive details of the patient’s admission	**Often**
I receive details of their admission within 30days of patient’s discharge	**Often**
The details I receive include whether patient was admitted to the ICU or not	**Often**

Page 5

Q5 If you receive information about your patient’s ICU stay, by which method(s) would you receive this information?

In a discharge summary or letter from other non-ICU medical/surgical team	**Always**
The patient’s relatives tell me	**Often**
The patient tells me after hospital discharge	**Often**

Page 6

Q6 If you received information about your patient’s stay in ICU, how often would it include details about the following aspects of their critical illness?

Shock	**Rarely**
Respiratory failure and mechanical ventilation	**Often**
Acute kidney injury requiring acute dialysis	**Often**
Acute encephalopathy / Delirium	**Rarely**
ARDS (acute respiratory distress syndrome)	**Occasionally**
Neuromuscular weakness	**Rarely**
Tracheostomy insertion	**Rarely**
The duration of patient’s stay in ICU	**Rarely**

Page 7

Q7 If, during a recent hospital stay, your patient was in ICU, would you record this ICU admission in the medical/surgical history section of their notes? **No**

Page 8

Q8 You receive a discharge summary in the post about your patient who was recently discharged from hospital after a severe illness. The summary confirms that they were in ICU during the hospital stay. Please comment on the following statements about the patient’s follow-up care:

Because of the patient’s ICU admission, I would make contact with them, even if the discharge summary did not request specific follow-up	**Disagree**
If the patient did not self-present to my surgery for follow-up, the ICU admission would prompt me to schedule a consultation with them	**Disagree**
If the patient did not self-present to my surgery for follow-up, the ICU admission would prompt me to schedule a consultation with the patient and a close relative	**Disagree**

Page 9

Q9 Have you ever attended an educational meeting at which you learned about the long term complications of critical illness?

No

Page 10

Q10 Are you aware of any published Guidelines about the rehabilitation of patients following hospital discharge after ICU admission with critical illness?

No

Page 11

Q11 Do you think it would benefit your patient and/or their family members if you received details about their ICU admission?

No

#83

COMPLETE

Collector: Web Link - Manual Entry 3 (Web Link)
Started: Wednesday, September 28, 2016 9:56:34 AM
Last Modified: Wednesday, September 28, 2016 9:58:24 AM
Time Spent: 00:01:50
IP Address: 86.145.61.199

Page 1

Q1 Which of the following best describes your GP practice?

Rural practice (outside city)

Page 2

Q2 Please indicate how long you have been working as a general practitioner.

>20years

Page 3

Q3 In which county do you conduct most of your GP work?

Tipperary

Page 4

Q4 Please comment on the following statements regarding communication you receive after your patients are discharged from hospital.

I receive details of the patient’s admission **Always**

I receive details of their admission within 30days of patient’s discharge **Always**

The details I receive include whether patient was admitted to the ICU or not **Often**

Page 5

Q5 If you receive information about your patient's ICU stay, by which method(s) would you receive this information?

The ICU/anaesthetic staff phone me directly	**Never**
In a discharge summary or letter from ICU medical team	**Rarely**
In a discharge summary or letter from other non-ICU medical/surgical team	**Often**
I contact the hospital myself to find out	**Rarely**
The patient's relatives tell me	**Often**
The patient tells me after hospital discharge	**Often**

Page 6

Q6 If you received information about your patient's stay in ICU, how often would it include details about the following aspects of their critical illness?

Shock	**Rarely**
Respiratory failure and mechanical ventilation	**Often**
Acute kidney injury requiring acute dialysis	**Often**
Acute encephalopathy / Delirium	**Often**
ARDS (acute respiratory distress syndrome)	**Often**
Neuromuscular weakness	**Occasionally**
Tracheostomy insertion	**Occasionally**
The duration of patient's stay in ICU	**Occasionally**

Page 7

Q7 If, during a recent hospital stay, your patient was in ICU, would you record this ICU admission in the medical/surgical history section of their notes? **No**

Page 8

Q8 You receive a discharge summary in the post about your patient who was recently discharged from hospital after a severe illness. The summary confirms that they were in ICU during the hospital stay. Please comment on the following statements about the patient's follow-up care:

Because of the patient's ICU admission, I would make contact with them, even if the discharge summary did not request specific follow-up	**Disagree**
If the patient did not self-present to my surgery for follow-up, the ICU admission would prompt me to schedule a consultation with them	**Neutral**
If the patient did not self-present to my surgery for follow-up, the ICU admission would prompt me to schedule a consultation with the patient and a close relative	**Neutral**

Page 9

Q9 Have you ever attended an educational meeting at which you learned about the long term complications of critical illness?

No

Page 10

Q10 Are you aware of any published Guidelines about the rehabilitation of patients following hospital discharge after ICU admission with critical illness?

No

Page 11

Q11 Do you think it would benefit your patient and/or their family members if you received details about their ICU admission?

Yes,
If Yes, what the possible benefits?:
would have a more comprehensive report on their hospital stay

#84

COMPLETE

Collector: Web Link - Manual Entry 3 (Web Link)
Started: Wednesday, September 28, 2016 9:58:44 AM
Last Modified: Wednesday, September 28, 2016 9:59:43 AM
Time Spent: 00:00:59
IP Address: 86.145.61.199

Page 1

Q1 Which of the following best describes your GP practice?

Rural practice (outside city)

Page 2

Q2 Please indicate how long you have been working as a general practitioner.

<10years

Page 3

Q3 In which county do you conduct most of your GP work?

Laoise

Page 4

Q4 Please comment on the following statements regarding communication you receive after your patients are discharged from hospital.

I receive details of the patient’s admission	**Often**
I receive details of their admission within 30days of patient’s discharge	**Often**
The details I receive include whether patient was admitted to the ICU or not	**Often**

Page 5

Q5 If you receive information about your patient's ICU stay, by which method(s) would you receive this information?

The ICU/anaesthetic staff phone me directly	**Never**
In a discharge summary or letter from ICU medical team	**Often**
In a discharge summary or letter from other non-ICU medical/surgical team	**Occasionally**
I contact the hospital myself to find out	**Often**
The patient's relatives tell me	**Occasionally**
The patient tells me after hospital discharge	**Always**

Page 6

Q6 If you received information about your patient's stay in ICU, how often would it include details about the following aspects of their critical illness?

Shock	**Never**
Respiratory failure and mechanical ventilation	**Never**
Acute kidney injury requiring acute dialysis	**Never**
Acute encephalopathy / Delirium	**Never**
ARDS (acute respiratory distress syndrome)	**Never**
Neuromuscular weakness	**Never**
Tracheostomy insertion	**Never**
The duration of patient's stay in ICU	**Never**

Page 7

Q7 If, during a recent hospital stay, your patient was in ICU, would you record this ICU admission in the medical/surgical history section of their notes? **No**

Page 8

Q8 You receive a discharge summary in the post about your patient who was recently discharged from hospital after a severe illness. The summary confirms that they were in ICU during the hospital stay. Please comment on the following statements about the patient's follow-up care:

Because of the patient's ICU admission, I would make contact with them, even if the discharge summary did not request specific follow-up	**Neutral**
If the patient did not self-present to my surgery for follow-up, the ICU admission would prompt me to schedule a consultation with them	**Agree**
If the patient did not self-present to my surgery for follow-up, the ICU admission would prompt me to schedule a consultation with the patient and a close relative	**Neutral**

Page 9

Q9 Have you ever attended an educational meeting at which you learned about the long term complications of critical illness?

No

Page 10

Q10 Are you aware of any published Guidelines about the rehabilitation of patients following hospital discharge after ICU admission with critical illness?

No

Page 11

Q11 Do you think it would benefit your patient and/or their family members if you received details about their ICU admission?

No

#85

COMPLETE

Collector: Web Link - Manual Entry 3 (Web Link)
Started: Wednesday, September 28, 2016 10:00:01 AM
Last Modified: Wednesday, September 28, 2016 10:00:57 AM
Time Spent: 00:00:56
IP Address: 86.145.61.199

Page 1

Q1 Which of the following best describes your GP practice?

Rural practice (outside city)

Page 2

Q2 Please indicate how long you have been working as a general practitioner.

<10years

Page 3

Q3 In which county do you conduct most of your GP work?

Louth

Page 4

Q4 Please comment on the following statements regarding communication you receive after your patients are discharged from hospital.

I receive details of the patient's admission	**Often**
I receive details of their admission within 30days of patient's discharge	**Often**
The details I receive include whether patient was admitted to the ICU or not	**Occasionally**

Page 5

Q5 If you receive information about your patient's ICU stay, by which method(s) would you receive this information?

The ICU/anaesthetic staff phone me directly	**Never**
In a discharge summary or letter from ICU medical team	**Never**
In a discharge summary or letter from other non-ICU medical/surgical team	**Occasionally**
The patient's relatives tell me	**Often**
The patient tells me after hospital discharge	**Often**

Page 6

Q6 If you received information about your patient's stay in ICU, how often would it include details about the following aspects of their critical illness?

Shock	**Never**
Respiratory failure and mechanical ventilation	**Never**
Acute kidney injury requiring acute dialysis	**Rarely**
Acute encephalopathy / Delirium	**Never**
ARDS (acute respiratory distress syndrome)	**Never**
Neuromuscular weakness	**Never**
Tracheostomy insertion	**Rarely**
The duration of patient's stay in ICU	**Occasionally**

Page 7

Q7 If, during a recent hospital stay, your patient was in ICU, would you record this ICU admission in the medical/surgical history section of their notes? **Yes**

Page 8

Q8 You receive a discharge summary in the post about your patient who was recently discharged from hospital after a severe illness. The summary confirms that they were in ICU during the hospital stay. Please comment on the following statements about the patient's follow-up care:

Because of the patient's ICU admission, I would make contact with them, even if the discharge summary did not request specific follow-up	**Disagree**
If the patient did not self-present to my surgery for follow-up, the ICU admission would prompt me to schedule a consultation with them	**Neutral**
If the patient did not self-present to my surgery for follow-up, the ICU admission would prompt me to schedule a consultation with the patient and a close relative	**Neutral**

Page 9

Q9 Have you ever attended an educational meeting at which you learned about the long term complications of critical illness? **No**

Page 10

Q10 Are you aware of any published Guidelines about the rehabilitation of patients following hospital discharge after ICU admission with critical illness? **No**

Page 11

Q11 Do you think it would benefit your patient and/or their family members if you received details about their ICU admission? **Yes**

#86

COMPLETE

Collector: Web Link - Manual Entry 3 (Web Link)
Started: Wednesday, September 28, 2016 10:01:14 AM
Last Modified: Wednesday, September 28, 2016 10:02:10 AM
Time Spent: 00:00:56
IP Address: 86.145.61.199

Page 1

Q1 Which of the following best describes your GP practice?

Urban practice (Dublin, Cork, Galway, Limerick)

Page 2

Q2 Please indicate how long you have been working as a general practitioner.

10 years to 20 years

Page 3

Q3 In which county do you conduct most of your GP work?

Dublin

Page 4

Q4 Please comment on the following statements regarding communication you receive after your patients are discharged from hospital.

I receive details of the patient’s admission	**Often**
I receive details of their admission within 30days of patient’s discharge	**Occasionally**
The details I receive include whether patient was admitted to the ICU or not	**Occasionally**

Page 5

Q5 If you receive information about your patient’s ICU stay, by which method(s) would you receive this information?

The patient’s relatives tell me	**Occasionally**
The patient tells me after hospital discharge	**Occasionally**

Page 6

Q6 If you received information about your patient's stay in ICU, how often would it include details about the following aspects of their critical illness?

Shock	**Never**
Respiratory failure and mechanical ventilation	**Never**
Acute kidney injury requiring acute dialysis	**Never**
Acute encephalopathy / Delirium	**Never**
ARDS (acute respiratory distress syndrome)	**Never**
Neuromuscular weakness	**Never**
Tracheostomy insertion	**Never**
The duration of patient's stay in ICU	**Never**

Page 7

Q7 If, during a recent hospital stay, your patient was in ICU, would you record this ICU admission in the medical/surgical history section of their notes?

Yes

Page 8

Q8 You receive a discharge summary in the post about your patient who was recently discharged from hospital after a severe illness. The summary confirms that they were in ICU during the hospital stay. Please comment on the following statements about the patient's follow-up care:

Because of the patient's ICU admission, I would make contact with them, even if the discharge summary did not request specific follow-up	**Disagree**
If the patient did not self-present to my surgery for follow-up, the ICU admission would prompt me to schedule a consultation with them	**Agree**
If the patient did not self-present to my surgery for follow-up, the ICU admission would prompt me to schedule a consultation with the patient and a close relative	**Neutral**

Page 9

Q9 Have you ever attended an educational meeting at which you learned about the long term complications of critical illness?

No

Page 10

Q10 Are you aware of any published Guidelines about the rehabilitation of patients following hospital discharge after ICU admission with critical illness?

No

Page 11

Q11 Do you think it would benefit your patient and/or their family members if you received details about their ICU admission?

Yes

#87

COMPLETE

Collector: Web Link - Manual Entry 3 (Web Link)
Started: Wednesday, September 28, 2016 10:02:28 AM
Last Modified: Wednesday, September 28, 2016 10:04:30 AM
Time Spent: 00:02:02
IP Address: 86.145.61.199

Page 1

Q1 Which of the following best describes your GP practice?

Rural practice (outside city)

Page 2

Q2 Please indicate how long you have been working as a general practitioner.

>20years

Page 3

Q3 In which county do you conduct most of your GP work?

Donegal

Page 4

Q4 Please comment on the following statements regarding communication you receive after your patients are discharged from hospital.

I receive details of the patient's admission **Always**

I receive details of their admission within 30days of patient's discharge **Occasionally**

The details I receive include whether patient was admitted to the ICU or not **Rarely**

Page 5

Q5 If you receive information about your patient's ICU stay, by which method(s) would you receive this information?

The ICU/anaesthetic staff phone me directly	**Never**
In a discharge summary or letter from ICU medical team	**Never**
In a discharge summary or letter from other non-ICU medical/surgical team	**Often**
I contact the hospital myself to find out	**Never**
The patient's relatives tell me	**Occasionally**
The patient tells me after hospital discharge	**Occasionally**

Page 6

Q6 If you received information about your patient's stay in ICU, how often would it include details about the following aspects of their critical illness?

Shock	**Never**
Respiratory failure and mechanical ventilation	**Occasionally**
Acute kidney injury requiring acute dialysis	**Rarely**
Acute encephalopathy / Delirium	**Rarely**
ARDS (acute respiratory distress syndrome)	**Never**
Neuromuscular weakness	**Never**
Tracheostomy insertion	**Rarely**
The duration of patient's stay in ICU	**Never**

Page 7

Q7 If, during a recent hospital stay, your patient was in ICU, would you record this ICU admission in the medical/surgical history section of their notes?

No

Page 8

Q8 You receive a discharge summary in the post about your patient who was recently discharged from hospital after a severe illness. The summary confirms that they were in ICU during the hospital stay. Please comment on the following statements about the patient's follow-up care:

Because of the patient's ICU admission, I would make contact with them, even if the discharge summary did not request specific follow-up	**Disagree**
If the patient did not self-present to my surgery for follow-up, the ICU admission would prompt me to schedule a consultation with them	**Neutral**
If the patient did not self-present to my surgery for follow-up, the ICU admission would prompt me to schedule a consultation with the patient and a close relative	**Neutral**

Page 9

Q9 Have you ever attended an educational meeting at which you learned about the long term complications of critical illness?

No

Page 10

Q10 Are you aware of any published Guidelines about the rehabilitation of patients following hospital discharge after ICU admission with critical illness?

No

Page 11

Q11 Do you think it would benefit your patient and/or their family members if you received details about their ICU admission?

Yes,
If Yes, what the possible benefits?:
follow up pt closely, assess discharge health status and any psychological problems

#88

COMPLETE

Collector: Web Link - Manual Entry 3 (Web Link)
Started: Wednesday, September 28, 2016 10:04:46 AM
Last Modified: Wednesday, September 28, 2016 10:07:10 AM
Time Spent: 00:02:24
IP Address: 86.145.61.199

Page 1

Q1 Which of the following best describes your GP practice?

Rural practice (outside city)

Page 2

Q2 Please indicate how long you have been working as a general practitioner.

>20years

Page 3

Q3 In which county do you conduct most of your GP work?

Kerry

Page 4

Q4 Please comment on the following statements regarding communication you receive after your patients are discharged from hospital.

I receive details of the patient's admission	**Always**
I receive details of their admission within 30days of patient's discharge	**Always**
The details I receive include whether patient was admitted to the ICU or not	**Always**

Page 5

Q5 If you receive information about your patient’s ICU stay, by which method(s) would you receive this information?

The ICU/anaesthetic staff phone me directly	**Never**
In a discharge summary or letter from ICU medical team	**Never**
In a discharge summary or letter from other non-ICU medical/surgical team	**Often**
I contact the hospital myself to find out	**Never**
The patient’s relatives tell me	**Rarely**
The patient tells me after hospital discharge	**Occasionally**

Page 6

Q6 If you received information about your patient’s stay in ICU, how often would it include details about the following aspects of their critical illness?

Shock	**Rarely**
Respiratory failure and mechanical ventilation	**Occasionally**
Acute kidney injury requiring acute dialysis	**Occasionally**
Acute encephalopathy / Delirium	**Rarely**
ARDS (acute respiratory distress syndrome)	**Rarely**
Neuromuscular weakness	**Never**
Tracheostomy insertion	**Rarely**
The duration of patient’s stay in ICU	**Often**

Page 7

Q7 If, during a recent hospital stay, your patient was in ICU, would you record this ICU admission in the medical/surgical history section of their notes?

No

Page 8

Q8 You receive a discharge summary in the post about your patient who was recently discharged from hospital after a severe illness. The summary confirms that they were in ICU during the hospital stay. Please comment on the following statements about the patient's follow-up care:

Because of the patient's ICU admission, I would make contact with them, even if the discharge summary did not request specific follow-up	**Disagree**
If the patient did not self-present to my surgery for follow-up, the ICU admission would prompt me to schedule a consultation with them	**Disagree**
If the patient did not self-present to my surgery for follow-up, the ICU admission would prompt me to schedule a consultation with the patient and a close relative	**Disagree**

Page 9

Q9 Have you ever attended an educational meeting at which you learned about the long term complications of critical illness?

No

Page 10

Q10 Are you aware of any published Guidelines about the rehabilitation of patients following hospital discharge after ICU admission with critical illness?

No

Page 11

Q11 Do you think it would benefit your patient and/or their family members if you received details about their ICU admission?

Yes

#89

COMPLETE

Collector: Web Link - Manual Entry 3 (Web Link)
Started: Wednesday, September 28, 2016 10:07:11 AM
Last Modified: Wednesday, September 28, 2016 10:08:21 AM
Time Spent: 00:01:10
IP Address: 86.145.61.199

Page 1

Q1 Which of the following best describes your GP practice?

Urban practice (Dublin, Cork, Galway, Limerick)

Page 2

Q2 Please indicate how long you have been working as a general practitioner.

10 years to 20 years

Page 3

Q3 In which county do you conduct most of your GP work?

Dublin

Page 4

Q4 Please comment on the following statements regarding communication you receive after your patients are discharged from hospital.

I receive details of the patient’s admission	**Always**
I receive details of their admission within 30days of patient’s discharge	**Always**
The details I receive include whether patient was admitted to the ICU or not	**Occasionally**

Page 5

Q5 If you receive information about your patient's ICU stay, by which method(s) would you receive this information?

The ICU/anaesthetic staff phone me directly	**Never**
In a discharge summary or letter from ICU medical team	**Never**
In a discharge summary or letter from other non-ICU medical/surgical team	**Occasionally**
I contact the hospital myself to find out	**Never**
The patient's relatives tell me	**Often**
The patient tells me after hospital discharge	**Always**

Page 6

Q6 If you received information about your patient's stay in ICU, how often would it include details about the following aspects of their critical illness?

Shock	**Never**
Respiratory failure and mechanical ventilation	**Often**
Acute kidney injury requiring acute dialysis	**Often**
Acute encephalopathy / Delirium	**Occasionally**
ARDS (acute respiratory distress syndrome)	**Never**
Neuromuscular weakness	**Never**
Tracheostomy insertion	**Rarely**
The duration of patient's stay in ICU	**Occasionally**

Page 7

Q7 If, during a recent hospital stay, your patient was in ICU, would you record this ICU admission in the medical/surgical history section of their notes? **No**

Page 8

Q8 You receive a discharge summary in the post about your patient who was recently discharged from hospital after a severe illness. The summary confirms that they were in ICU during the hospital stay. Please comment on the following statements about the patient’s follow-up care:

Because of the patient’s ICU admission, I would make contact with them, even if the discharge summary did not request specific follow-up	**Neutral**
If the patient did not self-present to my surgery for follow-up, the ICU admission would prompt me to schedule a consultation with them	**Disagree**
If the patient did not self-present to my surgery for follow-up, the ICU admission would prompt me to schedule a consultation with the patient and a close relative	**Disagree**

Page 9

Q9 Have you ever attended an educational meeting at which you learned about the long term complications of critical illness?

No

Page 10

Q10 Are you aware of any published Guidelines about the rehabilitation of patients following hospital discharge after ICU admission with critical illness?

No

Page 11

Q11 Do you think it would benefit your patient and/or their family members if you received details about their ICU admission?

Yes

#90

COMPLETE

Collector: Web Link - Manual Entry 3 (Web Link)
Started: Wednesday, September 28, 2016 10:08:39 AM
Last Modified: Wednesday, September 28, 2016 10:09:33 AM
Time Spent: 00:00:54
IP Address: 86.145.61.199

Page 1

Q1 Which of the following best describes your GP practice?

Rural practice (outside city)

Page 2

Q2 Please indicate how long you have been working as a general practitioner.

>20years

Page 3

Q3 In which county do you conduct most of your GP work?

Galway

Page 4

Q4 Please comment on the following statements regarding communication you receive after your patients are discharged from hospital.

I receive details of the patient's admission	**Often**
I receive details of their admission within 30days of patient's discharge	**Often**
The details I receive include whether patient was admitted to the ICU or not	**Rarely**

Page 5

Q5 If you receive information about your patient's ICU stay, by which method(s) would you receive this information?

The ICU/anaesthetic staff phone me directly	**Never**
In a discharge summary or letter from ICU medical team	**Never**
In a discharge summary or letter from other non-ICU medical/surgical team	**Never**
I contact the hospital myself to find out	**Occasionally**
The patient's relatives tell me	**Often**
The patient tells me after hospital discharge	**Always**

Page 6

Q6 If you received information about your patient's stay in ICU, how often would it include details about the following aspects of their critical illness?

Shock	**Never**
Respiratory failure and mechanical ventilation	**Never**
Acute kidney injury requiring acute dialysis	**Never**
Acute encephalopathy / Delirium	**Never**
ARDS (acute respiratory distress syndrome)	**Never**
Neuromuscular weakness	**Never**
Tracheostomy insertion	**Never**
The duration of patient's stay in ICU	**Never**

Page 7

Q7 If, during a recent hospital stay, your patient was in ICU, would you record this ICU admission in the medical/surgical history section of their notes? **Yes**

Page 8

Q8 You receive a discharge summary in the post about your patient who was recently discharged from hospital after a severe illness. The summary confirms that they were in ICU during the hospital stay. Please comment on the following statements about the patient's follow-up care:

Because of the patient's ICU admission, I would make contact with them, even if the discharge summary did not request specific follow-up	**Disagree**
If the patient did not self-present to my surgery for follow-up, the ICU admission would prompt me to schedule a consultation with them	**Disagree**
If the patient did not self-present to my surgery for follow-up, the ICU admission would prompt me to schedule a consultation with the patient and a close relative	**Disagree**

Page 9

Q9 Have you ever attended an educational meeting at which you learned about the long term complications of critical illness?

No

Page 10

Q10 Are you aware of any published Guidelines about the rehabilitation of patients following hospital discharge after ICU admission with critical illness?

No

Page 11

Q11 Do you think it would benefit your patient and/or their family members if you received details about their ICU admission?

Yes

#91

COMPLETE

Collector: Web Link - Manual Entry 3 (Web Link)
Started: Wednesday, September 28, 2016 10:09:52 AM
Last Modified: Wednesday, September 28, 2016 10:11:07 AM
Time Spent: 00:01:15
IP Address: 86.145.61.199

Page 1

Q1 Which of the following best describes your GP practice?

Rural practice (outside city)

Page 2

Q2 Please indicate how long you have been working as a general practitioner.

>20years

Page 3

Q3 In which county do you conduct most of your GP work?

Clare

Page 4

Q4 Please comment on the following statements regarding communication you receive after your patients are discharged from hospital.

I receive details of the patient’s admission	**Often**
I receive details of their admission within 30days of patient’s discharge	**Always**
The details I receive include whether patient was admitted to the ICU or not	**Often**

Page 5

Q5 If you receive information about your patient's ICU stay, by which method(s) would you receive this information?

The ICU/anaesthetic staff phone me directly	**Never**
In a discharge summary or letter from ICU medical team	**Often**
The patient's relatives tell me	**Rarely**
The patient tells me after hospital discharge	**Rarely**

Page 6

Q6 If you received information about your patient's stay in ICU, how often would it include details about the following aspects of their critical illness?

Shock	**Rarely**
Respiratory failure and mechanical ventilation	**Rarely**
Acute kidney injury requiring acute dialysis	**Rarely**
Acute encephalopathy / Delirium	**Rarely**
ARDS (acute respiratory distress syndrome)	**Rarely**
Neuromuscular weakness	**Rarely**
Tracheostomy insertion	**Always**
The duration of patient's stay in ICU	**Rarely**

Page 7

Q7 If, during a recent hospital stay, your patient was in ICU, would you record this ICU admission in the medical/surgical history section of their notes?

Yes

Page 8

Q8 You receive a discharge summary in the post about your patient who was recently discharged from hospital after a severe illness. The summary confirms that they were in ICU during the hospital stay. Please comment on the following statements about the patient's follow-up care:

Because of the patient's ICU admission, I would make contact with them, even if the discharge summary did not request specific follow-up	**Disagree**
If the patient did not self-present to my surgery for follow-up, the ICU admission would prompt me to schedule a consultation with them	**Strongly disagree**
If the patient did not self-present to my surgery for follow-up, the ICU admission would prompt me to schedule a consultation with the patient and a close relative	**Strongly disagree**

Page 9

Q9 Have you ever attended an educational meeting at which you learned about the long term complications of critical illness?

No

Page 10

Q10 Are you aware of any published Guidelines about the rehabilitation of patients following hospital discharge after ICU admission with critical illness?

No

Page 11

Q11 Do you think it would benefit your patient and/or their family members if you received details about their ICU admission?

Yes

#92

COMPLETE

Collector: Web Link - Manual Entry 3 (Web Link)
Started: Wednesday, September 28, 2016 10:11:11 AM
Last Modified: Wednesday, September 28, 2016 10:20:56 AM
Time Spent: 00:09:45
IP Address: 86.145.61.199

Page 1

Q1 Which of the following best describes your GP practice?

Rural practice (outside city)

Page 2

Q2 Please indicate how long you have been working as a general practitioner.

>20years

Page 3

Q3 In which county do you conduct most of your GP work?

Mayo

Page 4

Q4 Please comment on the following statements regarding communication you receive after your patients are discharged from hospital.

I receive details of their admission within 30days of patient's discharge **Often**

The details I receive include whether patient was admitted to the ICU or not **Often**

Page 5

Q5 If you receive information about your patient's ICU stay, by which method(s) would you receive this information?

In a discharge summary or letter from other non-ICU medical/surgical team **Often**

Page 6

Q6 If you received information about your patient's stay in ICU, how often would it include details about the following aspects of their critical illness?

Shock	**Often**
Respiratory failure and mechanical ventilation	**Often**
Acute kidney injury requiring acute dialysis	**Often**
Acute encephalopathy / Delirium	**Occasionally**
ARDS (acute respiratory distress syndrome)	**Often**
The duration of patient's stay in ICU	**Often**

Page 7

Q7 If, during a recent hospital stay, your patient was in ICU, would you record this ICU admission in the medical/surgical history section of their notes?

No

Page 8

Q8 You receive a discharge summary in the post about your patient who was recently discharged from hospital after a severe illness. The summary confirms that they were in ICU during the hospital stay. Please comment on the following statements about the patient's follow-up care:

Because of the patient's ICU admission, I would make contact with them, even if the discharge summary did not request specific follow-up	**Agree**
If the patient did not self-present to my surgery for follow-up, the ICU admission would prompt me to schedule a consultation with them	**Neutral**
If the patient did not self-present to my surgery for follow-up, the ICU admission would prompt me to schedule a consultation with the patient and a close relative	**Neutral**

Page 9

Q9 Have you ever attended an educational meeting at which you learned about the long term complications of critical illness?

No

Page 10

Q10 Are you aware of any published Guidelines about the rehabilitation of patients following hospital discharge after ICU admission with critical illness?

No

Page 11

Q11 Do you think it would benefit your patient and/or their family members if you received details about their ICU admission?

Yes

#93

COMPLETE

Collector: Web Link - Manual Entry 3 (Web Link)
Started: Wednesday, September 28, 2016 10:54:44 AM
Last Modified: Wednesday, September 28, 2016 10:55:51 AM
Time Spent: 00:01:07
IP Address: 86.145.61.199

Page 1

Q1 Which of the following best describes your GP practice?

Urban practice (Dublin, Cork, Galway, Limerick)

Page 2

Q2 Please indicate how long you have been working as a general practitioner.

10 years to 20 years

Page 3

Q3 In which county do you conduct most of your GP work?

Dublin

Page 4

Q4 Please comment on the following statements regarding communication you receive after your patients are discharged from hospital.

I receive details of the patient’s admission	**Often**
I receive details of their admission within 30days of patient’s discharge	**Occasionally**
The details I receive include whether patient was admitted to the ICU or not	**Rarely**

Page 5

Q5 If you receive information about your patient's ICU stay, by which method(s) would you receive this information?

The ICU/anaesthetic staff phone me directly	**Never**
In a discharge summary or letter from ICU medical team	**Never**
In a discharge summary or letter from other non-ICU medical/surgical team	**Never**
I contact the hospital myself to find out	**Never**
The patient's relatives tell me	**Occasionally**
The patient tells me after hospital discharge	**Occasionally**

Page 6

Q6 If you received information about your patient's stay in ICU, how often would it include details about the following aspects of their critical illness?

Shock	**Never**
Respiratory failure and mechanical ventilation	**Never**
Acute kidney injury requiring acute dialysis	**Never**
Acute encephalopathy / Delirium	**Never**
ARDS (acute respiratory distress syndrome)	**Never**
Neuromuscular weakness	**Never**
Tracheostomy insertion	**Never**
The duration of patient's stay in ICU	**Never**

Page 7

Q7 If, during a recent hospital stay, your patient was in ICU, would you record this ICU admission in the medical/surgical history section of their notes? **Yes**

Page 8

Q8 You receive a discharge summary in the post about your patient who was recently discharged from hospital after a severe illness. The summary confirms that they were in ICU during the hospital stay. Please comment on the following statements about the patient's follow-up care:

Because of the patient's ICU admission, I would make contact with them, even if the discharge summary did not request specific follow-up	**Disagree**
If the patient did not self-present to my surgery for follow-up, the ICU admission would prompt me to schedule a consultation with them	**Disagree**
If the patient did not self-present to my surgery for follow-up, the ICU admission would prompt me to schedule a consultation with the patient and a close relative	**Disagree**

Page 9

Q9 Have you ever attended an educational meeting at which you learned about the long term complications of critical illness?

No

Page 10

Q10 Are you aware of any published Guidelines about the rehabilitation of patients following hospital discharge after ICU admission with critical illness?

No

Page 11

Q11 Do you think it would benefit your patient and/or their family members if you received details about their ICU admission?

Yes

#94

COMPLETE

Collector: Web Link - Manual Entry 3 (Web Link)
Started: Wednesday, September 28, 2016 10:56:07 AM
Last Modified: Wednesday, September 28, 2016 10:57:06 AM
Time Spent: 00:00:59
IP Address: 86.145.61.199

Page 1

Q1 Which of the following best describes your GP practice?

Rural practice (outside city)

Page 2

Q2 Please indicate how long you have been working as a general practitioner.

>20years

Page 3

Q3 In which county do you conduct most of your GP work?

-

Page 4

Q4 Please comment on the following statements regarding communication you receive after your patients are discharged from hospital.

I receive details of their admission within 30days of patient's discharge **Occasionally**

Page 5

Q5 If you receive information about your patient's ICU stay, by which method(s) would you receive this information?

The ICU/anaesthetic staff phone me directly	**Never**
In a discharge summary or letter from ICU medical team	**Never**
In a discharge summary or letter from other non-ICU medical/surgical team	**Occasionally**
The patient's relatives tell me	**Often**
The patient tells me after hospital discharge	**Occasionally**

Page 6

Q6 If you received information about your patient's stay in ICU, how often would it include details about the following aspects of their critical illness?

Shock	**Rarely**
Respiratory failure and mechanical ventilation	**Rarely**
Acute kidney injury requiring acute dialysis	**Rarely**
Acute encephalopathy / Delirium	**Rarely**
ARDS (acute respiratory distress syndrome)	**Rarely**
Neuromuscular weakness	**Rarely**
Tracheostomy insertion	**Rarely**
The duration of patient's stay in ICU	**Rarely**

Page 7

Q7 If, during a recent hospital stay, your patient was in ICU, would you record this ICU admission in the medical/surgical history section of their notes? **Yes**

Page 8

Q8 You receive a discharge summary in the post about your patient who was recently discharged from hospital after a severe illness. The summary confirms that they were in ICU during the hospital stay. Please comment on the following statements about the patient's follow-up care:

Because of the patient's ICU admission, I would make contact with them, even if the discharge summary did not request specific follow-up	**Neutral**
If the patient did not self-present to my surgery for follow-up, the ICU admission would prompt me to schedule a consultation with them	**Disagree**
If the patient did not self-present to my surgery for follow-up, the ICU admission would prompt me to schedule a consultation with the patient and a close relative	**Disagree**

Page 9

Q9 Have you ever attended an educational meeting at which you learned about the long term complications of critical illness? **No**

Page 10

Q10 Are you aware of any published Guidelines about the rehabilitation of patients following hospital discharge after ICU admission with critical illness?

No

Page 11

Q11 Do you think it would benefit your patient and/or their family members if you received details about their ICU admission?

No

#95

COMPLETE

Collector: Web Link - Manual Entry 3 (Web Link)
Started: Wednesday, September 28, 2016 10:57:28 AM
Last Modified: Wednesday, September 28, 2016 10:58:57 AM
Time Spent: 00:01:29
IP Address: 86.145.61.199

Page 1

Q1 Which of the following best describes your GP practice?

Rural practice (outside city)

Page 2

Q2 Please indicate how long you have been working as a general practitioner.

10 years to 20 years

Page 3

Q3 In which county do you conduct most of your GP work?

Limerick

Page 4

Q4 Please comment on the following statements regarding communication you receive after your patients are discharged from hospital.

I receive details of the patient’s admission	**Often**
I receive details of their admission within 30days of patient’s discharge	**Always**
The details I receive include whether patient was admitted to the ICU or not	**Often**

Page 5

Q5 If you receive information about your patient’s ICU stay, by which method(s) would you receive this information?

The ICU/anaesthetic staff phone me directly	**Never**
In a discharge summary or letter from ICU medical team	**Rarely**
In a discharge summary or letter from other non-ICU medical/surgical team	**Often**
I contact the hospital myself to find out	**Never**
The patient’s relatives tell me	**Often**
The patient tells me after hospital discharge	**Always**

Page 6

Q6 If you received information about your patient’s stay in ICU, how often would it include details about the following aspects of their critical illness?

Shock	**Rarely**
Respiratory failure and mechanical ventilation	**Occasionally**
Acute kidney injury requiring acute dialysis	**Occasionally**
Acute encephalopathy / Delirium	**Rarely**
ARDS (acute respiratory distress syndrome)	**Often**
Neuromuscular weakness	**Rarely**
Tracheostomy insertion	**Often**
The duration of patient’s stay in ICU	**Often**

Page 7

Q7 If, during a recent hospital stay, your patient was in ICU, would you record this ICU admission in the medical/surgical history section of their notes? **No**

Page 8

Q8 You receive a discharge summary in the post about your patient who was recently discharged from hospital after a severe illness. The summary confirms that they were in ICU during the hospital stay. Please comment on the following statements about the patient's follow-up care:

Because of the patient's ICU admission, I would make contact with them, even if the discharge summary did not request specific follow-up	**Disagree**
If the patient did not self-present to my surgery for follow-up, the ICU admission would prompt me to schedule a consultation with them	**Agree**
If the patient did not self-present to my surgery for follow-up, the ICU admission would prompt me to schedule a consultation with the patient and a close relative	**Disagree**

Page 9

Q9 Have you ever attended an educational meeting at which you learned about the long term complications of critical illness?

No

Page 10

Q10 Are you aware of any published Guidelines about the rehabilitation of patients following hospital discharge after ICU admission with critical illness?

No

Page 11

Q11 Do you think it would benefit your patient and/or their family members if you received details about their ICU admission?

Yes,
If Yes, what the possible benefits?:
follow up
care

#96

COMPLETE

Collector: Web Link - Manual Entry 3 (Web Link)
Started: Wednesday, September 28, 2016 10:59:18 AM
Last Modified: Wednesday, September 28, 2016 11:00:46 AM
Time Spent: 00:01:28
IP Address: 86.145.61.199

Page 1

Q1 Which of the following best describes your GP practice?

Rural practice (outside city)

Page 2

Q2 Please indicate how long you have been working as a general practitioner.

>20years

Page 3

Q3 In which county do you conduct most of your GP work?

-

Page 4

Q4 Please comment on the following statements regarding communication you receive after your patients are discharged from hospital.

I receive details of the patient's admission **Always**

I receive details of their admission within 30days of patient's discharge **Always**

The details I receive include whether patient was admitted to the ICU or not **Rarely**

Page 5

Q5 If you receive information about your patient's ICU stay, by which method(s) would you receive this information?

The ICU/anaesthetic staff phone me directly	**Never**
In a discharge summary or letter from ICU medical team	**Never**
In a discharge summary or letter from other non-ICU medical/surgical team	**Rarely**
I contact the hospital myself to find out	**Never**
The patient's relatives tell me	**Often**
The patient tells me after hospital discharge	**Often**

Page 6

Q6 If you received information about your patient's stay in ICU, how often would it include details about the following aspects of their critical illness?

Shock	**Never**
Respiratory failure and mechanical ventilation	**Never**
Acute kidney injury requiring acute dialysis	**Never**
Acute encephalopathy / Delirium	**Never**
ARDS (acute respiratory distress syndrome)	**Never**
Neuromuscular weakness	**Never**
Tracheostomy insertion	**Never**
The duration of patient's stay in ICU	**Never**

Page 7

Q7 If, during a recent hospital stay, your patient was in ICU, would you record this ICU admission in the medical/surgical history section of their notes? **No**

Page 8

Q8 You receive a discharge summary in the post about your patient who was recently discharged from hospital after a severe illness. The summary confirms that they were in ICU during the hospital stay. Please comment on the following statements about the patient's follow-up care:

Because of the patient's ICU admission, I would make contact with them, even if the discharge summary did not request specific follow-up	**Agree**
If the patient did not self-present to my surgery for follow-up, the ICU admission would prompt me to schedule a consultation with them	**Disagree**
If the patient did not self-present to my surgery for follow-up, the ICU admission would prompt me to schedule a consultation with the patient and a close relative	**Disagree**

Page 9

Q9 Have you ever attended an educational meeting at which you learned about the long term complications of critical illness?

No,
If Yes, what was the event? If No, would you attend one if it was available?:
Yes

Page 10

Q10 Are you aware of any published Guidelines about the rehabilitation of patients following hospital discharge after ICU admission with critical illness?

No

Page 11

Q11 Do you think it would benefit your patient and/or their family members if you received details about their ICU admission?

Yes,
If Yes, what the possible benefits?:
psychological support

#97

COMPLETE

Collector: Web Link - Manual Entry 4 (Web Link)
Started: Wednesday, September 28, 2016 1:41:02 PM
Last Modified: Wednesday, September 28, 2016 1:42:10 PM
Time Spent: 00:01:08
IP Address: 86.145.61.199

Page 1

Q1 Which of the following best describes your GP practice?

Urban practice (Dublin, Cork, Galway, Limerick)

Page 2

Q2 Please indicate how long you have been working as a general practitioner.

>20years

Page 3

Q3 In which county do you conduct most of your GP work?

-

Page 4

Q4 Please comment on the following statements regarding communication you receive after your patients are discharged from hospital.

I receive details of the patient's admission	**Occasionally**
I receive details of their admission within 30days of patient's discharge	**Always**
The details I receive include whether patient was admitted to the ICU or not	**Always**

Page 5

Q5 If you receive information about your patient's ICU stay, by which method(s) would you receive this information?

The ICU/anaesthetic staff phone me directly	**Never**
In a discharge summary or letter from ICU medical team	**Never**
In a discharge summary or letter from other non-ICU medical/surgical team	**Always**
I contact the hospital myself to find out	**Always**
The patient's relatives tell me	**Often**
The patient tells me after hospital discharge	**Often**

Page 6

Q6 If you received information about your patient's stay in ICU, how often would it include details about the following aspects of their critical illness?

Shock	**Always**
Respiratory failure and mechanical ventilation	**Always**
Acute kidney injury requiring acute dialysis	**Often**
Acute encephalopathy / Delirium	**Occasionally**
ARDS (acute respiratory distress syndrome)	**Occasionally**
Neuromuscular weakness	**Occasionally**
Tracheostomy insertion	**Occasionally**
The duration of patient's stay in ICU	**Occasionally**

Page 7

Q7 If, during a recent hospital stay, your patient was in ICU, would you record this ICU admission in the medical/surgical history section of their notes? **Yes**

Page 8

Q8 You receive a discharge summary in the post about your patient who was recently discharged from hospital after a severe illness. The summary confirms that they were in ICU during the hospital stay. Please comment on the following statements about the patient's follow-up care:

Because of the patient's ICU admission, I would make contact with them, even if the discharge summary did not request specific follow-up	**Strongly agree**
If the patient did not self-present to my surgery for follow-up, the ICU admission would prompt me to schedule a consultation with them	**Strongly agree**
If the patient did not self-present to my surgery for follow-up, the ICU admission would prompt me to schedule a consultation with the patient and a close relative	**Agree**

Page 9

Q9 Have you ever attended an educational meeting at which you learned about the long term complications of critical illness?

No

Page 10

Q10 Are you aware of any published Guidelines about the rehabilitation of patients following hospital discharge after ICU admission with critical illness?

No

Page 11

Q11 Do you think it would benefit your patient and/or their family members if you received details about their ICU admission?

Yes
