# Supplementary material for: Information sharing between intensive care and primary care after an episode of critical illness; A mixed methods analysis
Source: PLoS One. 2019 Feb 28;14(2):e0212438. doi: 10.1371/journal.pone.0212438 (PMC6394993; doi:10.1371/journal.pone.0212438)
Supplement: S1 File — (DOCX) [file pone.0212438.s005.docx]

**Consultant interviews:**

**FICP: ICU getting patient info from GP**

Very often our patients come to it with a complex medical history and they’re on numerous medications and often the GP is aware of all of this history (XXX page 1)

I think one of the first places where we can find reliable information is the GP (XXX page 1)

… rang the GP to go through what was going to be done and what wouldn’t be done and to verify the advanced care directive. (XXX page 1)

the GP would have in summary about – that can tell us a little bit in summary about the patients previous conditions, which helps us in treatment and also the medication that he is on (XXX page 1)

we can have talk with the GP and we can explain it to them that this is a thing that can happen (XXX page 1) [concept that we can prepare GP for what the problems patient may have after hospital discharge]

by and large the GP will have the full medical history, the background, often social information about the patient that we don’t have here in the Intensive Care Unit (XXX page 1)

And equally, physicians who are hospital based and seeing the patient maybe on a sort of a yearly basis or a six monthly basis probably don’t have that same relationship that a General Practitioner does. (XXX page 1) [added benefit of GP over referring primary hospital physician]

…it certainly could maybe drive the direction of therapy. We don’t know frequently, nor do families, what are the patient’s own – and particularly elderly patients, we don’t know what their wishes are in relation to resuscitation, in relation to admission to Intensive Care. (XXX page 1)

particularly for the patients that we are looking at, coming through the Emergency Department with a direct admission to Intensive Care, there is nobody really in the hospital who had any prior knowledge of the patient. (XXX page 1) [particular importance for patient with “direct” admission to ICU]

One of the main determinants of whether you’ll be admitted *(to ICU)* is about your pre-morbid … your functionality and how you’re able to cope with your ADLs and stuff like that.(XXX page 1) [concept that this info is often only – or best – available from patient’s GP]

…emergency admissions I suppose… GPs are not available. But at the same time, yeah, if they’re available they’re useful.(XXX page 1) [problem with ICU being an emergency 24-hour service and how this hinders early GP communication]

**FICP: Who calls the GP to get patient information?**

Pharmacist (XXX page 1)

Junior doctor (XXX page 1)

**PBIS: Comment about why ICU should contact GP to give information about patient**

… it means that the patient is critically ill and in the community that’s important information for the family doctor to have. (XXX page 1)

…the impact of critical illnesses is on the, sort of the chronic scale and there is no doubt that there is a huge impact there. (XXX page 2)

Well, I suppose it gives a bigger picture of the patient’s overall journey. (XXX, page 1)

… they(GPs) see the bigger picture more and the impact of ICU is multisystem in the longer term as well as the short term. (XXX page 2)

And it’s all part of this business of, like, you know, the gap between acute hospitals and primary care is big anyway. It’s going to be massive between the most acute part of it, which is ICU and primary care. So why not just cut out the middle bit? Because there’s bound to be dilution. (XXX page 3) [Concept in literature of loss of information during transitions of care]

Whereas GPs are more total body doctors really, aren’t they? (XXX page 2) [interesting concept that ICU doctors and GPs are both “total body doctors” – my thought not XXX’s]

With regards to muscle weakness or social needs and psychological impact and all that sort of thing… they’re better dealt with all round good solid GP, I would have thought. (XXX page 2)

…at the very least it is an opportunity to have a discussion about ongoing end of life decisions, because there probably is a marker… And I think definitely the GPs are the best place there. (XXX page 2) [ie. For GP to have a red flag to alert them to those discussions with the patient]

I think it’s a flag that someone is in a very high risk group. (XXX page 2)

…we definitely can offer them a proper discharge summary from ICU, outlining the ICU component of the patient’s hospital stay. And the likely complications thereof. (XXX page 1)

For example, if you have a patient who is a long stay patient in ICU who has a polymyoneuropathy and who is likely to have residual effects, I find that the discharge summaries that are done by the primary teams, often don’t even mention that the patient was in ICU. (XXX page 1) [concept that important information about residual pathology may be missing from primary team summaries]

…for example a patient, say a patient is in a long-term patient in the ICU, who ends up having a Post Traumatic Stress reaction. And we know from the literature now that that is not uncommon and that while patients immediately after their discharge from ICU frequently don’t remember very much about the admission. That over time, they often have flashbacks. (XXX page 2)

… or indeed informing the GP that their patient is in Intensive Care. So they are not sort of door stepped by an anxious family member or next of kin. And the GP is sort of not even aware that the patient is or has been in Intensive Care. (XXX page 2)

And I think that it would benefit the GP knowing that the patient had had an Intensive Care stay. And what the major interventions were and any potential complications that they should be aware of. (XXX page 2) [concept that ICU contact would help GP flag a high risk patient with potential complications]

I am just talking the actual discharge summary page, the problem list. But I also do think that we would, for the individual patient have to identify future problems that could arise. (XXX page 3)

It is a two-way process… (XXX page 6)

**SOIS: How should information be sent to GP about their patient in ICU?**

…if there was a way that the secretary could just put through a phone call or if we had a collection of emails. (XXX page 2)

We don’t actually have discharge letters. (XXX page 2)

…one of our goals over the next five years is to work towards an electronic system…When that comes in then I think the discharge letter would be a great way of doing it. (XXX page 2)

(Regarding electronic discharge letters from ICU to the ward team)… just cc the GP at the same time so they can at least track the patient’s progress, yeah. (XXX page 2)

I think the best way to do it would be, you know, if our secretaries could just dedicate maybe 10/15 minutes in the morning to just sending an email to whichever practice the patient belongs to. (XXX page 3)

The phone call will be better in the sense that you’ll speak to somebody and you’ll know the message has got through. (XXX page 3)

But I think the phone call or the email are more efficient and they are a better line of communication. Because the discharge letter won’t get to them until some time after the ICU discharge. (XXX page 3) [phone call and email in real time, phone enables comfirmation that message received, discharge letter may not arrive in time to the GP]

I suppose, you know, making contact with the GP when the patient comes in initially might be an outset, we could start from the outset… (XXX page 4)

… maybe if we wrote discharge summaries and sent them to GPs separate to the actual hospital discharge letter, that could be a way, I suppose. (XXX page 4)

I mean when you write a discharge summary, let’s say from the ICU, perhaps we should be writing it to the GP as well as to the hospital team. (XXX page 5)

I feel that there should be a separate discharge letter from the ICU, detailing what the ICU course and what the treatment we did. (XXX page 2)

I think a letter is a way of communicating with the GP, a discharge letter. And that will tell in short about what the patient had in the ICU. (XXX page 3)

…he will register the brief information but the total information may not be with him. (XXX page 5) [relates to transitory nature of information delivered by phone]

I think it is probably best that ICU relays that information at the time of the discharge. (XXX page 2) [see comment about relatives “door-stepping” GPs who do not have necessary information]

I think if we had enter into a formal letter writing correspondence, I just don’t think we have the support at the moment, the administrative support available to us to do that. (XXX page 3)

Well time is an issue for most GPs. I would have to take sort of the advice of the GP group to say do they want to be called. I mean…only a small number…of their patients will end up in an Intensive Care, at any given time. So, the other option would be to send the discharge and invite them to call us. (XXX page 3) [discussion about whether ICU should directly call GPs about their patients in ICU]

We could put in a phone call to the practice to say “Mr So-and-so has been admitted to the ICU and we will send a discharge. His current problems are…” And we will send a summary of his course of events when he is discharged. (XXX page 5)

I mean I suppose my preferred method of communication now is by email but we don’t like to pass sensitive information over email. (XXX page 5)

So like potentially something that’s translated to communicate via Health Link. (XXX page 6)

I think it should be at the end of ICU because there’s so many unknowns at the time of admission. I think a summary at the end of the episode is useful. (XXX page 6)

**SOIS: Should primary team communicate with GP about ICU care?**

I think it should be our responsibility to do that. (XXX page 3)

… just simply keeping track of all those patients and which wards they’ve gone to and what tests they’ve had done is difficult. Only a small number of patients are admitted to critical care. (XXX page 3) [ICU patients comprise small fraction of total primary team workload so not reasonable to expect them to communicate with GP]

… but to my mind while they’re in ICU, they’re yours and the whole patient journey is important. (XXX page 1)

…the primary people that they would write that the patient was admitted into ICU without writing about the course in the ICU. (XXX page 2) [Primary team might make brief reference to ICU stay but not provide enough detail to help GP in the post-discharge patient management]

The discharge summaries now that go out with the patient or that are sent immediately to the GPs tend to be very cursory, there is a tick box but they get the principal diagnoses, the discharge medications. (XXX page 3)

I don’t think they’d have enough time…or insight or access to the information to be able to summarise adequately the IC Unit. (XXX page 2)

**PBIS: Potential benefits for patient of having better communication**

I think it might help generate some conversation with the patient. For example if the patient has many chronic diseases and is slowly deteriorating I think it may generate that conversation about how the patient wants to proceed when they get unwell again. (XXX page 4) [stimulate end of life discussions]

And then to find the follow up services, whether they’re rehabilitation services, whether they’re community services, and very often those services are accessed through a GP. (XXX page 5) [GPs armed with information to direct post-discharge supports for patient]

I’m sure there are lots of things that get initiated and started in ICU that need follow up. (XXX page 3) [related to drugs such as amiodarone being commenced in ICU and not stopped prior to discharge)

And from the patient’s point of view that missing piece, that gap in their life, I think it might help to know that they could communicate with someone or someone could communicate through the GP. (XXX page 2) [concept that patient is amnestic for their ICU stay and informing GP about the details would equip them with the knowledge needed to speak with their patient and help them make sense of the experience]

*…an informed GP might be better able to sort of manage and cope with and help the post ICU patient than an uninformed GP.* (XXX in XXX page 4) XXX emphatically agrees with this.

…the topic that it is more about the cognitive impairment and functional limitations that the patient would experience. (XXX page 2) [preparing the GP for the post-discharge problems likely to arise]

*“So, maybe what you are suggesting is that the GP is able to give them some reassurance because they have insight and they understand what has gone on.* Yes.” (XXX page 2)

Oh, yeah, I think so, yeah for sure. It’s going to inform the GP’s judgement with more insight into what’s going on and what the patient is liable to suffer, or has suffered already. So they’re going to make better informed decisions… (XXX page 4)

…if it’s there, and it’s popping up on a screen in front of a GP, you know, it’s less likely to slip through the net. (XXX page 4) [concept that important post-ICU information, if sent to GPs, can be used by then to alert them to ongoing current medical issues]

**PBIS: Lack of benefit of having better communication**

I think not all general practitioners understand the long term sequelae. (XXX page 4) [Communication useless if insight/knowledge about how to use the communicated info is lacking]

So having those lines of communication open is great if one has the resources for it. (XXX page 4) [Communication useless if resources not available to act on info communicated – XXX thinks they are largely absent]

…it can be very misleading then, you know, if you’re a GP looking at a patient who has just walked in and you look at the records that’s in the computer, there’s all these tests, diagnoses, and you don’t know how much weight there is to different things. (XXX page 30 [issue that there may be too much information in ICU summaries that they GP may not be able to decipher and apply to patient care]

**PBIS: Benefit (or not) for relatives of better communication**

…you know, it’s going to take you months to recover from this thing and it could have psychological and social impacts and, you know, they’re expecting, you know yourself when you have the conversation and they say – well what day can we expect them to be out of there? (XXX page 5) [GP info would help relatives understand the longevity of recovery and set realistic expectations]

…or indeed informing the GP that their patient is in Intensive Care. So they are not sort of door stepped by an anxious family member or next of kin. And the GP is sort of not even aware that the patient is or has been in Intensive Care. (XXX page 2)

The families will remember them, but sometimes they would like to, I think they would often like to just put the ICU episode behind them. *Behind them, yeah. And are you suggesting if they had a bit more information and the GP was able to explain what had happened, it might help them put it behind them?* They may have questions, yeah. (XXX page 4) [concept that info to the GPs may help relatives gain closure on the ICU experience – relatives have a more detailed memory of the experience than patients]

I don’t think that the relative, other than sort of looking after their own needs at a time of stress, has any role in the communication. And that is what I meant by sort of door stepping the GP with the information that their patient is in the ICU. Is sort of puts them on the back foot then, that they don’t know. (XXX page 6) [takes the onus of relatives to be the primary conduit of information]

…you know, it’s very difficult to expect any GP to go answering questions, you know, for somebody else if somebody is in the hospital. You know, like in other words the GP answering questions on behalf of the ICU team. (XXX page 5) [Two issues raised here: issue of confidentiality, talking about ICU patient with relative / and GP expected to have medical insight to discuss ICU treatments with patient’s relative]

**Emergent: GP involved in decision making in the ICU**

…in terms of having the GP come into an Intensive Care Unit and make decisions I think that might be a bridge too far. (XXX page 5)

You may run into similar scenarios if you’ve a GP who has the ability to take decisions in an ICU. (XXX page 6) [describing conflicts and disagreements about patient management in ICU]

**Emergent: Other comments that need coding**

… I already find it challenging to ensure that the primary team gets a phone call from one of our doctors to let them know that the patient is coming onto the ward and will now be under their care. That is something is supposed to happen and I’ve said it again and again but it doesn’t always happen. (XXX page 2) [Compliance a problem with any system)

(Regarding electronic discharge letters in a previous job)… the teams would automatically look for that on the ward and it really helped communication.(XXX page 2) [Making something routine is the key so it is “automatic”, aided by electronic system)

…whereas if it became accepted practice, it would just be accepted practice. (XXX page 5) [another reference to making it routine]

And some of our patients are actually going home from ICU because of the bed problems. (XXX page 3) [a product of bed issues and one directly impacting on GP-ICU communication]

Well I think the first step really is having a follow up clinic.. I think that’s a very good idea. (XXX page 4)

GPs are at the heart of their community. (XXX page 5) [a key statement that demonstrates how they need the necessary information to have this role in the community]

…and the whole patient journey is important. So what we’re aiming for is not just to get them out of ICU but to get them to the best place that they should be and the best level of care they should be. (XXX page 1) [concept of patient journey, and that ICU – and post-ICU hospital stay – is only a part of the overall journey, the destination being home ideally]

…I mean the ICU experience is probably the most invasive and traumatic part of their hospital stay… (XXX page 3)

People carry around a lot of things that they can’t square away. (XXX page 4) [after a stay in ICU, or after a relative has been in – or has died in – the ICU: all XXX’s thoughts not mine]

We don’t see the fact that they can’t do what they used to be able to do, that they’re weak. (XXX page 5) [ICU staff blinkered to patients’ functional limitations post-ICU]

We’ve thought for a long time of having, like, you know, an MDT on for the longer term patients. But a way of kind of bringing people into the fold could be to invite the GP into that… (XXX page 6)

…coming in the other direction, most of the time anyway, you’re just depending on whatever somebody puts on the discharge form… most of the time you won’t have any insight into the complexity of what happened in ICU. (XXX page 2) [XXX was a prior GP and refers here to the dependence on the discharge summary for getting info about a patient’s hospital stay]

*…perhaps if you were going to send an interim summary from ICU to GPs it possibly shouldn’t actually include medicines at all. Because there’s going to be some medicines there that perhaps aren’t even relevant and may not be the.* Yeah, that’s true. (XXX page 2) [XXX raises point that interim reports may carry unnecessary or incorrect information to the GP so content of them should be selected carefully to avoid errors or misleading information]

*So, not computer generated?* Well I don’t know, you could end up with 10 different diagnoses coming out of ICU, you know like myopathy and pneumonia and delirium and whatever ways you code for all that type of thing. (XXX page 3) [concept that discharge summaries might be better written by medical staff rather than computer/code-generated, leading to a lot of unedited information] [information needs to be delivered *and put in context* for the GP perhaps – my thought]

But like the problem with most things that’s generated by computers, because that’s all that’s in ICU now, is non-configured information. You know?... It needs to be in a format that’s digestible in the context that you’re working in… (XXX page 6) [see issue above about computer-generation of information]

**GP interviews**

**FICP/SOIS: Comment about how much a GP learns about their patients’ stay in the ICU during a recent hospital admission**

Very little, is the answer, very little. (XXX page 1)

…in truth we would know very, very little about it. We might know that they were in Intensive Care or we might not. And that’s about the extent of what we’d know most of the time. Which isn’t very helpful, to be honest, but that’s the way it is. (XXX page 1)

you don’t really know what went on in there, to be truthful. (XXX)

You might have kind of a broad sweep about what the issue was, you know, we’ll say they had septicaemia or they’d had an aneurism repair or they’d had a major surgery of some sort. You might just have a broad brush stroke as to what the nature or the overall reason they were in Intensive Care was but little or no detail as to what went on when they were there. (XXX page 1)

Generally no (XXX page1) [answer to Q: do you know if they have been in ICU or not?]

Not from the hospital at all….almost never(XXX page 1)[would you hear much about the stay in ICU or not?]

No, never, I don’t ever remember getting anything from an Intensive Care team. (XXX page 2)

I don’t think that it is often specifically mentioned (XXX page 1)

Never that I can think of (XXX Page 1) [to question about getting direct communication from ICU staff]

…generally not. Once I think. (XXX page 1)

**PBIS: Potential benefits (or lack thereof) for GPs (and patients) of having more info about ICU stay**

we could do with a little more information than we’re being given, to be honest…it would be useful to discuss with people, you know, what went on, to a certain extent, and how they reacted to it (XXX page 1-2) [potential psychological benefit for patients if GP had knowledge to inform these discussions]

…at least you kind of clear up your own thinking and in turn you clear up their thinking a bit; you inform them a little bit about it. (XXX)

…we might make contact and see how they are and that there, and sometimes visit people in the hospital there if they’re local. (XXX) New concept that early notification to GP might prompt them to seek more info themselves.

…there’s the perception that you don’t care,…it’s someone you’ve looked after for multitudes of years…but you’re just unaware that they’re in. [ICU] (XXX) Other new concept that lack of info affects patients’/relatives’ perception of GPs’ care

an acute kidney injury is a kind of a marker for maybe a chronic injury down the road… So yeah, I mean it *(ICU admission)* does have some issues down the line for ongoing care. (XXX page 2)

…in relation to the delirium, I mean that would be a marker for, you know, possibly cognitive impairment down the line and you would kind of say to yourself – well, right, maybe we should do a mental test score on this person just to document it and have it and see down the line is that slipping... (XXX page 3)

…you would look at the medications that they’re on in the light of the problems that have been highlighted here.(XXX page 3)

if they’re not turning up I would certainly send for them after a couple of weeks and see what the story is. (XXX)

…legal issues and making wills and all that sort of stuff…at least if you know that there was some *(cognitive)* impairment, albeit acute, you know, if solicitors do write to you, again you would be kind of wary of it… (XXX page 4) [legal implications for GPs and their patients of knowing about patient’s capacity post-ICU discharge]

There’s no point highlighting it if you can’t deal with it. (XXX page 6) [refers to futility of having info if the lack of resources or GP skills hinders using that info to benefit patients]

It’s not that the hospitals don’t explain, there’re doctors at the hospital that sit down at the side of the bed and explain things in great detail, but it goes in one ear and out the other. Now, they’ll come in here and sit in the surgery where they’re familiar and they’ll imbibe an awful lot of information.(XXX page 4)[GPs have more time to speak with patients, and patients may be more receptive to information with their familiar GP]

…at least we’re forewarned and forearmed. (XXX page 2)

*And of course by the time the patient arrives in your practice, perhaps five or six or seven days after the discharge, armed with a prescription, that information is already there.* It’s already there.(XXX page 5)[regarding info sent by HealthLink]

I think that’s the type of information – that should be communicated because that puts us on alert to say right, this was a very ill person. They weren’t just somebody who had pneumonia, they actually went into shock or something else happened. So therefore there could be other problems that we need to know about, that we need to deal about, like if they go into renal failure you may or may not get that information. (XXX page 1)

So if we had that information we are much more then in a better position maybe to help them on the road and see maybe what needs they have and set realistic goals for them. (XXX page 5)

…I mean you want to know if your patient is about to leave or is seriously ill because you might have family ringing you and saying, ‘Did you know?’(XXX page 8)

…maybe most of the time they[patients] are not aware but they will be coming out of a hurricane of sickness and it’s important that, I’m sure they would appreciate that I would have known about that… (XXX page 5)

**PBIS/FICP: What GPs might do differently if they had more info about ICU stay**

…there would be a certain number that you would, you know, you’d say to the secretary “Ring them and tell them I want to see them in four or five days’ time.” So, people who’ve been in intensive care, if you knew about it, would be one of those.(XXX page 3)

..if they’re not turning up I would certainly send for them after a couple of weeks and see what the story is. (XXX page 3)

…in relation to the delirium, I mean that would be a marker for, you know, possibly cognitive impairment down the line and you would kind of say to yourself – well, right, maybe we should do a mental test score on this person just to document it and have it and see down the line is that slipping... (XXX page 3)

…you would look at the medications that they’re on in the light of the problems that have been highlighted here.(XXX page 3)

“Look, your kidneys have recovered pretty well but you need to be mindful of it and be sure and tell somebody else if you happen to see somebody else.”… You could put them in the picture (XXX page 4) [concept that info from ICU would help a GP keep the patient informed about their illnesses and the impact of those illnesses]

…you could say to them, you know, it was after your big operation, you had an aneurism repaired, that situation is not likely to recur…you’re OK now. Whereas, if it was some other sort of a situation where, you know, it might recur you could say to them “Well look, that illness might well recur and you’d want to be mindful and if you begin to feel unwell or you start to develop whatever, that you need to come fairly quickly and be assessed.” (XXX page 4-5)

…the neuromuscular stuff, OK, you’re probably into physiotherapy and that, funnily enough, is available in primary care teams. (XXX page 6)

…it would come in the email and you will just read it, download it, integrate it into the patient’s file and it would be there when they come in. (XXX page 8)

…they *(GPs)* would have to, or should offer these people a review appointment, if say they don’t turn up within a week of discharge from hospital, they probably should offer them a review appointment and probably make it, instead of a ten minute job, that it would be a twenty minute appointment, at a fairly quiet time and just go through it with them. (XXX page 8)

…well then at least I know how sick they were. So at least I can then tread a lot more carefully than I would if they had walked in off the street…(XXX page 4) [if GP had info about ICU stay]

…it alerts me to be that little bit more watchful of the red flag signs as they say. As I said knowledge is king, knowledge is power. If we have the knowledge it makes us more aware of things to look out for.(XXX, page 4)

…post-stay supportive and management of any later complications depending upon what the nature of the beast was, you know…it would be more from the supportive side for either the patient or the family.(XXX page 3)

So what you’re going to do is you’re going to put it into a framework for them, say “No you’re not going to be OK in a week, you’ll be lucky if you’re alright in six months”. Set a kind of a realistic target for them so they’re not into you saying, “I’m still feeling desperate”, that there’s a reason for it. (XXX page 3)

…probe a little bit more closely and probably be a little bit more proactive…(XXX page 5)

**SOIS: GP opinion about what information is required about the ICU stay**

Just what happened, what the consequences were, and are there any ongoing consequences.. (XXX page 2)

…if somebody has had an acute kidney injury, that may have implications down the road in terms of possibly developing a chronic kidney issue, which if we’re not told about it we’re never probably going to pick it up or even look for it.(XXX page 2)

…we could do with a bit more info as to why they were in there and the issues that arose consequent to being in there. (XXX page 2)

Maybe the duration of their stay there, it’d be worth knowing how long they were in there for. (XXX page 2)

…that we could just know why they were in there, what their consequences were, what we are to look out for… (XXX page 8)

…you would only have to just put in the four or five issues that have arisen and the overall diagnosis and that would be enough. And maybe suggestions for follow-up if there were suggestions, of you know, check creatinine in a fortnight or refer for psychology or whatever you think would be appropriate. (XXX page 8)

I think the fact of being in there would be helpful...I don’t know to know the in-depth details is a requirement…the fact of somebody being in and the dying notice is, I think, a critical point for GPs… (XXX page 3)

It’s instantaneous and it’s a two-liner, admitted with bronchial pneumonia, discharged to Cherry Ward, under care of chest physician or whatever. That’s all I need.(XXX page 5) [about content that would be sent by HealthLink]

…what we need to know, what was the confirmation of diagnosis and what is the level of care that this patient needed and what’s their after-care? Because we are the people who are going to be looking after her…(XXX page 3)

…whether ICU would consider kind of doing their own discharge and say “Well this lady spent, you know, this period of time in intensive care, her problems were as such, this is what we did and this is the follow up. She’s now with the medical people…” and that’s it. (XXX page 6)

Tell us they were there and what happened to them and what you can anticipate in the future. (XXX page 7)

…I suppose how long they were in it for a start, why and what’s the prognosis I suppose basically in a nutshell… how they are likely to recover from this (XXX page 2)

**SOIS: Comments about how (format, timing and medium) information should be delivered to GPs**

…this secure email? This Healthlink thing that is available. (XXX, page 7)

I think if there was a fairly straightforward, pro forma, A4 sheet that could be filled in when the patient leaves Intensive Care… if that could be sent via the Healthlink email system to GPs from the appropriate consultant who would just have to have a Healthlink email address…that would do it. (XXX, page 7-8)

…that would be contingent on us actually knowing that they came out of hospital in the first place, at the end of the day. Now, clearly we know eventually but I mean the discharges come weeks, months later which isn’t any great help. (XXX page 8) [useless ness of late discharge summaries]

…if we got something to know they were in Intensive Care so that when you are discharging them out, we know they are gone to an open ward and here are the issues. (XXX page 9) [suggestion that ICU info come to GP when patient leaves ICU instead of when they leave hospital]

Come directly to us as soon as you discharge the patient to the other physician; why not send us a letter out to say that you’ve done that… go through a thing called Health Link.(XXX, page 3) [and the concept that HeathLink notes can be easily incorporated into a patient’s electronic chart]

If you’re writing one letter ask one of the interns copy that and send it to the GP please! (XXX page 5)

I don’t need the big chief in ICU to be ringing me; I’ll take it from anybody just to know that they’re there. Then I will phone the nurse and say listen, are you looking after, can you tell me what the condition is. (XXX page 2)

I think that the onus would be on the most junior member of staff or a student nurse to say who is the GP of this guy who died and a phone call or an email.(XXX page 4)

…the medical information of someone who’s you’re finished with them, you’re discharging them to the ward, I think should come via Healthlink…(XXX page 5)

But the phone call, if you’re asking my opinion, yeah, that would be the number one, two and three if I could get it. (XXX page 9)[as a way of notifying the GP of a major event occurring – ie. The stay in ICU]

Using healthlink (XXX page 2-3) [not a quote but a statement]

…some kind of a letter or a plan clearly written, you know, your patient is at risk of, we won’t be seeing them again, you know, can you do this… (XXXpage 4)

…probably a letter as they leave, you know, or a note, just a summary probably. (XXX page 2)

…I’d love phone calls in that case yeah and certainly it would, yeah but that would be putting a bit of pressure on you guys of course ,yeah (XXX page 7)

**PBIS: Comments about the implications of ICU-GP communication on patients’ relatives**

…it’d be very nice to say well we could have a long conversation with the relatives as well and we could but truth is time probably wouldn’t permit too much of that, to be honest. (XXX page 5)

…my view about the relatives is if they’re not sure what’s going on in intensive care, they’re in a position to ask *(the ICU staff)*. (XXX page 5)

Third hand information is lousy, they never get it right. Always misinterpret it. (XXX page 5) [thinks relatives as a conduit of information are unreliable]

Mother and wife came in and long faces and I didn’t know what had happened there and had I not heard the news of the catastrophic brain injury peri-operatively one week before.(XXX page 2)

…at least we’re forewarned and forearmed. (XXX page 2)

…a phone call I know exactly who I spoke to, where they are, what’s wrong and I’m armed and then if I know the family well or feel look, I need to get involved here, I’ll then phone the family and say can I do anything, can I help with anything. (XXX page 9)

We’re looking at the whole package and the ramifications there, psychologically, children minding wise, you know, work wise… (XXX page 9)[concept of dealing with the social and domestic implications of critical illness]

The patients are very ill, that’s the priority. And that can actually, how would you say, de-escalate or kind of deflate people getting very anxious and, oh I’m making a complaint for this, that and the other. (XXX page 6) [concept that GPs could help relatives understand an episode of critical illness and perhaps reduce stress and litigation]

**FICP: GP opinion about the role of education for GPs about implications of critical illness**

I think there is a role, certainly…yeah I think there is, to be honest. And I think it would be something that would be useful if there were some educational materials put out there about what to look for in given scenarios…There are probably only four or five such scenarios if you begin to count them down, really. (XXX page 6)

I don’t think … and I go to a lot of CME, I don’t think ICU CME meeting would be high on my list of attendance. (XXX page 7)

…let’s do a faculty meeting…I suppose you could title it “What does a GP need to know about ICU outcomes and the effect on their patients”. (XXX page 6)

I think that is fairly new information, to me anyway (EL page 4) [discovery by GP about long term complications of critical illness]

…well again this is all brand new to me (XXX page 4) [about ICU complications]

definitely, yeah, definitely (XXX page 8) [to suggestion about ICU doing education sessions for GPs]

**Emergent: GP involvement in end-of-life discussions and decisions with ICU inpatients**

I can’t see what information or input that we could have that would improve the quality of end of life for a patient in Intensive Care.(XXX page 6)

… are you aware of any wishes of the patient? No problems, I’d happily take that phone call and impart the information, from anybody. But other than that, I don’t really see a role for us.(XXX page 6)

So I think our role would be more supportive rather than in the active management at that stage there. (XXX page 8)

I wouldn’t object to that actually because we would probably know those patients a long time, and I might know their wishes. (XXX page 7)

And very often the patients will talk to us, particularly if they’re elderly, you know, they’re quite happy to discuss what they want done and what they’re prepared to do. (XXX page 8)

That probably would be, that would make more sense wouldn’t it) XXX page 7) [suggestion that GPs could contribute to end of life care in ICU based on patient’s wishes]

**Emergent: Other comments or points to include or to code (including current provision of discharge information to GPs)**

Two reasons why GPs don’t hear much about ICU part of hospital stay; first, there is seldom a direct referral link between GP and patient going to or coming from ICU(“other steps along the way”)and second, the patients “know very little about it themselves”. (XXX page 1)

Third reason is the weak position relatives are in; “Their relatives would know they were there but often wouldn’t have much detail on what was going because it kind of fazes relatives, as you can understand; they’re very high tech environments. So people don’t quite know what’s going on.” (XXX page 1)

But like you don’t want three daughters and two sons all arriving having a conversation about it when the information we have is fairly sparse. (XXX page 6) [concept of being door-stepped by relatives when a GP is under-equipped with info about ICU stay]

*…or getting them back to hospital to some degree to see psychiatry or psychology.* Yeah. And that is, without spending the rest of the afternoon talking to you, that is immensely difficult because those services are basically almost unavailable to people who can’t afford to pay for them. (XXX page 6) [difficulty expressed about dealing with psychological issues post-ICU in the absence of resources to do so]

…the post traumatic stress, in terms of having services available, they are not there really and they should be. (XXX page 7)

…it would probably have to be made available at secondary care level that they go back to a psychologist attached to the hospital or else that our services are very much enhanced but that is a whole other debate basically. *But at least you are alert, at least there is a red flag on your side to say this is a problem, to enable you to try and refer back.* Yes. (XXX page 7)

But we are kind of out of that loop. (XXX page 9) [the loop of intra-hospital referrals between ICU and non-ICU teams]

…they *(patients)* tend to turn up anyway because they have a prescription or they need a cert or there is some issue. But sometimes they turn up and you know they have had big surgery but you don’t actually know they have been in Intensive Care, which is a shortcoming. (XXX page 9) [issue of being door-stepped by patient post-ICU]

More often than not it’s illegible because it’s handwritten and quite frequently they don’t even have appropriate prescriptions attached (XXX page 1) [regarding discharge letter patient has with them from hospital]

Frequently I would only get a letter after they’ve been back to an outpatient clinic and then it’s a month to six weeks after they’ve been to the clinic when I get the whole lot back. (XXX page 1)

It would never be on the discharge summary if they’ve spent any time in it.(XXX page 1) [in the ICU]

The point I was making to you is that Intensive Care physicians have the courtesy of handing on the information to the secondary care physician. Why doesn’t that go to the primary care physician? We’re at the coal face**,** and most of these patients end up in under your care having being referred through us…. So if you’re passing that information on to one set of colleagues, why not all sets of colleagues? (XXX page 2) [XXX agreed with the concept of an inpatient stay as a “circular event” where patient starts at and returns to the GP, therefore the info pertinent to that circular journey should return to the GP]

Everybody was just fantastic to me, but why was I in there? That’s the next question. What happened to me? Why did they put me in there? (XXX page 4) [patients respect and trust hospital staff, but often have little insight into what happened to them while they were there]

It does start in here in our surgery. (XXX page 7) [the circular patient journey to and from hospital]

Occasionally it would be put on the green discharge letter*.[the letter patient gives to GP after discharge]* That’s for the GP, that is basic information and it might have ICU sepsis or whatever, CT done, what have you but that would be very, very occasionally so it would be only when we get the detailed letter some weeks later that we would be aware of how ill someone might be.(XXX page 1)

It is usually it’s the most junior; probably the intern of the SHO does that brief discharge *[the green letter]* and may not have been involved in the care at all while they were in the ICU setting.(XXX page 1) [concept that any info about ICU is second hand from junior medical staff]

It’s terrible. It’s appalling. In an information highway that we’re in now.(XXX page 2) [concept that info should not reach GPs by patients or relatives, given the technology advances that exist for communication]

…there’s definitely, I think,…a lack of communication because ICU and … maybe not a lack of communication but a lack of documentation between ICU and the ward… (XXX page 6) [concept that primary team are underequipped to send accurate info about ICU to GPs, because ICUs don’t send that information to the primary teams]

Your patients are our patients and they go home to a community where a GP is at the focus of that community…we’re there at the start and we’re there at the end (XXX page 6)

It’s usually the patient tells me that they’ve been in Intensive Care, and then you spend time asking them about it. They wouldn’t often remember a lot about it.(XXX page 1)

…the letter either hasn’t arrived or else they’ve been given some piece of paper from the hospital that’s totally inadequate and the list of drugs then they are on. And you’re trying to figure out what exactly happened to them…(XXX page 1)

…it’s almost as if procedures done, yeah we had CTs, we had MRIs and the devil knows what, you still have no idea how your poor patient managed because it’s all about tests.(XXX page 2) [regarding current itemized letters, focused on interventions rather than diagnoses and problems]

I think sometimes communication between the ICU team and the in-patient, kind of medical or surgical team could probably be better and then from that, then the discharge summary could contain more kind of information about the patient’s stay. (Trainee in XXX page 7)

…my patient population…their…comprehension of what has happened to them in hospital is, you know, it is a scary low. (XXX page 5) [makes a case for informing GP and not relying on flawed information from patients or relatives

I’m talking about the relatives yeah; you’d hear it through the grapevine they were in ICU. (XXX page 2)

In fact we don’t get it for a month or so after. (XXX page 3) [the discharge letter]

I always kind of thought that the ICU admission was separate (GP trainee who in previous year had been a physician trainee) XXX Interesting thought that gatekeeper teams might think that ICU do send their own summaries and therefore intentionally omit this part of the patient’s stay in their summary

…so we don’t look stupid and caught in the headlights (XXX we’re there at the start and we’re there at the end) when they are approached by a family

It’s usually the patient tells me that they’ve been in Intensive Care, and then you spend time asking them about it. They wouldn’t often remember a lot about it. (XXX)

that should be communicated because that puts us on alert to say right, this was a very ill person. (XXX)

it’s almost as if procedures done, yeah we had CTs, we had MRIs and the devil knows what, you still have no idea how your poor patient managed because it’s all about tests. There’s been a huge shift, and I’m in general practice since 1984 and there’s been a huge shift towards test orientated care. (XXX) Theme about type of information received – test results rather than interpretative/diagnostic info.

That’s what we need to know, what was the confirmation of diagnosis and what is the level of care that this patient needed and what’s their after-care?(XXX).

Tell us they were there and what happened to them and what you can anticipate in the future

Set a kind of a realistic target for them so they’re not into you saying, “I’m still feeling desperate”, that there’s a reason for it. Most patients are excellent that way. If you can say to them it’s going to take a certain amount of time for recovery they will work with you on that and they will pace themselves.(XXX)

I mean, because it’s like the guy who had angina and he had all these stents put in, and he was told his heart was perfect. (XXX) (patients minsinterpreting info or being given erroneous info – GP can address that better if they have the right info)

So you already are flagged (XXX) benefit of knowing about ICU stay

with the patients when you know them a long time the fact that you’ve so much information about them you’re also able to kind of almost, I suppose the word is affirm their fears and their worries. I mean they must have been very worried in Intensive Care but couldn’t tell anybody, and maybe disorientated and didn’t – confused and thought they’d lost their mind or something, which is a big worry for older people. (XXX) Nice counselling role quote

we would often get relatives very angry with the hospital because they feel there’s no communication. It’s not that there’s not communication, it’s because they don’t hear the communication and they often don’t understand what the communication is about. (XXX) Relatives quote

that can actually, how would you say, de-escalate or kind of deflate people getting very anxious and ‘Oh I’m making a complaint for this’(XXX) concept that communication can be useful to defuse complaints relatives might have about hospital (and ICU) care

you could have somebody coming in being very demanding and that demand is related to the fact that they’re worried about something else. So you are keyed in to that.(XXX) being on alert about relative having someone in ICU

very often if a patient of mine dies in hospital I mightn’t hear, I mightn’t hear for weeks that somebody died and that’s disastrous.(XXX) embarrassing issue avoided if GPs are informed promptly about patient death

If you know a patient has been seriously ill or life threatening situation, they do become a more special patient for a while I might note it; there is no fixed policy but I might let others know if somebody was very ill or if they would be needing something (XXX) vagueness of response to information

I don’t have a rigid policy about contacting them, it just depends.(XXX) vagueness again

So I am not sure if the ICU contacting the GP directly when the patient might be in hospital for another three months, there might be a lot of information that would come through after. But maybe some generic information like myopathy or different psychological complications would be really useful at the end of the discharge summary for the GP, yeah. (XXX in XXX Worthwhile point that ICU info may be redundant after a long hospital stay post-ICU discharge thought some basic general information still of value probably

I think sometimes communication between the ICU team and the in-patient, kind of medical or surgical team could probably be better and then from that, then the discharge summary could contain more kind of information about the patient’s stay.(XXX in XXX) Interesting point that gatekeeper summaries could improve if ICU-hospital team summaries improved. Still, this wouldn’t address the “dorrstepping” phenomenon.

but it goes in one ear and out the other (XXX) Info given to patients…

Third hand information is lousy, they never get it right. Always misinterpret it.(XXX)

He has very little recollection of it. When he came home he was home for two or three days and he comes into me to let me know what went on or to find out did I know what went on. I had no information until he went back to the clinic, he came home on Warfarin and his partner was able to tell me that he had a clot in his leg. It was only when I got the discharge summary from the first outpatient visit that it was found out the exact details of that. So for like three months I’m shooting in the dark (XXX) Good case study after hospital discharge which included ICU stay for 10days.

Mother and wife came in and long faces and I didn’t know what had happened there and had I not heard the news of the catastrophic brain injury peri-operatively one week before?(XXX) another case study

there’s the perception that you don’t care…it’s someone you’ve looked after for multitudes of years or for many conditions but you’re just unaware that they’re in (ICU). (XXX) Issue about professionalism

Well, I suppose there there’s one being more empathetic perhaps regarding an active management of their condition. (XXX) Empathy theme.

But there’s definitelya lack of communication…maybe a lack of documentation between ICU and the ward (XXX in XXX) Concept of improving info from ICU to gatekeeper.

I’ll then phone the family and say can I do anything, can I help with anything? And I think just the mere fact of saying that is a huge help. (XXX) Vague general offer of assistance to patients ater hospital d/c

We’re looking at the whole package and the ramifications there, psychologically, children minding wise, you know, work wise et cetera (XXX) concept that GP juggles lots of different priorities in the care of patients.

You’d probe a little bit more closely and probably be a little bit more proactive (XXX) Vagueness using information

It would be flagged in my head but now in my mind in some way or other well OK they were there but from now on I suppose I best be a bit more careful. I’ll watch them closely, you know. (XXX) Vagueness of information

If someone has been in ICU, their morbidity, their mortality is increased and all of this craic so you need to be a little bit more fine tuned perhaps,(XXX) Vagueness of response to information received

**LEGEND**

FICP: Factors influencing current practice

PBIS: Perceived benefits of current practice

SOIS: Strategies for optimal information sharing

Emergent: emergent themes
